# A Bayesian model for assessing organic matter supply in complex marine food webs using amino acid stable isotope analysis

**DOI:** 10.7717/peerj.20220

**Published:** 2025-11-19

**Authors:** Connor H.H. Shea, Jeffrey C. Drazen, Brian N. Popp

**Affiliations:** 1 Department of Oceanography, University of Hawaii at Manoa, Honolulu, HI, United States of America; 2 Department of Earth Science, University of Hawaii at Manoa, Honolulu, HI, United States of America

**Keywords:** AA-CSIA, Mesopelagic, Trophic discrimination, Trophic ecology, Mixing model, Zooplankton, Protist

## Abstract

While several software packages have been developed to solve stable isotope mixing models, none are currently equipped to trace the flow of organic matter through the lower trophic levels of planktonic food webs. To address this gap, we have developed a new Bayesian mixing model tailored for use with δ^15^N values of individual amino acids. This model simultaneously estimates trophic relationships between consumers and organic matter sources at the base of the food web, determines the relative contributions of these basal organic matter sources to consumers, and accounts for trophic discrimination affecting amino acid δ^15^N values during protozoan and metazoan trophic steps. This “Organic Matter Supply Model” is uniquely suited for applications where food web structure is unknown and trophic intermediaries, such as protozoan and metazoan grazers with distinct amino acid trophic discrimination factors, play a critical role in nutrient transfer. In this paper, we describe the model’s basic structure, outline key considerations for adapting it to specific applications, evaluate its performance using simulated zooplankton data, discuss its strengths and limitations, and offer recommendations for its further development. By testing the model on simulated zooplankton amino acid δ^15^N data, we demonstrate that the Organic Matter Supply Model can enhance our understanding of the roles of small particles and diel vertical migration in deep-sea organic matter supply pathways. Furthermore, it provides a new framework for exploring the foundational role of heterotrophic protists in marine ecosystems. We find specific subsets of amino acids to be most useful as markers of trophic ecology (in this case including glutamic acid and proline) and to identify supply from basal organic matter sources (phenylalanine, lysine, and threonine). Other amino acids may be more ideal source tracers in other settings, although amino acids with inconsistent or poorly constrained isotope fractionation behavior (*e.g.*, isoleucine, valine) should be excluded to optimize model reliability.

## Introduction

Stable isotope data have been widely used in diet tracing studies, where the isotopic composition of a consumer and its food are compared to estimate relative contributions of each food item to the consumer’s diet (Nielsen et al., 2018). These studies often require multiple isotope tracers to adequately constrain contributions from different dietary sources. The most effective approaches use tracers that are independent (uncorrelated) and sufficient in number, requiring at least n-1 tracers for n possible dietary sources. Additionally, the isotopic composition of dietary items often changes upon incorporation into consumer tissues, a process known as trophic discrimination (Post, 2002). This must be accounted for by applying empirically determined trophic discrimination factors (TDFs or Δ^15^N values; Peterson & Fry, 1987), which quantify the change in isotopic composition with each trophic transfer.

Several models have been developed to address these types of mixing problems (IsoSource, MixSIR, SIAR, MixSIAR, simmr), with MixSIAR (Stock et al., 2018) and simmr (Govan et al., 2023) being the most comprehensive and widely used tools in isotope-based diet studies. Both packages use Bayesian statistical analysis and can incorporate multiple isotopic values (as well as additional tracers) to estimate dietary contributions to a consumer. They also include robust methods for propagating uncertainty in tracer values of both source and consumer populations and can incorporate TDFs to account for trophic discrimination between diet and consumer tissues. MixSIAR, simmr, and similar models have made sophisticated Bayesian mixing models more accessible to the ecological research community (García-Seoane, Viana & Bode, 2023; Chang et al., 2023).

Available tools are generally designed to model simple consumer-food relationships involving a single trophic transfer. However, studies aiming to trace nutrients through more complex food webs may find challenges adapting existing isotope mixing models, particularly when the number of trophic transfers is unknown or when dietary sources occupy different trophic positions (TP). Recent work has applied amino acid compound-specific nitrogen isotope analysis (AA-CSIA) to investigate the relative contributions of various nutrient sources to marine food webs (Gloeckler et al., 2018; Hannides et al., 2020; Shea et al., 2023). In these systems, zooplankton or fish may acquire carbon and nitrogen from multiple sources of particulate organic matter (POM), ranging from fresh phytoplankton with a TP of 1 to microbially degraded fecal matter with a TP as high as 3. While this POM can be consumed directly, it is often first assimilated into the biomass of protozoans and/or metazoans, which are then consumed by the study organisms.

Collectively, these characteristics introduce several complicating factors that are not explicitly accounted for in currently available models:

1. Multiple trophic transfers may separate consumers from dietary sources at the base of the food web.
2. These trophic transfers may include both protozoan and metazoan steps, which can lead to different amounts of trophic nitrogen isotope discrimination among amino acids (Gutiérrez-Rodríguez et al., 2014).
3. The number of protozoan and metazoan trophic steps, and thus the amount of trophic discrimination, depends on which dietary sources are utilized.

Although not ideal, points 1 and 2 can be addressed in packages like MixSIAR by redefining TDFs and their associated uncertainties to reflect isotope fractionation occurring over multiple trophic transfers. Point 3, however, presents a more complex issue. To illustrate this, consider a copepod with a TP of 3. Its food web could be based on one of several sources: phytoplankton (TP = 1), fecal pellets (TP = 2), or small microbially degraded particles (TP = 2). This means that, depending on the organic matter source, the food web spans one to two trophic transfers. Now, suppose we can fully differentiate these three sources using the δ^15^N values of phenylalanine and threonine, and that phenylalanine and threonine exhibit 0.5‰ and −5‰ fractionation per metazoan trophic step, respectively. To solve the mixing model, we must account for trophic fractionation. However, to account for trophic fractionation, we first need to know the length of the food web, which, as noted, depends on the source of organic matter (*i.e.,* the solution to the mixing model itself!). In other words, the mixing equations and trophic discrimination equations are interdependent and must be solved simultaneously. This type of problem falls outside the scope of existing Bayesian models (Moore & Semmens, 2008; Stock et al., 2018; Govan et al., 2023).

In response to these limitations, we have developed a Bayesian mixing model (the Organic Matter Supply Model or OMSM) tailored for use with δ^15^N values of individual amino acids which simultaneously:

(a) Estimates the number of metazoan and protozoan trophic steps between a consumer and organic matter sources at the base of the food web;
(b) Solves for the relative contributions of basal organic matter sources to the consumer; and
(c) Accounts for trophic discrimination affecting amino acid δ^15^N-based tracers during protozoan and metazoan trophic steps.

While unnecessarily complex for straightforward diet-tracing problems, our model represents a significant departure from MixSIAR. It is uniquely suited for applications in which food web structure is unknown and intermediate trophic steps, such as protozoans and metazoans with distinct amino acid TDFs, play a critical role in nutrient transfer. Though the OMSM is optimized for use with amino acid δ^15^N values, it is flexible and could be used with other types of tracers as well (*e.g.*, bulk isotopic compositions, amino acid δ^13^C values).

This paper describes the fundamental structure of the OMSM and outlines key considerations for adapting it to specific applications. We begin with a generalized overview of the model, detailing its structure, equations, and parameters. The model is then tailored to investigate the supply of POM to a mesopelagic zooplankton food web using δ^15^N values of amino acids. In this context, we describe the data and methods used for model adaptation, explaining the rationale behind specific modelling choices, and evaluate performance using simulated zooplankton data. Finally, we discuss the model’s strengths and limitations, and offer recommendations for its further development, choices of specific amino acids, and application in future research.

## Organic Matter Supply Model

### Model description

**Figure 1:**
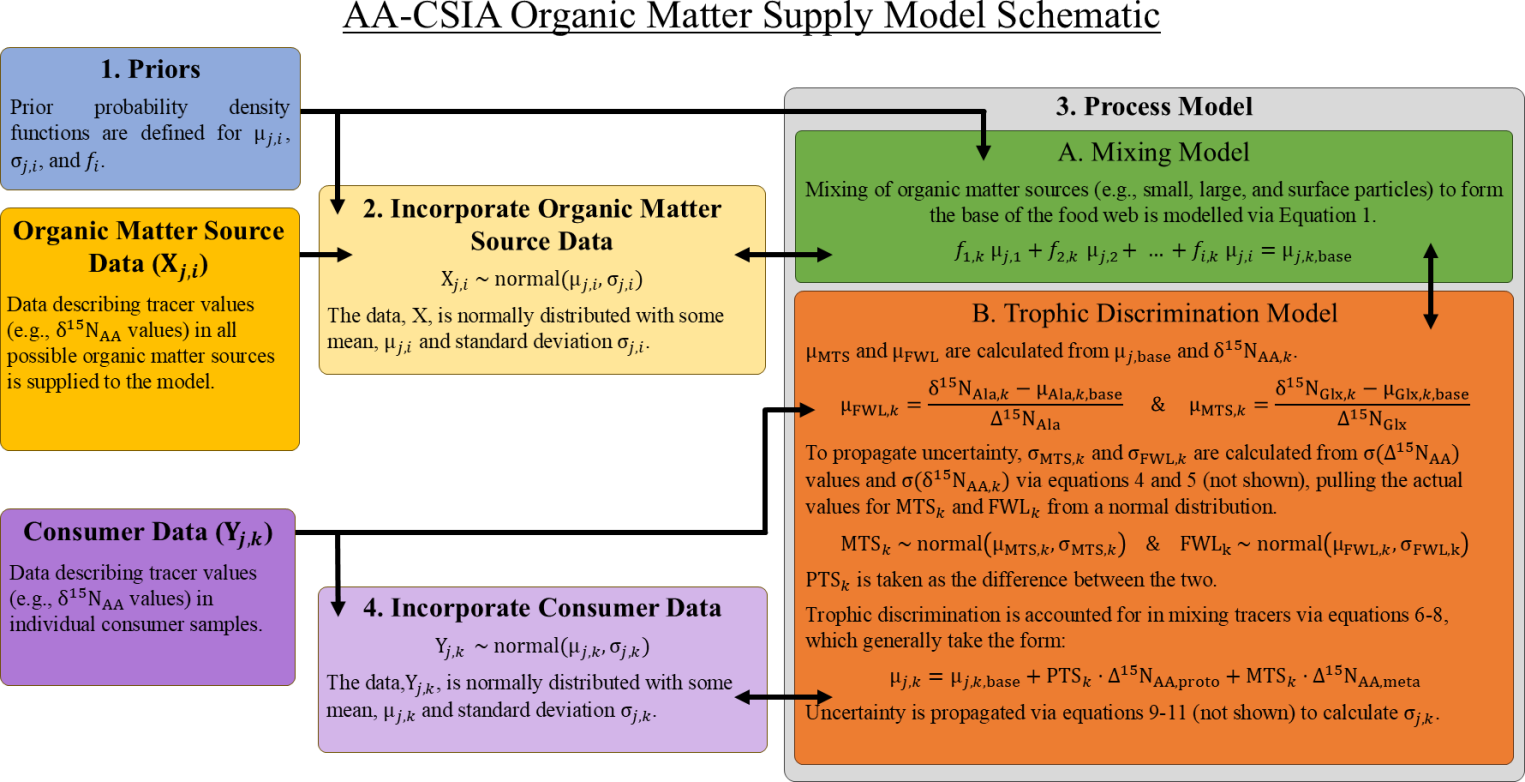
Basic structure and relationships comprising the organic matter supply model. Greek letters μ and *σ* represent the mean and standard deviation of the indicated quantity. See Table 1 for a complete description of all parameters.

Food webs are complex networks in which organic matter is assimilated from basal resources and transferred across trophic levels in a series of predator–prey interactions. When considering the origin of the organic matter contained within an individual organism, various possible supply pathways with distinct sources can be envisioned. The OMSM described here uses a Bayesian framework designed to identify the basal source(s) of organic matter to an organism (or group of organisms) while also quantifying the number of trophic steps involved in the supply pathway. It achieves this by modeling the relationships between the isotopic compositions of amino acids measured in consumers (Y_*j*,*k*_) and those measured in potential organic matter sources (X_*j*,*i*_), where *j* represents a specific tracer (*e.g.*, the δ^15^N value of an amino acid), *i* a possible organic matter source, and *k* an individual consumer sample. The model consists of four main components, each described in detail below (Fig. 1).

1. Designation of prior probability density functions (priors) for model parameters (*e.g.*, mixing coefficients)
2. Incorporation of organic matter source data, X_*j*,*i*_
3. Process model
  (A) Linear mixing model
  (B) Trophic discrimination model
4. Fitting of the model to consumer data, Y_*j*,*k*_

Various parameters are specified or estimated within the model to represent the trophic structure of the food web, the relative contributions of organic matter sources, and the behavior of isotopic tracers within the system (Table 1). Markov Chain Monte Carlo simulation (MCMC) is used to explore the parameter space and identify the most probable parameter sets based on the observed data and defined prior information. Importantly, this approach allows the model to identify the breadth of possible and likely solutions, even when the mixing model is underdetermined and a single precise solution does not exist (Phillips & Gregg, 2003). The output of the MCMC is a set of posterior probability density functions, referred to hereafter as “posteriors,” which represent the most likely values for each parameter (Table 1).

**Table 1:** Names and descriptions of relevant model parameters.

Parameter	Name/Description	Fixed/Varied
μ_*j*,*i*_/μ_*j*,*k*_	The average isotopic composition of some tracer (*j*) in an organic matter source (*i*) or consumer sample (*k*).	Varied
*σ*_*j*,*i*_/*σ*_*j*,*k*_	The standard deviation of some tracer (*j*) in an organic matter source (*i*) or consumer sample (*k*).	Varied
*f*_*i*,*k*_	The mixing coefficient, or fractional contribution of some organic matter source (*i*) to the organic material in a consumer (*k*).	Varied
MTS_*k*_	Number of metazoan trophic steps for a consumer sample (*k*).	Varied
PTS_*k*_	Number of protozoan trophic steps for a consumer sample (*k*).	Varied
FWL_*k*_	Total number of trophic steps, or food web length for a consumer sample (*k*).	Varied
μ_MTS,*k*_/μ_FWL,*k*_	The average value for the designated trophic parameter for a consumer sample (*k*).	Varied
*σ*_MTS,*k*_/*σ*_FWL,*k*_	The standard deviation of the designated trophic parameter for a consumer sample (*k*).	Varied
Δ^15^N_AA_	The trophic discrimination factor for a given amino acid (AA)	Fixed
*σ*(Δ^15^N_AA_)	The standard deviation of the trophic discrimination factor for a given amino acid (AA)	Fixed
*α*_*j*,*k*_	The analytical uncertainty associated with the measurement of some tracer (*j*) in a consumer sample (*k*).	Fixed

Part 1 – Prior Information: Prior probability density functions are a necessary component of all Bayesian models and must be specified for all unknown parameters in the model. Priors are often defined to be intentionally vague, but they can also be used to bias the model towards or against specific solutions (*e.g.*, specific mixing proportions) based on available knowledge that is independent of the main data inputs (*e.g.*, the relative abundance of dietary sources in the environment or findings from stomach content analysis). While the default behavior of the OMSM is to use minimally informative priors (as described in Section ‘Choice of Tracers and Organic Matter Source Separation’), informative priors can be specified for mixing coefficients for each organic matter source (*f*_*i*_) as in previous Bayesian mixing models (Moore & Semmens, 2008; Stock et al., 2018), as well as for the mean (μ_*j*,*i*_) and standard deviation (*σ*_*j*,*i*_) of each tracer within each organic matter source group. The construction of informative priors closely follows procedures used for MixSIAR and is thoroughly discussed by Stock et al. (2018). Unlike MixSIAR and other models, priors cannot be defined for trophic parameters (*e.g.*, food web length, protistan trophic steps) due to the way that they are explicitly calculated within the model.

Part 2 – Organic Matter Source Data Incorporation: The model is provided with tracer values (here δ^15^N values of individuals amino acids) for samples representing each potential organic matter source. It assumes that the frequency of tracer values for each source can be described by a normal distribution characterized by a mean (μ_*j*,*i*_) and standard deviation (*σ*_*j*,*i*_). It is essential that sufficient data are available to accurately characterize the natural variability within each organic matter source. During the MCMC, the values for the mean (μ_*j*,*i*_) and standard deviation (*σ*_*j*,*i*_) of source tracer values are allowed to vary, approximating the variability contained in the organic matter source data. As a result, the model inherently incorporates natural variability in tracer values for each organic matter source without requiring explicit propagation of *σ*_*j*,*i*_ through subsequent equations. An example workflow for the selection of organic matter source data is presented in Section ‘Determining Possible Sources of Organic Matter’.

Part 3 – Process Model: The process model consists of two components: a linear isotope mixing model and a trophic discrimination model. Distinct sets of tracers are used to solve mixing equations (mixing tracers) and trophic equations (trophic tracers), requiring independent selection of the tracer sets best suited to quantify each process. To maintain the integrity of the model, there must be no overlap between mixing and trophic tracer sets.

The mixing model (3A in Fig. 1), which is similar to Bayesian mixing models implemented in other studies (Stock et al., 2018; Govan et al., 2023), describes how potential sources of organic matter combine to form the base of the food web. While this mixture of organic matter sources is often referred to as the “base” of the food web, we acknowledge that it may include detrital or non-algal material that does not strictly have a trophic position of 1. The model assumes linear mixing dynamics, such that the isotopic composition for any amino acid at the base of the food web can be estimated as:

(1) \begin{eqnarray*}{\mathrm{\mu }}_{j,k,\text{base}}=\sum _{\mathrm{all}~i}{f}_{i,k}{\mathrm{\mu }}_{j,i}={f}_{1,k}{\mathrm{\mu }}_{j,1}+{f}_{2,k}{\mathrm{\mu }}_{j,2}+\ldots \end{eqnarray*}

where the mixing coefficient, *f*_*i*,*k*_, represents the fractional contribution of organic matter sources, *i*, to the base of the food web for an individual consumer, *k*, and μ_*j*,base_ is the average value of tracer, *j*, at the base of the food web. While mixing coefficients (here *f*_*i*,*k*_) are the only unknowns in a typical mixing model, in the OMSM the tracer values at the base of the food web (*i.e.,* “the mixture” or μ_*j*,base_) are also unknown. The OMSM addresses this additional unknown by relating μ_*j*,base_ to tracer values measured in zooplankton *via* the trophic discrimination model.

The trophic discrimination model (3B) describes how the values of mixing tracers change, or are conserved, during trophic transfer. To implement this, trophic tracers are first used to estimate three key variables: the total food web length (FWL), the number of protistan trophic steps (PTS), and the number of metazoan trophic steps (MTS). Controlled feeding studies of zooplankton suggests that certain amino acids, such as the δ^15^N value of alanine (δ^15^N_Ala_), exhibit a relatively constant rate of trophic discrimination across both metazoan and protozoan trophic steps (Décima et al., 2017). This discrimination is quantified using a trophic discrimination factor (Δ^15^N_AA_), which represents the increase in δ^15^N_AA_ per trophic level (McClelland & Montoya, 2002; Chikaraishi et al., 2009; Bradley et al., 2015). By comparing the δ^15^N_Ala_ value measured in a consumer sample (δ^15^N_Ala,__*k*_) with the mean value at the base of the food web (μ_Ala,*k*,base_), the total food web length for that sample can be estimated using the following equation:

(2) \begin{eqnarray*}{\mathrm{\mu }}_{\mathrm{FWL},k}= \frac{{\mathrm{\delta }}^{15}{\mathrm{N}}_{\mathrm{Ala},k}-{\mathrm{\mu }}_{\mathrm{Ala},k,\text{base}}}{{\Delta }^{15}{\mathrm{N}}_{\mathrm{Ala}}} \end{eqnarray*}

where Δ^15^N_Ala_ is the trophic discrimination factor for δ^15^N_Ala_. Alternatively, controlled feeding studies suggest that other amino acids (*e.g.*, δ^15^N in glutamic acid/glutamate, or δ^15^N_Glx_) only exhibit significant trophic discrimination during metazoan trophic steps (Gutiérrez-Rodríguez et al., 2014). Following the same logic used to estimate FWL, we can estimate metazoan trophic steps using the following equation:

(3) \begin{eqnarray*}{\mathrm{\mu }}_{\mathrm{MTS},k}= \frac{{\mathrm{\delta }}^{15}{\mathrm{N}}_{\mathrm{Glx},k}-{\mathrm{\mu }}_{\mathrm{Glx},k,\text{base}}}{{\Delta }^{15}{\mathrm{N}}_{\mathrm{Glx},\text{meta}}} \end{eqnarray*}

where Δ^15^N_Glx,meta_ is the trophic discrimination factor for δ^15^N_Glx_ specific to metazoan trophic steps. Other tracers with comparable fractionation dynamics (*i.e.,* those that exhibit constant trophic discrimination for estimating FWL or variable trophic discrimination across trophic groups for estimating MTS) can be substituted in these equations in place of δ^15^N_Ala_ or δ^15^N_Glx_, provided the appropriate Δ^15^N_AA_ values are applied (see Table 2 and evaluation of TDFs below). An example workflow for the selection of Δ^15^N values are given in Section ‘Determination of δ^15^N_AA_ values’, while considerations and specific guidance for the selection of trophic tracers are given in Section ‘Choice of tracers and organic matter source separation’.

**Table 2:** Average Δ^15^N values determined from controlled feeding studies and regression analysis. Averages Δ^15^N_AA_ values from the feeding studies included in our data compilation were determined for each amino acid across all experiments, only metazoan taxa, and only protozoan taxa (ciliates and dinoflagelates). Δ^15^N_AA_ values that were significantly different (*p* < 0.05) or marginally different (0.05 < *p* < 0.1) in protozoa relative to metazoa are indicated with ** or * respectively. Not all amino acids were reported from all experiments, with Thr and Lys being frequently omitted. Data included in the compilations are described in Section ‘Determination of Δ^15^N_AA_ values’. Values from regression analysis were determined by examining relationships between Δ^15^N_AA_ values and TP in wild zooplankton and particles, also described in Section ‘Determination of Δ^15^N_AA_ values’. Recommended δ^15^N values are indicated in bold, as are the amino acids that are included as tracers when the model is adapted for assessing mesopelagic organic supply at station ALOHA.

ALL UNITS: ‰	Glx	AsX	Ala	Ile	Leu	Pro	Val	Gly	Ser	Phe	Lys	Thr
All (*n* = 21)	7 ± 3.1	4.4 ± 2.8	**6.3 ± 2.6**	4.6 ± 3.2	5 ± 2.7	**5.8 ± 1.7**	3.9 ± 2.8	**2.9 ± 3.1**	**2.6** ± **3.2**	**0.3 ± 0.5**	**1.2** ± **1.2**	−4.9 ± 3.5
Metazoa (*N* = 18)	**8** ± **1.7**	**5.7 ± 1.9**	6.4 ± 2.7	5.5 ± 2.3	**5.6** ± **2.4**	6.1 ± 1.6	4.4 ± 2.6	2.7 ± 3.2	3.2 ± 2.8	0.3 ± 0.5	1 ± 1.3	−5.9 ± 3.6
Protozoa (*N* = 3)	**0.5** ± 1**	0.8 ± 1.4**	6.3 ± 1.5	−0.5 ± 2.7*	**1.4** ± 0.6**	4.3 ± 1.5	0.7 ± 1.6*	3.8 ± 1.3	−0.7 ± 3.7	0.3 ± 0.6	1.7 ± 0.5	**−2** ± 0.6**
Regression analysis		6.2 ± 1.2			4.9 ± 2	4.2 ± 1.7		3.4 ± 1	3.7 ± 1.5		1.2 ± 0.5	**−5.9** ± **1.5**

While uncertainty in μ_*j*,base_ is inherently incorporated into the model output, analytical uncertainty in δ^15^N_Ala/Glx,k_ and experimental uncertainty in trophic discrimination factors (Δ^15^N_Ala/Glx_) must be explicitly propagated through Eqs. (2) and (3) to our estimates of food web length (FWL_*k*_) and metazoan trophic steps (MTS_*k*_). Using standard methods of error propagation, we can estimate the uncertainty in trophic parameters *via* the equations:

(4) \begin{eqnarray*}{\sigma }_{\mathrm{FWL},k}& ={\mathrm{\mu }}_{\mathrm{FWL},k}\cdot \sqrt{{ \left( \frac{\sigma \left( {\mathrm{\delta }}^{15}{\mathrm{N}}_{\mathrm{Ala},k} \right) }{{\mathrm{\delta }}^{15}{\mathrm{N}}_{\mathrm{Ala},k}} \right) }^{2}+{ \left( \frac{\sigma \left( {\Delta }^{15}{\mathrm{N}}_{\mathrm{Ala}} \right) }{{\Delta }^{15}{\mathrm{N}}_{\mathrm{Ala}}} \right) }^{2}}\end{eqnarray*}

(5) \begin{eqnarray*}{\sigma }_{\mathrm{MTS},k}& ={\mathrm{\mu }}_{\mathrm{MTS},k}\cdot \sqrt{{ \left( \frac{\sigma \left( {\mathrm{\delta }}^{15}{\mathrm{N}}_{\mathrm{Glx},k} \right) }{{\mathrm{\delta }}^{15}{\mathrm{N}}_{\mathrm{Glx},k}} \right) }^{2}+{ \left( \frac{\sigma \left( {\Delta }^{15}{\mathrm{N}}_{\mathrm{Glx}} \right) }{{\Delta }^{15}{\mathrm{N}}_{\mathrm{Glx}}} \right) }^{2}}\end{eqnarray*}

where *σ* represents the standard deviation associated with the indicated quantity. This uncertainty is incorporated into the model by drawing posterior values for FWL_*k*_ and MTS_*k*_ from normal distributions, with means defined by Eqs. (2) and (3), and standard deviations defined by Eqs. (4) and (5). The number of protistan trophic steps (PTS_*k*_) is then calculated as the difference between FWL_*k*_ and MTS_*k*_.

Now that we have used our trophic tracers to estimate PTS, MTS, and FWL, we can account for trophic discrimination in the mixing tracers between the base of the food web and consumers. For some tracers we can assume no trophic fractionation (termed conservative tracers; *e.g.*, δ^15^N values of certain “source” amino acids (Popp et al., 2007) or, if used, δ^13^C values of essential amino acids (Fantle et al., 1999), yielding the simple equation:

(6) \begin{eqnarray*}{\mathrm{\mu }}_{j,k}={\mathrm{\mu }}_{j,k,\text{base}}\end{eqnarray*}

where μ_*j*,*k*_ is the mean tracer value for some consumer sample. For δ^15^N_AA_ tracers where fractionation occurs identically during meta- and protozoan metabolism (termed constant trophic discrimination tracers; *i.e.,* Δ^15^N_AA,meta_ = Δ^15^N_AA,proto_), trophic discrimination can be accounted for uniformly throughout the food web *via* the equation:

(7) \begin{eqnarray*}{\mathrm{\mu }}_{j,k}={\mathrm{\mu }}_{j,k,\text{base}}+{\mathrm{FWL}}_{k}\cdot {\Delta }^{15}{\mathrm{N}}_{\mathrm{ AA},\text{meta}}.\end{eqnarray*}

Recognizing that, for some amino acids, isotope fractionation occurs differently during metazoan or protozoan metabolisms (termed variable trophic discrimination tracers; *i.e.,* Δ^15^N_*j*,meta_ ≠ Δ^15^N_*j*,proto_), fractionation occurring during meta- *vs* protozoan trophic steps is differentiated, yielding the equation:

(8) \begin{eqnarray*}{\mathrm{\mu }}_{j,k}={\mathrm{\mu }}_{j,k,\text{base}}+{\mathrm{PTS}}_{k}\cdot {\Delta }^{15}{\mathrm{N}}_{\mathrm{ AA},\text{proto}}+{\mathrm{MTS}}_{k}\cdot {\Delta }^{15}{\mathrm{N}}_{\mathrm{ AA},\text{meta}}.\end{eqnarray*}

While variance in μ_*j*,base_, PTS, MTS, and FWL affecting μ_*j*,*k*_ is inherently accounted for in μ_*j*,*k*_ due to the model’s structure, uncertainty in trophic discrimination factors (Δ^15^N_AA_) still needs to be propagated through Eqs. (7) and (8). This uncertainty is then combined with analytical uncertainty in tracer measurements from consumer samples (hereon referred to as *α*_*j*,*k*_). Using standard error propagation methods, we can estimate total uncertainty in the mean consumer tracer values (μ_*j*,*k*_) with the following equations:

(9) \begin{eqnarray*}{\sigma }_{j,k}& ={\alpha }_{j,k}\end{eqnarray*}

(10) \begin{eqnarray*}{\sigma }_{j,k}& =\sqrt{{\alpha }_{j,k}^{2}+{ \left( {\mathrm{FWL}}_{k}\cdot \sigma ({\Delta }^{15}{\mathrm{N}}_{\mathrm{AA},\text{meta}}) \right) }^{2}}\end{eqnarray*}

(11) \begin{eqnarray*}{\sigma }_{j,k}& =\sqrt{{\alpha }_{j,k}^{2}+{ \left( {\mathrm{PTS}}_{k}\cdot \sigma ({\Delta }^{15}{\mathrm{N}}_{\mathrm{AA},\text{proto}}) \right) }^{2}+{ \left( {\mathrm{MTS}}_{k}\cdot \sigma ({\Delta }^{15}{\mathrm{N}}_{\mathrm{AA},\text{meta}}) \right) }^{2}}\end{eqnarray*}

where Eqs. (9), (10) and (11) pertain to conservative (Eq. (6)), constant trophic discrimination (Eq. (7)), and variable trophic discrimination (Eq. (8)) tracers, respectively. Tracers are categorized into these three groups based on whether statistically significant differences between trophic discrimination factors in metazoans (Δ^15^N_AA,meta_) and protozoans (Δ^15^N_AA,proto_) have been observed in the literature (as in Section ‘Statistical Tests’), or if the tracer can be justifiably assumed to behave conservatively.

When the process model equations are combined by substituting Eq. (1) into Eq. (8), we obtain the full process model equation:

(12) \begin{eqnarray*}{\mathrm{\mu }}_{i,k}=\sum _{\mathrm{all}i} \left[ {f}_{i,k}{\mathrm{\mu }}_{j,i} \right] +{\mathrm{PTS}}_{k}\cdot {\Delta }^{15}{\mathrm{N}}_{\mathrm{ AA},\text{proto}}+{\mathrm{MTS}}_{k}\cdot {\Delta }^{15}{\mathrm{N}}_{\mathrm{ AA},\text{meta}}.\end{eqnarray*}

Inspecting Eq. (12) clarifies that the only true unknowns in the process model are the mixing coefficients, MTS, and PTS. This suggests that n sources + 1 independent tracers are required to fully constrain the model.

Part 4 – Model Fit: Results from model equations (*i.e.,* the mean consumer tracer values, μ_*j*,*k*_) are compared with the consumer data (Y_*j*,*i*_) to assess how well the model parameters fit the observations. We assume that the tracer data in consumers (Y_*j*,*i*_) follow a normal distribution with a mean (μ_*i*,*k*_, Eq. (12)) and standard deviation (*σ*_AA,*k*_, Eqs. (9)–(11)).

The output of the model is a set of posterior probability density functions (posteriors), which describe the most likely values for each model parameter (Table 1), given the data, the model equations, and priors. The model uses a Gibbs sampling protocol *via* the JAGS software package (Plummer, 2023) to identify the parameter values that produce the best model fit. Model posteriors are summarized by calculating the posterior mean and mode, as well as 50%, 75%, 90%, and 95% highest density intervals (HDIs), which represent the smallest interval that contains the respective percentage of posterior values. The mode, representing the peak of the posterior distribution, is the single most likely model solution, though all solutions within the 95% HDI range are considered reasonable and mathematically plausible solutions.

It is important to highlight the key differences between this model compared with other Bayesian mixing models. First, the number and nature of trophic levels comprising the food web (FWL, PTS, and MTS) is estimated within the model, whereas earlier models assume a single trophic transfer or can be forced to fit a defined number of trophic transfers. Second, this model explicitly differentiates between metazoan and protozoan trophic steps, allowing for each to be characterized by distinct Δ^15^N_AA_ values. This distinction is a critical advancement that enables more accurate modeling of trophic discrimination in the lower levels of planktonic food webs. While these features significantly expand the model’s applicability, they also increase the degrees of freedom, which can result in greater uncertainty and broader credible intervals in the model estimates.

### OMSM implementation

The OMSM is implemented within a single R Markdown document that utilizes several functions defined in accompanying R scripts. The R Markdown guides the user through data importation, assessment of data properties, specification of model parameters, execution of the model, and analysis and visualization of the model output. The document and code are written in a general format, allowing them to be adapted to the specific application of other users. The OMSM itself is written in the BUGS (Thomas, 2006) and executed using the R implementation of the Bayesian analysis software JAGS (Plummer, 2023). All model code and dependencies are publicly available in a GitHub repository (https://github.com/CH-Shea/AA-CSIA-Organic-Matter-Supply-Model with DOI: https://doi.org/10.5281/zenodo.15548791).

The model requires three main data inputs. First, it uses tracer values measured in multiple samples from each potential organic matter source group. While the model can technically run with only two samples per group, three or more samples are preferred for more robust results. Second, it requires tracer values and associated measurement uncertainties for at least one consumer sample. If multiple consumer samples are included, the model fits separate mixing and trophic discrimination models for each sample. Finally, the model requires estimates for trophic discrimination factors for each tracer affected by trophic discrimination, along with their associated uncertainties. For tracers designated as having constant fractionation dynamics (following Eq. (7)), a single trophic discrimination factor is used (Δ^15^N_*j*,meta_). For tracers with variable fractionation dynamics (following Eq. (8)), separate trophic discrimination factors are needed for protozoan (Δ^15^N_*j*,proto_) and metazoan (Δ^15^N_*j*,meta_) trophic steps. Recommended values for these parameters in marine planktonic ecosystems are given in Table 2.

### OMSM interpretation

Because the outputs of the model are probability density functions (*i.e.,* posteriors), the results must be interpreted probabilistically. Considering probabilistic intervals, such as highest density functions (*e.g.*, HDIs), along with the distribution mode provides a convenient framework for hypothesis testing. The null hypothesis should be tailored to the specific research question. For example, if the question is: “Does surface POM contribute to mesopelagic organic matter supply?”, the corresponding null hypothesis would be “surface POM does not contribute to organic matter supply.” This hypothesis could be rejected with 95% confidence if the 95% HDI excludes zero. Regardless of this outcome, the lower and upper bounds of the HDI indicate the minimum and maximum proportional contribution of that source to the consumer food web, while the mode represents the most likely value. A similar logic applies to trophic parameters. For example, the HDI for PTS or MTS must exceed zero to confidently infer that these trophic steps are present in the food web. In this case, the HDI bounds provide the minimum and maximum estimated number of trophic steps, and the mode indicates the most likely value for each parameter.

### Constraining sources of uncertainty to the OMSM

There are three main sources of uncertainty affecting the accuracy and precision of OMSM outputs:

1. Analytical uncertainty in consumer tracers values.
2. Observed uncertainty in organic matter source tracer values.
3. Observed uncertainty in tracer Δ^15^N values.

Although 2 and 3 have the greatest influence, the relative impact of each source depends on the structure of the food web being analyzed and can be assessed through careful consideration of Eq. (12).

Uncertainty in consumer tracers values (μ_*i*,*k*_) will consistently affect model precision. However, analytical uncertainty in AA-CSIA measurements is generally small (standard deviation (SD) < 1‰). Moreover, because μ_*i*,*k*_ is not multiplied by any other term in Eq. (12), this uncertainty does not compound under different model conditions. As a result, this represents a relatively minor but consistent source of model uncertainty.

Variability in organic matter source tracer values can substantially affect model precision, particularly for the sources that contribute most to organic matter supply. While the multiplicative factor (*f*_*i*,*k*_) associated with the organic matter source tracer value in Eq. (12) cannot exceed 1, within-source variability can be high (SD > 1‰), leading to significant error. Output precision can be improved by increasing sample size or reducing variance within source groups, especially for sources contributing most to organic matter supply.

Uncertainty in Δ^15^N values is another major contributor to model error, due both to high variability observed across controlled feeding experiments for many amino acids (Table 2) and to the fact that Δ^15^N values are multiplied by PTS, MTS, or FWL in Eq. (12). This means that Δ^15^N uncertainty has a greater effect as food web length increases. The most effective long-term mitigation will come from further research on amino acid fractionation in wild and laboratory organisms. In the meantime, users can reduce uncertainty by selecting tracers with lower observed variability in Δ^15^N values in the literature. Additional guidance on tracer selection and the effects of uncertainty in Δ^15^N values is provided in Section ‘Choice of Tracers and Organic Matter Source Separation’.

## Adapting the OMSM to Assess Organic Matter Supply Pathways in the Deep Sea

The OMSM is adaptable to any situation where multiple sources of organic matter need to be traced across multiple trophic levels with varying trophic discrimination factors. To evaluate model performance, we assess the ability of OMSM to diagnose relevant sources of organic matter to the mesopelagic food web at Station ALOHA using amino acid δ^15^N data.

### Methods & Data

#### AA-CSIA data

To adapt the OMSM to a specific case study, we used particle AA-CSIA data collected during a series of cruises to Station ALOHA in February 2014, August/September 2014, and May 2015, described originally in Hannides et al. (2020) and again more recently in Miller et al. (2025) (full data available at https://www.bco-dmo.org/dataset/970402 with DOI: 10.26008/1912/bco-dmo.970402.1). Briefly, size fractionated particulate matter was collected using large volume, *in-situ* filtration (Umhau et al., 2019) and analyzed for its amino acid nitrogen isotopic composition (Hannides et al., 2013; Hannides et al., 2020). As no significant seasonal differences were observed in particle δ^15^N_AA_ values (see Supplemental Materials at https://doi.org/10.5281/zenodo.15548791), data from all cruises were pooled. Although this dataset is geographically limited to a specific location, the depth-related trends in δ^15^N_AA_ values and the relationships between particle types are broadly representative of North Pacific open ocean particles, consistent with patterns observed in the equatorial Pacific (Romero-Romero et al., 2020), the northeast Pacific (Wojtal et al., 2023), and the California Current upwelling system (Miller et al., 2025).

#### Statistical tests

Collinearity of δ^15^N_AA_ values in the Station ALOHA particle data was assessed using Pearson’s correlation coefficients (P), calculated with the stats package in R (R Core Team, 2018). Statistical differences in δ^15^N_AA_ values among organic matter source groups were evaluated using Tukey’s pairwise comparison tests, also *via* the R stats package (R Core Team, 2018). Multivariate differences in δ^15^N_AA_ values among organic matter source groups were assessed using PERMANOVA *via* the vegan package (Dixon, 2003). The effective dimensionality of data was assessed *via* the Shannon entropy, quantified using the functions described in Del Giudice (2021). To visualize and quantify multidimensional separation of organic matter sources, we performed principal component analysis (PCA) using unscaled δ^15^N_AA_ values *via* vegan and linear discriminant functional analysis (LDA) *via* the MASS package (Venables & Ripley, 2002). LDAs were trained on δ^15^N_AA_ values from known organic matter sources, with group identity supplied as the classification variable. Differences in δ^15^N_AA_ values between groups were assessed using two-tailed, heteroskedastic *t*-tests. Predictive relationships were modelled using linear and generalized linear models *via* the stats package, applying a generalized linear model with a quasibinomial distribution and logistic linking function when the response variable was compositional (*i.e.,* mixing coefficients). Visualizations were created using ggplot2 (Wickham, 2016), ggtern (Hamilton & Ferry, 2018), and the effects package (Fox & Weisberg, 2018). All statistical analyses and visualizations were conducted in R Studio using R version 4.4.1 (R Core Team, 2018).

### Determining possible sources of organic matter

The first step in adapting the model is to identify all possible isotopically distinct sources of organic matter in the system. Previous studies at Station ALOHA (Gloeckler et al., 2018; Hannides et al., 2020), in the equatorial Pacific (Romero-Romero et al., 2020), and at Ocean Station Papa (Shea et al., 2023; Wojtal et al., 2023) have collectively identified three likely, isotopically distinct sources of organic matter that could support deep-sea food webs:

1. **Surface POM** – Particles collected by in-situ filtration above 50 to 100 m can be considered representative of fresh surface POM. This material may enter the mesopelagic food web directly by diel vertically migrating organisms that feed near the surface during the night, or indirectly through consumption of these migrators or their fecal pellets at depth.
2. **Large mesopelagic particles** – Particles >6 µm (Station Papa) or >51 µm (5 N, 8 N, Station ALOHA), as well as those collected in sediment traps below about 200 m depth, represent passively sinking particles in the mesopelagic zone. These particles are largely composed of fecal material or aggregated detritus.
3. **Small mesopelagic particles** – Particles 1–6 µm (Station Papa) or ∼1–51 µm (5 N, 8 N, Station ALOHA) collected below about 200 m represent the suspended or slow-sinking fraction in the mesopelagic zone. They are likely to have undergone some degree of microbial degradation and/or resynthesis.

Detailed descriptions of sample collection procedures and particle size classifications can be found in the primary literature (5N and 8N: Umhau et al. (2019); ALOHA: Hannides et al. (2020); Station Papa: Wojtal et al. (2023)).

**Figure 2:**
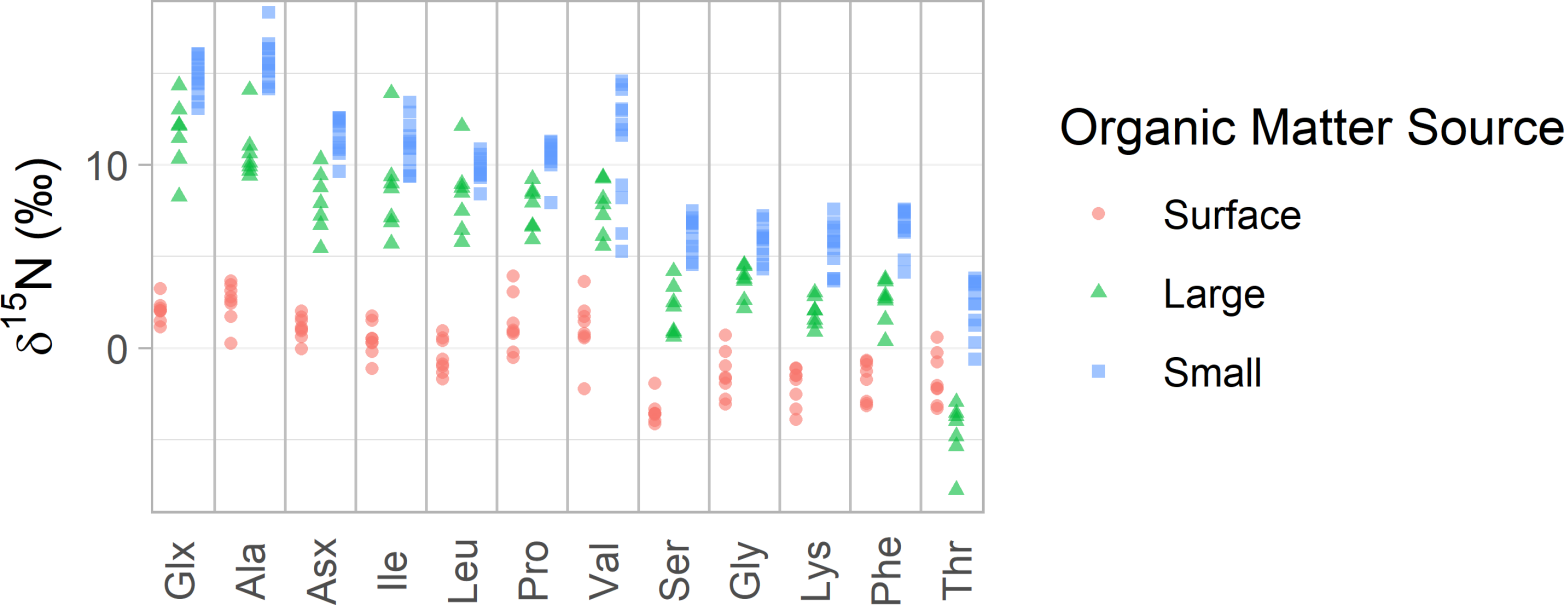
δ^15^N values of amino acids in particles from Station ALOHA. δ^15^N values of 12 amino acids are plotted with colors distinguishing samples belonging to each of the three organic matter sources identified in this study. Surface particles are those collected at or above 100 m depth. Large and small particles are those larger than 51 µm or between 1 and 51 µm, respectively, collected below 200 m.

**Figure 3:**
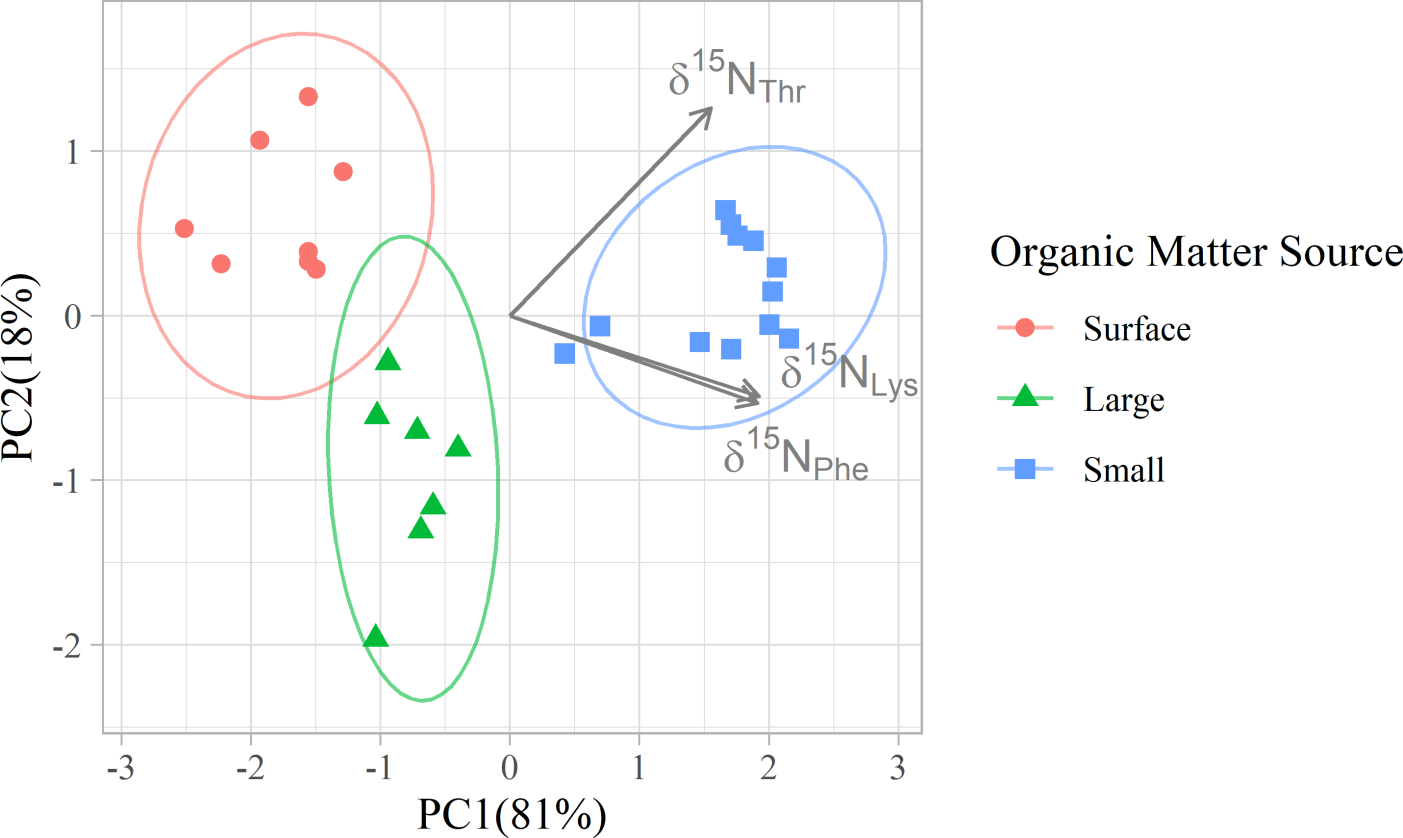
Principal component analyses of mixing tracers for particles at Station ALOHA. PCA was applied to the δ^15^N values of three amino acids (Thr, Phe, and Lys) to visualize multivariate separation of organic matter source groups. Colors differentiate organic matter sources, and arrows indicate the loadings of mixing tracers on the 1st and 2nd principal components.

AA-CSIA data from Station ALOHA clearly illustrates the general isotopic distinctions between these particle groups (Figs. 2 and 3). Small particles tend to exhibit the highest δ^15^N_AA_ values, while surface particles show the lowest, except in the case of δ^15^N_Thr_ values. Broader trends, criteria for sample selection and exclusion, and statistical comparisons among organic matter source groups are discussed in detail in the Supplemental Materials.

### Determination of Δ^15^N_AA_ values

Trophic discrimination factors (Δ^15^N_AA_) are used in the model to calculate trophic parameters (PTS, MTS, FWL) and account for trophic discrimination in mixing tracers. To determine an appropriate set of Δ^15^N_AA_ values for planktonic marine biota, Δ^15^N_AA_ values estimated in controlled feeding studies were aggregated (Gutiérrez-Rodríguez et al., 2014; McMahon & McCarthy, 2016; Décima et al., 2017) and filtered to only include aquatic organisms that excrete ammonia and have a TP of 2 or 3. In addition, one experiment on the fish *Acanthopagrus butcheri* fed vegetable meal was excluded for having anomalously high Δ^15^N_AA_ values. Experimental replicates were pooled such that the final compilation includes 21 experiments on four species of teleost, three species of marine gastropod, three species of pelagic crustacea (representing copepods and shrimp), the planktonic rotifer *Brachionus plicatilis*, and two species of planktonic protozoa (representing dinoflagellates and ciliates). Taxa were categorized as protozoa or metazoa, so that Δ^15^N_AA_ values could be compared between functional groups. The full data compilation is available in Table S2.

Several amino acids do not exhibit significant differences in Δ^15^N values between protozoan and metazoan taxa (Ala: *p* = 0.97, Pro: *p* = 0.21, Gly: *p* = 0.42, Ser: *p* = 0.21, Phe: *p* = 0.98, Lys: *p* = 0.29; Table 2). For these amino acids, Δ^15^N values are calculated as the mean across all experiments in the data compilation, including those of protozoans and metazoans. In the model, their δ^15^N_AA_ values are treated as tracers with uniform fractionation dynamics, following Eq. (7) for trophic discrimination.

In contrast, other amino acids show significant differences in Δ^15^N values between protozoan and metazoan taxa (Glx: *p* = 0.002, Asp: *p* = 0.02, Leu: *p* = 0.0001, Thr: *p* = 0.02; Table 2). For these amino acids, Δ^15^N values are calculated by averaging experiments conducted separately on protozoa and metazoa. In the model, their δ^15^N_AA_ values are treated as tracers with variable fractionation dynamics, following Eq. (8) for trophic discrimination. Regression analysis of δ^15^N_AA-Phe_ against δ^15^N_Glx-Phe_ values also yielded comparable Δ^15^N_AA_ values (Fig. S3), supporting their use in the model.

Two amino acids, isoleucine (Ile: *p* = 0.08) and valine (Val: *p* = 0.05), show marginal differences in their Δ^15^N values between protozoans and metazoans. Because of this uncertainty, we recommend excluding them from the model. Although they likely exhibit variable fractionation dynamics, their Δ^15^N_proto_ values are close to 0, resembling that of δ^15^N_Glx_.

Δ^15^N_Thr_ exhibits the greatest variability across metazoan feeding experiments, ranging from −10.7‰to −1.4‰. In contrast, experiments with protozoans yielded more consistent values, ranging from −2.9‰to −1.5‰. This variability introduces considerable uncertainty when selecting an appropriate value for Δ^15^N_meta_.

Given the large variability in Δ^15^N_AA_ values observed across controlled feeding experiments for many amino acids, we also used a linear regression approach similar to that of Bradley et al. (2015) to estimate Δ^15^N_AA_ values from wild zooplankton AA-CSIA data. This approach regresses δ^15^N_AA_ values against the known TP of the sample, such that Δ^15^N_AA_ values can be derived from the slope of that regression. While Bradley et al. (2015) based their TP estimates on stomach content analysis of teleosts, we instead used δ^15^N_Glx−Phe_ and δ^15^N_Ala−Phe_ as proxies for trophic position, assuming that our Δ^15^N values for these well-studied amino acids are reasonably accurate. Notably, this means we could not reliably estimate Δ^15^N_Ala_ or Δ^15^N_Glx_ using this method.

The regression analysis was based on a large compilation of AA-CSIA data measured in zooplankton and particles at equatorial Pacific sites at 5°N and 8°N (Romero-Romero et al., 2020), Station ALOHA (Hannides et al., 2020; Miller et al., 2025), and Station Papa (Shea et al., 2023; Wojtal et al., 2023). Particles collected above 100 m and zooplankton collected above 200 m were used to represent the surface-ocean food web, primarily composed of phytoplankton and their direct consumers. Phe-normalized δ^15^N_AA_ values (δ^15^N_AA-Phe_) were used to minimize the effects of bacterially mediated isotope fractionation and account for isotopic baseline variability. Linear regressions were fit with δ^15^N_AA-Phe_ as the response variable and either δ^15^N_Glx-Phe_ or δ^15^N_Ala-Phe_ as the predictor. δ^15^N_Glx-Phe_ was used for amino acids that showed significant differences between their Δ^15^N values in metazoa and protozoa consumers in laboratory studies (Table 2), while δ^15^N_Ala-Phe_ was used for amino acids without such differences. Location was also included as a second, non-interacting predictor. Δ^15^N_AA_ was then calculated from the slope of the regression, m, using the equation:

\begin{eqnarray*}{\Delta }^{15}{\mathrm{N}}_{\mathrm{ AA}}=\mathrm{m}\cdot \mathrm{TD}{\mathrm{F}}_{\mathrm{Glx}/\mathrm{Ala}}+{\Delta }^{15}{\mathrm{N}}_{\mathrm{ Phe}} \end{eqnarray*}

where TDF_Glx/Ala_ is the Δ^15^N value of Glx or Ala relative to that of Phe. Additional details of these calculations and methods used for propagation of uncertainty are provided in the Supplemental Materials. The zooplankton and particle AA-CSIA data used, including all analytical uncertainties, and relevant code are available on GitHub (https://github.com/CH-Shea/Organic-Matter-Supply-Model/tree/main/Data) and published *via* Zenodo (DOI: 10.5281/zenodo.15548791).

Regression analysis comparing δ^15^N_AA-Phe_ against δ^15^N_Ala/Glx-Phe_ values in wild zooplankton produced Δ^15^N_AA_ values similar to those observed in controlled feeding studies (Fig. S3), providing confidence in the applicability of those values to wild zooplankton. Regression of δ^15^N_Thr−Phe_ against δ^15^N_Glx−Phe_ values produces a significant slope (ANOVA, *F*_2,89_ = 103, *p* < 0.0001) with a reasonably strong fit (*R*^2^ = 0.86). Because the resulting regression-based Δ^15^N_Thr_ value is in good agreement with the mean value observed in metazoan controlled feeding experiments but has less uncertainty (Table 2, Fig. S3), the model adopts a regression-based estimate of Δ^15^N_Thr_ = −5.9 ±1.5‰ for metazoans.

Choices for Δ^15^N_AA_ values used in the OMSM are summarized in Table 2.

### Choice of tracers and organic matter source separation

Although the OMSM allows for the use of multiple amino acid tracers with non-zero TDFs, careful selection of the tracer set is essential and should be guided by the specific research questions. Notably, the considerations for selecting trophic tracers differ from those for mixing tracers.

For trophic tracers, two amino acids are needed: one with a constant trophic discrimination factor to solve Eq. (2) for food web length, and one with a variable trophic discrimination factor to solve Eq. (3) for metazoan trophic steps. Examination of Eqs. (4) and (5) suggests that choosing trophic tracers with the lowest relative uncertainty (std dev/mean) in their trophic discrimination factor minimizes the uncertainty of model outputs. Among amino acids with constant trophic discrimination factors (Ala, Pro, Gly, Ser, Phe, Lys), relative uncertainty is smallest for Pro (0.30) and Ala (0.40), making either one a strong candidate. We chose to use Pro because of its slightly lower relative uncertainty. For trophic amino acids with variable trophic discrimination factors (Glx, Asx, Leu, Thr), Glx has the smallest relative uncertainty (0.21), and was therefore selected as the most reliable variable TDF tracer. Accordingly, Pro and Glx were used as the trophic tracers in the model.

Selecting mixing tracers is more complex and requires evaluating multiple factors. First and foremost, a tracer’s ability to distinguish among different organic matter sources should be assessed, typically using Tukey’s pairwise comparison tests (or an alternative test if the data are not normally distributed). Effective mixing tracers should differentiate at least two, and ideally more, organic matter sources. Second, the degree of multicollinearity among potential tracers should be evaluated using correlation analysis (*e.g.*, Pearson’s correlation coefficients, *r*) or by evaluating the effective dimensionality of the data set (Del Giudice, 2021). For n sources of organic matter, the best resolution is achieved using at least n-1 *independent* tracers. Therefore, identifying multiple, *uncorrelated* tracers is advantageous. However, if analytical error is a specific concern for a given tracer, including a second, redundant tracer may reduce the model sensitivity to analytical error, even if it does not provide additional information. Finally, inspection of Eqs. (10) and (11) reveals that uncertainty in the model output caused by fractionating tracers is influenced by the number of trophic steps (*i.e.,* FWL, PTS, MTS) and by uncertainty in trophic discrimination factors. This implies that model uncertainty will be lower for shorter food webs, and that amino acid mixing tracers with lower absolute uncertainty in their Δ^15^N values should be prioritized.

For the Station ALOHA particle data, we find that the three organic matter sources (surface, large, and small particles) have distinct δ^15^N_AA_ values, which is visually apparent (Fig. 2) and statistically supported by permutational multivariate analysis of variance (PERMANOVA) (*p* < 0.001). Tukey’s pairwise comparison tests show that all 12 δ^15^N_AA_ values examined here differentiate at least two pairs of organic matter sources with >95% confidence, while 10 of them distinguish all three sources (see pairwise mean difference plots in Fig. S2). Despite this, visual inspection of the data shows that many amino acids display similar patterns in δ^15^N values across the dataset (Fig. 2). This is supported by correlation analysis (Fig. S3), which shows that many amino acid δ^15^N values are highly correlated, with a mean absolute Pearson’s correlation coefficient of |*r*| = 0.86. Trophic amino acid δ^15^N values are consistently highly correlated with one another (*r* = 0.87 - 0.99) and also correlated with source amino acid δ^15^N values (*r* = 0.85 –0.98). δ^15^N_Thr_ stands out as the least correlated to other amino acid δ^15^N values, with Pearson’s correlation coefficients ranging from 0.29 to 0.59, indicating it may provide uniquely independent information relative to other amino acids.

**Figure 4:**
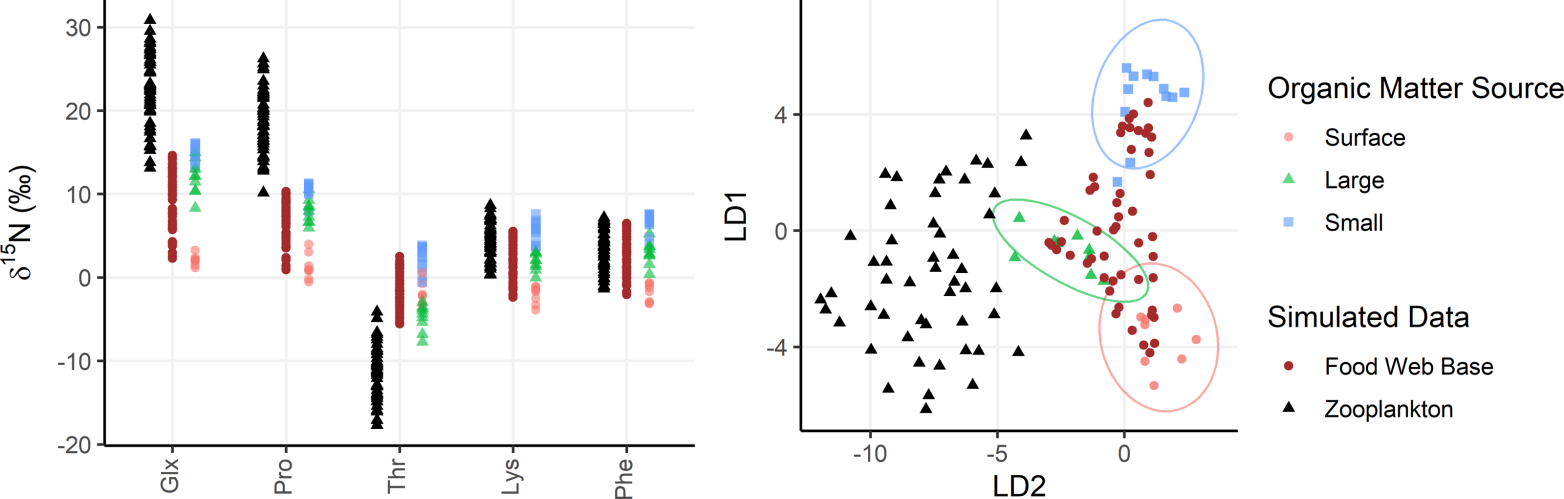
Simulated zooplankton data shown relative to organic matter sources. **Left:** δ^15^N values of trophic tracers (Glx, Pro) and mixing tracers (Lys, Phe, Thr) are plotted for the base of the food web (brown dots) and in zooplankton themselves (black triangles) relative to organic matter sources (red, green, and blue shapes). **Right:** The simulated data is plotted in an LDA trained using the organic matter source groups and plotted using the same color/shape scheme. The LDA is only based on mixing tracers (Lys, Phe, Thr).

In addition to their ability to statistically distinguish all organic matter sources, δ^15^N_Phe_ and δ^15^N_Lys_ have the smallest associated uncertainties in their trophic discrimination factors (*σ*(Δ^15^N_Phe_) = 0.5 and *σ*(Δ^15^N_Lys_) = 1.2‰). Moreover, while highly correlated (*r* = 0.96), including both phenylalanine and lysine as mixing tracers could help buffer the model against analytical error arising from interference peaks that can occur around both amino acids during AA-CSIA (Fig. S4). These characteristics make them a logical pair of tracers to include in the model. While most other δ^15^N_AA_ values are also correlated with δ^15^N_Phe_ and δ^15^N_Lys_ values (Fig. S3), δ^15^N_Thr_ is notable for its weak correlation with these and other tracers, offering more orthogonal information. Principal component analysis of δ^15^N_Phe_, δ^15^N_Lys_ and δ^15^N_Thr_ values (Fig. 4), reveals that 81% of the variance in the data is explained by the first principal component (PC1), which primarily reflects variation in δ^15^N_Phe_ and δ^15^N_Lys_ values and distinguishes small particles from surface and large particles. An additional 18% of the variance is explained by the second component (PC2), which primarily captures variation in δ^15^N_Thr_ values and separates surface and large particles. These three tracers have 1.7 effective dimensions, suggesting they can almost fully determine a 3-component mixture. Together, these findings support the combined use of δ^15^N_Phe_, δ^15^N_Lys_, and δ^15^N_Thr_ values to minimize model uncertainty and enhance the ability to resolve organic matter sources.

Finally, because phenylalanine has such a low trophic discrimination factor (Δ^15^N_Phe_ = 0.3 ±0.5‰), we propose that δ^15^N_Phe_ values can be treated as a conservative tracer in relatively short food webs, allowing us to avoid propagation of uncertainty in Δ^15^N_Phe_, thus increasing the precision of model outputs. This assumption is supported by a complementary analysis presented in the Supplemental Materials, in which δ^15^N_Phe_ values were modeled non-conservatively. These analyses yielded similar, though slightly more variable, results (see Supplemental Materials at https://doi.org/10.5281/zenodo.15548791).

### Procedures for model assessment using simulated zooplankton data

To assess model performance, the OMSM is tested using simulated zooplankton data, allowing model outputs to be compared with the known or “true” parameters used to generate the data. Particle data collected at Station ALOHA are used to represent realistic AA-CSIA data on organic matter sources to the mesopelagic zone. AA-CSIA data for 50 zooplankton samples (see Data S1) are then simulated by selecting realistic values for key ecological parameters (*e.g.*, mixing coefficients, PTS, MTS, Δ^15^N_AA_, *etc.*). Each of the 50 samples is assigned a unique set of mixing coefficients, representing a range of potential organic matter supply scenarios, drawn from a uniform Dirichlet distribution. These coefficients are applied to Eq. (1) to calculate the isotopic composition of each tracer at the base of the food web for each sample. Values for PTS and MTS for each sample are drawn from uniform distributions ranging from 0 to 1 and 1 to 2, respectively, yielding food web lengths ranging from 1 to 3. Using Eq. (12) and the mean Δ^15^N_AA_ values provided in Table 2, the δ^15^N_AA_ values for 50 simulated zooplankton samples are calculated. This data is then used as an input to the OMSM, enabling evaluation of model performance by comparing its output to the ecological parameters used to generate the simulated data. In diagnostic plots, the model output (or posterior distribution) for each model parameter (*f*_*j*,*k*_, FWL, MTS, PTS, δ^15^N_AA,source_, δ^15^N_AA,base_, δ^15^N_AA,zoop_) is compared to the “true” value selected or calculated during data simulation.

In model validation exercises we assume that we possess minimal prior knowledge. To do this, we use priors that will exert little influence over model outputs. For the mean organic matter source tracer value (μ_*j*,*i*_) we use uniform prior distributions ranging from -100 to 100, for the standard deviation of organic matter source tracer values (*σ*_*j*,*i*_) we use gamma prior distributions with shape and rate parameters set to 0.001 (Lemoine, 2019), and for mixing coefficients (*f*_*i*_) we use a uniform Dirichlet prior distribution. When fitting the simulated zooplankton data, the OMSM is run using three parallel chains. Each chain includes a 5,000 step adaptation phase, a 10,000 step burn-in, and a 10,000 step sampling phase with a thinning factor of 10.

To assess the accuracy of the model’s estimated values for trophic and mixing parameters (posterior probability density functions), the known, simulated values for each parameter are compared to the mode of each posterior, identifying if the true value falls within the 50, 75, 90, or 95% HDI of the posterior. We also fit regressions of both the modelled value and the discrepancies against the true value to assess systematic error in model outputs.

## Model Results and Assessment Using Simulated Zooplankton Data

**Figure 5:**
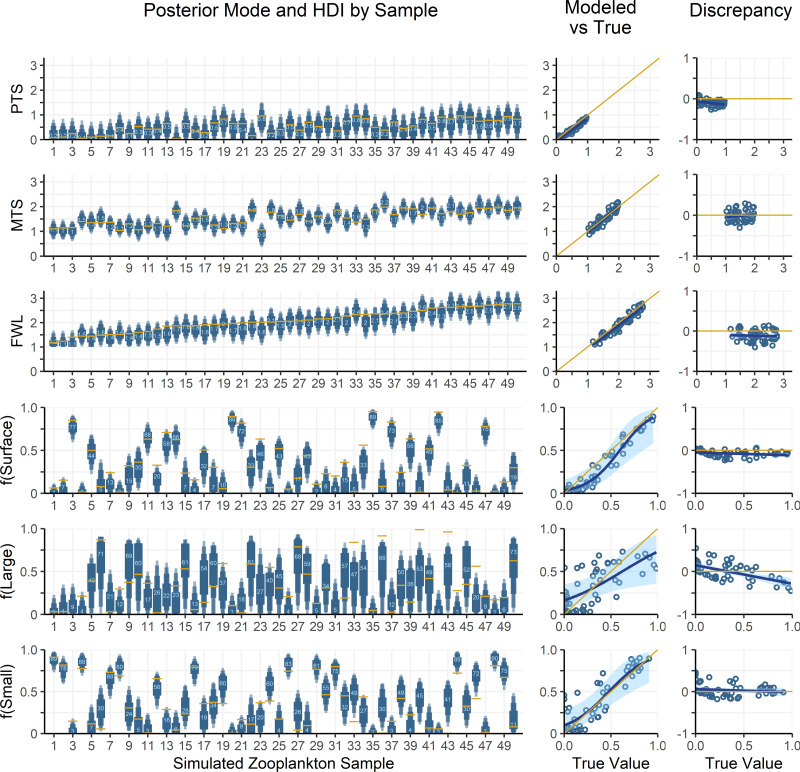
Comparing modelled and true parameter values for 50 simulated zooplankton samples. **Left:** True values for mixing coefficients and trophic parameters used to synthesize zooplankton δ^15^N data are plotted as yellow lines. Model posterior highest density intervals (HDIs) are plotted as blue boxes with thin *vs* thick boxes indicating 50, 75, 90 and 95% HDIs. Light blue numbers indicate the posterior mode for each sample. The *x*-axis indicates the sample number for each of the simulated zooplankton samples. **Middle:** Modelled parameter values are regressed against true values. The posterior modes are plotted in open circles for each sample, with the regression through the modes shown as a dark blue line. The light blue region is the 95% confidence interval about the regression, while the yellow 1:1 line indicates a perfect fit for reference. **Right:** The discrepancy between modelled and true values is regressed against the true values. The plotting scheme is the same as for the middle plot.

Zooplankton samples were simulated using particle data from Station ALOHA (Fig. 4), providing realistic data that reflect known mixing and trophic parameters. Comparing model outputs to the known, true parameter values reveals that the model generally performs well (Fig. 5). For most parameters, the true values are closely approximated by the posterior modes and fall within the 95% highest density intervals of the posterior distributions, except for f(large) which shows a larger discrepancy. Full details of the model outputs are available in the Supplemental Materials.

The OMSM performed especially well in estimating the contribution of surface particles to zooplankton diets (Fig. 5). The true value of f(surface) aligns closely with the posterior mode (average absolute discrepancy: 8%, maximum: 23%), was captured within the 95% HDI of the posterior probability density function (PDF) for 49 out of 50 samples, and was within the 50% HDI for 25 out of 50 samples. This strong performance is driven by the distinctly low δ^15^N_Phe_ and δ^15^N_Lys_ values in surface particles relative to large and small particles at depth, combined with the fact that these tracers experience minimal trophic discrimination (Figs. 3; 4). When the model misidentified f(surface), the error was consistently in the direction of underestimation, suggesting that the OMSM has a particularly strong and conservative ability to detect the contribution of surface-derived organic matter to the Station ALOHA food web.

The model also effectively quantified contributions of small particles (Fig. 5, f(small)), although error rates were slightly higher than for f(surface), with an average absolute discrepancy of 12%, and a maximum of 45%. The accuracy of the posterior mode increased with higher values of f(small). Although the posterior mode became a less reliable estimator of the true value at lower values of f(small), the 95% HDI remained a dependable measure, containing true value in 46 out of 50 simulated samples. These results indicate that the OMSM has a robust ability to positively identify the importance of small particles at Station ALOHA.

In contrast, the model had greater difficulty estimating the importance of large particles (Fig. 5, f(large)). The HDIs for f(large) were generally broader than those for f(small) and f(surface), reflecting greater uncertainty. This is largely because large particles have δ^15^N_Phe_ and δ^15^N_Lys_ values that are intermediate between surface and small particles. They are primarily differentiated by δ^15^N_Thr_ values, which are more sensitive to trophic discrimination. Still, the model performed reasonably well. The true values for 44 out of 50 samples were contained within the 95% HDI and 21 out of 50 are contained within the 50% HDI. The posterior mode loosely tacked the true value of f(large) with an average absolute discrepancy of 18%, and a maximum of 54%.

Finally, the OMSM reproduced trophic parameters with a high degree of accuracy (Fig. 5, PTS, MTS, FWL). In every case, the true parameter value fell within the 95% HDI, and within the 50% HDI for most samples (37/50 for FWL, 50/50 for PTS, 45/50 for MTS). The posterior modes closely approximated the true values, with the mean absolute discrepancy of 0.12 for PTS, 0.11 for MTS and 0.15 for FWL.

## Discussion

The OMSM enables the modeling of isotopic tracers across lower trophic levels of planktonic food webs in a manner consistent with our current understanding of amino acid isotope fraction dynamics (McMahon & McCarthy, 2016; Décima et al., 2017; Ohkouchi et al., 2017). This approach permits the integration of trophic amino acid δ^15^N values as mixing tracers, while explicitly accounting for the distinct isotope fractionation that occurs during protistan *versus* metazoan trophic steps. By simultaneously solving isotope mixing and trophic discrimination equations, the model incorporates trophic effects into mixing tracers without requiring a priori assumptions about the trophic structure of the food web. These innovations significantly broaden the applicability of stable isotope mixing models and enhance the potential of AA-CSIA to provide deeper insight into the structure and function of marine ecosystems.

Proline and glutamic acid were found to be ideal trophic tracers for food web length and metazoan trophic steps, respectively, though alanine would be a suitable replacement for proline if necessary. Because these findings were based on low relative uncertainty in trophic discrimination factors for these amino acids, they should be generalizable to other studies focused on similar planktonic taxa. Choices for mixing tracers, however, will likely be unique to the research question, and so these should be assessed in future studies based on the criteria given in Section ‘Choice of Tracers and Organic Matter Source Separation’, avoiding amino acids with inconsistent or poorly constrained isotope fractionation behavior (*e.g.*, Ile, Val) as much as possible.

When adapted to quantify organic matter supply pathways to deep-sea zooplankton, consumer data simulation exercises highlight both the strengths and limitations of the OMSM in a more specific context. One of the model’s clearest strengths is its ability to accurately estimate contributions from deep small and surface-derived particles to the base of the planktonic food web. This capability stems from the distinct isotopic composition of these sources and specifically, to the uniquely high δ^15^N_Phe_ and δ^15^N_Lys_ values in small particles and low values of these same tracers in surface particles. Similar patterns have been noted in prior studies using the average δ^15^N value of Ser, Gly, Phe, and Lys (δ^15^N_SAA_) to differentiate particle sources (Gloeckler et al., 2018; Romero-Romero et al., 2020; Shea et al., 2023). However, these earlier approaches relied on the assumption that all source amino acids undergo negligible trophic discrimination, allowing direct comparison of δ^15^N_SAA_ values in particles with those of zooplankton or micronekton. In contrast, the δ^15^N_AA_ values compiled here and derived from earlier controlled feeding studies (Gutiérrez-Rodríguez et al., 2014; McMahon & McCarthy, 2016; Décima & Landry, 2020) and supported by our regression analysis, indicate that this assumption does not hold universally (Nielsen, Popp & Winder, 2015). While it may be reasonable for Phe and possibly for Lys, both Ser and Gly can exhibit positive Δ^15^N_AA_ values (Table 2), potentially biasing prior mixing model results. By accounting for this trophic discrimination, the OMSM provides a more accurate and flexible framework. These results suggest that future studies using OMSM can refine understanding of the role of small particles and diel vertical migration in deep-sea organic matter supply pathways.

**Figure 6:**
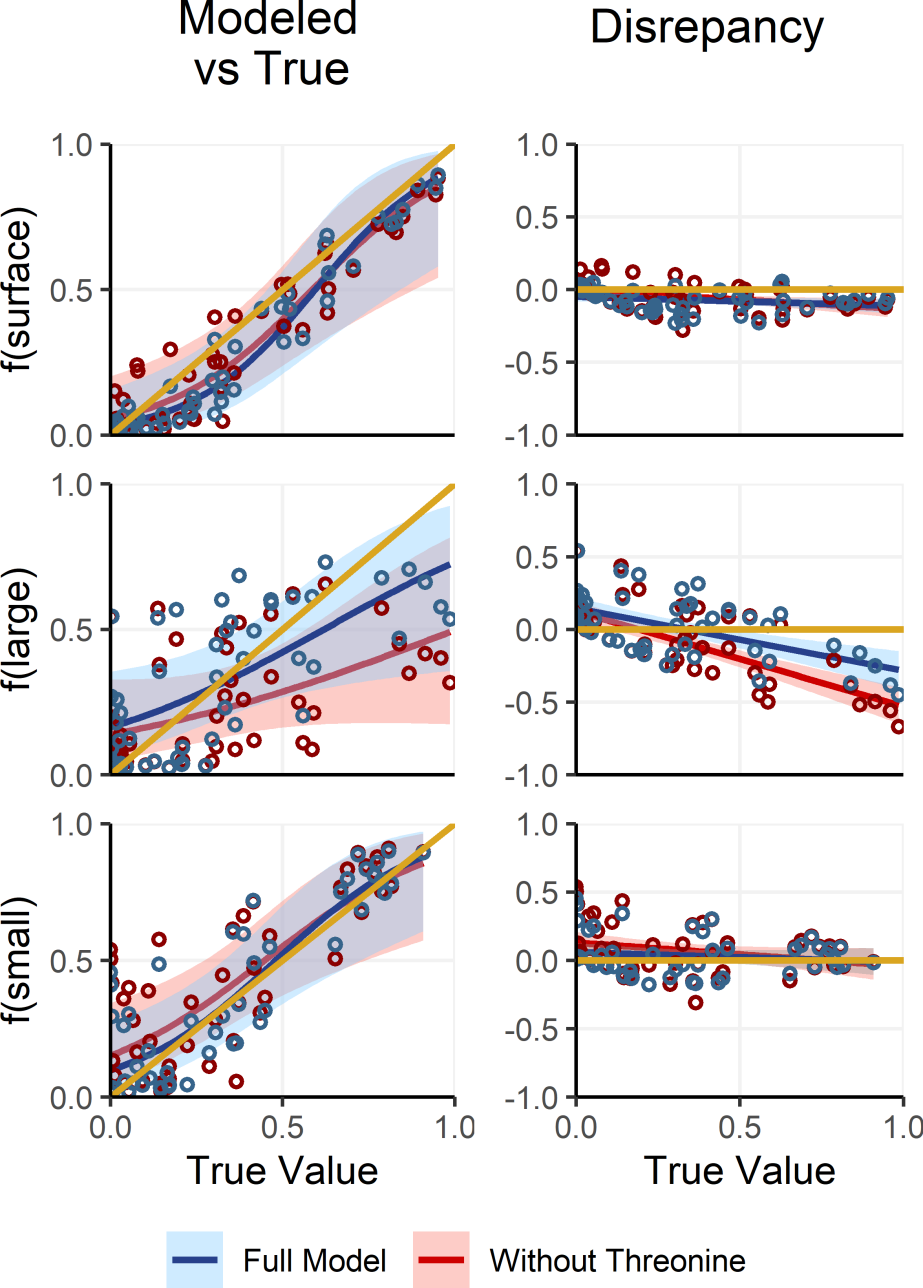
Comparing model posteriors for the full OMSM and a non-fractionating OMSM. A separate version of the OMSM was fit where δ^15^N_Phe_ and δ^15^N_Lys_ were used as mixing tracers and δ^15^*N*_Thr_ was excluded. The mode of the model posterior for mixing coefficients are plotted against the true coefficient values (**left**), while the difference between modeled and true values is plotted against the true values (**right**). The posterior modes and regressions for the full model are plotted in blue, while the non-fractionating model is plotted in red. Shaded regions indicate the 95% confidence interval about the regression.

Deep large particles, in contrast to small and surface particles, had intermediate δ^15^N_Phe_ and δ^15^N_Lys_ values and were primarily distinguished by their uniquely low δ^15^N_Thr_ values. However, because δ^15^N_Thr_ values are affected by trophic discrimination (Bradley et al., 2015; Fuller & Petzke, 2017), uncertainty in Δ^15^N_Thr_ propagates through the process model, resulting in increased uncertainty in estimates of f(large). To assess whether including δ^15^N_Thr_ values improves model performance or whether it would be preferable to adopt a simplified, non-fractionating model akin to those used in previous studies (Gloeckler et al., 2018; Romero-Romero et al., 2020; Shea et al., 2023), we refit the OMSM described in Section ‘Adapting the OMSM to Assess Organic Matter Supply Pathways in the Deep Sea’. In this alternative model, δ^15^N_Thr_ values were excluded and both δ^15^N_Phe_ and δ^15^N_Lys_ values were treated as conservative tracers, based on their low Δ^15^N_AA_ values (Table 2). We then compared posterior estimates of mixing coefficients against the true parameter values (Fig. 6). Results reveal that the alternative, non-fractionating model performs significantly worse in estimating f(large), with discrepancies between posterior modes and the true values reaching up to 71%, compared to a maximum of 43% for the full model. While the full OMSM also shows improved accuracy for f(small) relative to the non-fractionating model, this is largely due to the incorporation of the 1.2‰Δ^15^N value for δ^15^N_Lys_. Estimates of f(surface) were comparable between the two approaches. Overall, these findings support the inclusion of δ^15^N_Thr_ values in the model, affirming that accounting for trophic discrimination enhances the accuracy of mixing estimates and underscoring the value of the OMSM as a meaningful advancement over non-fractionating models used in earlier studies.

The OMSM also aligns with current understanding of isotope discrimination in natural systems by independently parameterizing Δ^15^N_AA_ values in protozoan and metazoan trophic steps (see McMahon & McCarthy, 2016; Ohkouchi et al., 2017). This distinction reflects growing evidence that Δ^15^N values differ markedly between protozoan and metazoan metabolisms for certain amino acids (Gutiérrez-Rodríguez et al., 2014; Décima et al., 2017; Décima & Landry, 2020). By explicitly modeling these differences, the OMSM more accurately captures the contribution of marine protists to amino acid isotopic compositions and assures robust accounting for trophic discrimination across food webs. This capability enhances model accuracy and opens new avenues for exploring the foundational role of heterotrophic protists in marine ecosystems. However, this strength is tempered by a significant limitation. The current estimates of trophic discrimination factors for protists (Δ^15^N_AA,proto_) rely on a small number of controlled feeding studies involving protists. Further experimental research is needed to refine and build confidence in Δ^15^N_AA,proto_ values and to assess variability across taxonomic groups, which would bolster confidence in this component of the model.

Finally, although the OMSM is optimized for use with δ^15^N_AA_ values, it is flexible and could be used with other types of tracers. Most notably, δ^13^C values of essential amino acids (δ^13^C_EAA_) are powerful tracers for differentiating inputs from various primary producer clades in marine ecosystems (Larsen et al., 2013; Arthur et al., 2014; Stahl, Rynearson & McMahon, 2023), and could enhance the ability of δ^15^N_AA_ values to resolve organic matter sources. δ^13^C_EAA_ values could be incorporated directly into the OMSM as conservative tracers, however, further evaluation of carbon isotope discrimination in essential amino acids is warranted. Although the assumption of conservative behavior in essential amino acids (EAAs) is common in the literature, and current evidence suggest metazoan metabolism does not alter their δ^13^C values (Fantle et al., 1999; McMahon et al., 2015), very little is known about carbon isotope fractionation of essential amino acids in protozoan trophic steps. Similarly, there is a lack of data on trophic carbon isotope discrimination in non-essential amino acids, which could also serve as tracers of organic matter sources, provided that their isotopic discrimination in food webs is better understood.

## Conclusion

In summary, the OMSM described here represents a significant advancement in our ability to accurately model organic matter incorporation and trophic discrimination in planktonic ecosystems, particularly where marine protists play an active role and the food web’s trophic structure is uncertain. The model enables more accurate assessments of the relative contributions of different organic matter supply pathways to mesopelagic zone food webs using δ^15^N_AA_ data, although its ability to precisely quantify the importance of large particles in the settings assessed here is limited. Importantly, the model’s flexible structure allows it to leverage a greater portion of the detailed information typically generated by AA-CSIA than previous approaches, making it suitable for a broad range of diet-tracing and organic matter source attribution problems. Documentation and implementation guidance are available on GitHub at https://github.com/CH-Shea/AA-CSIA-Organic-Matter-Supply-Model (DOI: 10.5281/zenodo.15548791). Further resources including working examples with varying food web complexity, and code templates will continue to be made available following publication, and can be accessed *via* the GitHub repository linked above.

##  Supplemental Information

10.7717/peerj.20220/supp-1 Supplemental Information 1 Comparisons of δ^15^N_SAA_ values estimated from regression analysis to those derived from the controlled feeding studies Red circles and error bars show the mean and standard deviation of δ^15^N_SAA_ values estimated from 18 controlled feeding studies of marine metazoans with TP ≤ 3, as described in section 3.3. Blue squares and green triangles show the δ 15 N values estimated by regressing δ^15^N_AA-Phe_ values against δ^15^N_Ala/Glx-Phe_ and δ^15^N_Ala-Phe_, respectively. Some amino acids are shown to fractionate uniformly throughout food webs (Ala, Pro, Ser, Gly, Lys), so for these we emphasize the results of linear regression against δ^15^N_Ala-Phe_. Others show variable trophic discrimination during metazoan vs protozoan metabolism (Glx, Asx, Leu, Thr), so for these we emphasize the results of linear regression against δ^15^N_Ala/Glx-Phe_.

10.7717/peerj.20220/supp-2 Supplemental Information 2 Pairwise mean difference plots for all possible tracers Each panel identifies the ability of the designated tracer to separate pairs of organic matter sources from one another. The y-axis identifies the contrast being assessed (e.g., Large-Surface indicates the difference between large deep particles and surface particles) which the x-axis indicates the magnitude of that difference. The center line on each bar indicates the average difference between groups in the pairwise comparison, while the ends of the segment indicate the difference necessary to differentiate the groups with >95% confidence. When the entire bar lies above or below 0, this indicates a significant difference between groups with respect to the indicated tracer.

10.7717/peerj.20220/supp-3 Supplemental Information 3 An example mass 28 chromatogram The signal intensity from the mass 28 detector is plotted through time for a zooplankton sample analyzed as trifluoroacetic amino acid esters using a gas chromatograph coupled to an isotope ratio mass spectrometer equipped with a 60 m ×0.32 mm BP×5 column (for details see Hannides et al. , 2009, 2020; (Shea et al., 2023) . The blue and red insets show closeups of the areas around phenylalanine (top) and lysine (bottom). Though many samples exhibit clean baselines around these peaks, it is not uncommon to observe interference by unknown, co-eluting compounds as exhibited by this sample. This occurrence is specific to the chemical preparation methods, derivatization techniques, and chromatographic conditions used to generate these data.

10.7717/peerj.20220/supp-4 Supplemental Information 4 Pearson’s correlation coefficients for δ^15^N_AA_ values in organic matter sources Pairwise correlations between all δ^15^N_AA_ values were assessed by calculating Pearsons correlation coefficients for all possible pairs of tracers. Coefficients are shown for the indicated pair in the top right, histograms of tracer values are shown along the diagonal, and linear regressions are plotted in the bottom left.

10.7717/peerj.20220/supp-5 Supplemental Information 5 Ecological parameters and amino acid δ15N values of simulated zooplankton Simulated sample ID numbers are stored in column A, true values for the fractional contribution of surface, deep large, and deep small particles in columns B-D, protistan trophic steps, metazoan trophic steps, and food web length in columns E-G, and amino acid δ15N values (‰) are columns H-P.

10.7717/peerj.20220/supp-6 Supplemental Information 6 δ^15^*N* values determined in controlled feeding studies The species name and number of experimental replicates are stored in column A, common name in column B, designation as a protozoan or metazoan in column C, diet in column D, amino acid δ^15^*N* values in columns E-Q, presumed trophic position in column S, mode of nitrogen excretion in column T, sample type in column U, natural habitat type in column V, and original reference in column W. This table is an adaptation of that provided in McMahon & McCarthy (2016).

10.7717/peerj.20220/supp-7 Supplemental Information 7 The outputs of statistical analyses and OMSM model runs that expand upon those in the main text These are also available at GitHub (https://github.com/CH-Shea/Organic-Matter-Supply-Model).

## Additional Information and Declarations

### Data Availability

The following information was supplied regarding data availability: Data is available at GitHub and Zenodo: - https://github.com/CH-Shea/Organic-Matter-Supply-Model
- Connor Shea. (2025). CH-Shea/Organic-Matter-Supply-Model: OMSM v0.2 - pre-release of model code (v0.2). Zenodo. https://doi.org/10.5281/zenodo.15548791.
