# Supplementary figures and images for: A Bayesian model for assessing organic matter supply in complex marine food webs using amino acid stable isotope analysis
Source: PeerJ. 2025 Nov 19;13:e20220. doi: 10.7717/peerj.20220 (PMC12640130; doi:10.7717/peerj.20220)

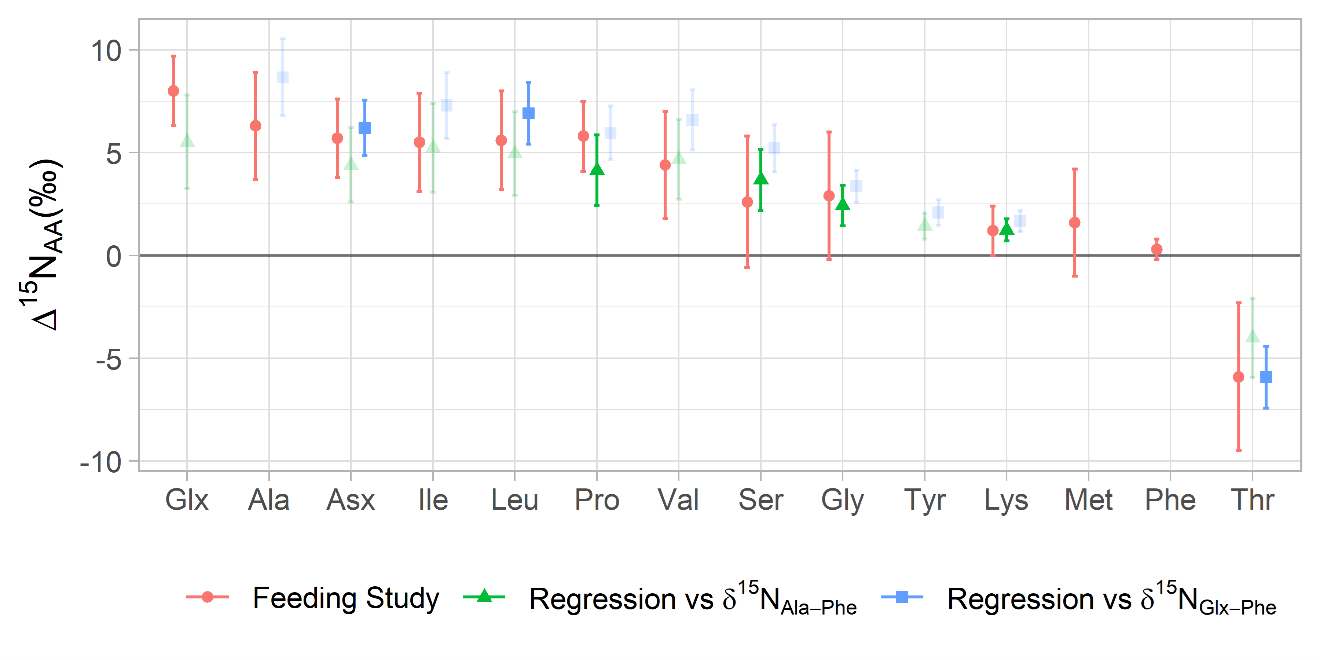

Supplement: Supplemental Information 1 — Red circles and error bars show the mean and standard deviation of δ15NSAA values estimated from 18 controlled feeding studies of marine metazoans with TP ≤ 3, as described in section 3.3. Blue squares and green triangles show the δ 15 N values estimated by regressing δ15NAA-Phe values against δ15NAla/Glx-Phe and δ15NAla-Phe, respectively. Some amino acids are shown to fractionate uniformly throughout food webs (Ala, Pro, Ser, Gly, Lys), so for these we emphasize the results of linear regression against δ15NAla-Phe. Others show variable trophic discrimination during metazoan vs protozoan metabolism (Glx, Asx, Leu, Thr), so for these we emphasize the results of linear regression against δ15NAla/Glx-Phe. [file peerj-13-20220-s001.png]

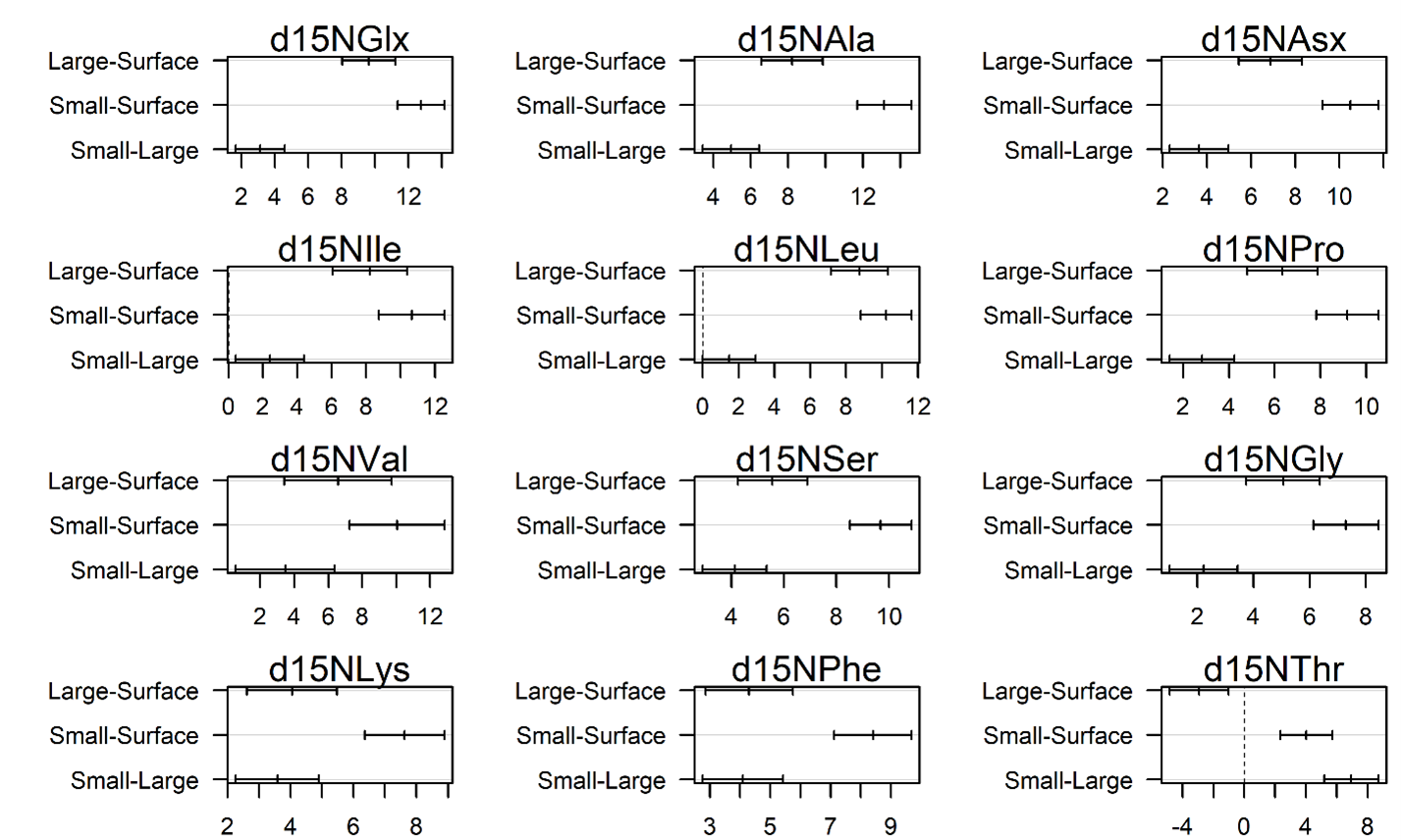

Supplement: Supplemental Information 2 — Each panel identifies the ability of the designated tracer to separate pairs of organic matter sources from one another. The y-axis identifies the contrast being assessed (e.g., Large-Surface indicates the difference between large deep particles and surface particles) which the x-axis indicates the magnitude of that difference. The center line on each bar indicates the average difference between groups in the pairwise comparison, while the ends of the segment indicate the difference necessary to differentiate the groups with >95% confidence. When the entire bar lies above or below 0, this indicates a significant difference between groups with respect to the indicated tracer. [file peerj-13-20220-s002.png]

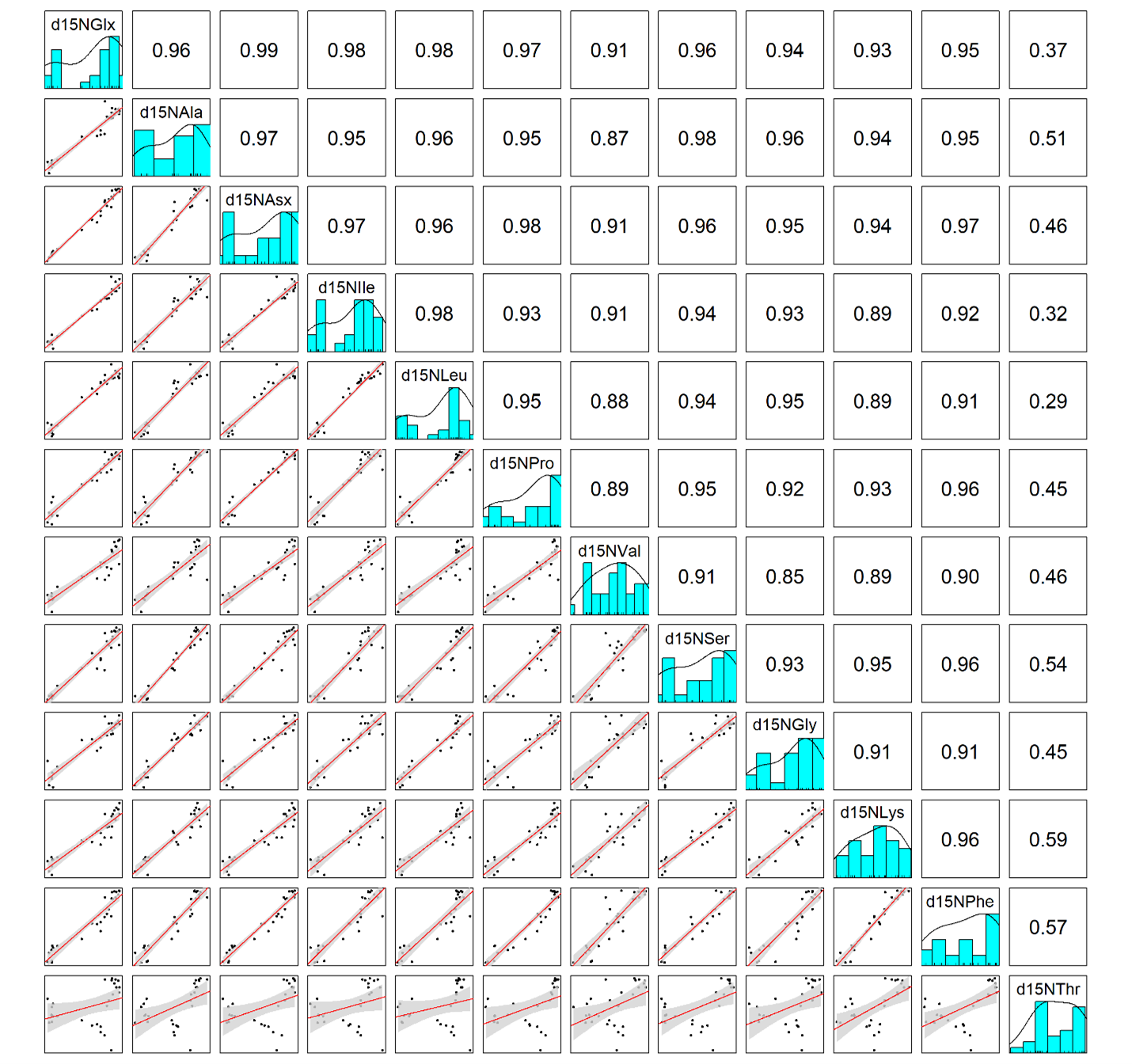

Supplement: Supplemental Information 3 — The signal intensity from the mass 28 detector is plotted through time for a zooplankton sample analyzed as trifluoroacetic amino acid esters using a gas chromatograph coupled to an isotope ratio mass spectrometer equipped with a 60 m ×0.32 mm BP×5 column (for details see Hannides et al. , 2009, 2020; (Shea et al., 2023) . The blue and red insets show closeups of the areas around phenylalanine (top) and lysine (bottom). Though many samples exhibit clean baselines around these peaks, it is not uncommon to observe interference by unknown, co-eluting compounds as exhibited by this sample. This occurrence is specific to the chemical preparation methods, derivatization techniques, and chromatographic conditions used to generate these data. [file peerj-13-20220-s003.png]

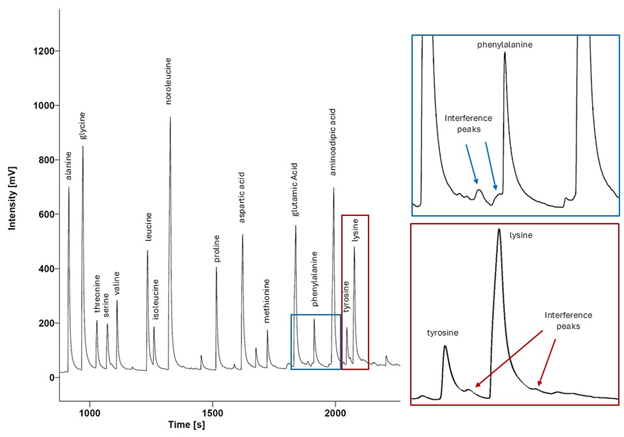

Supplement: Supplemental Information 4 — Pairwise correlations between all δ15NAA values were assessed by calculating Pearsons correlation coefficients for all possible pairs of tracers. Coefficients are shown for the indicated pair in the top right, histograms of tracer values are shown along the diagonal, and linear regressions are plotted in the bottom left. [file peerj-13-20220-s004.png]
