# Supplementary material for: A Bayesian model for assessing organic matter supply in complex marine food webs using amino acid stable isotope analysis
Source: PeerJ. 2025 Nov 19;13:e20220. doi: 10.7717/peerj.20220 (PMC12640130; doi:10.7717/peerj.20220)
Supplement: Supplemental Information 7 — These are also available at GitHub (https://github.com/CH-Shea/Organic-Matter-Supply-Model). [file peerj-13-20220-s007.zip › Supplemental Files/S3_Source_Separation_ALOHA.html]

Organic Matter Source Separation and Sample Selection - Station ALOHA


Code 

- Show All Code
- Hide All Code

# Organic Matter Source Separation and Sample Selection - Station ALOHA

#### Connor Shea

#### May 12, 2025

The goal of this document is to assess the separation of different possible sources of organic matter in isotope space, as well as to filter out the samples that are not representative of likely sources. We will begin by just looking at particle data from Station ALOHA. The organic matter sources we will be considering are:

1. Surface particles
2. Large particles (>51 μm)
3. Small particles (1-51 μm)
4. Submicron particles (0.3-1.0 μm)

```
## SPECIFYING TRACERS TO BE USED IN MODEL ##

## Should amino acid δ15N data be included? (T/F)
include_d15N <- TRUE

## Should amino acid δ13C data be included? (T/F)
include_d13C <- FALSE
## Should essential amino acid δ13C data be mean normalized?
meannorm_d13C <- FALSE

# If using δ15N values in model
if(include_d15N == TRUE) {
  ## Which amino acid δ15N values should be included in the model? (should be 
  ## consistent with column names in data file)
  tracers_d15N <- c("d15NGlx","d15NAla","d15NAsx","d15NIle","d15NLeu","d15NPro","d15NVal","d15NSer","d15NGly","d15NLys","d15NPhe","d15NThr")
  ## What columns in the data file contain the uncertainties in those δ15N values?
  SDtracers_d15N <- c("SDd15NGlx","SDd15NAla","SDd15NAsx","SDd15NIle","SDd15NLeu","SDd15NPro","SDd15NVal","SDd15NSer","SDd15NGly","SDd15NLys","SDd15NPhe","SDd15NThr")
} else {
  tracers_d15N <- c()
  SDtracers_d15N <- c()
}

# If using δ13C values in model
if(include_d13C == TRUE) {
  ## Which essential amino acid δ13C values should be included in the model? 
  ## (should be consistent with column names in data file)
  tracers_d13C <- c()
  ## What columns in the data file contain the uncertainties in those δ13C values?
  SDtracers_d13C <- c()
} else {
  tracers_d13C <- c()
  SDtracers_d13C <- c()
}

## Now we'll make one vector containing all of the tracers to be used in the model
tracers_all <- c(tracers_d15N,tracers_d13C)
SDtracers_all <- c(SDtracers_d15N,SDtracers_d13C)

## We'll also specify other subsets of tracers to be assessed
tracers_SAA <- c("d15NSer","d15NGly","d15NLys","d15NPhe")
SDtracers_SAA <- c("SDd15NSer","SDd15NGly","SDd15NLys","SDd15NPhe")

tracers_select <- c("d15NLys","d15NPhe","d15NThr")
SDtracers_select <- c("Sd15NLys","SDd15NPhe","SDd15NThr")

tracers_complete <- c(tracers_select)
SDtracers_complete <- c(SDtracers_select)

## Code in chunks below will generally refer to the vector "tracers."
## this is done to make code easily portable, but we must update the "tracers"
## vector to reflect the current tracers of interest in a given portion of code
tracers <- tracers_all
SDtracers <- SDtracers_all

# Last we'll define some generic groups of AAs that may be useful later on.
# Update to reflect the column names in your data
# all amino acids
allAA = c("Ala", "Gly", "Thr", "Ser", "Val", "Leu", "Ile", "Pro", "Asx", "Met", "Glx", "Phe", "Tyr", "Lys", "SAA", "EAA")
# columns with SD
SDallAA <- c("SDAla", "SDGly", "SDThr", "SDSer", "SDVal", "SDLeu", "SDIle", "SDPro", "SDAsx", "SDMet", "SDGlx", "SDPhe", "SDTyr", "SDLys", "SDSAA", "SDEAA")
# source amino acids
srcAA = c("Phe","Gly","Ser","Lys")
# columns with SD
SDsrcAA = c("SDPhe","SDGly","SDSer","SDLys")
# trophic amino acids
trAA = c("Glx", "Asx", "Ala", "Ile", "Leu", "Pro", "Val","Thr")
# columns with SD
SDtrAA = c("SDGlx", "SDAsx", "SDAla", "SDIle", "SDLeu", "SDPro", "SDVal","SDThr")
# All amino acids - sorted by SAA TAA and Thr
allAA.ord <- c("Glx", "SDGlx",  "Ala", "SDAla", "Asx", "SDAsx", "Ile", "SDIle", "Leu", "SDLeu", "Pro", "SDPro", "Val", "SDVal", "Ser", "SDSer", "Gly", "SDGly", "Tyr", "SDTyr", "Lys", "SDLys", "Met", "SDMet", "Phe", "SDPhe", "Thr", "SDThr","EAA","SDEAA")
```

```
## δ13C and δ15N data of organic matter sources should be stored in one, single 
## .xlsx file. 

## Columns containing amino acid δ-values and the associated standard 
## deviations should match those defined in the chunk above.

# Indicate the name of the column describing the organic matter source to which
# each sample belongs
Source_Variable <- "Group"
# Indicate the names representing each possible source of organic matter in the
# order in which you would like the referenced
Sources <- c("Surface","Large","Small","Submicron")

# List the name of any additional variables that should be stored for analysis
Additional_variables <- c("Location","Epoch","Event","Size","Sizecat","Depth","Type")

variables <- c(Source_Variable, Additional_variables)

## IMPORTING DATA ##
# Define the location of .xlsx file, and sheet name if multiple sheets are 
# present. If additional data beyond organic matter sources is present in this 
# file that is ok. We will subset the data below.
Data.all <- rbind(
  read_excel("Data/AA-CSIA_ALOHA_Summer.xlsx",
                       sheet = "d15N")[c(variables,tracers,SDtracers)],
  read_excel("Data/AA-CSIA_ALOHA_Winter.xlsx",
                       sheet = "d15N")[c(variables,tracers,SDtracers)]
)
  
# Defining the preferred order in which to reference organic matter sources
Data.all[[Source_Variable]] <- factor(Data.all[[Source_Variable]], levels = c(Sources,"Mixed","Taxa"))

Data.sources <- subset(Data.all, Type == "Particle" | Type == "Trap")
Data.sources$Group <- factor(NA, levels = Sources)
```

# Sample Selection

The first thing we need to do is figure out what is the appropriate minimum depth cutoff for “deep” particles. We want \(\mathrm{\delta^{15}\text{N}}\) values to be essentially invariant with depth delow this cutoff, so let’s have a look at depth trends in particle \(\mathrm{\delta^{15}\text{N}}\) values.

```
Data.sources.long <- 
  melt(
    Data.sources,
    id.vars = variables,
    variable.name = "Tracer",
    value.name = "Value"
  )
Data.sources.long <- Data.sources.long[which(Data.sources.long$Tracer %in% tracers),]

ggplot(data = Data.sources.long)+
  geom_point(
    aes(x=Value, y=Depth, color=Sizecat)
  )+
  facet_wrap(~Tracer, nrow=2, scales = "free_x")+
  scale_y_reverse()
```

Let’s zoom in on the top 500 m.

```
Data.sources.long <- 
  melt(
    Data.sources,
    id.vars = variables,
    variable.name = "Tracer",
    value.name = "Value"
  )
Data.sources.long <- Data.sources.long[which(Data.sources.long$Tracer %in% tracers),]

ggplot(data = Data.sources.long)+
  geom_point(
    aes(x=Value, y=Depth, color=Sizecat)
  )+
  coord_cartesian(ylim = c(500,0))+
  facet_wrap(~Tracer, nrow=2, scales = "free_x")+
  scale_y_reverse()
```

These plots show us that the data becomes mostly invariant with depth at and below about 200 m.

```
## FILTERING DATA ##
Data.sources <- subset(Data.all, Type == "Particle" | Type == "Trap")
Data.sources$Group <- factor(NA, levels = Sources)
Data.sources$Group[
  which(Data.sources$Depth < 100)
  ] <- "Surface"
Data.sources$Group[
  which(Data.sources$Depth > 190 & 
        (Data.sources$Sizecat == "Large" | Data.sources$Sizecat == "Trap"))
  ] <- "Large"
Data.sources$Group[
  which(Data.sources$Depth > 190 & Data.sources$Sizecat == "Small")
  ] <- "Small"
Data.sources$Group[
  which(Data.sources$Depth > 190 & Data.sources$Sizecat == "Submicron")
  ] <- "Submicron"
Data.sources <- subset(Data.sources, !is.na(Group))

Data.zoops <- subset(Data.all,
                     Group == "Mixed" | Group == "Taxa")
```

Next we need to decide which particle samples represent likely sources of organic matter to the zooplankton food web and which do not. We will do this by comparing the \(\mathrm{\delta^{15}\text{N}}\) values of Phe and Lys between zooplankton and particles.

First let’s take a look at all our organic matter sources and decide if there are any we can exclude right off the bat.

```
ggplot()+
  geom_point(data = Data.sources,
             aes(x=d15NPhe, y=d15NLys, color=Group),
             size = 3)+
  geom_point(data = Data.zoops,
             aes(x=d15NPhe, y=d15NLys, shape=Size),
             size = 2)+
  labs(shape="Zooplankton",
       color="Particles")+
  ylab(expression(delta^{15}*N[" Lys"]))+
  xlab(expression(delta^{15}*N[" Phe"]))
   Warning: Removed 1 row containing missing values or values outside the scale range
   (`geom_point()`).
```

```
ggplot()+
  geom_point(data = Data.sources,
             aes(x=d15NPhe, y=d15NThr, color=Group),
             size = 3)+
  geom_point(data = Data.zoops,
             aes(x=d15NPhe, y=d15NThr, shape=Size),
             size = 2)+
  labs(shape="Zooplankton",
       color="Particles")+
  ylab(expression(delta^{15}*N[" Thr"]))+
  xlab(expression(delta^{15}*N[" Phe"]))
   Warning: Removed 1 row containing missing values or values outside the scale range
   (`geom_point()`).
   Warning: Removed 2 rows containing missing values or values outside the scale range
   (`geom_point()`).
```

This suggests that submicron particles are probably not relevant to organic matter supply and can thus be excluded from our analysis. Small particles are also not likely to be very important in most samples, but because they are more abundant and strongly implicated in previous works we will retain them.

Now let’s have a look at surface particles and zooplankton and see if there is some subset of surface particles that are more relevant to our study.

```
ggplot()+
  geom_point(data = subset(Data.sources, 
                           Group == "Surface"),
             aes(x=d15NPhe, y=d15NLys, color=Size),
             size = 3)+
  geom_point(data = subset(Data.zoops, 
                           (Depth < 50)),
             aes(x=d15NPhe, y=d15NLys, shape=interaction(Size,Type)),
             size = 2)+
  labs(shape="Zooplankton",
       color="Particles")+
  ylab(expression(delta^{15}*N[" Lys"]))+
  xlab(expression(delta^{15}*N[" Phe"]))
```

There is really just one >53 μm particle that we can exclude for having a phenylalanine \(\mathrm{\delta^{15}\text{N}}\) value that is higher than all surface zooplankton samples.

```
## FILTERING DATA ##

Data.sources <- subset(Data.all, Type == "Particle" | Type == "Trap")
Data.sources$Group <- factor(NA, levels = Sources)
Data.sources$Group[
  which(Data.sources$Depth < 100 & Data.sources$d15NPhe < 0)
  ] <- "Surface"
Data.sources$Group[
  which(Data.sources$Depth > 190 & 
        (Data.sources$Sizecat == "Large" | Data.sources$Sizecat == "Trap"))
  ] <- "Large"
Data.sources$Group[
  which(Data.sources$Depth > 190 & Data.sources$Sizecat == "Small")
  ] <- "Small"
Data.sources <- subset(Data.sources, !is.na(Group))

## PLOTTING ##

ggplot()+
  geom_point(data = subset(Data.sources, 
                           Group == "Surface"),
             aes(x=d15NPhe, y=d15NLys, color=Size),
             size = 3)+
  geom_point(data = subset(Data.zoops, 
                           (Depth < 100)),
             aes(x=d15NPhe, y=d15NLys, shape=interaction(Size,Type)),
             size = 2)+
  labs(shape="Zooplankton",
       color="Particles")+
  ylab(expression(delta^{15}*N[" Lys"]))+
  xlab(expression(delta^{15}*N[" Phe"]))
```

```
ggplot()+
  geom_point(data = Data.sources,
             aes(x=d15NPhe, y=d15NLys, color=Group),
             size = 3)+
  geom_point(data = Data.zoops,
             aes(x=d15NPhe, y=d15NLys, shape=Size),
             size = 2)+
  labs(shape="Zooplankton",
       color="Particles")+
  ylab(expression(delta^{15}*N[" Lys"]))+
  xlab(expression(delta^{15}*N[" Phe"]))
   Warning: Removed 1 row containing missing values or values outside the scale range
   (`geom_point()`).
```

Given the small amount of trophic discrimination we expect to see in Lys these data makes sense, with the majority of zooplankton falling within the particle phenylalanine \(\mathrm{\delta^{15}\text{N}}\) domain.

Next we need to deal with any gaps that exist in our data. Here will will just omit any samples for which all tracer data is not available.

```
## DEALING WITH GAPS IN THE DATA ##

# Should representative samples of organic matter end members be simulated
# from available data? (useful if a large number of samples have only δ13C or 
# δ15N data). If FALSE, samples without values for all tracers will be excluded.
simulate_rep <- FALSE # SHOULD CHOOSE FALSE BY DEFAULT
Data.sources_orig <- Data.sources

## if we chose not to simulate representative samples...
if (simulate_rep == FALSE) { 
  Data.sources <- na.omit(Data.sources_orig[c(variables,tracers)]) # all samples containing NAs will be removed from data
  ## we will also find the mean tracer value for each source group
  src.mn <- aggregate(Data.sources_orig[tracers], # aggregate source data
                      by=list(Group = Data.sources_orig[[Source_Variable]]), # by organic matter source group
                      FUN = mean, na.rm=TRUE)[-1] # taking a mean
  
## if we chose to simulate representative samples...
} else {
  ## first we will find the mean tracer value for each source group
  src.mn <- aggregate(Data.sources_orig[tracers], # aggregate source data
                      by=list(Group = Data.sources_orig[[Source_Variable]]), # by organic matter source group
                      FUN = mean, na.rm=TRUE)[-1] # taking a mean
  ## Next we will find the most conservative estimate of tracer variance within 
  ## each source group
  # we will calculate the standard deviation within each population
  src.SD.pop <- aggregate(Data.sources_orig[tracers], # aggregate source data
                          by=list(Group = Data.sources_orig[[Source_Variable]]), # by organic matter source group
                          FUN = sd, na.rm=TRUE)[-1] # calculating SD within the population
  colnames(src.SD.pop) <- c(SDtracers)
  # we will also propagate analytical uncertainty through the averaging equation
  src.SD.prop <- aggregate(Data.sources_orig[SDtracers], # aggregate source data SDs
                           by=list(Group = Data.sources_orig[[Source_Variable]]), # by organic matter source group
                           FUN = SDmean, na.rm=TRUE)[-1] # calculating the SD propagated through the averaging equation
  # and we will select the greater of the two estimates of uncertainty
  src.SD <- pmax(src.SD.pop, src.SD.prop, na.rm=TRUE)
  ## Now we will simulate n samples for each organic matter source group
  nsams <- 10 # number of samples to simulate from each source
  Data.sources <- data.frame(matrix(nrow = nsams*length(Sources), ncol = length(tracers)+1)) # initializing empty data frame
  colnames(Data.sources) <- c(tracers,Source_Variable) # naming columns
  set.seed(123) # set seed for repeatable sample synthesis
  for (i in 1:length(tracers)) { # loop over each tracer
    Data.sources[[tracers[i]]] <- rnorm(n = nsams*length(Sources), # pulling n samples from a normal distribution
                                        mean = src.mn[[tracers[i]]], # with a given mean
                                        sd = src.SD[[SDtracers[i]]]) # and given SD
  }
  for (i in variables) {
    Data.sources[[i]] <- seq(1,nsams*length(Sources))
  }
  Data.sources[[Source_Variable]] <- rep(Sources,nsams)
}
```

# Statistical comparison tests

Now we will do a series of statistical tests on our data. We will look at three specific tracer sets:

1. All tracer \(dNAA\) values
2. Just SAA \(\mathrm{\delta^{15}\text{N}\_\text{AA}}\) values (Phe, Lys, Ser, Gly)
3. A select group of \(\mathrm{\delta^{15}\text{N}\_\text{AA}}\) values (Phe, Lys, Thr)

We’ll wan to calculate Person’s correlation coefficients for all tracer pairs, and then for each set of tracers we’ll calculate the effective dimensionality, and fit PCAs and LDAs.

## 1. All Tracers

Here’s the data we are able to work with for these sources of organic matter.

```
kable(Data.sources, format="pipe")
```

| Group | Location | Epoch | Event | Size | Sizecat | Depth | Type | d15NGlx | d15NAla | d15NAsx | d15NIle | d15NLeu | d15NPro | d15NVal | d15NSer | d15NGly | d15NLys | d15NPhe | d15NThr |
| --- | --- | --- | --- | --- | --- | --- | --- | --- | --- | --- | --- | --- | --- | --- | --- | --- | --- | --- | --- |
| Surface | ALOHA S | Summer | Pump Cast | 0.7-53 μm | Small | 25.0 | Particle | 1.484829 | 0.2621566 | 0.6311993 | 0.3282588 | -0.8689297 | 1.3601086 | 0.5503920 | -3.5679724 | -2.7768154 | -3.3241724 | -1.7010684 | -3.2925741 |
| Surface | ALOHA S | Summer | Pump Cast | 1-53 μm | Small | 50.0 | Particle | 2.044472 | 3.4539394 | 1.0298898 | 1.7623601 | -0.9956259 | 0.8069006 | 2.0269545 | -3.9616378 | -1.6631337 | -3.9014198 | -2.9171287 | -3.1572709 |
| Surface | ALOHA S | Summer | Pump Cast | 1-53 μm | Small | 75.0 | Particle | 3.250667 | 3.6536667 | 2.0230000 | 1.5026667 | 0.3960000 | 0.8923333 | 3.6500000 | -1.9240000 | -0.1750000 | -1.0910000 | -0.7136667 | -2.2276667 |
| Small | ALOHA S | Summer | Pump Cast | 1-53 μm | Small | 200.0 | Particle | 13.880354 | 14.1489502 | 10.8244576 | 9.4036231 | 9.3201721 | 9.9939215 | 6.2563283 | 4.5728580 | 6.0175357 | 3.7900082 | 4.8152633 | 0.3038518 |
| Small | ALOHA S | Summer | Pump Cast | 0.7-53 μm | Small | 247.5 | Particle | 14.699000 | 14.2663019 | 10.9235018 | 11.1843429 | 9.4458805 | 10.3536870 | 11.6212750 | 5.9833180 | 4.5618276 | 5.8408147 | 6.3369334 | 1.2524864 |
| Small | ALOHA S | Summer | Pump Cast | 0.7-53 μm | Small | 402.5 | Particle | 16.041232 | 15.1469502 | 12.5842852 | 11.5994349 | 9.4906657 | 10.1706786 | 8.9069479 | 4.6910557 | 7.2411040 | 5.7922405 | 7.5999218 | 1.5328477 |
| Small | ALOHA S | Summer | Pump Cast | 0.7-53 μm | Small | 600.0 | Particle | 16.084200 | 15.8286057 | 12.1225680 | 13.4255673 | 10.3490486 | 10.6624084 | 14.5783106 | 7.1154815 | 6.0129585 | 7.5881735 | 7.5178318 | 2.4000000 |
| Small | ALOHA S | Summer | Pump Cast | 0.7-53 μm | Small | 850.0 | Particle | 15.462667 | 15.5673333 | 12.3956667 | 10.9023333 | 10.5886667 | 11.2226667 | 12.9753333 | 7.4873333 | 6.0143333 | 6.8370000 | 7.4163333 | 2.4030000 |
| Small | ALOHA S | Summer | Pump Cast | 0.7-53 μm | Small | 1200.0 | Particle | 15.833715 | 16.3456049 | 12.5943486 | 12.1666423 | 9.7894775 | 10.7412939 | 14.3863024 | 6.8556156 | 6.3851387 | 6.6023863 | 7.0300927 | 3.4594236 |
| Surface | ALOHA S | Summer | Pump Cast | >53 μm | Large | 25.0 | Particle | 1.167459 | 3.1290725 | -0.0246575 | 0.2931710 | 0.9471575 | -0.2311250 | 1.7367140 | -4.1415565 | 0.6974598 | -1.7106356 | -3.0397050 | 0.5753777 |
| Large | ALOHA S | Summer | Pump Cast | >53 μm | Large | 245.0 | Particle | 8.292695 | 9.4215170 | 5.4600401 | 5.7175542 | 5.8033922 | 5.9414570 | 6.1301973 | 0.9230347 | 3.6817450 | 1.5502729 | 0.3899082 | -2.9458818 |
| Large | ALOHA S | Summer | Pump Cast | >53 μm | Large | 400.0 | Particle | 12.143149 | 11.0651517 | 7.9297650 | 8.9995456 | 8.7545694 | 7.9349008 | 9.3277212 | 2.5105021 | 3.9978206 | 0.8965012 | 1.5617544 | -3.9677564 |
| Large | ALOHA S | Summer | Pump Cast | >53 μm | Large | 600.0 | Particle | 12.181353 | 9.9185662 | 8.7742397 | 8.7404411 | 8.4962629 | 8.5514444 | 8.1667259 | 2.2735137 | 2.6139146 | 2.8256736 | 2.8672322 | -4.8145505 |
| Large | ALOHA S | Summer | Pump Cast | >53 μm | Large | 1200.0 | Particle | 10.343974 | 10.6392268 | 7.2430510 | 6.8627782 | 7.4888370 | 6.6870695 | 7.2719063 | 3.3419059 | 3.8087448 | 1.3385177 | 2.7256373 | -3.7219956 |
| Surface | ALOHA W | Winter | Pump Cast | 0.2-0.7 μm | Submicron | 25.0 | Particle | 2.322083 | 2.4471667 | 1.6890833 | -1.1169167 | -1.3130833 | 3.9470000 | 0.8112500 | -3.5631667 | -3.0676667 | -1.4660000 | -0.9154167 | -0.2457500 |
| Surface | ALOHA W | Winter | Pump Cast | 0.7-53 μm | Small | 25.0 | Particle | 2.027155 | 2.8039849 | 1.1273515 | 0.5149970 | 0.5613993 | 3.0583099 | 1.4477369 | -3.3370298 | -1.6066036 | -1.1066867 | -1.2505243 | -2.0367443 |
| Surface | ALOHA W | Winter | Pump Cast | 1-53 μm | Small | 50.0 | Particle | 2.166333 | 2.5840000 | 1.5290000 | 0.5430000 | -0.6033333 | 0.9650000 | 0.6343333 | -3.5643333 | -0.9803333 | -1.5170000 | -0.6526667 | -2.1916667 |
| Surface | ALOHA W | Winter | Pump Cast | 1-53 μm | Small | 75.0 | Particle | 2.060727 | 1.7198007 | 0.9348772 | -0.1906121 | -1.6836965 | -0.5200061 | -2.2163488 | -3.6222195 | -1.9154255 | -2.5226780 | -3.1584011 | -0.7535370 |
| Small | ALOHA W | Winter | Pump Cast | 1-53 μm | Small | 200.0 | Particle | 13.442670 | 14.5217861 | 9.6566291 | 9.3731502 | 8.4201415 | 7.9392462 | 8.2062151 | 5.2432115 | 4.3260328 | 3.6740667 | 4.1409110 | -0.6041100 |
| Small | ALOHA W | Winter | Pump Cast | 0.7-53 μm | Small | 250.0 | Particle | 13.092000 | 18.3280000 | 10.6310000 | 9.6893333 | 10.1280000 | 11.2886667 | 5.2836667 | 6.5903333 | 7.0386667 | 5.8466667 | 6.6136667 | 3.6270000 |
| Small | ALOHA W | Winter | Pump Cast | 0.7-53 μm | Small | 400.0 | Particle | 14.424954 | 15.5065000 | 11.2179758 | 10.3030000 | 9.4642994 | 10.8963544 | 12.2105000 | 6.8052500 | 6.1265000 | 5.4328426 | 6.4251383 | 3.5220000 |
| Small | ALOHA W | Winter | Pump Cast | 0.7-53 μm | Small | 600.0 | Particle | 15.240750 | 16.3385000 | 12.5092500 | 12.8867500 | 10.8735000 | 11.0805000 | 13.0555000 | 6.8430000 | 5.9295000 | 4.8970000 | 6.5962500 | 3.6400000 |
| Small | ALOHA W | Winter | Pump Cast | 0.7-53 μm | Small | 850.0 | Particle | 15.025667 | 15.1166667 | 11.5986667 | 10.9413333 | 9.5676667 | 10.4040000 | 14.1173333 | 6.9376667 | 5.4343333 | 6.4146667 | 7.4680000 | 3.0073333 |
| Small | ALOHA W | Winter | Pump Cast | 0.7-53 μm | Small | 1195.0 | Particle | 14.499500 | 16.6225000 | 12.3325000 | 11.3030000 | 9.9117500 | 10.8220000 | 11.9137500 | 5.5637500 | 5.0992500 | 3.8030000 | 7.3565000 | 3.8367500 |
| Large | ALOHA W | Winter | Pump Cast | >53 μm | Large | 250.0 | Particle | 11.467638 | 9.6885269 | 6.7369709 | 7.1564848 | 6.4648852 | 6.6199591 | 5.5892913 | 0.8378478 | 2.1776661 | 3.0323176 | 2.6104768 | -3.5351356 |
| Large | ALOHA W | Winter | Pump Cast | >53 μm | Large | 250.0 | Particle | 14.344732 | 14.0970793 | 9.4382148 | 13.9198995 | 12.1231701 | 8.4286556 | 7.8475857 | 4.2032666 | 4.4954637 | 2.0644772 | 3.7648048 | -7.7372552 |
| Large | ALOHA W | Winter | Pump Cast | >53 μm | Large | 850.0 | Particle | 13.018884 | 10.1461334 | 10.3261097 | 9.3815778 | 8.9776616 | 9.2483129 | 9.2769923 | 0.6273126 | 4.5665259 | 2.0559601 | 3.6494712 | -5.3567263 |

Now lets visualize the \(\mathrm{\delta^{15}\text{N}}\) values of each amino acid in each organic matter source.

```
if (include_d13C == TRUE) {
  sources.dC.long <- 
    melt(Data.sources, id.vars=c(variables), measure.vars = intersect(tracers_d13C, tracers),
         variable.name="AA")
  sources.dC.long$AA <- sub('....','',sources.dC.long$AA)
  sources.dC.long$iso <- "d13C"
  if (include_d15N == FALSE) {
    sources.long <- sources.dC.long
  }
}
if (include_d15N == TRUE) {
  sources.dN.long <- 
    melt(Data.sources, id.vars=c(variables), measure.vars = intersect(tracers_d15N, tracers),
         variable.name="AA")
  sources.dN.long$AA <- sub('....','',sources.dN.long$AA)
  sources.dN.long$iso <- "d15N"
  if (include_d13C == FALSE) {
    sources.long <- sources.dN.long
  }
}
if (include_d13C == include_d15N) {
  sources.long <- rbind(sources.dN.long,sources.dC.long)
}
# Defining the preferred order in which to reference amino acids
sources.long$AA <- factor(sources.long$AA, levels = allAA.ord)

theme_set(theme_light()+
            theme(panel.grid.major.x = element_line(colour = NA),
                  panel.grid.major.y = element_line(colour = "grey95"),
                  # axis.text.x = element_text(angle = 60, vjust = 1.6, hjust=1.7),
                  axis.text.x = element_text(angle = 90, vjust = 0.5),
                  axis.ticks.x = element_blank(),
                  axis.title.x = element_blank()))

AA_plots <-
  ggplot(sources.long, aes(x = AA, y = value, color = Group, shape = Group, group=Group))+
  geom_point(alpha=0.6, size=1, position = position_dodge(width = 0.8))+
  ylab(expression(delta^{15}*N*" (\u2030)"))+
  labs(color="Organic Matter Source", shape="Organic Matter Source")+
  theme(axis.title.x = element_blank())+
  coord_cartesian(ylim = c(-9, 19),expand=FALSE)+
  geom_vline(xintercept = seq(0.5,length(sources.long$AA),1), color="grey75", lwd=0.3)
AA_plots
```

### PERMANOVA and Tukey’s pairwise comparison test

Our first task is to conduct a pairwise comparison tests to see which tracers can differentiate which sources. We’ll use PERMANOVA as a multivariate of weather or not different sources have significantly different \(\mathrm{\delta^{15}\text{N}}\) values. We will use Tukey as a pairwise assessment to see which amino acids alone have different \(\mathrm{\delta^{15}\text{N}}\) values between groups. We’ll run these tests first with source group and season as predictors to see if summer vs winter differences are impacting the data. Expand the code chunk to see permanova results.

```
# First doing statistical analyses with particle size and season as predictor variables
adonis2(Data.sources[tracers] ~ Group + Epoch, data=Data.sources, method="euclidean", by="terms")
   Permutation test for adonis under reduced model
   Terms added sequentially (first to last)
   Permutation: free
   Number of permutations: 999
   
   adonis2(formula = Data.sources[tracers] ~ Group + Epoch, data = Data.sources, method = "euclidean", by = "terms")
            Df SumOfSqs      R2        F Pr(>F)    
   Group     2   5707.7 0.91248 122.6062  0.001 ***
   Epoch     1     12.1 0.00194   0.5207  0.504    
   Residual 23    535.4 0.08559                    
   Total    26   6255.2 1.00000                    
   ---
   Signif. codes:  0 '***' 0.001 '**' 0.01 '*' 0.05 '.' 0.1 ' ' 1
Tukey <- list()
par(mar=c(2,8,2,0.5),mfrow=c(6,4),col.main="white")
for (i in tracers) {
  AOV <- aov(Data.sources[[i]] ~ Group + Epoch, data = Data.sources)
  print(i)
  print(summary(AOV))
  TukeyHSD(AOV, conf.level=.95)
  Tukey[[i]] <- TukeyHSD(AOV, conf.level=.95)
  Tukey[[i]]
  plot(Tukey[[i]], las=1, sub=i)
  mtext(i, side=3)
}
   [1] "d15NGlx"
               Df Sum Sq Mean Sq F value Pr(>F)    
   Group        2  799.9   399.9 255.928 <2e-16 ***
   Epoch        1    0.1     0.1   0.087  0.771    
   Residuals   23   35.9     1.6                   
   ---
   Signif. codes:  0 '***' 0.001 '**' 0.01 '*' 0.05 '.' 0.1 ' ' 1
   [1] "d15NAla"
               Df Sum Sq Mean Sq F value Pr(>F)    
   Group        2  829.1   414.5 261.478 <2e-16 ***
   Epoch        1    2.3     2.3   1.423  0.245    
   Residuals   23   36.5     1.6                   
   ---
   Signif. codes:  0 '***' 0.001 '**' 0.01 '*' 0.05 '.' 0.1 ' ' 1
   [1] "d15NAsx"
               Df Sum Sq Mean Sq F value   Pr(>F)    
   Group        2  530.7  265.36 207.861 1.88e-15 ***
   Epoch        1    0.4    0.38   0.299     0.59    
   Residuals   23   29.4    1.28                     
   ---
   Signif. codes:  0 '***' 0.001 '**' 0.01 '*' 0.05 '.' 0.1 ' ' 1
   [1] "d15NIle"
               Df Sum Sq Mean Sq F value   Pr(>F)    
   Group        2  561.4  280.68  96.674 6.39e-12 ***
   Epoch        1    0.0    0.01   0.003    0.955    
   Residuals   23   66.8    2.90                     
   ---
   Signif. codes:  0 '***' 0.001 '**' 0.01 '*' 0.05 '.' 0.1 ' ' 1
   [1] "d15NLeu"
               Df Sum Sq Mean Sq F value   Pr(>F)    
   Group        2  537.1  268.56 168.916 1.78e-14 ***
   Epoch        1    0.2    0.18   0.112    0.741    
   Residuals   23   36.6    1.59                     
   ---
   Signif. codes:  0 '***' 0.001 '**' 0.01 '*' 0.05 '.' 0.1 ' ' 1
   [1] "d15NPro"
               Df Sum Sq Mean Sq F value Pr(>F)    
   Group        2  408.1  204.06 145.218  9e-14 ***
   Epoch        1    1.7    1.68   1.197  0.285    
   Residuals   23   32.3    1.41                   
   ---
   Signif. codes:  0 '***' 0.001 '**' 0.01 '*' 0.05 '.' 0.1 ' ' 1
   [1] "d15NVal"
               Df Sum Sq Mean Sq F value   Pr(>F)    
   Group        2  486.0  242.99  40.268 3.06e-08 ***
   Epoch        1    5.1    5.14   0.852    0.366    
   Residuals   23  138.8    6.03                     
   ---
   Signif. codes:  0 '***' 0.001 '**' 0.01 '*' 0.05 '.' 0.1 ' ' 1
   [1] "d15NSer"
               Df Sum Sq Mean Sq F value   Pr(>F)    
   Group        2  450.5  225.25 206.080 2.07e-15 ***
   Epoch        1    0.0    0.01   0.008    0.928    
   Residuals   23   25.1    1.09                     
   ---
   Signif. codes:  0 '***' 0.001 '**' 0.01 '*' 0.05 '.' 0.1 ' ' 1
   [1] "d15NGly"
               Df Sum Sq Mean Sq F value   Pr(>F)    
   Group        2 257.17  128.58 125.433 4.25e-13 ***
   Epoch        1   1.00    1.00   0.972    0.334    
   Residuals   23  23.58    1.03                     
   ---
   Signif. codes:  0 '***' 0.001 '**' 0.01 '*' 0.05 '.' 0.1 ' ' 1
   [1] "d15NLys"
               Df Sum Sq Mean Sq F value   Pr(>F)    
   Group        2 280.39  140.19 109.604 1.74e-12 ***
   Epoch        1   0.01    0.01   0.006    0.938    
   Residuals   23  29.42    1.28                     
   ---
   Signif. codes:  0 '***' 0.001 '**' 0.01 '*' 0.05 '.' 0.1 ' ' 1
   [1] "d15NPhe"
               Df Sum Sq Mean Sq F value   Pr(>F)    
   Group        2  341.8  170.89  135.59 1.87e-13 ***
   Epoch        1    1.0    1.03    0.82    0.375    
   Residuals   23   29.0    1.26                     
   ---
   Signif. codes:  0 '***' 0.001 '**' 0.01 '*' 0.05 '.' 0.1 ' ' 1
   [1] "d15NThr"
               Df Sum Sq Mean Sq F value   Pr(>F)    
   Group        2 225.60  112.80  49.881 4.32e-09 ***
   Epoch        1   0.29    0.29   0.128    0.724    
   Residuals   23  52.01    2.26                     
   ---
   Signif. codes:  0 '***' 0.001 '**' 0.01 '*' 0.05 '.' 0.1 ' ' 1
```

Seeing that season is not a significant predictor of \(\mathrm{\delta^{15}\text{N}\_\text{AA}}\) values, we will rerun these analysis without differentiating when samples were collected.

```
# Next, seeing that season is not significant, we just use particle size as a predictor
adonis2(Data.sources[tracers] ~ Group, data=Data.sources, method="euclidean", by="terms")
   Permutation test for adonis under reduced model
   Terms added sequentially (first to last)
   Permutation: free
   Number of permutations: 999
   
   adonis2(formula = Data.sources[tracers] ~ Group, data = Data.sources, method = "euclidean", by = "terms")
            Df SumOfSqs      R2     F Pr(>F)    
   Group     2   5707.7 0.91248 125.1  0.001 ***
   Residual 24    547.5 0.08752                 
   Total    26   6255.2 1.00000                 
   ---
   Signif. codes:  0 '***' 0.001 '**' 0.01 '*' 0.05 '.' 0.1 ' ' 1
Tukey <- list()
par(mar=c(2,8,2,0.5),mfrow=c(4,3),col.main="white")
for (i in tracers) {
  AOV <- aov(Data.sources[[i]] ~ Group, data = Data.sources)
  print(i)
  print(summary(AOV))
  TukeyHSD(AOV, conf.level=.95)
  Tukey[[i]] <- TukeyHSD(AOV, conf.level=.95)
  Tukey[[i]]
  plot(Tukey[[i]], las=1, sub=i)
  mtext(i, side=3)
}
   [1] "d15NGlx"
               Df Sum Sq Mean Sq F value Pr(>F)    
   Group        2  799.9   399.9   266.1 <2e-16 ***
   Residuals   24   36.1     1.5                   
   ---
   Signif. codes:  0 '***' 0.001 '**' 0.01 '*' 0.05 '.' 0.1 ' ' 1
   [1] "d15NAla"
               Df Sum Sq Mean Sq F value Pr(>F)    
   Group        2  829.1   414.5   256.9 <2e-16 ***
   Residuals   24   38.7     1.6                   
   ---
   Signif. codes:  0 '***' 0.001 '**' 0.01 '*' 0.05 '.' 0.1 ' ' 1
   [1] "d15NAsx"
               Df Sum Sq Mean Sq F value   Pr(>F)    
   Group        2  530.7  265.36   214.1 4.99e-16 ***
   Residuals   24   29.7    1.24                     
   ---
   Signif. codes:  0 '***' 0.001 '**' 0.01 '*' 0.05 '.' 0.1 ' ' 1
   [1] "d15NIle"
               Df Sum Sq Mean Sq F value   Pr(>F)    
   Group        2  561.4  280.68   100.9 2.09e-12 ***
   Residuals   24   66.8    2.78                     
   ---
   Signif. codes:  0 '***' 0.001 '**' 0.01 '*' 0.05 '.' 0.1 ' ' 1
   [1] "d15NLeu"
               Df Sum Sq Mean Sq F value   Pr(>F)    
   Group        2  537.1  268.56   175.4 4.75e-15 ***
   Residuals   24   36.7    1.53                     
   ---
   Signif. codes:  0 '***' 0.001 '**' 0.01 '*' 0.05 '.' 0.1 ' ' 1
   [1] "d15NPro"
               Df Sum Sq Mean Sq F value   Pr(>F)    
   Group        2  408.1  204.06     144 4.28e-14 ***
   Residuals   24   34.0    1.42                     
   ---
   Signif. codes:  0 '***' 0.001 '**' 0.01 '*' 0.05 '.' 0.1 ' ' 1
   [1] "d15NVal"
               Df Sum Sq Mean Sq F value   Pr(>F)    
   Group        2  486.0     243   40.52 2.02e-08 ***
   Residuals   24  143.9       6                     
   ---
   Signif. codes:  0 '***' 0.001 '**' 0.01 '*' 0.05 '.' 0.1 ' ' 1
   [1] "d15NSer"
               Df Sum Sq Mean Sq F value   Pr(>F)    
   Group        2  450.5  225.25     215 4.77e-16 ***
   Residuals   24   25.1    1.05                     
   ---
   Signif. codes:  0 '***' 0.001 '**' 0.01 '*' 0.05 '.' 0.1 ' ' 1
   [1] "d15NGly"
               Df Sum Sq Mean Sq F value   Pr(>F)    
   Group        2 257.17  128.58   125.6 1.94e-13 ***
   Residuals   24  24.57    1.02                     
   ---
   Signif. codes:  0 '***' 0.001 '**' 0.01 '*' 0.05 '.' 0.1 ' ' 1
   [1] "d15NLys"
               Df Sum Sq Mean Sq F value   Pr(>F)    
   Group        2 280.39  140.19   114.3 5.39e-13 ***
   Residuals   24  29.43    1.23                     
   ---
   Signif. codes:  0 '***' 0.001 '**' 0.01 '*' 0.05 '.' 0.1 ' ' 1
   [1] "d15NPhe"
               Df Sum Sq Mean Sq F value   Pr(>F)    
   Group        2  341.8  170.89   136.6 7.68e-14 ***
   Residuals   24   30.0    1.25                     
   ---
   Signif. codes:  0 '***' 0.001 '**' 0.01 '*' 0.05 '.' 0.1 ' ' 1
   [1] "d15NThr"
               Df Sum Sq Mean Sq F value   Pr(>F)    
   Group        2  225.6  112.80   51.76 1.97e-09 ***
   Residuals   24   52.3    2.18                     
   ---
   Signif. codes:  0 '***' 0.001 '**' 0.01 '*' 0.05 '.' 0.1 ' ' 1
```

### Correlation analysis

Now we need to assess which of these are correlated and thus may only provide redundant information. We will generate a correlation matrix, quantifying colinearity with Pearson’s correlation coefficients.

```
corel <- cor(Data.sources[tracers], method = "pearson")
kable(corel, format = "pipe")
```

|  | d15NGlx | d15NAla | d15NAsx | d15NIle | d15NLeu | d15NPro | d15NVal | d15NSer | d15NGly | d15NLys | d15NPhe | d15NThr |
| --- | --- | --- | --- | --- | --- | --- | --- | --- | --- | --- | --- | --- |
| d15NGlx | 1.0000000 | 0.9633562 | 0.9886089 | 0.9808368 | 0.9765613 | 0.9652689 | 0.9070356 | 0.9551533 | 0.9446843 | 0.9315001 | 0.9491057 | 0.3700737 |
| d15NAla | 0.9633562 | 1.0000000 | 0.9683775 | 0.9529956 | 0.9568742 | 0.9548824 | 0.8724740 | 0.9770930 | 0.9645771 | 0.9355227 | 0.9521047 | 0.5062913 |
| d15NAsx | 0.9886089 | 0.9683775 | 1.0000000 | 0.9654164 | 0.9579344 | 0.9762420 | 0.9121958 | 0.9611684 | 0.9464814 | 0.9403546 | 0.9733671 | 0.4610596 |
| d15NIle | 0.9808368 | 0.9529956 | 0.9654164 | 1.0000000 | 0.9788854 | 0.9340866 | 0.9059659 | 0.9387214 | 0.9305482 | 0.8923038 | 0.9205900 | 0.3198700 |
| d15NLeu | 0.9765613 | 0.9568742 | 0.9579344 | 0.9788854 | 1.0000000 | 0.9491280 | 0.8781665 | 0.9360644 | 0.9506110 | 0.8890549 | 0.9064529 | 0.2885070 |
| d15NPro | 0.9652689 | 0.9548824 | 0.9762420 | 0.9340866 | 0.9491280 | 1.0000000 | 0.8859570 | 0.9462793 | 0.9169509 | 0.9310110 | 0.9581904 | 0.4481009 |
| d15NVal | 0.9070356 | 0.8724740 | 0.9121958 | 0.9059659 | 0.8781665 | 0.8859570 | 1.0000000 | 0.9055753 | 0.8520602 | 0.8857249 | 0.8968807 | 0.4602613 |
| d15NSer | 0.9551533 | 0.9770930 | 0.9611684 | 0.9387214 | 0.9360644 | 0.9462793 | 0.9055753 | 1.0000000 | 0.9342859 | 0.9518558 | 0.9607323 | 0.5365553 |
| d15NGly | 0.9446843 | 0.9645771 | 0.9464814 | 0.9305482 | 0.9506110 | 0.9169509 | 0.8520602 | 0.9342859 | 1.0000000 | 0.9144850 | 0.9134890 | 0.4538430 |
| d15NLys | 0.9315001 | 0.9355227 | 0.9403546 | 0.8923038 | 0.8890549 | 0.9310110 | 0.8857249 | 0.9518558 | 0.9144850 | 1.0000000 | 0.9649867 | 0.5893354 |
| d15NPhe | 0.9491057 | 0.9521047 | 0.9733671 | 0.9205900 | 0.9064529 | 0.9581904 | 0.8968807 | 0.9607323 | 0.9134890 | 0.9649867 | 1.0000000 | 0.5728253 |
| d15NThr | 0.3700737 | 0.5062913 | 0.4610596 | 0.3198700 | 0.2885070 | 0.4481009 | 0.4602613 | 0.5365553 | 0.4538430 | 0.5893354 | 0.5728253 | 1.0000000 |

```
mapcc <- mean(abs(corel[lower.tri(corel)]))

#create pairs plot
pairs.panels(Data.sources[tracers], xaxt="n", yaxt="n", lm=TRUE, smoother=FALSE, stars = FALSE, scale=FALSE, ellipses = FALSE, ci=TRUE, method = "pearson")
```

The mean absolute Pearson’s correlation coefficient for these data is 0.8570597.

### Effective Dimensionality

Let’s also quickly calculate the effective dimensionality of the dataset.

```
ED <- estimate.ED(Data.sources[c(tracers)])
   [1] ED estimated from the correlation matrix; no disattenuation; no small-sample correction.
ED$n1
   [1] 1.66
```

The effective dimensionaltiy of the dataset is 1.66.

### Principal Components Analysis

Now we will do a PCA to produce a 2-D visualization of how these groups separate in this multivariate parameter space.

```
# Fitting PCA and adding Type as a supplimental qualitative variable
PCA.OSP = PCA(Data.sources[c(tracers)], scale.unit = TRUE, graph = FALSE)
# summary(PCA.lit)
# Plot component eigenvalues and print
fviz_eig(PCA.OSP, addlabels = FALSE)
```

```
# Plot PC1&2 results and project variable vectors and print
fviz_pca_biplot(PCA.OSP, axes = c(1,2), col.ind = Data.sources$Group, 
                addlabels=FALSE, addEllipses = TRUE, col.var = 'grey50', repel=TRUE)
```

```
fviz_pca_biplot(PCA.OSP, axes = c(1,3), col.ind = Data.sources$Group, 
                addlabels=FALSE, addEllipses = TRUE, col.var = 'grey50', repel=TRUE)
```

```
# generate 3d plot of PC1 PC2 and PC3
PCA.results <- as.data.frame(PCA.OSP$ind$coord)
PCA.results$Type <- Data.sources$Group

plot_ly(data=PCA.results, x=~Dim.1, y=~Dim.2, z=~Dim.3,
        type="scatter3d", mode="markers", color=PCA.results$Type,
        marker = list(line = list(color = "1", width = 0.5)))
```

```
# Last let's have a look at PCA loadings
p1<-ggplot(aes(x=rownames(PCA.OSP$var$contrib),y=PCA.OSP$var$contrib[,1]),data=NULL)+
    geom_col()+ylab("PC1")+ggtitle("PCA Loadings")+
    theme(axis.text.x = element_blank(),axis.title.x = element_blank(),
          plot.title=element_text(hjust=0.5))
p2<-ggplot(aes(x=rownames(PCA.OSP$var$contrib),y=PCA.OSP$var$contrib[,2]),data=NULL)+
    geom_col()+ylab("PC2")+
    theme(axis.text.x = element_blank(),axis.title.x = element_blank())
p3<-ggplot(aes(x=rownames(PCA.OSP$var$contrib),y=PCA.OSP$var$contrib[,3]),data=NULL)+
    geom_col()+ylab("PC3")+
    theme(axis.text.x = element_text(angle = 60, vjust = 1, hjust=1),
          axis.title.x = element_blank())
ggarrange(p1,p2,p3,nrow=3,heights=c(1.2,1,1.5 ))
```

```
# saving out PCA results for later
PCA.all <- PCA.OSP
```

### Linear Discriminant Analysis

Next we will carry out an LDA to see if that improves group separation.

```
# Defining a data frame with sample type in column 1 and 6 EAA's in columns 2-7.
data.train = Data.sources[c("Group",tracers)]
# Subsetting data to include Microalgae, Fungi, and Bacteria, 
# and not macroalgae, plants, and seagrass
# data.train = subset(data.train, Type == "Microalgae" | Type == "Fungi" | Type == "Bacteria")
ntypes = nlevels(as.factor(data.train$Group))
# fitting the model with leave one out cross validation
LDA.test = lda(Group ~ . ,data = data.train, CV = TRUE,
               prior = rep(1/ntypes, ntypes))
   Warning in lda.default(x, grouping, ...): group Submicron is empty
# print model result
# LDA.train

# create a table which compares the classification of the LDA model to the actual producer type
ct.prod.norm <- table(data.train$Group, 
                      LDA.test$class)
# total percent of samples correctly classified is the sum of the diagonal of this table
noquote(c('% successfully categorized: ', sum(diag(prop.table(ct.prod.norm))))) #85% effective
   [1] % successfully categorized:  0.962962962962963

# Refitting the model using all of the available training data
LDA.full = lda(Group ~ . ,data = data.train, CV = FALSE, prior = rep(1/ntypes, ntypes))
   Warning in lda.default(x, grouping, ...): group Submicron is empty
LDA.full
   Call:
   lda(Group ~ ., data = data.train, CV = FALSE, prior = rep(1/ntypes, 
       ntypes))
   
   Prior probabilities of groups:
   Surface   Large   Small 
      0.25    0.25    0.25 
   
   Group means:
             d15NGlx   d15NAla   d15NAsx    d15NIle   d15NLeu   d15NPro   d15NVal
   Surface  2.065466  2.506723  1.117468  0.4546156 -0.445014  1.284815  1.080129
   Large   11.684632 10.710886  7.986913  8.6826116  8.301254  7.630257  7.658631
   Small   14.810559 15.644808 11.615904 11.0982092  9.779106 10.464619 11.125955
             d15NSer   d15NGly   d15NLys   d15NPhe   d15NThr
   Surface -3.460240 -1.435940 -2.079949 -1.793572 -1.666229
   Large    2.102483  3.620269  1.966246  2.509898 -4.582757
   Small    6.224073  5.848932  5.543239  6.609737  2.365049
   
   Coefficients of linear discriminants:
                   LD1         LD2         LD3
   d15NGlx  1.62520301 -0.61090276 -0.04124058
   d15NAla  0.54643298 -0.67613137  0.91935973
   d15NAsx -0.37756591  0.46811820  0.52708874
   d15NIle -0.78530840  0.81345393  0.01697736
   d15NLeu -0.17903611 -0.50794449 -0.51416226
   d15NPro  0.32368444 -0.06607683 -0.27000541
   d15NVal  0.03705049 -0.38977915  0.11144004
   d15NSer  0.25449266  0.95072197 -0.69750294
   d15NGly  0.31480716  0.28110414 -0.67243509
   d15NLys -0.11084839 -0.05994609  0.05846311
   d15NPhe -0.49079099  0.37333643 -0.13698729
   d15NThr  0.01411409  0.59792649  0.25418698
   
   Proportion of trace:
      LD1    LD2    LD3 
   0.8334 0.1275 0.0391

# save LDA for later use
LDA.all <- LDA.full

# store locations of training data in LD space for later plotting
pred.train = predict(LDA.full, data.train[-1])
class.train = data.frame('Type' = data.train$Group, pred.train$x)


theme_set(theme_light()+
            theme(panel.grid.major.x = element_line(colour = NA),
                  panel.grid.major.y = element_line(colour = "grey95")))

# Generate biplot with Zooplankton overlayed onto training data
plot.mix.LD12 = ggplot(data = class.train, 
                       aes(x = LD2, y = LD1, color = Type), size = 2) + 
  geom_point(alpha = 0.6) + 
  stat_ellipse(type = 't', alpha = 0.6)+
  # geom_point(data = class.zoops, 
  #            aes(x = LD2, y = LD1, shape = Predicted),color = 'black')+
  # geom_text(data = class.zoops,
            # aes(label=Depth),hjust=-0.2, vjust=1.1)+
  # coord_cartesian(xlim=c(-5,5), ylim=c(-7.5,5)) + 
  theme(legend.position = 'none')
plot.mix.LD13 = ggplot(data = class.train, 
                       aes(x = LD3, y = LD1, color = Type), size = 2) + 
  geom_point(alpha = 0.6) +  
  stat_ellipse(type = 't', alpha = 0.6)+
  # geom_point(data = class.zoops, 
  #            aes(x = LD3, y = LD1, shape = Predicted), color = 'black')+
  # geom_text(data = class.zoops,
            # aes(label=Depth),hjust=-0.2, vjust=1.1)+
  # coord_cartesian(xlim=c(-5,5), ylim=c(-7.5,5)) + 
  labs(shape = "Zooplankton") + labs(color = "Training Data")
grid.arrange(ncol = 2, widths = c(1,1.4), plot.mix.LD12, plot.mix.LD13, top = 'Literature Producers') + theme(legend.position = 'top')
```

```
   NULL

# We'll also try and put LD1, 2, and 3 on 3D axes
plot_ly(data=class.train, x=~LD1, y=~LD2, z=~LD3,
        type="scatter3d", mode="markers", color=class.train$Type,
        marker = list(line = list(color = "1", width = 0.5)))
```

```
# Last let's have a look at PCA loadings
p1<-ggplot(aes(x=rownames(LDA.all[[4]]),y=LDA.all[[4]][,1]),data=NULL)+
    geom_col()+ylab("LD1")+ggtitle("LDA Loadings")+
    theme(axis.text.x = element_blank(),axis.title.x = element_blank(),
          plot.title=element_text(hjust=0.5))
p2<-ggplot(aes(x=rownames(LDA.all[[4]]),y=LDA.all[[4]][,2]),data=NULL)+
    geom_col()+ylab("LD2")+
    theme(axis.text.x = element_blank(),axis.title.x = element_blank())
p3<-ggplot(aes(x=rownames(LDA.all[[4]]),y=LDA.all[[4]][,3]),data=NULL)+
    geom_col()+ylab("LD3")+
    theme(axis.text.x = element_text(angle = 60, vjust = 1, hjust=1),
          axis.title.x = element_blank())
ggarrange(p1,p2,p3,nrow=3,heights=c(1.2,1,1.5 ))
```

## 2. Just SAAs

Now we’ll run some of the same multivariate analyses but only looking at \(\mathrm{\delta^{15}\text{N}\_\text{SAA}}\) values.

```
tracers <- tracers_SAA
SDtracers <- SDtracers_SAA
include_d13C <- FALSE
```

```
if (include_d13C == TRUE) {
  sources.dC.long <- 
    melt(Data.sources, id.vars=c(variables), measure.vars = intersect(tracers_d13C, tracers),
         variable.name="AA")
  sources.dC.long$AA <- sub('....','',sources.dC.long$AA)
  sources.dC.long$iso <- "d13C"
  if (include_d15N == FALSE) {
    sources.long <- sources.dC.long
  }
}
if (include_d15N == TRUE) {
  sources.dN.long <- 
    melt(Data.sources, id.vars=c(variables), measure.vars = intersect(tracers_d15N, tracers),
         variable.name="AA")
  sources.dN.long$AA <- sub('....','',sources.dN.long$AA)
  sources.dN.long$iso <- "d15N"
  if (include_d13C == FALSE) {
    sources.long <- sources.dN.long
  }
}
if (include_d13C == include_d15N) {
  sources.long <- rbind(sources.dN.long,sources.dC.long)
}
# Defining the preferred order in which to reference amino acids
sources.long$AA <- factor(sources.long$AA, levels = allAA.ord)

theme_set(theme_light()+
            theme(panel.grid.major.x = element_line(colour = NA),
                  panel.grid.major.y = element_line(colour = "grey95"),
                  axis.text.x = element_text(angle = 60, vjust = 1.6, hjust=1.7),
                  axis.ticks.x = element_blank()))

AA_plots <-
  ggplot(sources.long, aes(x = AA, y = value, color = Group, group=Group))+
  geom_point(alpha=0.6, position = position_dodge(width = 0.8))+
  ylab(expression(delta^{15}*N*"   or   "*delta^{13}*C*" (\u2030)"))+
  labs(color="Organic Matter Source", shape="Food Web Base", fill="Zooplankton Samples")+
  facet_row(~iso, scales="free", space="free")+
  geom_vline(xintercept = seq(0.5,length(sources.long$AA),1), color="grey75", lwd=0.3)
AA_plots
```

### Effective Dimensionality

Let’s also quickly calculate the effective dimensionality of the dataset.

```
ED <- estimate.ED(Data.sources[c(tracers)])
   [1] ED estimated from the correlation matrix; no disattenuation; no small-sample correction.
ED$n1
   [1] 1.25
```

The effective dimensionaltiy of the dataset is 1.25.

### Principal Components Analysis

And a PCA to produce a 2-D visualization of how these groups separate in this multivariate parameter space.

```
# Fitting PCA and adding Type as a supplimental qualitative variable
PCA.OSP = PCA(Data.sources[c(tracers)], scale.unit = TRUE, graph = FALSE)
# summary(PCA.lit)
# Plot component eigenvalues and print
fviz_eig(PCA.OSP, addlabels = FALSE)
```

```
# Plot PC1&2 results and project variable vectors and print
fviz_pca_biplot(PCA.OSP, axes = c(1,2), col.ind = Data.sources$Group, 
                addlabels=FALSE, addEllipses = TRUE, col.var = 'grey50', repel=TRUE)
```

```
fviz_pca_biplot(PCA.OSP, axes = c(1,3), col.ind = Data.sources$Group, 
                addlabels=FALSE, addEllipses = TRUE, col.var = 'grey50', repel=TRUE)
```

```
# generate 3d plot of PC1 PC2 and PC3
PCA.results <- as.data.frame(PCA.OSP$ind$coord)
PCA.results$Type <- Data.sources$Group

plot_ly(data=PCA.results, x=~Dim.1, y=~Dim.2, z=~Dim.3,
        type="scatter3d", mode="markers", color=PCA.results$Type,
        marker = list(line = list(color = "1", width = 0.5)))
```

```
# saving out PCA results for later
PCA.SAA <- PCA.OSP
```

### Linear Discriminant Analysis

Next we will carry out an LDA to see if that improves group separation.

```
# Defining a data frame with sample type in column 1 and 6 EAA's in columns 2-7.
data.train = Data.sources[c("Group",tracers)]
# Subsetting data to include Microalgae, Fungi, and Bacteria, 
# and not macroalgae, plants, and seagrass
# data.train = subset(data.train, Type == "Microalgae" | Type == "Fungi" | Type == "Bacteria")
ntypes = nlevels(as.factor(data.train$Group))
# fitting the model with leave one out cross validation
LDA.test = lda(Group ~ . ,data = data.train, CV = TRUE,
               prior = rep(1/ntypes, ntypes))
   Warning in lda.default(x, grouping, ...): group Submicron is empty
# print model result
# LDA.train

# create a table which compares the classification of the LDA model to the actual producer type
ct.prod.norm <- table(data.train$Group, 
                      LDA.test$class)
# total percent of samples correctly classified is the sum of the diagonal of this table
noquote(c('% successfully categorized: ', sum(diag(prop.table(ct.prod.norm))))) #85% effective
   [1] % successfully categorized:  0.962962962962963

# Refitting the model using all of the available training data
LDA.full = lda(Group ~ . ,data = data.train, CV = FALSE, prior = rep(1/ntypes, ntypes))
   Warning in lda.default(x, grouping, ...): group Submicron is empty
LDA.full
   Call:
   lda(Group ~ ., data = data.train, CV = FALSE, prior = rep(1/ntypes, 
       ntypes))
   
   Prior probabilities of groups:
   Surface   Large   Small 
      0.25    0.25    0.25 
   
   Group means:
             d15NSer   d15NGly   d15NLys   d15NPhe
   Surface -3.460240 -1.435940 -2.079949 -1.793572
   Large    2.102483  3.620269  1.966246  2.509898
   Small    6.224073  5.848932  5.543239  6.609737
   
   Coefficients of linear discriminants:
                  LD1        LD2        LD3
   d15NSer -0.5631996 -0.1983251  0.8680862
   d15NGly -0.4695364  0.8748368 -0.2071318
   d15NLys -0.1260141 -0.2558877  0.1168813
   d15NPhe -0.2005972 -0.3217850 -0.9178335
   
   Proportion of trace:
      LD1    LD2    LD3 
   0.9841 0.0115 0.0044

# save LDA for later use
LDA.SAA <- LDA.full

# store locations of training data in LD space for later plotting
pred.train = predict(LDA.full, data.train[-1])
class.train = data.frame('Type' = data.train$Group, pred.train$x)


theme_set(theme_light()+
            theme(panel.grid.major.x = element_line(colour = NA),
                  panel.grid.major.y = element_line(colour = "grey95")))

# Generate biplot with Zooplankton overlayed onto training data
plot.mix.LD12 = ggplot(data = class.train, 
                       aes(x = LD2, y = LD1, color = Type), size = 2) + 
  geom_point(alpha = 0.6) + 
  stat_ellipse(type = 't', alpha = 0.6)+
  # geom_point(data = class.zoops, 
  #            aes(x = LD2, y = LD1, shape = Predicted),color = 'black')+
  # geom_text(data = class.zoops,
            # aes(label=Depth),hjust=-0.2, vjust=1.1)+
  # coord_cartesian(xlim=c(-5,5), ylim=c(-7.5,5)) + 
  theme(legend.position = 'none')
plot.mix.LD13 = ggplot(data = class.train, 
                       aes(x = LD3, y = LD1, color = Type), size = 2) + 
  geom_point(alpha = 0.6) +  
  stat_ellipse(type = 't', alpha = 0.6)+
  # geom_point(data = class.zoops, 
  #            aes(x = LD3, y = LD1, shape = Predicted), color = 'black')+
  # geom_text(data = class.zoops,
            # aes(label=Depth),hjust=-0.2, vjust=1.1)+
  # coord_cartesian(xlim=c(-5,5), ylim=c(-7.5,5)) + 
  labs(shape = "Zooplankton") + labs(color = "Training Data")
grid.arrange(ncol = 2, widths = c(1,1.4), plot.mix.LD12, plot.mix.LD13, top = 'Literature Producers') + theme(legend.position = 'top')
```

```
   NULL

# We'll also try and put LD1, 2, and 3 on 3D axes
plot_ly(data=class.train, x=~LD1, y=~LD2, z=~LD3,
        type="scatter3d", mode="markers", color=class.train$Type,
        marker = list(line = list(color = "1", width = 0.5)))
```

## 3. Select AA \(\mathrm{\delta^{15}\text{N}}\) values

Now we’ll run some of the same multivariate analyses but looking at a specific selection of amino acid \(\mathrm{\delta^{15}\text{N}}\). Values. This is going to be Phe, Lys, and Thr, since these are the tracers used in the model.

```
tracers <- tracers_select
SDtracers <- SDtracers_select
include_d13C <- FALSE
```

```
if (include_d13C == TRUE) {
  sources.dC.long <- 
    melt(Data.sources, id.vars=c(variables), measure.vars = intersect(tracers_d13C, tracers),
         variable.name="AA")
  sources.dC.long$AA <- sub('....','',sources.dC.long$AA)
  sources.dC.long$iso <- "d13C"
  if (include_d15N == FALSE) {
    sources.long <- sources.dC.long
  }
}
if (include_d15N == TRUE) {
  sources.dN.long <- 
    melt(Data.sources, id.vars=c(variables), measure.vars = intersect(tracers_d15N, tracers),
         variable.name="AA")
  sources.dN.long$AA <- sub('....','',sources.dN.long$AA)
  sources.dN.long$iso <- "d15N"
  if (include_d13C == FALSE) {
    sources.long <- sources.dN.long
  }
}
if (include_d13C == include_d15N) {
  sources.long <- rbind(sources.dN.long,sources.dC.long)
}
# Defining the preferred order in which to reference amino acids
sources.long$AA <- factor(sources.long$AA, levels = allAA.ord)

theme_set(theme_light()+
            theme(panel.grid.major.x = element_line(colour = NA),
                  panel.grid.major.y = element_line(colour = "grey95"),
                  axis.text.x = element_text(angle = 60, vjust = 1.6, hjust=1.7),
                  axis.ticks.x = element_blank()))

AA_plots <-
  ggplot(sources.long, aes(x = AA, y = value, color = Group, group=Group))+
  geom_point(alpha=0.6, position = position_dodge(width = 0.8))+
  ylab(expression(delta^{15}*N*"   or   "*delta^{13}*C*" (\u2030)"))+
  labs(color="Organic Matter Source", shape="Food Web Base", fill="Zooplankton Samples")+
  facet_row(~iso, scales="free", space="free")+
  geom_vline(xintercept = seq(0.5,length(sources.long$AA),1), color="grey75", lwd=0.3)
AA_plots
```

### Effective Dimensionality

Let’s also quickly calculate the effective dimensionality of the dataset.

```
ED <- estimate.ED(Data.sources[c(tracers)])
   [1] ED estimated from the correlation matrix; no disattenuation; no small-sample correction.
ED$n1
   [1] 1.69
```

The effective dimensionaltiy of the dataset is 1.69.

### Principal Components Analysis

PCA to produce a 2-D visualization of how these groups separate in this multivariate parameter space.

```
# Fitting PCA and adding Type as a supplimental qualitative variable
PCA.OSP = PCA(Data.sources[c(tracers)], scale.unit = TRUE, graph = FALSE)
# summary(PCA.lit)
# Plot component eigenvalues and print
fviz_eig(PCA.OSP, addlabels = FALSE)
```

```
# Plot PC1&2 results and project variable vectors and print
fviz_pca_biplot(PCA.OSP, axes = c(1,2), col.ind = Data.sources$Group, 
                addlabels=FALSE, addEllipses = TRUE, col.var = 'grey50', repel=TRUE)
```

```
fviz_pca_biplot(PCA.OSP, axes = c(1,3), col.ind = Data.sources$Group, 
                addlabels=FALSE, addEllipses = TRUE, col.var = 'grey50', repel=TRUE)
```

```
# generate 3d plot of PC1 PC2 and PC3
PCA.results <- as.data.frame(PCA.OSP$ind$coord)
PCA.results$Type <- Data.sources$Group

plot_ly(data=PCA.results, x=~Dim.1, y=~Dim.2, z=~Dim.3,
        type="scatter3d", mode="markers", color=PCA.results$Type,
        marker = list(line = list(color = "1", width = 0.5)))
```

```
# saving out PCA results for later
PCA.select <- PCA.OSP
```

### Linear Discriminant Analysis

Next we will carry out an LDA to see if that improves group separation.

```
# Defining a data frame with sample type in column 1 and 6 EAA's in columns 2-7.
data.train = Data.sources[c("Group",tracers)]
# Subsetting data to include Microalgae, Fungi, and Bacteria, 
# and not macroalgae, plants, and seagrass
# data.train = subset(data.train, Type == "Microalgae" | Type == "Fungi" | Type == "Bacteria")
ntypes = nlevels(as.factor(data.train$Group))
# fitting the model with leave one out cross validation
LDA.test = lda(Group ~ . ,data = data.train, CV = TRUE,
               prior = rep(1/ntypes, ntypes))
   Warning in lda.default(x, grouping, ...): group Submicron is empty
# print model result
# LDA.train

# create a table which compares the classification of the LDA model to the actual producer type
ct.prod.norm <- table(data.train$Group, 
                      LDA.test$class)
# total percent of samples correctly classified is the sum of the diagonal of this table
noquote(c('% successfully categorized: ', sum(diag(prop.table(ct.prod.norm))))) #85% effective
   [1] % successfully categorized:  0.962962962962963

# Refitting the model using all of the available training data
LDA.full = lda(Group ~ . ,data = data.train, CV = FALSE, prior = rep(1/ntypes, ntypes))
   Warning in lda.default(x, grouping, ...): group Submicron is empty
LDA.full
   Call:
   lda(Group ~ ., data = data.train, CV = FALSE, prior = rep(1/ntypes, 
       ntypes))
   
   Prior probabilities of groups:
   Surface   Large   Small 
      0.25    0.25    0.25 
   
   Group means:
             d15NLys   d15NPhe   d15NThr
   Surface -2.079949 -1.793572 -1.666229
   Large    1.966246  2.509898 -4.582757
   Small    5.543239  6.609737  2.365049
   
   Coefficients of linear discriminants:
                 LD1         LD2         LD3
   d15NLys 0.3040489  0.50299556  1.04672643
   d15NPhe 0.6237580 -0.09112708 -0.95327460
   d15NThr 0.1663696 -0.69689051  0.01889849
   
   Proportion of trace:
      LD1    LD2    LD3 
   0.7959 0.2031 0.0010

# save LDA for later use
LDA.select <- LDA.full

# store locations of training data in LD space for later plotting
pred.train = predict(LDA.full, data.train[-1])
class.train = data.frame('Type' = data.train$Group, pred.train$x)


theme_set(theme_light()+
            theme(panel.grid.major.x = element_line(colour = NA),
                  panel.grid.major.y = element_line(colour = "grey95")))

# Generate biplot with Zooplankton overlayed onto training data
plot.mix.LD12 = ggplot(data = class.train, 
                       aes(x = LD2, y = LD1, color = Type), size = 2) + 
  geom_point(alpha = 0.6) + 
  stat_ellipse(type = 't', alpha = 0.6)+
  # geom_point(data = class.zoops, 
  #            aes(x = LD2, y = LD1, shape = Predicted),color = 'black')+
  # geom_text(data = class.zoops,
            # aes(label=Depth),hjust=-0.2, vjust=1.1)+
  # coord_cartesian(xlim=c(-5,5), ylim=c(-7.5,5)) + 
  theme(legend.position = 'none')
plot.mix.LD13 = ggplot(data = class.train, 
                       aes(x = LD3, y = LD1, color = Type), size = 2) + 
  geom_point(alpha = 0.6) +  
  stat_ellipse(type = 't', alpha = 0.6)+
  # geom_point(data = class.zoops, 
  #            aes(x = LD3, y = LD1, shape = Predicted), color = 'black')+
  # geom_text(data = class.zoops,
            # aes(label=Depth),hjust=-0.2, vjust=1.1)+
  # coord_cartesian(xlim=c(-5,5), ylim=c(-7.5,5)) + 
  labs(shape = "Zooplankton") + labs(color = "Training Data")
grid.arrange(ncol = 2, widths = c(1,1.4), plot.mix.LD12, plot.mix.LD13, top = 'Literature Producers') + theme(legend.position = 'top')
```

```
   NULL

# We'll also try and put LD1, 2, and 3 on 3D axes
plot_ly(data=class.train, x=~LD1, y=~LD2, z=~LD3,
        type="scatter3d", mode="markers", color=class.train$Type,
        marker = list(line = list(color = "1", width = 0.5)))
```

## Summary Plots

Next we’re going to generate some plots, summarizing how the our explanatory power changes as a function of the tracers included. To do this we’ll try and plot results from PCAs and LDAs, paired with the variance explained by each dimension.

```
var.exp.PCA <- 
  data.frame("AAs" = 
               factor(c("SAAs","Select d15N","All d15N"),
                      levels = c("SAAs","Select d15N","All d15N")))
var.exp.PCA[1,c("Dim1","Dim2","Dim3")] <- PCA.SAA$eig[1:3,2]
var.exp.PCA[2,c("Dim1","Dim2","Dim3")] <- PCA.select$eig[1:3,2]
var.exp.PCA[3,c("Dim1","Dim2","Dim3")] <- PCA.all$eig[1:3,2]

var.exp.PCA.long <- 
  melt(var.exp.PCA,id.vars = "AAs", value.name = "Variance_Explained", variable.name = "Dimension")

ggplot(data = var.exp.PCA.long, aes(x=AAs, y=Variance_Explained, 
                                    fill = Dimension, group = Dimension))+
  geom_col(position = "fill")+
  ggtitle("Relative Variance Explained by 1st 3 PCA Dimensions")+
  xlab("Tracer Suite") + ylab("% of Variance Explained")+
  scale_fill_manual(values=c("steelblue1","royalblue1","royalblue4"))+
  scale_color_manual(values=c("steelblue1","royalblue1","royalblue4"))
```

```
PCA.results.SAA <- as.data.frame(PCA.SAA$ind$coord)
PCA.results.SAA$Type <- Data.sources$Group

PCA.results.select <- as.data.frame(
  cbind(PCA.select$ind$coord), PCA.select$eig)
PCA.results.select$Type <- Data.sources$Group


PCA.results.all <- as.data.frame(PCA.all$ind$coord)
PCA.results.all$Type <- Data.sources$Group

biplot.SAA <-
  ggplot(data = PCA.results.SAA,
         aes(y = Dim.2, x = Dim.1, color = Type), size = 2) + 
  geom_point(alpha = 0.6) + 
  stat_ellipse(type = 't', alpha = 0.6)+
  ggtitle(expression("SAA "*delta^{15}*"N"))+
  xlab(paste("PC1(",round(var.exp.PCA[1,2],0),"%)",sep = ""))+
  ylab(paste("PC2(",round(var.exp.PCA[1,3],0),"%)",sep = ""))+
  theme_light()+theme(text=element_text(family="serif"))+
  theme(legend.position = 'none')
biplot.select <-
  ggplot(data = PCA.results.select,
         aes(y = Dim.2, x = Dim.1, color = Type), size = 2) + 
  geom_point(alpha = 0.6) + 
  stat_ellipse(type = 't', alpha = 0.6)+
  ggtitle(expression("Select "*delta^{15}*"N"))+
  xlab(paste("PC1(",round(var.exp.PCA[2,2],0),"%)",sep = ""))+
  ylab(paste("PC2(",round(var.exp.PCA[2,3],0),"%)",sep = ""))+
  theme_light()+theme(text=element_text(family="serif"))+
  theme(legend.position = 'none')
biplot.all1 <-
  ggplot(data = PCA.results.all,
         aes(y = Dim.2, x = Dim.1, color = Type), size = 2) + 
  geom_point(alpha = 0.6) + 
  stat_ellipse(type = 't', alpha = 0.6)+
  ggtitle(expression("All "*delta^{15}*"N, "*delta^{13}*"C"))+
  xlab(NULL)+#paste("PC1(",round(var.exp.PCA[3,2],0),"%)",sep = ""))+
  ylab(paste("PC2(",round(var.exp.PCA[4,3],0),"%)",sep = ""))+
  theme_light()+theme(text=element_text(family="serif"))+
  theme(legend.position = 'none')

ggarrange(biplot.SAA,
          biplot.select,
          biplot.all1, 
          ncol = 3)
```

```
var.exp.LDA <- 
  data.frame("AAs" = 
               factor(c("SAAs","Select d15N","All d15N"),
                      levels = c("SAAs","Select d15N","All d15N")))
var.exp.LDA[1,c("Dim1","Dim2","Dim3")] <- LDA.SAA$svd^2/sum(LDA.SAA$svd^2)
var.exp.LDA[2,c("Dim1","Dim2","Dim3")] <- LDA.select$svd^2/sum(LDA.select$svd^2)
var.exp.LDA[3,c("Dim1","Dim2","Dim3")] <- LDA.all$svd^2/sum(LDA.all$svd^2)

var.exp.LDA.long <- 
  melt(var.exp.LDA,id.vars = "AAs", value.name = "Variance_Explained", variable.name = "Dimension")

ggplot(data = var.exp.LDA.long, aes(x=AAs, y=Variance_Explained, 
                                    fill = Dimension, group = Dimension))+
  geom_col()+
  ggtitle("Variance Explained by LDA Dimensions")+
  xlab("Tracer Suite") + ylab("% of Variance Explained")+
  scale_fill_manual(values=c("steelblue1","royalblue1","royalblue4"))+
  scale_color_manual(values=c("steelblue1","royalblue1","royalblue4"))
```

```
data.train = Data.sources[c("Group",tracers_SAA)]
pred.train = predict(LDA.SAA, data.train[-1])
class.train.SAA = data.frame('Type' = data.train$Group, pred.train$x)

data.train = Data.sources[c("Group",tracers_select)]
pred.train = predict(LDA.select, data.train[-1])
class.train.select = data.frame('Type' = data.train$Group, pred.train$x)

data.train = Data.sources[c("Group",tracers_all)]
pred.train = predict(LDA.all, data.train[-1])
class.train.all= data.frame('Type' = data.train$Group, pred.train$x)

biplot.SAA <-
  ggplot(data = class.train.SAA,
         aes(x = LD2, y = LD1, color = Type), size = 2) + 
  geom_point(alpha = 0.6) + 
  stat_ellipse(type = 't', alpha = 0.6)+
  theme(legend.position = 'none')+
  ggtitle("SAA d15N")+
  ylab(paste("LD1(",round(var.exp.LDA[1,2]*100,0),"%)",sep = ""))+
  xlab(paste("LD2(",round(var.exp.LDA[1,3]*100,0),"%)",sep = ""))
biplot.select <-
  ggplot(data = class.train.select,
         aes(x = LD2, y = LD1, color = Type), size = 2) + 
  geom_point(alpha = 0.6) + 
  stat_ellipse(type = 't', alpha = 0.6)+
  theme(legend.position = 'none')+
  ggtitle("Select d15N")+
  ylab(paste("LD1(",round(var.exp.LDA[2,2]*100,0),"%)",sep = ""))+
  xlab(paste("LD2(",round(var.exp.LDA[2,3]*100,0),"%)",sep = ""))
biplot.all <-
  ggplot(data = class.train.all,
         aes(x = LD2, y = LD1, color = Type), size = 2) + 
  geom_point(alpha = 0.6) + 
  stat_ellipse(type = 't', alpha = 0.6)+
  theme(legend.position = 'none')+
  ggtitle("All d15N, d13C")+
  ylab(paste("LD1(",round(var.exp.LDA[4,2]*100,0),"%)",sep = ""))+
  xlab(paste("LD2(",round(var.exp.LDA[4,3]*100,0),"%)",sep = ""))

ggarrange(biplot.SAA,
          biplot.select,
          biplot.all, ncol = 3)
```

Making a plot for the paper.

```
eigens <- data.frame(PCA.select$var$coord)
eigens$AA <- row.names(eigens)

AA.custom <- c(
  as.character(expression(delta^{15}*"N"[Lys])),
  as.character(expression(delta^{15}*"N"[Phe])),
  as.character(expression(delta^{15}*"N"[Thr]))
)
  

biplot.select <-
  ggplot(data = PCA.results.select,
         aes(y = Dim.2, x = Dim.1, color = Type), size = 2) + 
  geom_point(aes(shape = Type), alpha = 1, size=2) +
  stat_ellipse(type = 't', alpha = 0.6, size=0.5)+
  geom_segment(data = eigens,
               aes(x=0, xend = Dim.1*2,
                   y=0, yend =Dim.2*2),
               color = "grey50", size=0.5,
               arrow = arrow(length = unit(0.2, "cm")))+
  geom_text(data = eigens,
               aes(x = Dim.1*2 + c(0.7,0.3,0.2), 
                   y = Dim.2*2 + c(0.15,-0.3,0.2),
                   label = AA.custom),
               color = "grey50", parse = TRUE)+
  labs(color = "Organic Matter Source", shape = "Organic Matter Source")+
  xlab(paste("PC1(",round(var.exp.PCA[2,2],0),"%)",sep = ""))+
  ylab(paste("PC2(",round(var.exp.PCA[2,3],0),"%)",sep = ""))+
  theme_light()+theme(text=element_text(family="serif"))
   Warning: Using `size` aesthetic for lines was deprecated in ggplot2 3.4.0.
   ℹ Please use `linewidth` instead.
   This warning is displayed once every 8 hours.
   Call `lifecycle::last_lifecycle_warnings()` to see where this warning was
   generated.

biplot.select
```
