# Supplementary material for: A Bayesian model for assessing organic matter supply in complex marine food webs using amino acid stable isotope analysis
Source: PeerJ. 2025 Nov 19;13:e20220. doi: 10.7717/peerj.20220 (PMC12640130; doi:10.7717/peerj.20220)
Supplement: Supplemental Information 7 — These are also available at GitHub (https://github.com/CH-Shea/Organic-Matter-Supply-Model). [file peerj-13-20220-s007.zip › Supplemental Files/S4_D15N_Regression_Analysis.html]

Determination of D15N values in wild zooplankton by regression analysis


Code 

- Show All Code
- Hide All Code

# Determination of D15N values in wild zooplankton by regression analysis

#### Connor Shea

#### May 05, 2025

```
## This chunk sets up a working directory, loads packages, defines a couple functions, and sets global chunk options for the .Rmd document

# clean up
rm(list=ls()) 

# fresh start? If FALSE (select 2 in hard bracket), will only rerun a chunk if 
# changes are detected within the text of that chunk.
rmCache <- c(TRUE, FALSE)[2]
tfn <- knitr::current_input()              # name of this file
tfn <- substr(tfn, 1, nchar(tfn)-4)        # Remove the ".Rmd"
tfn_cache <- paste0("Caches/", tfn, "_cache")         # Append "_cache" to it
tfn_figures <- paste0("Figures/", tfn, "_figures")         # Append "_figures" to it
if (rmCache) {
  if (file.exists(tfn_cache))                # If the cache exists...
    unlink(tfn_cache, recursive=TRUE)        # ...delete it.
}

# load packages quietly
shhh <- suppressPackageStartupMessages # It's a library, so shhh!
want <- c("knitr", 
          "readxl", # for reading xl files
          "MASS", # some statistics including lda
          "vegan", # ecology statistics
          "FactoMineR", # includes PCA function and utilities
          "factoextra", # extra utilities for FactoMineR
          "runjags", # To run Bayesian models
          "TruncatedDistributions", # To generate truncate porbability distributions
          "coda",  
          "DT", # makes nice sortable tables
          "openxlsx", # writes to .xlsx files
          "compositions", 
          "kableExtra",
          "zeallot", 
          "magrittr", 
          "DirichletReg", 
          "Ternary", 
          "png",
          "ggplot2",
          "ggpubr",
          "ggstance", # for vertical position dodging
          "ggtern", # for ternary plots in ggplot2
          "ggforce", # for specific extra plotting capabilities (facet_row())
          "plotly",
          "gridExtra",
          "graphics",
          "reshape2",
          "psych"
          )
for (pkg in want) shhh(library(pkg, character.only=TRUE))

# set some chunk options
gr <- (1+sqrt(5))/2 # golden ratio, for figures
opts_chunk$set(comment="  ",
               fig.asp=0.9/gr,       # <--- gr used here
               fig.align="center",
               fig.width=5,
               out.width="95%",
               dpi=96, # set to 300 for important figures
               dev="png", # png, svg, pdf, jpg, tiff
               #echo=FALSE,
               cache=c(TRUE, FALSE)[1], 
               cache.path = paste0(tfn_cache,"/"),
               fig.path = paste0(tfn_figures,"/"),
               eval.after="fig.cap", # for dynamic captions
               collapse=TRUE
               )
## defining additional functions we might need

## a couple of lines we need to run every time we knit
# setting the ggplot theme
theme_set(theme_classic2()+
            theme(panel.grid.major.x = element_line(colour = "grey95"),
                  panel.grid.major.y = element_line(colour = "grey95")))
# defining a ggplot object to remove the x axis
no.x.axis <- theme(axis.title.x=element_blank(),
                   axis.text.x=element_blank(),
                   axis.ticks.x=element_blank())
# defining a ggplot object to remove the y axis
no.y.axis <- theme(axis.title.y=element_blank(),
                   axis.text.y=element_blank(),
                   axis.ticks.y=element_blank())

# Loading up some functions we might need
source("Functions/calcSDs.R") # contains functions SDmean() and SDsum()
source("Functions/Post_Mode.R") # contains function post.mode()
```

# Introduction

The following notebook describes linear regression analysis of wild zooplankton AA-CSIA data with the goal of estimating amino acid TDFs. The regressions are constructed as follows:

1. Zooplankton (0-200 m) and particle (0-100 m) data are aggregated from Station Papa, Station ALOHA, and equatorial sites at 5 N and 8 N.
2. Phe-normalized \(\mathrm{\delta^{15}\text{N}\_\text{AA}}\) values are calculated by subtracting \(\mathrm{\delta^{15}N\_{Phe}}\) from each \(\mathrm{\delta^{15}\text{N}\_\text{AA}}\) value.
3. Trophic positions are calculated by assuming TDFs and beta values for Phe, Glx, and Phe. TDFs are derived from those presented in Table 2, while \(\beta\)-values are from Chikaraishi et al. 2009.
4. To visualize trophic discrimination, Phe-normalized \(\mathrm{\delta^{15}\text{N}\_\text{AA}}\) values are regressed against TP-1.
5. The slope of the regression is equivalent to the TDF. The slope + TDF(Phe) yields the \(\Delta^{15}N\) value.
6. \(\Delta^{15}N\_{AA}\) values are calculated
7. Analytical uncertainty is propagated through all equations.

# Findings

The below analysis indicates significant relationships between all amino acid \(\mathrm{\delta^{15}\text{N}}\) values and TP, except for phenylalanine. Notably, the source amino acids Ser, Gly, and Lys all show significant, positive slopes, indicating trophic discrimination occurring in these AAs in wild zooplankton. From the slopes of those regressions we are able to estimate TDFs, which are in general agreement with those determined in controlled feeding studies. TDFs for Ser, Gly, and Lys are 3.7‰, 2.4‰, and 1.2‰, respectively.

Location does not appear to affect these regressions for most amino acids. The exceptions to this are Val and Pro. Given that it is not a significant effect in the vast majority of the regressions, we exclude interactions between location and TP in regressions used to estimate TDFs.

# Setup

Here we will be defining the functions and constants we will need to analyze the data, importing data, and then coercing that data into the different formats we need.

## Defining Functions and Parameters

First and foremost, let’s gather the parameters that we know we’ll need going forward.

```
## Defining some groups of amino acids

# all amino acids
allAA = c("d15NAla", "d15NGly", "d15NThr", "d15NSer", "d15NVal", "d15NLeu", "d15NIle", "d15NPro", "d15NAsx", "d15NMet", "d15NGlx", "d15NPhe", "d15NTyr", "d15NLys")
# columns with SD
SDallAA <- c("SDd15NAla", "SDd15NGly", "SDd15NThr", "SDd15NSer", "SDd15NVal", "SDd15NLeu", "SDd15NIle", "SDd15NPro", "SDd15NAsx", "SDd15NMet", "SDd15NGlx", "SDd15NPhe", "SDd15NTyr", "SDd15NLys")
# source amino acids
srcAA = c("d15NGly","d15NSer","d15NPhe","d15NLys") # ALL SRC AA: c("d15NPhe","d15NMet","d15NTyr","d15NGly","d15NSer","d15NLys")
# columns with SD
SDsrcAA = c("SDd15NGly","SDd15NSer","SDd15NPhe","SDd15NLys")
# columns with SD
SDsrcAA = c("SDd15NGly","SDd15NSer","SDd15NPhe","SDd15NLys")
# ALL SRC AA: c("SDd15NPhe","SDd15NMet","SDd15NTyr","SDd15NGly","SDd15NSer","SDd15NLys")
# trophic amino acids
trAA = c("d15NGlx", "d15NAsx", "d15NAla", "d15NIle", "d15NLeu", "d15NPro", "d15NVal","d15NThr")
# columns with SD
SDtrAA = c("SDd15NGlx", "SDd15NAsx", "SDd15NAla", "SDd15NIle", "SDd15NLeu", "SDd15NPro", "SDd15NVal","SDd15NThr")
# columns with SD
SDtrAA = c("SDd15NGlx", "SDd15NAsx", "SDd15NAla", "SDd15NIle", "SDd15NLeu", "SDd15NPro", "SDd15NVal","SDd15NThr")

# All amino acids - sorted by SAA TAA and Thr
allAA.ord <- c("d15NGlx", "SDd15NGlx",  "d15NAla", "SDd15NAla", "d15NAsx", "SDd15NAsx", "d15NIle", "SDd15NIle", "d15NLeu", "SDd15NLeu", "d15NPro", "SDd15NPro", "d15NVal", "SDd15NVal", "d15NSer", "SDd15NSer", "d15NGly", "SDd15NGly", "d15NTyr", "SDd15NTyr", "d15NLys", "SDd15NLys", "d15NMet", "SDd15NMet", "d15NPhe", "SDd15NPhe", "d15NThr", "SDd15NThr")

# sometimes will just want the AA names
allAA.names = c("Ala", "Gly", "Thr", "Ser", "Val", "Leu", "Ile", "Pro", "Asx", "Met", "Glx", "Phe", "Tyr", "Lys")
SDallAA.names <- c("SDAla", "SDGly", "SDThr", "SDSer", "SDVal", "SDLeu", "SDIle", "SDPro", "SDAsx", "SDMet", "SDGlx", "SDPhe", "SDTyr", "SDLys")
allAA.ord.names <- c("Glx", "SDGlx",    "Ala", "SDAla", "Asx", "SDAsx", "Ile", "SDIle", "Leu", "SDLeu", "Pro", "SDPro", "Val", "SDVal", "Ser", "SDSer", "Gly", "SDGly", "Tyr", "SDTyr", "Lys", "SDLys", "Met", "SDMet", "Phe", "SDPhe", "Thr", "SDThr")

## defining the TDF data from McMahon et al. 2016.

TP.parms = data.frame("AAs"   = c("glx-Phe", "ala-Phe", "thr-Phe", "trp-src"),
                      "TDF"   = c(7.7      , 6.1      ,-6.2      , 6.3),
                      "SDTDF" = c(1.8      , 2.8      , 3.6      , 0.9),
                      "beta"  = c(3.4      , 3.2      ,-2.4      , 2.2),
                      "SDbeta"= c(0.9      , 1.2      , 0.4      , 0.7)
                      )

DN.comp <-  data.frame("Ala" = 6.3, "SDAla" = 2.6,
                       "Gly" = 2.9, "SDGly" = 3.1,
                       "Thr" =-5.9, "SDThr" = 3.6,
                       "Ser" = 2.6, "SDSer" = 3.2,
                       "Val" = 4.4, "SDVal" = 2.6,
                       "Leu" = 5.6, "SDLeu" = 2.4,
                       "Ile" = 5.5, "SDIle" = 2.4,
                       "Pro" = 5.8, "SDPro" = 1.7,
                       "Asx" = 5.7, "SDAsx" = 1.9,
                       "Met" = 1.6, "SDMet" = 2.6,
                       "Glx" = 8.0, "SDGlx" = 1.7,
                       "Phe" = 0.3, "SDPhe" = 0.5,
                       "Tyr" = NA , "SDTyr" = NA,
                       "Lys" = 1.2, "SDLys" = 1.2)

TDF.comp <- DN.comp
TDF.comp[allAA.names] <- TDF.comp[allAA.names] - DN.comp$Phe

Locs <- c("5N","8N","ALOHA-S","ALOHA-W","OSP")
```

## Data Handling

### Importing Data

We will import all data from 5 N, 8 N, ALOHA-S, ALOHA-W, and OSP. We want this all in one big data frame with columns for descriptive variables, AA \(\mathrm{\delta^{15}\text{N}}\) values, and the standard deviations for each AA \(\mathrm{\delta^{15}\text{N}}\) value.

```
# Importing data from each location
dN.5N <- as.data.frame(read_excel("Data/AA-CSIA_5N.xlsx", 
    sheet = "d15N"))

dN.8N <- as.data.frame(read_excel("Data/AA-CSIA_8N.xlsx", 
    sheet = "d15N"))

dN.ALOHAS <- as.data.frame(read_excel("Data/AA-CSIA_ALOHA_Summer.xlsx", 
    sheet = "d15N"))

dN.ALOHAW <- as.data.frame(read_excel("Data/AA-CSIA_ALOHA_Winter.xlsx", 
    sheet = "d15N"))

dN.OSP <- as.data.frame(read_excel("Data/AA-CSIA_OSP.xlsx", 
    sheet = "d15N"))

# Consolidating into one master data frame
dN.all <- rbind(dN.5N, dN.8N, dN.ALOHAS, dN.ALOHAW, dN.OSP)
# ordering "size" factor to be in ascending order
dN.all$Size = factor(dN.all$Size,
                             c("0.2-0.7 μm","0.3-1 μm","1-5 μm","1-6 μm","0.7-53 μm","1-53 μm","6-51 μm",">51 μm",">53 μm","0.06-0.2 mm","0.2-0.5 mm", "0.5-1.0 mm", "1-2 mm", "2-5 mm", ">5 mm"))
# # Making group = Zooplankton for all zooplankton
# dN.all$Group[dN.all$Type=="Zooplankton"] = "Zooplankton"
# Prioritizing Group to be first
dN.all <- 
  cbind(
    dN.all[,
           c("Group","Type","Sizecat","Size","Depth","Event","Tow","Epoch","Location")],
    dN.all[allAA.ord])
```

### Calculating TP

Next we will be calculating **Trophic position (TP)** via the equation:
\[
\mathrm{
TP = \frac{\delta^{15}N\_{trp} - \delta^{15}N\_{src} - \beta\_{trp-src}}{TDF\_{trp-src}}
}
\]

To assess error in TP we will use the equation from Bradley et al. 2015:

\[\begin{align}
\sigma^2\_{TP} &= \left( \frac{\partial~TP}{\partial~\delta^{15}N\_{trp}} \right)^2 \sigma^2\_{\delta^{15}N\_{trp}} +
\left( \frac{\partial~TP}{\partial~\delta^{15}N\_{src}} \right)^2 \sigma^2\_{\delta^{15}N\_{src}} +
\left( \frac{\partial~TP}{\partial~\beta\_{trp-src}} \right)^2 \sigma^2\_{\beta\_{trp-src}} +
\left( \frac{\partial~TP}{\partial~TDF\_{trp-src}} \right)^2 \sigma^2\_{TDF\_{trp-src}} \\
&= \left( \frac{\sigma\_{\delta^{15}N\_{trp}}}{TDF\_{trp-src}} \right)^2 +
\left( \frac{\sigma\_{\delta^{15}N\_{src}}}{TDF\_{trp-src}} \right)^2 +
\left( \frac{\sigma\_{\beta\_{trp-src}}}{TDF\_{trp-src}} \right)^2 +
\left( \frac{\delta^{15}N\_{src} + \beta\_{trp-src} - \delta^{15}N\_{trp}}{TDF\_{trp-src}^2} \right)^2\sigma^2\_{TDF\_{trp-src}}
\end{align}\]

```
data.all <- dN.all
# Calculating TP_Glx-Phe with SD
data.all$TPGlx <- (data.all$d15NGlx - data.all$d15NPhe - TP.parms$beta[1])/TP.parms$TDF[1]+1
data.all$SDTPGlx <- (data.all$SDd15NGlx/TP.parms$TDF[1])^2+
  (data.all$SDd15NPhe/TP.parms$TDF[1])^2+
  (TP.parms$SDbeta[1]/TP.parms$TDF[1])^2+
  ((data.all$d15NPhe + TP.parms$beta[1] - data.all$d15NGlx)/
     TP.parms$TDF[1]^2)^2 * TP.parms$SDTDF[1]^2

# Calculating TP_Ala-Phe with SD
data.all$TPAla <- (data.all$d15NAla - data.all$d15NPhe - TP.parms$beta[2])/TP.parms$TDF[2]+1
data.all$SDTPAla <- (data.all$SDd15NAla/TP.parms$TDF[2])^2+
  (data.all$SDd15NPhe/TP.parms$TDF[2])^2+
  (TP.parms$SDbeta[2]/TP.parms$TDF[2])^2+
  ((data.all$d15NPhe + TP.parms$beta[2] - data.all$d15NAla)/
     TP.parms$TDF[2]^2)^2 * TP.parms$SDTDF[2]^2
```

### Subsetting the Data

We will subset the data just to try and capture the surface food web. We will include particles above 100 m and zooplankton above 200 m.

```
data.all.phenorm <- data.all
data.all.phenorm[allAA] <- data.all[allAA] - data.all$d15NPhe
data.all.phenorm[SDallAA] <-SDsum(c(data.all[SDallAA], data.all$d15NPhe))

data.reg <- subset(data.all, (Type == "Zooplankton" & Depth < 200) | (Type == "Particle" & Depth < 100))
data.reg.phenorm <- subset(data.all.phenorm, (Type == "Zooplankton" & Depth < 200) | (Type == "Particle" & Depth < 100))

# data.reg.phenorm <- subset(data.all.phenorm, Type == "Zooplankton" |
#                                      (Type == "Particle" & Depth <= 100))
```

# Estimating \(\Delta^{15}N\) values for all AAs from regression analysis

To calculate \(\Delta^{15}N\) values we will regress \(\delta^{15}N\_{AA-Phe}\) against \(\delta^{15}N\_{Glx/Ala-Phe}\). The regression can thus be described by the equation:
\[\begin{align}
\delta^{15}N\_{AA-Phe} = m \cdot \delta^{15}N\_{Glx/Ala-Phe} + b
\end{align}\]
An equation for the slope can thus be written as:
\[\begin{align}
m = \frac{TDF\_{AA-Phe}}{TDF\_{Glx/Ala-Phe}}
= \frac{\Delta^{15}N\_{AA} - \Delta^{15}N\_{Phe}}{TDF\_{Glx/Ala-Phe}}
\end{align}\]
We can then solve for \(\Delta^{15}N\_{AA}\), yielding the equaion:
\[\begin{align}
\Delta^{15}N\_{AA} = m \cdot TDF\_{Glx/Ala-Phe} + \Delta^{15}N\_{Phe}
\end{align}\]

Propagation of uncertainty through this equation is then takes the form:
\[\begin{align}
\sigma(\Delta^{15}N\_{AA})^2 =& \sigma(m \cdot TDF\_{Glx/Ala-Phe})^2 + \sigma(\Delta^{15}N\_{Phe})^2 \\
=& m^2 \cdot TDF\_{Glx/Ala-Phe}^2
\left[\left(\frac{\sigma(m)}{m}\right)^2 +
\left(\frac{\sigma(TDF\_{Glx/Ala-Phe})}{TDF\_{Glx/Ala-Phe}}\right)^2\right] +
\sigma(\Delta^{15}N\_{Phe})^2 \\
=& \left(TDF\_{Glx/Ala-Phe} \cdot \sigma(m)\right)^2 +
\left(m \cdot \sigma(TDF\_{Glx/Ala-Phe})\right)^2 +
\sigma(\Delta^{15}N\_{Phe})^2
\end{align}\]
or
\[\begin{align}
\sigma(\Delta^{15}N\_{AA}) = \sqrt{
\left(TDF\_{Glx/Ala-Phe} \cdot \sigma(m)\right)^2 +
\left(m \cdot \sigma(TDF\_{Glx/Ala-Phe})\right)^2 +
\sigma(\Delta^{15}N\_{Phe})^2
}
\end{align}\]

We will go ahead and calculate TDFs in that manner and present the results in a data table.

```
DN_Glx <- DN.comp$Glx
SDDN_Glx <- DN.comp$SDGlx
DN_Ala <-  DN.comp$Ala
SDDN_Ala <-  DN.comp$SDAla
DN_Phe <- DN.comp$Phe
SDDN_Phe <-  DN.comp$SDPhe
DN_GA <- c(DN.comp$Glx,DN.comp$Ala)
SDDN_GA <- c(DN.comp$SDGlx,DN.comp$SDAla)
TDF_GA <- c(TDF.comp$Glx,TDF.comp$Ala)
SDTDF_GA <- c(TDF.comp$SDGlx,TDF.comp$SDAla)

DN.reg = data.frame(Ala=c(NA,NA))
## Ala 
LM <- lm(d15NAla ~ d15NGlx + Location, data = data.reg.phenorm)
out1 <- summary(LM)
anova(LM)
   Analysis of Variance Table
   
   Response: d15NAla
             Df  Sum Sq Mean Sq   F value  Pr(>F)    
   d15NGlx    1 2864.64 2864.64 1527.5151 < 2e-16 ***
   Location   4   24.77    6.19    3.3023 0.01429 *  
   Residuals 89  166.91    1.88                      
   ---
   Signif. codes:  0 '***' 0.001 '**' 0.01 '*' 0.05 '.' 0.1 ' ' 1
# LM <- lm(d15NAla ~ d15NAla + Location, data = data.reg.phenorm)
# out2 <- summary(LM)
m_reg <- c(out1$coefficients[2,1],NA)
SDm_reg <- c(out1$coefficients[2,2],NA)

DN.reg$Ala <- m_reg*TDF_GA + DN_Phe
DN.reg$SDAla <- sqrt((TDF_GA * SDm_reg)^2 + (m_reg * SDTDF_GA)^2 + DN.comp$Phe^2)

## Gly 
LM <- lm(d15NGly ~ d15NGlx + Location, data = data.reg.phenorm)
out1 <- summary(LM)
anova(LM)
   Analysis of Variance Table
   
   Response: d15NGly
             Df Sum Sq Mean Sq F value    Pr(>F)    
   d15NGlx    1 421.86  421.86  144.23 < 2.2e-16 ***
   Location   4 172.69   43.17   14.76 2.748e-09 ***
   Residuals 89 260.32    2.92                      
   ---
   Signif. codes:  0 '***' 0.001 '**' 0.01 '*' 0.05 '.' 0.1 ' ' 1
LM <- lm(d15NGly ~ d15NAla + Location, data = data.reg.phenorm)
out2 <- summary(LM)
anova(LM)
   Analysis of Variance Table
   
   Response: d15NGly
             Df Sum Sq Mean Sq F value    Pr(>F)    
   d15NAla    1 419.05  419.05 130.463 < 2.2e-16 ***
   Location   4 168.86   42.21  13.143 1.798e-08 ***
   Residuals 90 289.08    3.21                      
   ---
   Signif. codes:  0 '***' 0.001 '**' 0.01 '*' 0.05 '.' 0.1 ' ' 1
m_reg <- c(out1$coefficients[2,1],out2$coefficients[2,1])
SDm_reg <- c(out1$coefficients[2,2],out2$coefficients[2,2])

DN.reg$Gly <- m_reg*TDF_GA + DN_Phe
DN.reg$SDGly <- sqrt((TDF_GA * SDm_reg)^2 + (m_reg * SDTDF_GA)^2 + DN.comp$Phe^2)

## Thr 
LM <- lm(d15NThr ~ d15NGlx + Location, data = data.reg.phenorm)
out1 <- summary(LM)
anova(LM)
   Analysis of Variance Table
   
   Response: d15NThr
             Df  Sum Sq Mean Sq F value    Pr(>F)    
   d15NGlx    1 1203.79 1203.79 103.079 < 2.2e-16 ***
   Location   4  665.88  166.47  14.255 4.965e-09 ***
   Residuals 89 1039.37   11.68                      
   ---
   Signif. codes:  0 '***' 0.001 '**' 0.01 '*' 0.05 '.' 0.1 ' ' 1
LM <- lm(d15NThr ~ d15NAla + Location, data = data.reg.phenorm)
out2 <- summary(LM)
anova(LM)
   Analysis of Variance Table
   
   Response: d15NThr
             Df  Sum Sq Mean Sq F value    Pr(>F)    
   d15NAla    1 1353.72 1353.72 113.284 < 2.2e-16 ***
   Location   4  529.03  132.26  11.068 2.407e-07 ***
   Residuals 90 1075.48   11.95                      
   ---
   Signif. codes:  0 '***' 0.001 '**' 0.01 '*' 0.05 '.' 0.1 ' ' 1
m_reg <- c(out1$coefficients[2,1],out2$coefficients[2,1])
SDm_reg <- c(out1$coefficients[2,2],out2$coefficients[2,2])

DN.reg$Thr <- m_reg*TDF_GA + DN_Phe
DN.reg$SDThr <- sqrt((TDF_GA * SDm_reg)^2 + (m_reg * SDTDF_GA)^2 + DN.comp$Phe^2)

## Ser 
LM <- lm(d15NSer ~ d15NGlx + Location, data = data.reg.phenorm)
out1 <- summary(LM)
anova(LM)
   Analysis of Variance Table
   
   Response: d15NSer
             Df  Sum Sq Mean Sq F value    Pr(>F)    
   d15NGlx    1 1049.55 1049.55 396.957 < 2.2e-16 ***
   Location   4  207.14   51.79  19.586 1.368e-11 ***
   Residuals 89  235.32    2.64                      
   ---
   Signif. codes:  0 '***' 0.001 '**' 0.01 '*' 0.05 '.' 0.1 ' ' 1
LM <- lm(d15NSer ~ d15NAla + Location, data = data.reg.phenorm)
out2 <- summary(LM)
anova(LM)
   Analysis of Variance Table
   
   Response: d15NSer
             Df  Sum Sq Mean Sq F value    Pr(>F)    
   d15NAla    1 1028.24 1028.24 337.350 < 2.2e-16 ***
   Location   4  208.19   52.05  17.076 1.878e-10 ***
   Residuals 90  274.32    3.05                      
   ---
   Signif. codes:  0 '***' 0.001 '**' 0.01 '*' 0.05 '.' 0.1 ' ' 1
m_reg <- c(out1$coefficients[2,1],out2$coefficients[2,1])
SDm_reg <- c(out1$coefficients[2,2],out2$coefficients[2,2])

DN.reg$Ser <- m_reg*TDF_GA + DN_Phe
DN.reg$SDSer <- sqrt((TDF_GA * SDm_reg)^2 + (m_reg * SDTDF_GA)^2 + DN.comp$Phe^2)

## Val 
LM <- lm(d15NVal ~ d15NGlx + Location, data = data.reg.phenorm)
out1 <- summary(LM)
anova(LM)
   Analysis of Variance Table
   
   Response: d15NVal
             Df  Sum Sq Mean Sq  F value    Pr(>F)    
   d15NGlx    1 1683.00 1683.00 356.4649 < 2.2e-16 ***
   Location   4  169.81   42.45   8.9918 3.807e-06 ***
   Residuals 89  420.20    4.72                       
   ---
   Signif. codes:  0 '***' 0.001 '**' 0.01 '*' 0.05 '.' 0.1 ' ' 1
LM <- lm(d15NVal ~ d15NAla + Location, data = data.reg.phenorm)
out2 <- summary(LM)
anova(LM)
   Analysis of Variance Table
   
   Response: d15NVal
             Df  Sum Sq Mean Sq  F value    Pr(>F)    
   d15NAla    1 1708.55 1708.55 323.9688 < 2.2e-16 ***
   Location   4  148.73   37.18   7.0505 5.525e-05 ***
   Residuals 90  474.64    5.27                       
   ---
   Signif. codes:  0 '***' 0.001 '**' 0.01 '*' 0.05 '.' 0.1 ' ' 1
m_reg <- c(out1$coefficients[2,1],out2$coefficients[2,1])
SDm_reg <- c(out1$coefficients[2,2],out2$coefficients[2,2])

DN.reg$Val <- m_reg*TDF_GA + DN_Phe
DN.reg$SDVal <- sqrt((TDF_GA * SDm_reg)^2 + (m_reg * SDTDF_GA)^2 + DN.comp$Phe^2)

## Leu 
LM <- lm(d15NLeu ~ d15NGlx + Location, data = data.reg.phenorm)
out1 <- summary(LM)
anova(LM)
   Analysis of Variance Table
   
   Response: d15NLeu
             Df  Sum Sq Mean Sq   F value    Pr(>F)    
   d15NGlx    1 1826.02 1826.02 1044.8068 < 2.2e-16 ***
   Location   4   59.53   14.88    8.5153 7.275e-06 ***
   Residuals 89  155.55    1.75                        
   ---
   Signif. codes:  0 '***' 0.001 '**' 0.01 '*' 0.05 '.' 0.1 ' ' 1
LM <- lm(d15NLeu ~ d15NAla + Location, data = data.reg.phenorm)
out2 <- summary(LM)
anova(LM)
   Analysis of Variance Table
   
   Response: d15NLeu
             Df  Sum Sq Mean Sq  F value    Pr(>F)    
   d15NAla    1 1898.48 1898.48 983.5455 < 2.2e-16 ***
   Location   4   45.98   11.49   5.9549 0.0002685 ***
   Residuals 90  173.72    1.93                       
   ---
   Signif. codes:  0 '***' 0.001 '**' 0.01 '*' 0.05 '.' 0.1 ' ' 1
m_reg <- c(out1$coefficients[2,1],out2$coefficients[2,1])
SDm_reg <- c(out1$coefficients[2,2],out2$coefficients[2,2])

DN.reg$Leu <- m_reg*TDF_GA + DN_Phe
DN.reg$SDLeu <- sqrt((TDF_GA * SDm_reg)^2 + (m_reg * SDTDF_GA)^2 + DN.comp$Phe^2)

## Ile 
LM <- lm(d15NIle ~ d15NGlx + Location, data = data.reg.phenorm)
out1 <- summary(LM)
anova(LM)
   Analysis of Variance Table
   
   Response: d15NIle
             Df  Sum Sq Mean Sq  F value  Pr(>F)    
   d15NGlx    1 2064.63 2064.63 640.5408 < 2e-16 ***
   Location   4   35.04    8.76   2.7174 0.03465 *  
   Residuals 89  286.87    3.22                     
   ---
   Signif. codes:  0 '***' 0.001 '**' 0.01 '*' 0.05 '.' 0.1 ' ' 1
LM <- lm(d15NIle ~ d15NAla + Location, data = data.reg.phenorm)
out2 <- summary(LM)
anova(LM)
   Analysis of Variance Table
   
   Response: d15NIle
             Df  Sum Sq Mean Sq  F value  Pr(>F)    
   d15NAla    1 2148.33 2148.33 670.0281 < 2e-16 ***
   Location   4   33.71    8.43   2.6287 0.03954 *  
   Residuals 90  288.57    3.21                     
   ---
   Signif. codes:  0 '***' 0.001 '**' 0.01 '*' 0.05 '.' 0.1 ' ' 1
m_reg <- c(out1$coefficients[2,1],out2$coefficients[2,1])
SDm_reg <- c(out1$coefficients[2,2],out2$coefficients[2,2])

DN.reg$Ile <- m_reg*TDF_GA + DN_Phe
DN.reg$SDIle <- sqrt((TDF_GA * SDm_reg)^2 + (m_reg * SDTDF_GA)^2 + DN.comp$Phe^2)

## Pro 
LM <- lm(d15NPro ~ d15NGlx + Location, data = data.reg.phenorm)
out1 <- summary(LM)
anova(LM)
   Analysis of Variance Table
   
   Response: d15NPro
             Df  Sum Sq Mean Sq  F value Pr(>F)    
   d15NGlx    1 1338.42 1338.42 657.3678 <2e-16 ***
   Location   4    7.66    1.92   0.9411 0.4441    
   Residuals 89  181.21    2.04                    
   ---
   Signif. codes:  0 '***' 0.001 '**' 0.01 '*' 0.05 '.' 0.1 ' ' 1
LM <- lm(d15NPro ~ d15NAla + Location, data = data.reg.phenorm)
out2 <- summary(LM)
anova(LM)
   Analysis of Variance Table
   
   Response: d15NPro
             Df  Sum Sq Mean Sq F value Pr(>F)    
   d15NAla    1 1311.02 1311.02 508.746 <2e-16 ***
   Location   4    8.40    2.10   0.815 0.5189    
   Residuals 90  231.93    2.58                   
   ---
   Signif. codes:  0 '***' 0.001 '**' 0.01 '*' 0.05 '.' 0.1 ' ' 1
m_reg <- c(out1$coefficients[2,1],out2$coefficients[2,1])
SDm_reg <- c(out1$coefficients[2,2],out2$coefficients[2,2])

DN.reg$Pro <- m_reg*TDF_GA + DN_Phe
DN.reg$SDPro <- sqrt((TDF_GA * SDm_reg)^2 + (m_reg * SDTDF_GA)^2 + DN.comp$Phe^2)

## Asx 
LM <- lm(d15NAsx ~ d15NGlx + Location, data = data.reg.phenorm)
out1 <- summary(LM)
anova(LM)
   Analysis of Variance Table
   
   Response: d15NAsx
             Df  Sum Sq Mean Sq   F value  Pr(>F)    
   d15NGlx    1 1497.82 1497.82 2104.2383 < 2e-16 ***
   Location   4    8.37    2.09    2.9388 0.02479 *  
   Residuals 89   63.35    0.71                      
   ---
   Signif. codes:  0 '***' 0.001 '**' 0.01 '*' 0.05 '.' 0.1 ' ' 1
LM <- lm(d15NAsx ~ d15NAla + Location, data = data.reg.phenorm)
out2 <- summary(LM)
anova(LM)
   Analysis of Variance Table
   
   Response: d15NAsx
             Df  Sum Sq Mean Sq   F value    Pr(>F)    
   d15NAla    1 1511.37 1511.37 1508.2904 < 2.2e-16 ***
   Location   4   18.33    4.58    4.5742  0.002079 ** 
   Residuals 90   90.18    1.00                        
   ---
   Signif. codes:  0 '***' 0.001 '**' 0.01 '*' 0.05 '.' 0.1 ' ' 1
m_reg <- c(out1$coefficients[2,1],out2$coefficients[2,1])
SDm_reg <- c(out1$coefficients[2,2],out2$coefficients[2,2])

DN.reg$Asx <- m_reg*TDF_GA + DN_Phe
DN.reg$SDAsx <- sqrt((TDF_GA * SDm_reg)^2 + (m_reg * SDTDF_GA)^2 + DN.comp$Phe^2)

## Met -- SKIP
# LM <- lm(d15NMet ~ d15NGlx + Location, data = data.reg.phenorm)
# out1 <- summary(LM)
# LM <- lm(d15NMet ~ d15NAla + Location, data = data.reg.phenorm)
# out2 <- summary(LM)
m_reg <- c(NA,NA)
SDm_reg <- c(NA,NA)

DN.reg$Met <- m_reg*TDF_GA + DN_Phe
DN.reg$SDMet <- sqrt((TDF_GA * SDm_reg)^2 + (m_reg * SDTDF_GA)^2 + DN.comp$Phe^2)

## Glx
# LM <- lm(d15NGlx ~ d15NGlx + Location, data = data.reg.phenorm)
# out1 <- summary(LM)
LM <- lm(d15NGlx ~ d15NAla + Location, data = data.reg.phenorm)
out2 <- summary(LM)
anova(LM)
   Analysis of Variance Table
   
   Response: d15NGlx
             Df  Sum Sq Mean Sq   F value    Pr(>F)    
   d15NAla    1 2362.10 2362.10 1573.5426 < 2.2e-16 ***
   Location   4   24.45    6.11    4.0723  0.004452 ** 
   Residuals 89  133.60    1.50                        
   ---
   Signif. codes:  0 '***' 0.001 '**' 0.01 '*' 0.05 '.' 0.1 ' ' 1
m_reg <- c(NA,out2$coefficients[2,1])
SDm_reg <- c(NA,out2$coefficients[2,2])

DN.reg$Glx <- m_reg*TDF_GA + DN_Phe
DN.reg$SDGlx <- sqrt((TDF_GA * SDm_reg)^2 + (m_reg * SDTDF_GA)^2 + DN.comp$Phe^2)

## Phe -- SKIP
# LM <- lm(d15NPhe ~ d15NGlx + Location, data = subset(data.all, Depth <= 100))
# out1 <- summary(LM)
# anova(LM)
# LM <- lm(d15NPhe ~ d15NAla + Location, data = subset(data.all, Depth <= 100))
# out2 <- summary(LM)
anova(LM)
   Analysis of Variance Table
   
   Response: d15NGlx
             Df  Sum Sq Mean Sq   F value    Pr(>F)    
   d15NAla    1 2362.10 2362.10 1573.5426 < 2.2e-16 ***
   Location   4   24.45    6.11    4.0723  0.004452 ** 
   Residuals 89  133.60    1.50                        
   ---
   Signif. codes:  0 '***' 0.001 '**' 0.01 '*' 0.05 '.' 0.1 ' ' 1
m_reg <- c(NA,NA)
SDm_reg <- c(NA,NA)

DN.reg$Phe <- m_reg
DN.reg$SDPhe <- SDm_reg

## Tyr
LM <- lm(d15NTyr ~ d15NGlx + Location, data = data.reg.phenorm)
out1 <- summary(LM)
anova(LM)
   Analysis of Variance Table
   
   Response: d15NTyr
             Df Sum Sq Mean Sq F value    Pr(>F)    
   d15NGlx    1 137.79 137.787 27.8623 1.115e-06 ***
   Location   4  86.81  21.703  4.3885  0.002956 ** 
   Residuals 79 390.68   4.945                      
   ---
   Signif. codes:  0 '***' 0.001 '**' 0.01 '*' 0.05 '.' 0.1 ' ' 1
LM <- lm(d15NTyr ~ d15NAla + Location, data = data.reg.phenorm)
out2 <- summary(LM)
anova(LM)
   Analysis of Variance Table
   
   Response: d15NTyr
             Df Sum Sq Mean Sq F value    Pr(>F)    
   d15NAla    1 104.07 104.074 19.8460 2.698e-05 ***
   Location   4  94.40  23.601  4.5005  0.002487 ** 
   Residuals 80 419.52   5.244                      
   ---
   Signif. codes:  0 '***' 0.001 '**' 0.01 '*' 0.05 '.' 0.1 ' ' 1
m_reg <- c(out1$coefficients[2,1],out2$coefficients[2,1])
SDm_reg <- c(out1$coefficients[2,2],out2$coefficients[2,2])

DN.reg$Tyr <- m_reg*TDF_GA + DN_Phe
DN.reg$SDTyr <- sqrt((TDF_GA * SDm_reg)^2 + (m_reg * SDTDF_GA)^2 + DN.comp$Phe^2)

## Lys
LM <- lm(d15NLys ~ d15NGlx + Location, data = data.reg.phenorm)
out1 <- summary(LM)
anova(LM)
   Analysis of Variance Table
   
   Response: d15NLys
             Df  Sum Sq Mean Sq F value    Pr(>F)    
   d15NGlx    1  62.422  62.422  19.845 2.434e-05 ***
   Location   4  81.294  20.323   6.461 0.0001303 ***
   Residuals 89 279.953   3.146                      
   ---
   Signif. codes:  0 '***' 0.001 '**' 0.01 '*' 0.05 '.' 0.1 ' ' 1
LM <- lm(d15NLys ~ d15NAla + Location, data = data.reg.phenorm)
out2 <- summary(LM)
anova(LM)
   Analysis of Variance Table
   
   Response: d15NLys
             Df  Sum Sq Mean Sq F value    Pr(>F)    
   d15NAla    1  65.216  65.216 20.7591 1.631e-05 ***
   Location   4  75.861  18.965  6.0369 0.0002382 ***
   Residuals 90 282.742   3.142                      
   ---
   Signif. codes:  0 '***' 0.001 '**' 0.01 '*' 0.05 '.' 0.1 ' ' 1
m_reg <- c(out1$coefficients[2,1],out2$coefficients[2,1])
SDm_reg <- c(out1$coefficients[2,2],out2$coefficients[2,2])

DN.reg$Lys <- m_reg*TDF_GA + DN_Phe
DN.reg$SDLys <- sqrt((TDF_GA * SDm_reg)^2 + (m_reg * SDTDF_GA)^2 + DN.comp$Phe^2)


DN.compare <- round(rbind(DN.reg,DN.comp),2)
DN.compare <- cbind(Method = c("Regression vs TP(Glx-Phe)","Regression vs TP(Ala-Phe)","Feeding Study"),
                     DN.compare)
datatable(DN.compare)
```

```
write.xlsx(DN.compare, "~/University_of_Hawaii/SIA_work/EXPORTS/Data/FWM-results/DNs_regression-analysis.xlsx", overwrite=TRUE)
```

Now, we’ll try and summarize these DN comparisons in a nice figure.

```
# DN.compare.long$Alpha <- 1

DN.compare.long <- melt(DN.compare[1:3,c("Method",allAA.names)],
                         id.vars = c("Method"), variable.name = "AA")
DN.compare.long.SD <- melt(DN.compare[1:3,c("Method",SDallAA.names)],
                         id.vars = c("Method"), variable.name = "AA")
DN.compare.long$SD <- DN.compare.long.SD$value
DN.compare.long$AA <- factor(DN.compare.long$AA, levels = allAA.ord.names)

DN.compare.long$Alpha <- 0.5
AA.constTDF <- 
  c("Phe", "Ala", "Pro", "Ser", "Gly", "Lys")
AA.varTDF <- 
  c("Glx", "Asx", "Leu", "Thr")
DN.compare.long$Alpha[
  which(DN.compare.long$AA %in% AA.constTDF & 
        DN.compare.long$Method == "Regression vs TP(Ala-Phe)")
] <- 1
DN.compare.long$Alpha[
  which(DN.compare.long$AA %in% AA.varTDF & 
        DN.compare.long$Method == "Regression vs TP(Glx-Phe)")
] <- 1
DN.compare.long$Alpha[
  which(DN.compare.long$Method == "Feeding Study")
] <- 1

labs <-  c(
  "Feeding Study",
  expression("Regression vs "*delta^{15}*"N"[Ala-Phe]),
  expression("Regression vs "*delta^{15}*"N"[Glx-Phe])
)
ggplot(data = DN.compare.long,
       aes(x = AA, y = value, 
           ymax = value+SD, ymin = value-SD,
           color = Method, shape = Method,
           alpha = Alpha))+
  geom_hline(yintercept=0, alpha=0.5)+
  geom_point(position = position_dodge(width = 0.5))+
  geom_errorbar(width=0.2, position = position_dodge(width = 0.5))+
  scale_alpha_continuous(range = c(0.2,1))+
  ylab(expression(Delta^{15}*"N"[AA]*"(‰)") )+
  labs(color="",shape="")+guides(alpha="none")+
  scale_color_discrete(labels = labs)+
  scale_shape_discrete(labels = labs)+
  theme_light()+
  theme(legend.position="bottom",axis.title.x=element_blank())
   Warning: Removed 7 rows containing missing values or values outside the scale range
   (`geom_point()`).
```

# Plots and Statistics

We’re also going to generate plots visualizing regressions for each amino acid against TP. We will prot regressings against TP(Ala-Phe) and TP(Glx-Phe).

We’ll also run some statistical tests.

1. Does location have an effect on the slope of the regression?
2. Assuming no, is the slope significant?
3. What is 3. The \(R^2\) value associated with the regression?

## Regression for Ala

```
noquote("Checking to see if location affects the slope of the regression...")
   [1] Checking to see if location affects the slope of the regression...

noquote("Regressing d15N(Ala-Phe) against TP(Glx-Phe)")
   [1] Regressing d15N(Ala-Phe) against TP(Glx-Phe)
LM.dN = lm(d15NAla ~ (TPGlx-1) * Location, data = data.reg.phenorm)
summary(LM.dN)
   
   Call:
   lm(formula = d15NAla ~ (TPGlx - 1) * Location, data = data.reg.phenorm)
   
   Residuals:
       Min      1Q  Median      3Q     Max 
   -2.8018 -0.8382  0.2267  0.6375  4.1224 
   
   Coefficients:
                         Estimate Std. Error t value Pr(>|t|)    
   TPGlx                   7.5465     0.5915  12.758  < 2e-16 ***
   Location5 N            -4.1888     1.4641  -2.861 0.005313 ** 
   Location8 N            -3.8034     1.5318  -2.483 0.014997 *  
   LocationALOHA S        -4.2981     1.2072  -3.560 0.000609 ***
   LocationALOHA W        -4.0982     1.0354  -3.958 0.000156 ***
   LocationOSP            -5.8515     0.7081  -8.263 1.67e-12 ***
   TPGlx:Location8 N       0.1976     0.8483   0.233 0.816388    
   TPGlx:LocationALOHA S   0.8152     0.8186   0.996 0.322110    
   TPGlx:LocationALOHA W   0.4856     0.7586   0.640 0.523812    
   TPGlx:LocationOSP       1.4250     0.6794   2.097 0.038937 *  
   ---
   Signif. codes:  0 '***' 0.001 '**' 0.01 '*' 0.05 '.' 0.1 ' ' 1
   
   Residual standard error: 1.349 on 85 degrees of freedom
     (3 observations deleted due to missingness)
   Multiple R-squared:  0.9918, Adjusted R-squared:  0.9909 
   F-statistic:  1031 on 10 and 85 DF,  p-value: < 2.2e-16
anova(LM.dN)
   Analysis of Variance Table
   
   Response: d15NAla
                  Df  Sum Sq Mean Sq    F value    Pr(>F)    
   TPGlx           1 18548.2 18548.2 10195.0468 < 2.2e-16 ***
   Location        5   199.7    39.9    21.9565  4.68e-14 ***
   TPGlx:Location  4    12.3     3.1     1.6852    0.1609    
   Residuals      85   154.6     1.8                         
   ---
   Signif. codes:  0 '***' 0.001 '**' 0.01 '*' 0.05 '.' 0.1 ' ' 1
coef.1 <- c(
  LM.dN$coefficients[1],
  LM.dN$coefficients[1] + LM.dN$coefficients[7],
  LM.dN$coefficients[1] + LM.dN$coefficients[8],
  LM.dN$coefficients[1] + LM.dN$coefficients[9],
  LM.dN$coefficients[1] + LM.dN$coefficients[10]
)
p1 <- anova(LM.dN)[3,5]
p1 <- anova(LM.dN)[1,5]

noquote("Regressing d15N(Ala-Phe) against TP(Ala-Phe)")
   [1] Regressing d15N(Ala-Phe) against TP(Ala-Phe)
LM.dN = lm(d15NAla ~ (TPAla-1) * Location, data = data.reg.phenorm)
summary(LM.dN)
   Warning in summary.lm(LM.dN): essentially perfect fit: summary may be
   unreliable
   
   Call:
   lm(formula = d15NAla ~ (TPAla - 1) * Location, data = data.reg.phenorm)
   
   Residuals:
          Min         1Q     Median         3Q        Max 
   -1.063e-14 -1.046e-15  1.000e-17  8.620e-16  4.851e-14 
   
   Coefficients:
                           Estimate Std. Error    t value Pr(>|t|)    
   TPAla                  6.100e+00  2.074e-15  2.941e+15   <2e-16 ***
   Location5 N           -2.900e+00  5.937e-15 -4.885e+14   <2e-16 ***
   Location8 N           -2.900e+00  6.324e-15 -4.586e+14   <2e-16 ***
   LocationALOHA S       -2.900e+00  4.266e-15 -6.797e+14   <2e-16 ***
   LocationALOHA W       -2.900e+00  4.080e-15 -7.108e+14   <2e-16 ***
   LocationOSP           -2.900e+00  2.584e-15 -1.122e+15   <2e-16 ***
   TPAla:Location8 N     -4.247e-15  2.929e-15 -1.450e+00   0.1506    
   TPAla:LocationALOHA S -5.107e-15  2.625e-15 -1.946e+00   0.0550 .  
   TPAla:LocationALOHA W -4.642e-15  2.568e-15 -1.808e+00   0.0741 .  
   TPAla:LocationOSP     -4.377e-15  2.289e-15 -1.912e+00   0.0592 .  
   ---
   Signif. codes:  0 '***' 0.001 '**' 0.01 '*' 0.05 '.' 0.1 ' ' 1
   
   Residual standard error: 5.893e-15 on 86 degrees of freedom
     (2 observations deleted due to missingness)
   Multiple R-squared:      1,  Adjusted R-squared:      1 
   F-statistic: 5.452e+31 on 10 and 86 DF,  p-value: < 2.2e-16
anova(LM.dN)
   Warning in anova.lm(...): ANOVA F-tests on an essentially perfect fit are
   unreliable
   Analysis of Variance Table
   
   Response: d15NAla
                  Df  Sum Sq Mean Sq    F value Pr(>F)    
   TPAla           1 18838.2 18838.2 5.4247e+32 <2e-16 ***
   Location        5    94.1    18.8 5.4172e+29 <2e-16 ***
   TPAla:Location  4     0.0     0.0 1.1287e+00 0.3484    
   Residuals      86     0.0     0.0                      
   ---
   Signif. codes:  0 '***' 0.001 '**' 0.01 '*' 0.05 '.' 0.1 ' ' 1
coef.2 <- c(
  LM.dN$coefficients[1],
  LM.dN$coefficients[1] + LM.dN$coefficients[7],
  LM.dN$coefficients[1] + LM.dN$coefficients[8],
  LM.dN$coefficients[1] + LM.dN$coefficients[9],
  LM.dN$coefficients[1] + LM.dN$coefficients[10]
)
p2 <- anova(LM.dN)[3,5]
   Warning in anova.lm(...): ANOVA F-tests on an essentially perfect fit are
   unreliable
R2 <- summary(LM.dN)$adj.r.squared
   Warning in summary.lm(LM.dN): essentially perfect fit: summary may be
   unreliable

noquote("Now re-running the regression without location interaction")
   [1] Now re-running the regression without location interaction

noquote("Regressing d15N(Ala-Phe) against TP(Glx-Phe)")
   [1] Regressing d15N(Ala-Phe) against TP(Glx-Phe)
LM.dN = lm(d15NAla ~ (TPGlx-1) + Location, data = data.reg.phenorm)
summary(LM.dN)
   
   Call:
   lm(formula = d15NAla ~ (TPGlx - 1) + Location, data = data.reg.phenorm)
   
   Residuals:
       Min      1Q  Median      3Q     Max 
   -2.9890 -0.8216  0.0981  0.8563  4.0098 
   
   Coefficients:
                   Estimate Std. Error t value Pr(>|t|)    
   TPGlx             8.3614     0.2161  38.689  < 2e-16 ***
   Location5 N      -6.1184     0.6704  -9.127 2.05e-14 ***
   Location8 N      -5.3078     0.6585  -8.060 3.27e-12 ***
   LocationALOHA S  -4.2975     0.5544  -7.752 1.40e-11 ***
   LocationALOHA W  -4.7835     0.5485  -8.721 1.42e-13 ***
   LocationOSP      -4.6242     0.4896  -9.445 4.51e-15 ***
   ---
   Signif. codes:  0 '***' 0.001 '**' 0.01 '*' 0.05 '.' 0.1 ' ' 1
   
   Residual standard error: 1.369 on 89 degrees of freedom
     (3 observations deleted due to missingness)
   Multiple R-squared:  0.9912, Adjusted R-squared:  0.9906 
   F-statistic:  1666 on 6 and 89 DF,  p-value: < 2.2e-16
anova(LM.dN)
   Analysis of Variance Table
   
   Response: d15NAla
             Df  Sum Sq Mean Sq  F value    Pr(>F)    
   TPGlx      1 18548.2 18548.2 9890.484 < 2.2e-16 ***
   Location   5   199.7    39.9   21.301 5.952e-14 ***
   Residuals 89   166.9     1.9                       
   ---
   Signif. codes:  0 '***' 0.001 '**' 0.01 '*' 0.05 '.' 0.1 ' ' 1
coef.1 <- c(
  LM.dN$coefficients[1]
)
p3 <- anova(LM.dN)[1,5]
R3 <- summary(LM.dN)$adj.r.squared


ggplot(data = data.reg.phenorm, aes(x = (TPGlx-1), y = d15NAla))+
  geom_point(aes(color=Location))+
  geom_smooth(method=lm, aes(y = predict(LM.dN, data.reg.phenorm), color=Location), lty=1, se=TRUE)+
  xlab(expression(TP[Glx-Phe]-1))+
  ylab(expression(delta^{15}*N[Ala-Phe]*" (\u2030)"))+
  theme_light()+
  facet_wrap(~ Location)
   `geom_smooth()` using formula = 'y ~ x'
   Warning: Removed 3 rows containing non-finite outside the scale range
   (`stat_smooth()`).
   Warning: Removed 3 rows containing missing values or values outside the scale range
   (`geom_point()`).
```

```
noquote("Regressing d15N(Ala-Phe) against TP(Ala-Phe)")
   [1] Regressing d15N(Ala-Phe) against TP(Ala-Phe)
LM.dN = lm(d15NAla ~ (TPAla-1) + Location, data = data.reg.phenorm)
summary(LM.dN)
   Warning in summary.lm(LM.dN): essentially perfect fit: summary may be
   unreliable
   
   Call:
   lm(formula = d15NAla ~ (TPAla - 1) + Location, data = data.reg.phenorm)
   
   Residuals:
          Min         1Q     Median         3Q        Max 
   -8.896e-15 -1.200e-15  5.300e-17  1.025e-15  5.058e-14 
   
   Coefficients:
                     Estimate Std. Error    t value Pr(>|t|)    
   TPAla            6.100e+00  6.532e-16  9.338e+15   <2e-16 ***
   Location5 N     -2.900e+00  2.578e-15 -1.125e+15   <2e-16 ***
   Location8 N     -2.900e+00  2.572e-15 -1.128e+15   <2e-16 ***
   LocationALOHA S -2.900e+00  2.150e-15 -1.349e+15   <2e-16 ***
   LocationALOHA W -2.900e+00  2.144e-15 -1.352e+15   <2e-16 ***
   LocationOSP     -2.900e+00  1.886e-15 -1.537e+15   <2e-16 ***
   ---
   Signif. codes:  0 '***' 0.001 '**' 0.01 '*' 0.05 '.' 0.1 ' ' 1
   
   Residual standard error: 5.91e-15 on 90 degrees of freedom
     (2 observations deleted due to missingness)
   Multiple R-squared:      1,  Adjusted R-squared:      1 
   F-statistic: 9.035e+31 on 6 and 90 DF,  p-value: < 2.2e-16
anova(LM.dN)
   Warning in anova.lm(...): ANOVA F-tests on an essentially perfect fit are
   unreliable
   Analysis of Variance Table
   
   Response: d15NAla
             Df  Sum Sq Mean Sq    F value    Pr(>F)    
   TPAla      1 18838.2 18838.2 5.3938e+32 < 2.2e-16 ***
   Location   5    94.1    18.8 5.3864e+29 < 2.2e-16 ***
   Residuals 90     0.0     0.0                         
   ---
   Signif. codes:  0 '***' 0.001 '**' 0.01 '*' 0.05 '.' 0.1 ' ' 1
coef.2 <- c(
  LM.dN$coefficients[1]
)
p4 <- anova(LM.dN)[1,5]
   Warning in anova.lm(...): ANOVA F-tests on an essentially perfect fit are
   unreliable
R4 <- summary(LM.dN)$adj.r.squared
   Warning in summary.lm(LM.dN): essentially perfect fit: summary may be
   unreliable

ggplot(data = data.reg.phenorm, aes(x = (TPAla-1), y = d15NAla))+
  geom_point(aes(color=Location))+
  geom_smooth(method=lm, aes(y = predict(LM.dN, data.reg.phenorm), color=Location), lty=1, se=TRUE)+
  xlab(expression(TP[Ala-Phe]-1))+
  ylab(expression(delta^{15}*N[Ala-Phe]*" (\u2030)"))+
  theme_light()+
  facet_wrap(~ Location)
   `geom_smooth()` using formula = 'y ~ x'
   Warning: Removed 2 rows containing non-finite outside the scale range
   (`stat_smooth()`).
   Warning: Removed 2 rows containing missing values or values outside the scale range
   (`geom_point()`).
```

```
DN.reg <- data.frame(
  "TP(Glx-Phe)" = coef.1,
  "TP(Ala-Phe)" = coef.2,
  row.names = "d15NAla"
)

DN.reg
           TP.Glx.Phe. TP.Ala.Phe.
   d15NAla    8.361447         6.1
```

1. We tested to see if location had a significant effect on the slope of the regression. The P values associated with this test were 2.6805725^{-90} and 0.3484227 for TP(Glx-Phe) and TP(Ala-Phe), respectively. This indicates that location MIGHT have a significant effect on the slope of the regression.
2. Removing the interaction with location, we tested to see if the slope is significant. The P value associated with these tests were 5.193651^{-93} and 0. This indicates that the slope of the regression IS significant.
3. The \(R^2\) values were 0.990581 and 1, respectively.

## Regression for Gly

```
noquote("Checking to see if location affects the slope of the regression...")
   [1] Checking to see if location affects the slope of the regression...

noquote("Regressing d15N(Gly-Phe) against TP(Glx-Phe)")
   [1] Regressing d15N(Gly-Phe) against TP(Glx-Phe)
LM.dN = lm(d15NGly ~ (TPGlx-1) * Location, data = data.reg.phenorm)
summary(LM.dN)
   
   Call:
   lm(formula = d15NGly ~ (TPGlx - 1) * Location, data = data.reg.phenorm)
   
   Residuals:
       Min      1Q  Median      3Q     Max 
   -4.6149 -1.3710  0.0104  1.0063  4.1593 
   
   Coefficients:
                         Estimate Std. Error t value Pr(>|t|)    
   TPGlx                   3.2759     0.7569   4.328 4.08e-05 ***
   Location5 N            -4.3887     1.8733  -2.343  0.02148 *  
   Location8 N            -3.7168     1.9600  -1.896  0.06132 .  
   LocationALOHA S        -0.8961     1.5447  -0.580  0.56334    
   LocationALOHA W        -4.0449     1.3248  -3.053  0.00302 ** 
   LocationOSP            -5.3632     0.9061  -5.919 6.63e-08 ***
   TPGlx:Location8 N      -0.3414     1.0854  -0.315  0.75385    
   TPGlx:LocationALOHA S  -0.9331     1.0474  -0.891  0.37549    
   TPGlx:LocationALOHA W   0.4618     0.9706   0.476  0.63541    
   TPGlx:LocationOSP      -0.3238     0.8694  -0.372  0.71045    
   ---
   Signif. codes:  0 '***' 0.001 '**' 0.01 '*' 0.05 '.' 0.1 ' ' 1
   
   Residual standard error: 1.726 on 85 degrees of freedom
     (3 observations deleted due to missingness)
   Multiple R-squared:  0.8229, Adjusted R-squared:  0.8021 
   F-statistic: 39.49 on 10 and 85 DF,  p-value: < 2.2e-16
anova(LM.dN)
   Analysis of Variance Table
   
   Response: d15NGly
                  Df Sum Sq Mean Sq  F value    Pr(>F)    
   TPGlx           1 843.01  843.01 283.0222 < 2.2e-16 ***
   Location        5 326.21   65.24  21.9038  4.95e-14 ***
   TPGlx:Location  4   7.14    1.78   0.5991    0.6643    
   Residuals      85 253.18    2.98                       
   ---
   Signif. codes:  0 '***' 0.001 '**' 0.01 '*' 0.05 '.' 0.1 ' ' 1
coef.1 <- c(
  LM.dN$coefficients[1],
  LM.dN$coefficients[1] + LM.dN$coefficients[7],
  LM.dN$coefficients[1] + LM.dN$coefficients[8],
  LM.dN$coefficients[1] + LM.dN$coefficients[9],
  LM.dN$coefficients[1] + LM.dN$coefficients[10]
)
p1 <- anova(LM.dN)[3,5] 
R1 <- summary(LM.dN)$adj.r.squared

noquote("Regressing d15N(Gly-Phe) against TP(Ala-Phe)")
   [1] Regressing d15N(Gly-Phe) against TP(Ala-Phe)
LM.dN = lm(d15NGly ~ (TPAla-1) * Location, data = data.reg.phenorm)
summary(LM.dN)
   
   Call:
   lm(formula = d15NGly ~ (TPAla - 1) * Location, data = data.reg.phenorm)
   
   Residuals:
       Min      1Q  Median      3Q     Max 
   -4.7615 -1.1969 -0.0109  1.0101  3.9083 
   
   Coefficients:
                         Estimate Std. Error t value Pr(>|t|)    
   TPAla                  2.65492    0.63709   4.167 7.32e-05 ***
   Location5 N           -3.84804    1.82374  -2.110   0.0378 *  
   Location8 N           -3.13477    1.94263  -1.614   0.1103    
   LocationALOHA S       -1.65300    1.31055  -1.261   0.2106    
   LocationALOHA W       -2.84534    1.25324  -2.270   0.0257 *  
   LocationOSP           -4.13774    0.79363  -5.214 1.26e-06 ***
   TPAla:Location8 N     -0.42481    0.89967  -0.472   0.6380    
   TPAla:LocationALOHA S -0.57066    0.80637  -0.708   0.4811    
   TPAla:LocationALOHA W -0.06868    0.78871  -0.087   0.9308    
   TPAla:LocationOSP     -0.75044    0.70303  -1.067   0.2888    
   ---
   Signif. codes:  0 '***' 0.001 '**' 0.01 '*' 0.05 '.' 0.1 ' ' 1
   
   Residual standard error: 1.81 on 86 degrees of freedom
     (2 observations deleted due to missingness)
   Multiple R-squared:  0.8036, Adjusted R-squared:  0.7807 
   F-statistic: 35.18 on 10 and 86 DF,  p-value: < 2.2e-16
anova(LM.dN)
   Analysis of Variance Table
   
   Response: d15NGly
                  Df Sum Sq Mean Sq  F value    Pr(>F)    
   TPAla           1 851.75  851.75 259.9254 < 2.2e-16 ***
   Location        5 293.86   58.77  17.9354 3.819e-12 ***
   TPAla:Location  4   7.27    1.82   0.5543    0.6964    
   Residuals      86 281.81    3.28                       
   ---
   Signif. codes:  0 '***' 0.001 '**' 0.01 '*' 0.05 '.' 0.1 ' ' 1
coef.2 <- c(
  LM.dN$coefficients[1],
  LM.dN$coefficients[1] + LM.dN$coefficients[7],
  LM.dN$coefficients[1] + LM.dN$coefficients[8],
  LM.dN$coefficients[1] + LM.dN$coefficients[9],
  LM.dN$coefficients[1] + LM.dN$coefficients[10]
)
p2 <- anova(LM.dN)[3,5] 
R2 <- summary(LM.dN)$adj.r.squared


noquote("Now re-running the regression without location interaction")
   [1] Now re-running the regression without location interaction

noquote("Regressing d15N(Gly-Phe) against TP(Glx-Phe)")
   [1] Regressing d15N(Gly-Phe) against TP(Glx-Phe)
LM.dN = lm(d15NGly ~ (TPGlx-1) + Location, data = data.reg.phenorm)
summary(LM.dN)
   
   Call:
   lm(formula = d15NGly ~ (TPGlx - 1) + Location, data = data.reg.phenorm)
   
   Residuals:
       Min      1Q  Median      3Q     Max 
   -4.6970 -1.1366 -0.1267  1.0528  4.2213 
   
   Coefficients:
                   Estimate Std. Error t value Pr(>|t|)    
   TPGlx             3.0635     0.2699  11.350  < 2e-16 ***
   Location5 N      -3.8857     0.8372  -4.641 1.19e-05 ***
   Location8 N      -4.0312     0.8224  -4.902 4.24e-06 ***
   LocationALOHA S  -2.3763     0.6923  -3.432 0.000910 ***
   LocationALOHA W  -2.6420     0.6850  -3.857 0.000217 ***
   LocationOSP      -5.5873     0.6115  -9.138 1.95e-14 ***
   ---
   Signif. codes:  0 '***' 0.001 '**' 0.01 '*' 0.05 '.' 0.1 ' ' 1
   
   Residual standard error: 1.71 on 89 degrees of freedom
     (3 observations deleted due to missingness)
   Multiple R-squared:  0.8179, Adjusted R-squared:  0.8056 
   F-statistic: 66.62 on 6 and 89 DF,  p-value: < 2.2e-16
anova(LM.dN)
   Analysis of Variance Table
   
   Response: d15NGly
             Df Sum Sq Mean Sq F value    Pr(>F)    
   TPGlx      1 843.01  843.01 288.215 < 2.2e-16 ***
   Location   5 326.21   65.24  22.306 1.982e-14 ***
   Residuals 89 260.32    2.92                      
   ---
   Signif. codes:  0 '***' 0.001 '**' 0.01 '*' 0.05 '.' 0.1 ' ' 1
coef.1 <- c(
  LM.dN$coefficients[1]
)
p3 <- anova(LM.dN)[1,5] 
R3 <- summary(LM.dN)$adj.r.squared

ggplot(data = data.reg.phenorm, aes(x = (TPGlx-1), y = d15NGly))+
  geom_point(aes(color=Location))+
  geom_smooth(method=lm, aes(y = predict(LM.dN, data.reg.phenorm), color=Location), lty=1, se=TRUE)+
  xlab(expression(TP[Glx-Phe]-1))+
  ylab(expression(delta^{15}*N[Gly-Phe]*" (\u2030)"))+
  theme_light()+
  facet_wrap(~ Location)
   `geom_smooth()` using formula = 'y ~ x'
   Warning: Removed 3 rows containing non-finite outside the scale range
   (`stat_smooth()`).
   Warning: Removed 3 rows containing missing values or values outside the scale range
   (`geom_point()`).
```

```
noquote("Regressing d15N(Gly-Phe) against TP(Ala-Phe)")
   [1] Regressing d15N(Gly-Phe) against TP(Ala-Phe)
LM.dN = lm(d15NGly ~ (TPAla-1) + Location, data = data.reg.phenorm)
summary(LM.dN)
   
   Call:
   lm(formula = d15NGly ~ (TPAla - 1) + Location, data = data.reg.phenorm)
   
   Residuals:
       Min      1Q  Median      3Q     Max 
   -4.9672 -1.0579 -0.0176  1.1293  4.2612 
   
   Coefficients:
                   Estimate Std. Error t value Pr(>|t|)    
   TPAla             2.1665     0.1981  10.937  < 2e-16 ***
   Location5 N      -2.5206     0.7817  -3.224 0.001759 ** 
   Location8 N      -2.9475     0.7798  -3.780 0.000282 ***
   LocationALOHA S  -1.8592     0.6520  -2.852 0.005392 ** 
   LocationALOHA W  -1.7780     0.6503  -2.734 0.007530 ** 
   LocationOSP      -4.7863     0.5720  -8.367 7.09e-13 ***
   ---
   Signif. codes:  0 '***' 0.001 '**' 0.01 '*' 0.05 '.' 0.1 ' ' 1
   
   Residual standard error: 1.792 on 90 degrees of freedom
     (2 observations deleted due to missingness)
   Multiple R-squared:  0.7985, Adjusted R-squared:  0.7851 
   F-statistic: 59.44 on 6 and 90 DF,  p-value: < 2.2e-16
anova(LM.dN)
   Analysis of Variance Table
   
   Response: d15NGly
             Df Sum Sq Mean Sq F value    Pr(>F)    
   TPAla      1 851.75  851.75 265.178 < 2.2e-16 ***
   Location   5 293.86   58.77  18.298 1.715e-12 ***
   Residuals 90 289.08    3.21                      
   ---
   Signif. codes:  0 '***' 0.001 '**' 0.01 '*' 0.05 '.' 0.1 ' ' 1
coef.2 <- c(
  LM.dN$coefficients[1]
)
p4 <- anova(LM.dN)[1,5] 
R4 <- summary(LM.dN)$adj.r.squared

ggplot(data = data.reg.phenorm, aes(x = (TPAla-1), y = d15NGly))+
  geom_point(aes(color=Location))+
  geom_smooth(method=lm, aes(y = predict(LM.dN, data.reg.phenorm), color=Location), lty=1, se=TRUE)+
  xlab(expression(TP[Ala-Phe]-1))+
  ylab(expression(delta^{15}*N[Gly-Phe]*" (\u2030)"))+
  theme_light()+
  facet_wrap(~ Location)
   `geom_smooth()` using formula = 'y ~ x'
   Warning: Removed 2 rows containing non-finite outside the scale range
   (`stat_smooth()`).
   Warning: Removed 2 rows containing missing values or values outside the scale range
   (`geom_point()`).
```

```
DN.reg <- data.frame(
  "TP(Glx-Phe)" = coef.1,
  "TP(Ala-Phe)" = coef.2,
  row.names = "d15NGly"
)

DN.reg
           TP.Glx.Phe. TP.Ala.Phe.
   d15NGly    3.063497    2.166534
```

1. We tested to see if location had a significant effect on the slope of the regression. The P values associated with this test were 0.6642867 and 0.6963739 for TP(Glx-Phe) and TP(Ala-Phe), respectively. This indicates that location DOES NOT have a significant effect on the slope of the regression.
2. Removing the interaction with location, we tested to see if the slope is significant. The P value associated with these tests were 1.1820413^{-29} and 1.4332731^{-28}. This indicates that the slope of the regression IS significant.
3. The \(R^2\) values were 0.8056241 and 0.7850749, respectively.

## Regression for Thr

```
noquote("Checking to see if location affects the slope of the regression...")
   [1] Checking to see if location affects the slope of the regression...

noquote("Regressing d15N(Thr-Phe) against TP(Glx-Phe)")
   [1] Regressing d15N(Thr-Phe) against TP(Glx-Phe)
LM.dN = lm(d15NThr ~ (TPGlx-1) * Location, data = data.reg.phenorm)
summary(LM.dN)
   
   Call:
   lm(formula = d15NThr ~ (TPGlx - 1) * Location, data = data.reg.phenorm)
   
   Residuals:
       Min      1Q  Median      3Q     Max 
   -8.8788 -2.2608  0.1168  1.7354  9.8700 
   
   Coefficients:
                         Estimate Std. Error t value Pr(>|t|)    
   TPGlx                  -6.1754     1.4870  -4.153 7.78e-05 ***
   Location5 N            10.3444     3.6805   2.811  0.00614 ** 
   Location8 N             2.9000     3.8509   0.753  0.45349    
   LocationALOHA S         7.9807     3.0348   2.630  0.01014 *  
   LocationALOHA W         5.2682     2.6028   2.024  0.04611 *  
   LocationOSP             4.4753     1.7802   2.514  0.01382 *  
   TPGlx:Location8 N       2.8398     2.1324   1.332  0.18651    
   TPGlx:LocationALOHA S  -1.4628     2.0578  -0.711  0.47911    
   TPGlx:LocationALOHA W   0.5011     1.9070   0.263  0.79335    
   TPGlx:LocationOSP      -0.7127     1.7081  -0.417  0.67755    
   ---
   Signif. codes:  0 '***' 0.001 '**' 0.01 '*' 0.05 '.' 0.1 ' ' 1
   
   Residual standard error: 3.391 on 85 degrees of freedom
     (3 observations deleted due to missingness)
   Multiple R-squared:  0.8806, Adjusted R-squared:  0.8665 
   F-statistic: 62.67 on 10 and 85 DF,  p-value: < 2.2e-16
anova(LM.dN)
   Analysis of Variance Table
   
   Response: d15NThr
                  Df Sum Sq Mean Sq  F value    Pr(>F)    
   TPGlx           1 6350.9  6350.9 552.3608 < 2.2e-16 ***
   Location        5  793.1   158.6  13.7962 7.344e-10 ***
   TPGlx:Location  4   62.1    15.5   1.3494    0.2584    
   Residuals      85  977.3    11.5                       
   ---
   Signif. codes:  0 '***' 0.001 '**' 0.01 '*' 0.05 '.' 0.1 ' ' 1
coef.1 <- c(
  LM.dN$coefficients[1],
  LM.dN$coefficients[1] + LM.dN$coefficients[7],
  LM.dN$coefficients[1] + LM.dN$coefficients[8],
  LM.dN$coefficients[1] + LM.dN$coefficients[9],
  LM.dN$coefficients[1] + LM.dN$coefficients[10]
)
p1 <- anova(LM.dN)[3,5] 
R1 <- summary(LM.dN)$adj.r.squared

noquote("Regressing d15N(Thr-Phe) against TP(Ala-Phe)")
   [1] Regressing d15N(Thr-Phe) against TP(Ala-Phe)
LM.dN = lm(d15NThr ~ (TPAla-1) * Location, data = data.reg.phenorm)
summary(LM.dN)
   
   Call:
   lm(formula = d15NThr ~ (TPAla - 1) * Location, data = data.reg.phenorm)
   
   Residuals:
       Min      1Q  Median      3Q     Max 
   -8.1569 -1.8795  0.0157  1.9667 10.1006 
   
   Coefficients:
                          Estimate Std. Error t value Pr(>|t|)    
   TPAla                 -4.540128   1.221266  -3.718 0.000357 ***
   Location5 N            8.062466   3.495994   2.306 0.023505 *  
   Location8 N            1.862793   3.723903   0.500 0.618194    
   LocationALOHA S        5.507307   2.512241   2.192 0.031067 *  
   LocationALOHA W        4.432290   2.402377   1.845 0.068485 .  
   LocationOSP            1.846835   1.521339   1.214 0.228089    
   TPAla:Location8 N      2.132740   1.724609   1.237 0.219583    
   TPAla:LocationALOHA S -0.569000   1.545763  -0.368 0.713701    
   TPAla:LocationALOHA W  0.226638   1.511909   0.150 0.881193    
   TPAla:LocationOSP      0.003161   1.347665   0.002 0.998134    
   ---
   Signif. codes:  0 '***' 0.001 '**' 0.01 '*' 0.05 '.' 0.1 ' ' 1
   
   Residual standard error: 3.47 on 86 degrees of freedom
     (2 observations deleted due to missingness)
   Multiple R-squared:  0.8735, Adjusted R-squared:  0.8587 
   F-statistic: 59.36 on 10 and 86 DF,  p-value: < 2.2e-16
anova(LM.dN)
   Analysis of Variance Table
   
   Response: d15NThr
                  Df Sum Sq Mean Sq  F value    Pr(>F)    
   TPAla           1 6480.9  6480.9 538.2132 < 2.2e-16 ***
   Location        5  627.2   125.4  10.4180 7.777e-08 ***
   TPAla:Location  4   39.9    10.0   0.8287    0.5105    
   Residuals      86 1035.6    12.0                       
   ---
   Signif. codes:  0 '***' 0.001 '**' 0.01 '*' 0.05 '.' 0.1 ' ' 1
coef.2 <- c(
  LM.dN$coefficients[1],
  LM.dN$coefficients[1] + LM.dN$coefficients[7],
  LM.dN$coefficients[1] + LM.dN$coefficients[8],
  LM.dN$coefficients[1] + LM.dN$coefficients[9],
  LM.dN$coefficients[1] + LM.dN$coefficients[10]
)
p2 <- anova(LM.dN)[3,5] 
R2 <- summary(LM.dN)$adj.r.squared


noquote("Now re-running the regression without location interaction")
   [1] Now re-running the regression without location interaction

noquote("Regressing d15N(Thr-Phe) against TP(Glx-Phe)")
   [1] Regressing d15N(Thr-Phe) against TP(Glx-Phe)
LM.dN = lm(d15NThr ~ (TPGlx-1) + Location, data = data.reg.phenorm)
summary(LM.dN)
   
   Call:
   lm(formula = d15NThr ~ (TPGlx - 1) + Location, data = data.reg.phenorm)
   
   Residuals:
       Min      1Q  Median      3Q     Max 
   -8.9121 -2.2896  0.0884  1.8535  9.8858 
   
   Coefficients:
                   Estimate Std. Error t value Pr(>|t|)    
   TPGlx            -6.2226     0.5393 -11.538  < 2e-16 ***
   Location5 N      10.4562     1.6728   6.251 1.38e-08 ***
   Location8 N       9.9350     1.6432   6.046 3.41e-08 ***
   LocationALOHA S   5.0735     1.3834   3.667 0.000417 ***
   LocationALOHA W   6.4091     1.3688   4.682 1.01e-05 ***
   LocationOSP       3.1365     1.2218   2.567 0.011924 *  
   ---
   Signif. codes:  0 '***' 0.001 '**' 0.01 '*' 0.05 '.' 0.1 ' ' 1
   
   Residual standard error: 3.417 on 89 degrees of freedom
     (3 observations deleted due to missingness)
   Multiple R-squared:  0.873,  Adjusted R-squared:  0.8644 
   F-statistic:   102 on 6 and 89 DF,  p-value: < 2.2e-16
anova(LM.dN)
   Analysis of Variance Table
   
   Response: d15NThr
             Df Sum Sq Mean Sq F value  Pr(>F)    
   TPGlx      1 6350.9  6350.9 543.822 < 2e-16 ***
   Location   5  793.1   158.6  13.583 7.6e-10 ***
   Residuals 89 1039.4    11.7                    
   ---
   Signif. codes:  0 '***' 0.001 '**' 0.01 '*' 0.05 '.' 0.1 ' ' 1
coef.1 <- c(
  LM.dN$coefficients[1]
)
p3 <- anova(LM.dN)[1,5] 
R3 <- summary(LM.dN)$adj.r.squared

ggplot(data = data.reg.phenorm, aes(x = (TPGlx-1), y = d15NThr))+
  geom_point(aes(color=Location))+
  geom_smooth(method=lm, aes(y = predict(LM.dN, data.reg.phenorm), color=Location), lty=1, se=TRUE)+
  xlab(expression(TP[Glx-Phe]-1))+
  ylab(expression(delta^{15}*N[Thr-Phe]*" (\u2030)"))+
  theme_light()+
  facet_wrap(~ Location)
   `geom_smooth()` using formula = 'y ~ x'
   Warning: Removed 3 rows containing non-finite outside the scale range
   (`stat_smooth()`).
   Warning: Removed 3 rows containing missing values or values outside the scale range
   (`geom_point()`).
```

```
noquote("Regressing d15N(Thr-Phe) against TP(Ala-Phe)")
   [1] Regressing d15N(Thr-Phe) against TP(Ala-Phe)
LM.dN = lm(d15NThr ~ (TPAla-1) + Location, data = data.reg.phenorm)
summary(LM.dN)
   
   Call:
   lm(formula = d15NThr ~ (TPAla - 1) + Location, data = data.reg.phenorm)
   
   Residuals:
       Min      1Q  Median      3Q     Max 
   -8.1941 -2.0408  0.0211  1.8550 10.0187 
   
   Coefficients:
                   Estimate Std. Error t value Pr(>|t|)    
   TPAla            -4.3783     0.3821 -11.459  < 2e-16 ***
   Location5 N       7.6227     1.5078   5.055 2.24e-06 ***
   Location8 N       7.6681     1.5041   5.098 1.89e-06 ***
   LocationALOHA S   3.6754     1.2575   2.923 0.004388 ** 
   LocationALOHA W   4.5972     1.2543   3.665 0.000417 ***
   LocationOSP       1.4542     1.1033   1.318 0.190826    
   ---
   Signif. codes:  0 '***' 0.001 '**' 0.01 '*' 0.05 '.' 0.1 ' ' 1
   
   Residual standard error: 3.457 on 90 degrees of freedom
     (2 observations deleted due to missingness)
   Multiple R-squared:  0.8686, Adjusted R-squared:  0.8598 
   F-statistic: 99.14 on 6 and 90 DF,  p-value: < 2.2e-16
anova(LM.dN)
   Analysis of Variance Table
   
   Response: d15NThr
             Df Sum Sq Mean Sq F value    Pr(>F)    
   TPAla      1 6480.9  6480.9 542.341 < 2.2e-16 ***
   Location   5  627.2   125.4  10.498 5.864e-08 ***
   Residuals 90 1075.5    11.9                      
   ---
   Signif. codes:  0 '***' 0.001 '**' 0.01 '*' 0.05 '.' 0.1 ' ' 1
coef.2 <- c(
  LM.dN$coefficients[1]
)
p4 <- anova(LM.dN)[1,5] 
R4 <- summary(LM.dN)$adj.r.squared

ggplot(data = data.reg.phenorm, aes(x = (TPAla-1), y = d15NThr))+
  geom_point(aes(color=Location))+
  geom_smooth(method=lm, aes(y = predict(LM.dN, data.reg.phenorm), color=Location), lty=1, se=TRUE)+
  xlab(expression(TP[Ala-Phe]-1))+
  ylab(expression(delta^{15}*N[Thr-Phe]*" (\u2030)"))+
  theme_light()+
  facet_wrap(~ Location)
   `geom_smooth()` using formula = 'y ~ x'
   Warning: Removed 2 rows containing non-finite outside the scale range
   (`stat_smooth()`).
   Warning: Removed 2 rows containing missing values or values outside the scale range
   (`geom_point()`).
```

```
DN.reg <- data.frame(
  "TP(Glx-Phe)" = coef.1,
  "TP(Ala-Phe)" = coef.2,
  row.names = "d15NThr"
)

DN.reg
           TP.Glx.Phe. TP.Ala.Phe.
   d15NThr   -6.222591   -4.378343
```

1. We tested to see if location had a significant effect on the slope of the regression. The P values associated with this test were 0.2584458 and 0.5104952 for TP(Glx-Phe) and TP(Ala-Phe), respectively. This indicates that location DOES NOT have a significant effect on the slope of the regression.
2. Removing the interaction with location, we tested to see if the slope is significant. The P value associated with these tests were 1.1194251^{-39} and 7.1493387^{-40}. This indicates that the slope of the regression IS significant.
3. The \(R^2\) values were 0.8644283 and 0.8598193, respectively.

## Regression for Ser

```
noquote("Checking to see if location affects the slope of the regression...")
   [1] Checking to see if location affects the slope of the regression...

noquote("Regressing d15N(Ser-Phe) against TP(Glx-Phe)")
   [1] Regressing d15N(Ser-Phe) against TP(Glx-Phe)
LM.dN = lm(d15NSer ~ (TPGlx-1) * Location, data = data.reg.phenorm)
summary(LM.dN)
   
   Call:
   lm(formula = d15NSer ~ (TPGlx - 1) * Location, data = data.reg.phenorm)
   
   Residuals:
       Min      1Q  Median      3Q     Max 
   -6.8745 -0.7597 -0.0139  1.0356  4.9782 
   
   Coefficients:
                         Estimate Std. Error t value Pr(>|t|)    
   TPGlx                   3.6194     0.7069   5.120 1.87e-06 ***
   Location5 N            -5.1560     1.7495  -2.947 0.004139 ** 
   Location8 N            -6.5774     1.8305  -3.593 0.000546 ***
   LocationALOHA S        -6.3931     1.4426  -4.432 2.77e-05 ***
   LocationALOHA W        -7.4024     1.2373  -5.983 5.03e-08 ***
   LocationOSP           -10.5330     0.8462 -12.447  < 2e-16 ***
   TPGlx:Location8 N       0.6175     1.0136   0.609 0.544012    
   TPGlx:LocationALOHA S   1.3857     0.9782   1.417 0.160239    
   TPGlx:LocationALOHA W   1.6752     0.9065   1.848 0.068080 .  
   TPGlx:LocationOSP       1.6707     0.8119   2.058 0.042688 *  
   ---
   Signif. codes:  0 '***' 0.001 '**' 0.01 '*' 0.05 '.' 0.1 ' ' 1
   
   Residual standard error: 1.612 on 85 degrees of freedom
     (3 observations deleted due to missingness)
   Multiple R-squared:  0.8891, Adjusted R-squared:  0.8761 
   F-statistic: 68.16 on 10 and 85 DF,  p-value: < 2.2e-16
anova(LM.dN)
   Analysis of Variance Table
   
   Response: d15NSer
                  Df Sum Sq Mean Sq  F value Pr(>F)    
   TPGlx           1 964.20  964.20 371.1331 <2e-16 ***
   Location        5 792.16  158.43  60.9822 <2e-16 ***
   TPGlx:Location  4  14.49    3.62   1.3939  0.243    
   Residuals      85 220.83    2.60                    
   ---
   Signif. codes:  0 '***' 0.001 '**' 0.01 '*' 0.05 '.' 0.1 ' ' 1
coef.1 <- c(
  LM.dN$coefficients[1],
  LM.dN$coefficients[1] + LM.dN$coefficients[7],
  LM.dN$coefficients[1] + LM.dN$coefficients[8],
  LM.dN$coefficients[1] + LM.dN$coefficients[9],
  LM.dN$coefficients[1] + LM.dN$coefficients[10]
)
p1 <- anova(LM.dN)[3,5] 
R1 <- summary(LM.dN)$adj.r.squared

noquote("Regressing d15N(Ser-Phe) against TP(Ala-Phe)")
   [1] Regressing d15N(Ser-Phe) against TP(Ala-Phe)
LM.dN = lm(d15NSer ~ (TPAla-1) * Location, data = data.reg.phenorm)
summary(LM.dN)
   
   Call:
   lm(formula = d15NSer ~ (TPAla - 1) * Location, data = data.reg.phenorm)
   
   Residuals:
       Min      1Q  Median      3Q     Max 
   -7.3183 -0.8721  0.1413  0.9634  4.6952 
   
   Coefficients:
                         Estimate Std. Error t value Pr(>|t|)    
   TPAla                   2.9038     0.6249   4.647 1.20e-05 ***
   Location5 N            -4.4785     1.7887  -2.504  0.01418 *  
   Location8 N            -5.8107     1.9053  -3.050  0.00304 ** 
   LocationALOHA S        -5.3091     1.2854  -4.130 8.37e-05 ***
   LocationALOHA W        -5.5821     1.2292  -4.541 1.81e-05 ***
   LocationOSP            -8.4565     0.7784 -10.864  < 2e-16 ***
   TPAla:Location8 N       0.3411     0.8824   0.387  0.70003    
   TPAla:LocationALOHA S   0.6325     0.7909   0.800  0.42604    
   TPAla:LocationALOHA W   0.7120     0.7736   0.920  0.35994    
   TPAla:LocationOSP       0.5572     0.6895   0.808  0.42125    
   ---
   Signif. codes:  0 '***' 0.001 '**' 0.01 '*' 0.05 '.' 0.1 ' ' 1
   
   Residual standard error: 1.775 on 86 degrees of freedom
     (2 observations deleted due to missingness)
   Multiple R-squared:  0.8642, Adjusted R-squared:  0.8484 
   F-statistic: 54.72 on 10 and 86 DF,  p-value: < 2.2e-16
anova(LM.dN)
   Analysis of Variance Table
   
   Response: d15NSer
                  Df  Sum Sq Mean Sq  F value Pr(>F)    
   TPAla           1 1001.64 1001.64 317.7478 <2e-16 ***
   Location        5  719.96  143.99  45.6784 <2e-16 ***
   TPAla:Location  4    3.22    0.81   0.2556 0.9055    
   Residuals      86  271.10    3.15                    
   ---
   Signif. codes:  0 '***' 0.001 '**' 0.01 '*' 0.05 '.' 0.1 ' ' 1
coef.2 <- c(
  LM.dN$coefficients[1],
  LM.dN$coefficients[1] + LM.dN$coefficients[7],
  LM.dN$coefficients[1] + LM.dN$coefficients[8],
  LM.dN$coefficients[1] + LM.dN$coefficients[9],
  LM.dN$coefficients[1] + LM.dN$coefficients[10]
)
p2 <- anova(LM.dN)[3,5] 
R2 <- summary(LM.dN)$adj.r.squared


noquote("Now re-running the regression without location interaction")
   [1] Now re-running the regression without location interaction

noquote("Regressing d15N(Ser-Phe) against TP(Glx-Phe)")
   [1] Regressing d15N(Ser-Phe) against TP(Glx-Phe)
LM.dN = lm(d15NSer ~ (TPGlx-1) + Location, data = data.reg.phenorm)
summary(LM.dN)
   
   Call:
   lm(formula = d15NSer ~ (TPGlx - 1) + Location, data = data.reg.phenorm)
   
   Residuals:
       Min      1Q  Median      3Q     Max 
   -6.6508 -0.8558  0.0053  1.0658  5.2484 
   
   Coefficients:
                   Estimate Std. Error t value Pr(>|t|)    
   TPGlx             4.9052     0.2566  19.115  < 2e-16 ***
   Location5 N      -8.2004     0.7960 -10.302  < 2e-16 ***
   Location8 N      -8.2058     0.7819 -10.495  < 2e-16 ***
   LocationALOHA S  -6.1879     0.6582  -9.401 5.56e-15 ***
   LocationALOHA W  -6.5923     0.6513 -10.122  < 2e-16 ***
   LocationOSP      -9.7587     0.5814 -16.786  < 2e-16 ***
   ---
   Signif. codes:  0 '***' 0.001 '**' 0.01 '*' 0.05 '.' 0.1 ' ' 1
   
   Residual standard error: 1.626 on 89 degrees of freedom
     (3 observations deleted due to missingness)
   Multiple R-squared:  0.8819, Adjusted R-squared:  0.8739 
   F-statistic: 110.7 on 6 and 89 DF,  p-value: < 2.2e-16
anova(LM.dN)
   Analysis of Variance Table
   
   Response: d15NSer
             Df Sum Sq Mean Sq F value    Pr(>F)    
   TPGlx      1 964.20  964.20 364.676 < 2.2e-16 ***
   Location   5 792.16  158.43  59.921 < 2.2e-16 ***
   Residuals 89 235.32    2.64                      
   ---
   Signif. codes:  0 '***' 0.001 '**' 0.01 '*' 0.05 '.' 0.1 ' ' 1
coef.1 <- c(
  LM.dN$coefficients[1]
)
p3 <- anova(LM.dN)[1,5] 
R3 <- summary(LM.dN)$adj.r.squared

ggplot(data = data.reg.phenorm, aes(x = (TPGlx-1), y = d15NSer))+
  geom_point(aes(color=Location))+
  geom_smooth(method=lm, aes(y = predict(LM.dN, data.reg.phenorm), color=Location), lty=1, se=TRUE)+
  xlab(expression(TP[Glx-Phe]-1))+
  ylab(expression(delta^{15}*N[Ser-Phe]*" (\u2030)"))+
  theme_light()+
  facet_wrap(~ Location)
   `geom_smooth()` using formula = 'y ~ x'
   Warning: Removed 3 rows containing non-finite outside the scale range
   (`stat_smooth()`).
   Warning: Removed 3 rows containing missing values or values outside the scale range
   (`geom_point()`).
```

```
noquote("Regressing d15N(Ser-Phe) against TP(Ala-Phe)")
   [1] Regressing d15N(Ser-Phe) against TP(Ala-Phe)
LM.dN = lm(d15NSer ~ (TPAla-1) + Location, data = data.reg.phenorm)
summary(LM.dN)
   
   Call:
   lm(formula = d15NSer ~ (TPAla - 1) + Location, data = data.reg.phenorm)
   
   Residuals:
       Min      1Q  Median      3Q     Max 
   -7.2823 -1.0678  0.1351  1.0519  4.7361 
   
   Coefficients:
                   Estimate Std. Error t value Pr(>|t|)    
   TPAla             3.4256     0.1930  17.751  < 2e-16 ***
   Location5 N      -5.8968     0.7615  -7.744 1.37e-11 ***
   Location8 N      -6.3429     0.7597  -8.350 7.72e-13 ***
   LocationALOHA S  -5.0315     0.6351  -7.922 5.89e-12 ***
   LocationALOHA W  -5.0985     0.6335  -8.049 3.24e-12 ***
   LocationOSP      -8.3688     0.5572 -15.019  < 2e-16 ***
   ---
   Signif. codes:  0 '***' 0.001 '**' 0.01 '*' 0.05 '.' 0.1 ' ' 1
   
   Residual standard error: 1.746 on 90 degrees of freedom
     (2 observations deleted due to missingness)
   Multiple R-squared:  0.8626, Adjusted R-squared:  0.8534 
   F-statistic: 94.14 on 6 and 90 DF,  p-value: < 2.2e-16
anova(LM.dN)
   Analysis of Variance Table
   
   Response: d15NSer
             Df  Sum Sq Mean Sq F value    Pr(>F)    
   TPAla      1 1001.64 1001.64 328.620 < 2.2e-16 ***
   Location   5  719.96  143.99  47.241 < 2.2e-16 ***
   Residuals 90  274.32    3.05                      
   ---
   Signif. codes:  0 '***' 0.001 '**' 0.01 '*' 0.05 '.' 0.1 ' ' 1
coef.2 <- c(
  LM.dN$coefficients[1]
)
p4 <- anova(LM.dN)[1,5] 
R4 <- summary(LM.dN)$adj.r.squared

ggplot(data = data.reg.phenorm, aes(x = (TPAla-1), y = d15NSer))+
  geom_point(aes(color=Location))+
  geom_smooth(method=lm, aes(y = predict(LM.dN, data.reg.phenorm), color=Location), lty=1, se=TRUE)+
  xlab(expression(TP[Ala-Phe]-1))+
  ylab(expression(delta^{15}*N[Ser-Phe]*" (\u2030)"))+
  theme_light()+
  facet_wrap(~ Location)
   `geom_smooth()` using formula = 'y ~ x'
   Warning: Removed 2 rows containing non-finite outside the scale range
   (`stat_smooth()`).
   Warning: Removed 2 rows containing missing values or values outside the scale range
   (`geom_point()`).
```

```
DN.reg <- data.frame(
  "TP(Glx-Phe)" = coef.1,
  "TP(Ala-Phe)" = coef.2,
  row.names = "d15NSer"
)

DN.reg
           TP.Glx.Phe. TP.Ala.Phe.
   d15NSer    4.905207    3.425631
```

1. We tested to see if location had a significant effect on the slope of the regression. The P values associated with this test were 0.242973 and 0.9055156 for TP(Glx-Phe) and TP(Ala-Phe), respectively. This indicates that location DOES NOT have a significant effect on the slope of the regression.
2. Removing the interaction with location, we tested to see if the slope is significant. The P value associated with these tests were 3.1255825^{-33} and 8.5880305^{-32}. This indicates that the slope of the regression IS significant.
3. The \(R^2\) values were 0.8738854 and 0.8533964, respectively.

## Regression for Val

```
noquote("Checking to see if location affects the slope of the regression...")
   [1] Checking to see if location affects the slope of the regression...

noquote("Regressing d15N(Val-Phe) against TP(Glx-Phe)")
   [1] Regressing d15N(Val-Phe) against TP(Glx-Phe)
LM.dN = lm(d15NVal ~ (TPGlx-1) * Location, data = data.reg.phenorm)
summary(LM.dN)
   
   Call:
   lm(formula = d15NVal ~ (TPGlx - 1) * Location, data = data.reg.phenorm)
   
   Residuals:
        Min       1Q   Median       3Q      Max 
   -11.2595  -0.9897  -0.2617   1.2510   4.2787 
   
   Coefficients:
                         Estimate Std. Error t value Pr(>|t|)    
   TPGlx                  5.13243    0.90570   5.667 1.95e-07 ***
   Location5 N           -1.19338    2.24170  -0.532 0.595870    
   Location8 N           -0.42417    2.34546  -0.181 0.856917    
   LocationALOHA S       -3.90691    1.84842  -2.114 0.037476 *  
   LocationALOHA W       -5.91604    1.58531  -3.732 0.000343 ***
   LocationOSP           -3.16673    1.08427  -2.921 0.004472 ** 
   TPGlx:Location8 N      0.02091    1.29880   0.016 0.987191    
   TPGlx:LocationALOHA S  2.56387    1.25334   2.046 0.043884 *  
   TPGlx:LocationALOHA W  2.94244    1.16147   2.533 0.013133 *  
   TPGlx:LocationOSP      0.48406    1.04033   0.465 0.642907    
   ---
   Signif. codes:  0 '***' 0.001 '**' 0.01 '*' 0.05 '.' 0.1 ' ' 1
   
   Residual standard error: 2.065 on 85 degrees of freedom
     (3 observations deleted due to missingness)
   Multiple R-squared:   0.97,  Adjusted R-squared:  0.9665 
   F-statistic: 274.7 on 10 and 85 DF,  p-value: < 2.2e-16
anova(LM.dN)
   Analysis of Variance Table
   
   Response: d15NVal
                  Df  Sum Sq Mean Sq  F value    Pr(>F)    
   TPGlx           1 11400.9 11400.9 2672.939 < 2.2e-16 ***
   Location        5   257.9    51.6   12.095 7.393e-09 ***
   TPGlx:Location  4    57.6    14.4    3.379    0.0129 *  
   Residuals      85   362.6     4.3                       
   ---
   Signif. codes:  0 '***' 0.001 '**' 0.01 '*' 0.05 '.' 0.1 ' ' 1
coef.1 <- c(
  LM.dN$coefficients[1],
  LM.dN$coefficients[1] + LM.dN$coefficients[7],
  LM.dN$coefficients[1] + LM.dN$coefficients[8],
  LM.dN$coefficients[1] + LM.dN$coefficients[9],
  LM.dN$coefficients[1] + LM.dN$coefficients[10]
)
p1 <- anova(LM.dN)[3,5] 
R1 <- summary(LM.dN)$adj.r.squared

noquote("Regressing d15N(Val-Phe) against TP(Ala-Phe)")
   [1] Regressing d15N(Val-Phe) against TP(Ala-Phe)
LM.dN = lm(d15NVal ~ (TPAla-1) * Location, data = data.reg.phenorm)
summary(LM.dN)
   
   Call:
   lm(formula = d15NVal ~ (TPAla - 1) * Location, data = data.reg.phenorm)
   
   Residuals:
        Min       1Q   Median       3Q      Max 
   -11.1582  -0.9984   0.1608   1.2983   4.2889 
   
   Coefficients:
                         Estimate Std. Error t value Pr(>|t|)    
   TPAla                  4.00792    0.74369   5.389  6.1e-07 ***
   Location5 N            0.06562    2.12890   0.031  0.97548    
   Location8 N            0.60915    2.26769   0.269  0.78886    
   LocationALOHA S       -2.95609    1.52984  -1.932  0.05662 .  
   LocationALOHA W       -4.35070    1.46294  -2.974  0.00381 ** 
   LocationOSP           -0.68419    0.92643  -0.739  0.46221    
   TPAla:Location8 N     -0.09536    1.05021  -0.091  0.92786    
   TPAla:LocationALOHA S  1.70854    0.94130   1.815  0.07300 .  
   TPAla:LocationALOHA W  1.98272    0.92068   2.154  0.03408 *  
   TPAla:LocationOSP     -0.44559    0.82067  -0.543  0.58856    
   ---
   Signif. codes:  0 '***' 0.001 '**' 0.01 '*' 0.05 '.' 0.1 ' ' 1
   
   Residual standard error: 2.113 on 86 degrees of freedom
     (2 observations deleted due to missingness)
   Multiple R-squared:  0.9682, Adjusted R-squared:  0.9645 
   F-statistic:   262 on 10 and 86 DF,  p-value: < 2.2e-16
anova(LM.dN)
   Analysis of Variance Table
   
   Response: d15NVal
                  Df  Sum Sq Mean Sq   F value    Pr(>F)    
   TPAla           1 11435.2 11435.2 2560.9141 < 2.2e-16 ***
   Location        5   175.2    35.0    7.8467 4.005e-06 ***
   TPAla:Location  4    90.6    22.7    5.0742  0.001017 ** 
   Residuals      86   384.0     4.5                        
   ---
   Signif. codes:  0 '***' 0.001 '**' 0.01 '*' 0.05 '.' 0.1 ' ' 1
coef.2 <- c(
  LM.dN$coefficients[1],
  LM.dN$coefficients[1] + LM.dN$coefficients[7],
  LM.dN$coefficients[1] + LM.dN$coefficients[8],
  LM.dN$coefficients[1] + LM.dN$coefficients[9],
  LM.dN$coefficients[1] + LM.dN$coefficients[10]
)
p2 <- anova(LM.dN)[3,5] 
R2 <- summary(LM.dN)$adj.r.squared


noquote("Now re-running the regression without location interaction")
   [1] Now re-running the regression without location interaction

noquote("Regressing d15N(Val-Phe) against TP(Glx-Phe)")
   [1] Regressing d15N(Val-Phe) against TP(Glx-Phe)
LM.dN = lm(d15NVal ~ (TPGlx-1) + Location, data = data.reg.phenorm)
summary(LM.dN)
   
   Call:
   lm(formula = d15NVal ~ (TPGlx - 1) + Location, data = data.reg.phenorm)
   
   Residuals:
        Min       1Q   Median       3Q      Max 
   -11.8602  -0.8806  -0.0311   1.2159   4.8615 
   
   Coefficients:
                   Estimate Std. Error t value Pr(>|t|)    
   TPGlx             6.2853     0.3429  18.329  < 2e-16 ***
   Location5 N      -3.9231     1.0636  -3.688 0.000388 ***
   Location8 N      -3.1825     1.0448  -3.046 0.003051 ** 
   LocationALOHA S  -1.0091     0.8796  -1.147 0.254343    
   LocationALOHA W  -2.1928     0.8703  -2.519 0.013540 *  
   LocationOSP      -4.5123     0.7769  -5.808 9.62e-08 ***
   ---
   Signif. codes:  0 '***' 0.001 '**' 0.01 '*' 0.05 '.' 0.1 ' ' 1
   
   Residual standard error: 2.173 on 89 degrees of freedom
     (3 observations deleted due to missingness)
   Multiple R-squared:  0.9652, Adjusted R-squared:  0.9629 
   F-statistic: 411.6 on 6 and 89 DF,  p-value: < 2.2e-16
anova(LM.dN)
   Analysis of Variance Table
   
   Response: d15NVal
             Df  Sum Sq Mean Sq  F value    Pr(>F)    
   TPGlx      1 11400.9 11400.9 2414.753 < 2.2e-16 ***
   Location   5   257.9    51.6   10.927 3.239e-08 ***
   Residuals 89   420.2     4.7                       
   ---
   Signif. codes:  0 '***' 0.001 '**' 0.01 '*' 0.05 '.' 0.1 ' ' 1
coef.1 <- c(
  LM.dN$coefficients[1]
)
p3 <- anova(LM.dN)[1,5] 
R3 <- summary(LM.dN)$adj.r.squared

ggplot(data = data.reg.phenorm, aes(x = (TPGlx-1), y = d15NVal))+
  geom_point(aes(color=Location))+
  geom_smooth(method=lm, aes(y = predict(LM.dN, data.reg.phenorm), color=Location), lty=1, se=TRUE)+
  xlab(expression(TP[Glx-Phe]-1))+
  ylab(expression(delta^{15}*N[Val-Phe]*" (\u2030)"))+
  theme_light()+
  facet_wrap(~ Location)
   `geom_smooth()` using formula = 'y ~ x'
   Warning: Removed 3 rows containing non-finite outside the scale range
   (`stat_smooth()`).
   Warning: Removed 3 rows containing missing values or values outside the scale range
   (`geom_point()`).
```

```
noquote("Regressing d15N(Val-Phe) against TP(Ala-Phe)")
   [1] Regressing d15N(Val-Phe) against TP(Ala-Phe)
LM.dN = lm(d15NVal ~ (TPAla-1) + Location, data = data.reg.phenorm)
summary(LM.dN)
   
   Call:
   lm(formula = d15NVal ~ (TPAla - 1) + Location, data = data.reg.phenorm)
   
   Residuals:
       Min      1Q  Median      3Q     Max 
   -12.381  -1.113   0.361   1.493   3.761 
   
   Coefficients:
                   Estimate Std. Error t value Pr(>|t|)    
   TPAla             4.4437     0.2538  17.506  < 2e-16 ***
   Location5 N      -1.1189     1.0017  -1.117 0.266976    
   Location8 N      -0.9554     0.9992  -0.956 0.341588    
   LocationALOHA S   0.2344     0.8354   0.281 0.779668    
   LocationALOHA W  -0.4167     0.8332  -0.500 0.618251    
   LocationOSP      -2.8656     0.7330  -3.910 0.000179 ***
   ---
   Signif. codes:  0 '***' 0.001 '**' 0.01 '*' 0.05 '.' 0.1 ' ' 1
   
   Residual standard error: 2.296 on 90 degrees of freedom
     (2 observations deleted due to missingness)
   Multiple R-squared:  0.9607, Adjusted R-squared:  0.9581 
   F-statistic: 366.9 on 6 and 90 DF,  p-value: < 2.2e-16
anova(LM.dN)
   Analysis of Variance Table
   
   Response: d15NVal
             Df  Sum Sq Mean Sq   F value    Pr(>F)    
   TPAla      1 11435.2 11435.2 2168.2923 < 2.2e-16 ***
   Location   5   175.2    35.0    6.6437  2.62e-05 ***
   Residuals 90   474.6     5.3                        
   ---
   Signif. codes:  0 '***' 0.001 '**' 0.01 '*' 0.05 '.' 0.1 ' ' 1
coef.2 <- c(
  LM.dN$coefficients[1]
)
p4 <- anova(LM.dN)[1,5] 
R4 <- summary(LM.dN)$adj.r.squared

ggplot(data = data.reg.phenorm, aes(x = (TPAla-1), y = d15NVal))+
  geom_point(aes(color=Location))+
  geom_smooth(method=lm, aes(y = predict(LM.dN, data.reg.phenorm), color=Location), lty=1, se=TRUE)+
  xlab(expression(TP[Ala-Phe]-1))+
  ylab(expression(delta^{15}*N[Val-Phe]*" (\u2030)"))+
  theme_light()+
  facet_wrap(~ Location)
   `geom_smooth()` using formula = 'y ~ x'
   Warning: Removed 2 rows containing non-finite outside the scale range
   (`stat_smooth()`).
   Warning: Removed 2 rows containing missing values or values outside the scale range
   (`geom_point()`).
```

```
DN.reg <- data.frame(
  "TP(Glx-Phe)" = coef.1,
  "TP(Ala-Phe)" = coef.2,
  row.names = "d15NVal"
)

DN.reg
           TP.Glx.Phe. TP.Ala.Phe.
   d15NVal    6.285322    4.443728
```

1. We tested to see if location had a significant effect on the slope of the regression. The P values associated with this test were 0.0129049 and 0.0010171 for TP(Glx-Phe) and TP(Ala-Phe), respectively. This indicates that location DOES have a significant effect on the slope of the regression.
2. Removing the interaction with location, we tested to see if the slope is significant. The P value associated with these tests were 2.7812824^{-66} and 8.9753939^{-65}. This indicates that the slope of the regression IS significant.
3. The \(R^2\) values were 0.9628672 and 0.9581062, respectively.

## Regression for Leu

```
noquote("Checking to see if location affects the slope of the regression...")
   [1] Checking to see if location affects the slope of the regression...

noquote("Regressing d15N(Leu-Phe) against TP(Glx-Phe)")
   [1] Regressing d15N(Leu-Phe) against TP(Glx-Phe)
LM.dN = lm(d15NLeu ~ (TPGlx-1) * Location, data = data.reg.phenorm)
summary(LM.dN)
   
   Call:
   lm(formula = d15NLeu ~ (TPGlx - 1) * Location, data = data.reg.phenorm)
   
   Residuals:
       Min      1Q  Median      3Q     Max 
   -2.2031 -0.8051 -0.1955  0.5536  4.7472 
   
   Coefficients:
                         Estimate Std. Error t value Pr(>|t|)    
   TPGlx                  5.93802    0.58160  10.210  < 2e-16 ***
   Location5 N           -5.12116    1.43951  -3.558 0.000615 ***
   Location8 N           -4.79888    1.50614  -3.186 0.002017 ** 
   LocationALOHA S       -5.15101    1.18696  -4.340 3.91e-05 ***
   LocationALOHA W       -5.65729    1.01801  -5.557 3.09e-07 ***
   LocationOSP           -7.19149    0.69627 -10.329  < 2e-16 ***
   TPGlx:Location8 N      0.01601    0.83403   0.019 0.984731    
   TPGlx:LocationALOHA S  0.81249    0.80483   1.010 0.315595    
   TPGlx:LocationALOHA W  0.90798    0.74584   1.217 0.226823    
   TPGlx:LocationOSP      0.92201    0.66805   1.380 0.171160    
   ---
   Signif. codes:  0 '***' 0.001 '**' 0.01 '*' 0.05 '.' 0.1 ' ' 1
   
   Residual standard error: 1.326 on 85 degrees of freedom
     (3 observations deleted due to missingness)
   Multiple R-squared:  0.9817, Adjusted R-squared:  0.9795 
   F-statistic: 454.8 on 10 and 85 DF,  p-value: < 2.2e-16
anova(LM.dN)
   Analysis of Variance Table
   
   Response: d15NLeu
                  Df Sum Sq Mean Sq   F value Pr(>F)    
   TPGlx           1 7634.6  7634.6 4340.7012 <2e-16 ***
   Location        5  358.3    71.7   40.7479 <2e-16 ***
   TPGlx:Location  4    6.0     1.5    0.8592 0.4919    
   Residuals      85  149.5     1.8                     
   ---
   Signif. codes:  0 '***' 0.001 '**' 0.01 '*' 0.05 '.' 0.1 ' ' 1
coef.1 <- c(
  LM.dN$coefficients[1],
  LM.dN$coefficients[1] + LM.dN$coefficients[7],
  LM.dN$coefficients[1] + LM.dN$coefficients[8],
  LM.dN$coefficients[1] + LM.dN$coefficients[9],
  LM.dN$coefficients[1] + LM.dN$coefficients[10]
)
p1 <- anova(LM.dN)[3,5] 
R1 <- summary(LM.dN)$adj.r.squared

noquote("Regressing d15N(Leu-Phe) against TP(Ala-Phe)")
   [1] Regressing d15N(Leu-Phe) against TP(Ala-Phe)
LM.dN = lm(d15NLeu ~ (TPAla-1) * Location, data = data.reg.phenorm)
summary(LM.dN)
   
   Call:
   lm(formula = d15NLeu ~ (TPAla - 1) * Location, data = data.reg.phenorm)
   
   Residuals:
       Min      1Q  Median      3Q     Max 
   -3.2453 -0.7667  0.0354  0.9147  3.8558 
   
   Coefficients:
                         Estimate Std. Error t value Pr(>|t|)    
   TPAla                  4.77293    0.49154   9.710 1.76e-15 ***
   Location5 N           -4.03398    1.40707  -2.867  0.00521 ** 
   Location8 N           -4.22206    1.49880  -2.817  0.00601 ** 
   LocationALOHA S       -4.60291    1.01113  -4.552 1.73e-05 ***
   LocationALOHA W       -4.14977    0.96691  -4.292 4.63e-05 ***
   LocationOSP           -4.39916    0.61231  -7.185 2.29e-10 ***
   TPAla:Location8 N     -0.04298    0.69412  -0.062  0.95077    
   TPAla:LocationALOHA S  0.32788    0.62214   0.527  0.59954    
   TPAla:LocationALOHA W  0.23510    0.60851   0.386  0.70019    
   TPAla:LocationOSP     -0.32495    0.54241  -0.599  0.55069    
   ---
   Signif. codes:  0 '***' 0.001 '**' 0.01 '*' 0.05 '.' 0.1 ' ' 1
   
   Residual standard error: 1.397 on 86 degrees of freedom
     (2 observations deleted due to missingness)
   Multiple R-squared:  0.9794, Adjusted R-squared:  0.977 
   F-statistic: 409.2 on 10 and 86 DF,  p-value: < 2.2e-16
anova(LM.dN)
   Analysis of Variance Table
   
   Response: d15NLeu
                  Df Sum Sq Mean Sq   F value    Pr(>F)    
   TPAla           1 7720.7  7720.7 3958.0986 < 2.2e-16 ***
   Location        5  254.7    50.9   26.1164 5.984e-16 ***
   TPAla:Location  4    6.0     1.5    0.7651    0.5508    
   Residuals      86  167.8     2.0                        
   ---
   Signif. codes:  0 '***' 0.001 '**' 0.01 '*' 0.05 '.' 0.1 ' ' 1
coef.2 <- c(
  LM.dN$coefficients[1],
  LM.dN$coefficients[1] + LM.dN$coefficients[7],
  LM.dN$coefficients[1] + LM.dN$coefficients[8],
  LM.dN$coefficients[1] + LM.dN$coefficients[9],
  LM.dN$coefficients[1] + LM.dN$coefficients[10]
)
p2 <- anova(LM.dN)[3,5] 
R2 <- summary(LM.dN)$adj.r.squared


noquote("Now re-running the regression without location interaction")
   [1] Now re-running the regression without location interaction

noquote("Regressing d15N(Leu-Phe) against TP(Glx-Phe)")
   [1] Regressing d15N(Leu-Phe) against TP(Glx-Phe)
LM.dN = lm(d15NLeu ~ (TPGlx-1) + Location, data = data.reg.phenorm)
summary(LM.dN)
   
   Call:
   lm(formula = d15NLeu ~ (TPGlx - 1) + Location, data = data.reg.phenorm)
   
   Residuals:
       Min      1Q  Median      3Q     Max 
   -2.2125 -0.7745 -0.1825  0.5757  4.9746 
   
   Coefficients:
                   Estimate Std. Error t value Pr(>|t|)    
   TPGlx             6.6112     0.2086  31.689  < 2e-16 ***
   Location5 N      -6.7152     0.6471 -10.377  < 2e-16 ***
   Location8 N      -6.4004     0.6357 -10.068 2.31e-16 ***
   LocationALOHA S  -4.8650     0.5352  -9.091 2.44e-14 ***
   LocationALOHA W  -5.1689     0.5295  -9.761 9.97e-16 ***
   LocationOSP      -6.6910     0.4727 -14.156  < 2e-16 ***
   ---
   Signif. codes:  0 '***' 0.001 '**' 0.01 '*' 0.05 '.' 0.1 ' ' 1
   
   Residual standard error: 1.322 on 89 degrees of freedom
     (3 observations deleted due to missingness)
   Multiple R-squared:  0.9809, Adjusted R-squared:  0.9796 
   F-statistic: 762.2 on 6 and 89 DF,  p-value: < 2.2e-16
anova(LM.dN)
   Analysis of Variance Table
   
   Response: d15NLeu
             Df Sum Sq Mean Sq  F value    Pr(>F)    
   TPGlx      1 7634.6  7634.6 4368.337 < 2.2e-16 ***
   Location   5  358.3    71.7   41.007 < 2.2e-16 ***
   Residuals 89  155.5     1.7                       
   ---
   Signif. codes:  0 '***' 0.001 '**' 0.01 '*' 0.05 '.' 0.1 ' ' 1
coef.1 <- c(
  LM.dN$coefficients[1]
)
p3 <- anova(LM.dN)[1,5] 
R3 <- summary(LM.dN)$adj.r.squared

ggplot(data = data.reg.phenorm, aes(x = (TPGlx-1), y = d15NLeu))+
  geom_point(aes(color=Location))+
  geom_smooth(method=lm, aes(y = predict(LM.dN, data.reg.phenorm), color=Location), lty=1, se=TRUE)+
  xlab(expression(TP[Glx-Phe]-1))+
  ylab(expression(delta^{15}*N[Leu-Phe]*" (\u2030)"))+
  theme_light()+
  facet_wrap(~ Location)
   `geom_smooth()` using formula = 'y ~ x'
   Warning: Removed 3 rows containing non-finite outside the scale range
   (`stat_smooth()`).
   Warning: Removed 3 rows containing missing values or values outside the scale range
   (`geom_point()`).
```

```
noquote("Regressing d15N(Leu-Phe) against TP(Ala-Phe)")
   [1] Regressing d15N(Leu-Phe) against TP(Ala-Phe)
LM.dN = lm(d15NLeu ~ (TPAla-1) + Location, data = data.reg.phenorm)
summary(LM.dN)
   
   Call:
   lm(formula = d15NLeu ~ (TPAla - 1) + Location, data = data.reg.phenorm)
   
   Residuals:
       Min      1Q  Median      3Q     Max 
   -3.0967 -0.6867 -0.0135  1.0315  3.8530 
   
   Coefficients:
                   Estimate Std. Error t value Pr(>|t|)    
   TPAla             4.7187     0.1536  30.727  < 2e-16 ***
   Location5 N      -3.8866     0.6060  -6.413 6.45e-09 ***
   Location8 N      -4.1889     0.6045  -6.929 6.15e-10 ***
   LocationALOHA S  -3.6450     0.5054  -7.212 1.66e-10 ***
   LocationALOHA W  -3.4140     0.5041  -6.772 1.26e-09 ***
   LocationOSP      -5.0692     0.4434 -11.432  < 2e-16 ***
   ---
   Signif. codes:  0 '***' 0.001 '**' 0.01 '*' 0.05 '.' 0.1 ' ' 1
   
   Residual standard error: 1.389 on 90 degrees of freedom
     (2 observations deleted due to missingness)
   Multiple R-squared:  0.9787, Adjusted R-squared:  0.9773 
   F-statistic: 688.6 on 6 and 90 DF,  p-value: < 2.2e-16
anova(LM.dN)
   Analysis of Variance Table
   
   Response: d15NLeu
             Df Sum Sq Mean Sq  F value    Pr(>F)    
   TPAla      1 7720.7  7720.7 3999.856 < 2.2e-16 ***
   Location   5  254.7    50.9   26.392 2.532e-16 ***
   Residuals 90  173.7     1.9                       
   ---
   Signif. codes:  0 '***' 0.001 '**' 0.01 '*' 0.05 '.' 0.1 ' ' 1
coef.2 <- c(
  LM.dN$coefficients[1]
)
p4 <- anova(LM.dN)[1,5] 
R4 <- summary(LM.dN)$adj.r.squared

ggplot(data = data.reg.phenorm, aes(x = (TPAla-1), y = d15NLeu))+
  geom_point(aes(color=Location))+
  geom_smooth(method=lm, aes(y = predict(LM.dN, data.reg.phenorm), color=Location), lty=1, se=TRUE)+
  xlab(expression(TP[Ala-Phe]-1))+
  ylab(expression(delta^{15}*N[Leu-Phe]*" (\u2030)"))+
  theme_light()+
  facet_wrap(~ Location)
   `geom_smooth()` using formula = 'y ~ x'
   Warning: Removed 2 rows containing non-finite outside the scale range
   (`stat_smooth()`).
   Warning: Removed 2 rows containing missing values or values outside the scale range
   (`geom_point()`).
```

```
DN.reg <- data.frame(
  "TP(Glx-Phe)" = coef.1,
  "TP(Ala-Phe)" = coef.2,
  row.names = "d15NLeu"
)

DN.reg
           TP.Glx.Phe. TP.Ala.Phe.
   d15NLeu    6.611247    4.718703
```

1. We tested to see if location had a significant effect on the slope of the regression. The P values associated with this test were 0.4919167 and 0.5508373 for TP(Glx-Phe) and TP(Ala-Phe), respectively. This indicates that location DOES NOT have a significant effect on the slope of the regression.
2. Removing the interaction with location, we tested to see if the slope is significant. The P value associated with these tests were 1.969271^{-77} and 2.1998488^{-76}. This indicates that the slope of the regression IS significant.
3. The \(R^2\) values were 0.9796241 and 0.977261, respectively.

## Regression for Ile

```
noquote("Checking to see if location affects the slope of the regression...")
   [1] Checking to see if location affects the slope of the regression...

noquote("Regressing d15N(Ile-Phe) against TP(Glx-Phe)")
   [1] Regressing d15N(Ile-Phe) against TP(Glx-Phe)
LM.dN = lm(d15NIle ~ (TPGlx-1) * Location, data = data.reg.phenorm)
summary(LM.dN)
   
   Call:
   lm(formula = d15NIle ~ (TPGlx - 1) * Location, data = data.reg.phenorm)
   
   Residuals:
       Min      1Q  Median      3Q     Max 
   -9.1291 -0.7469 -0.0540  0.8095  4.7373 
   
   Coefficients:
                         Estimate Std. Error t value Pr(>|t|)    
   TPGlx                   6.2342     0.7647   8.152  2.8e-12 ***
   Location5 N            -4.4307     1.8928  -2.341   0.0216 *  
   Location8 N            -4.2915     1.9804  -2.167   0.0330 *  
   LocationALOHA S        -3.7364     1.5607  -2.394   0.0189 *  
   LocationALOHA W        -4.6066     1.3386  -3.441   0.0009 ***
   LocationOSP            -8.6585     0.9155  -9.458  6.4e-15 ***
   TPGlx:Location8 N      -0.1239     1.0966  -0.113   0.9103    
   TPGlx:LocationALOHA S   0.1648     1.0583   0.156   0.8766    
   TPGlx:LocationALOHA W   0.1620     0.9807   0.165   0.8692    
   TPGlx:LocationOSP       1.7714     0.8784   2.017   0.0469 *  
   ---
   Signif. codes:  0 '***' 0.001 '**' 0.01 '*' 0.05 '.' 0.1 ' ' 1
   
   Residual standard error: 1.744 on 85 degrees of freedom
     (3 observations deleted due to missingness)
   Multiple R-squared:  0.9732, Adjusted R-squared:  0.9701 
   F-statistic: 309.2 on 10 and 85 DF,  p-value: < 2.2e-16
anova(LM.dN)
   Analysis of Variance Table
   
   Response: d15NIle
                  Df Sum Sq Mean Sq   F value    Pr(>F)    
   TPGlx           1 9023.5  9023.5 2967.4483 < 2.2e-16 ***
   Location        5  349.2    69.8   22.9688 1.618e-14 ***
   TPGlx:Location  4   28.4     7.1    2.3348   0.06201 .  
   Residuals      85  258.5     3.0                        
   ---
   Signif. codes:  0 '***' 0.001 '**' 0.01 '*' 0.05 '.' 0.1 ' ' 1
coef.1 <- c(
  LM.dN$coefficients[1],
  LM.dN$coefficients[1] + LM.dN$coefficients[7],
  LM.dN$coefficients[1] + LM.dN$coefficients[8],
  LM.dN$coefficients[1] + LM.dN$coefficients[9],
  LM.dN$coefficients[1] + LM.dN$coefficients[10]
)
p1 <- anova(LM.dN)[3,5] 
R1 <- summary(LM.dN)$adj.r.squared

noquote("Regressing d15N(Ile-Phe) against TP(Ala-Phe)")
   [1] Regressing d15N(Ile-Phe) against TP(Ala-Phe)
LM.dN = lm(d15NIle ~ (TPAla-1) * Location, data = data.reg.phenorm)
summary(LM.dN)
   
   Call:
   lm(formula = d15NIle ~ (TPAla - 1) * Location, data = data.reg.phenorm)
   
   Residuals:
        Min       1Q   Median       3Q      Max 
   -10.0388  -0.6287   0.1665   0.9562   3.4809 
   
   Coefficients:
                         Estimate Std. Error t value Pr(>|t|)    
   TPAla                   5.0141     0.6378   7.861 1.01e-11 ***
   Location5 N            -3.2979     1.8259  -1.806   0.0744 .  
   Location8 N            -3.6888     1.9449  -1.897   0.0612 .  
   LocationALOHA S        -3.3058     1.3121  -2.519   0.0136 *  
   LocationALOHA W        -2.9318     1.2547  -2.337   0.0218 *  
   LocationOSP            -5.6162     0.7946  -7.068 3.89e-10 ***
   TPAla:Location8 N      -0.1637     0.9007  -0.182   0.8562    
   TPAla:LocationALOHA S  -0.1623     0.8073  -0.201   0.8411    
   TPAla:LocationALOHA W  -0.4399     0.7896  -0.557   0.5789    
   TPAla:LocationOSP       0.2640     0.7039   0.375   0.7085    
   ---
   Signif. codes:  0 '***' 0.001 '**' 0.01 '*' 0.05 '.' 0.1 ' ' 1
   
   Residual standard error: 1.812 on 86 degrees of freedom
     (2 observations deleted due to missingness)
   Multiple R-squared:  0.9708, Adjusted R-squared:  0.9674 
   F-statistic: 285.5 on 10 and 86 DF,  p-value: < 2.2e-16
anova(LM.dN)
   Analysis of Variance Table
   
   Response: d15NIle
                  Df Sum Sq Mean Sq   F value    Pr(>F)    
   TPAla           1 9123.6  9123.6 2777.6380 < 2.2e-16 ***
   Location        5  247.6    49.5   15.0775 1.292e-10 ***
   TPAla:Location  4    6.1     1.5    0.4633    0.7625    
   Residuals      86  282.5     3.3                        
   ---
   Signif. codes:  0 '***' 0.001 '**' 0.01 '*' 0.05 '.' 0.1 ' ' 1
coef.2 <- c(
  LM.dN$coefficients[1],
  LM.dN$coefficients[1] + LM.dN$coefficients[7],
  LM.dN$coefficients[1] + LM.dN$coefficients[8],
  LM.dN$coefficients[1] + LM.dN$coefficients[9],
  LM.dN$coefficients[1] + LM.dN$coefficients[10]
)
p2 <- anova(LM.dN)[3,5] 
R2 <- summary(LM.dN)$adj.r.squared


noquote("Now re-running the regression without location interaction")
   [1] Now re-running the regression without location interaction

noquote("Regressing d15N(Ile-Phe) against TP(Glx-Phe)")
   [1] Regressing d15N(Ile-Phe) against TP(Glx-Phe)
LM.dN = lm(d15NIle ~ (TPGlx-1) + Location, data = data.reg.phenorm)
summary(LM.dN)
   
   Call:
   lm(formula = d15NIle ~ (TPGlx - 1) + Location, data = data.reg.phenorm)
   
   Residuals:
       Min      1Q  Median      3Q     Max 
   -9.4460 -0.7394  0.0302  0.8465  4.5333 
   
   Coefficients:
                   Estimate Std. Error t value Pr(>|t|)    
   TPGlx             6.9931     0.2833  24.682  < 2e-16 ***
   Location5 N      -6.2276     0.8788  -7.086 3.12e-10 ***
   Location8 N      -6.4426     0.8633  -7.463 5.42e-11 ***
   LocationALOHA S  -4.9567     0.7268  -6.820 1.06e-09 ***
   LocationALOHA W  -5.8485     0.7191  -8.133 2.32e-12 ***
   LocationOSP      -6.6216     0.6419 -10.316  < 2e-16 ***
   ---
   Signif. codes:  0 '***' 0.001 '**' 0.01 '*' 0.05 '.' 0.1 ' ' 1
   
   Residual standard error: 1.795 on 89 degrees of freedom
     (3 observations deleted due to missingness)
   Multiple R-squared:  0.9703, Adjusted R-squared:  0.9683 
   F-statistic: 484.6 on 6 and 89 DF,  p-value: < 2.2e-16
anova(LM.dN)
   Analysis of Variance Table
   
   Response: d15NIle
             Df Sum Sq Mean Sq  F value    Pr(>F)    
   TPGlx      1 9023.5  9023.5 2799.500 < 2.2e-16 ***
   Location   5  349.2    69.8   21.669 3.966e-14 ***
   Residuals 89  286.9     3.2                       
   ---
   Signif. codes:  0 '***' 0.001 '**' 0.01 '*' 0.05 '.' 0.1 ' ' 1
coef.1 <- c(
  LM.dN$coefficients[1]
)
p3 <- anova(LM.dN)[1,5] 
R3 <- summary(LM.dN)$adj.r.squared

ggplot(data = data.reg.phenorm, aes(x = (TPGlx-1), y = d15NIle))+
  geom_point(aes(color=Location))+
  geom_smooth(method=lm, aes(y = predict(LM.dN, data.reg.phenorm), color=Location), lty=1, se=TRUE)+
  xlab(expression(TP[Glx-Phe]-1))+
  ylab(expression(delta^{15}*N[Ile-Phe]*" (\u2030)"))+
  theme_light()+
  facet_wrap(~ Location)
   `geom_smooth()` using formula = 'y ~ x'
   Warning: Removed 3 rows containing non-finite outside the scale range
   (`stat_smooth()`).
   Warning: Removed 3 rows containing missing values or values outside the scale range
   (`geom_point()`).
```

```
noquote("Regressing d15N(Ile-Phe) against TP(Ala-Phe)")
   [1] Regressing d15N(Ile-Phe) against TP(Ala-Phe)
LM.dN = lm(d15NIle ~ (TPAla-1) + Location, data = data.reg.phenorm)
summary(LM.dN)
   
   Call:
   lm(formula = d15NIle ~ (TPAla - 1) + Location, data = data.reg.phenorm)
   
   Residuals:
        Min       1Q   Median       3Q      Max 
   -10.4484  -0.6857   0.0998   0.8654   3.6079 
   
   Coefficients:
                   Estimate Std. Error t value Pr(>|t|)    
   TPAla             5.0095     0.1979  25.310  < 2e-16 ***
   Location5 N      -3.2851     0.7810  -4.206 6.13e-05 ***
   Location8 N      -4.1571     0.7791  -5.335 7.05e-07 ***
   LocationALOHA S  -3.7009     0.6514  -5.682 1.62e-07 ***
   LocationALOHA W  -4.0385     0.6497  -6.216 1.56e-08 ***
   LocationOSP      -4.9512     0.5715  -8.663 1.73e-13 ***
   ---
   Signif. codes:  0 '***' 0.001 '**' 0.01 '*' 0.05 '.' 0.1 ' ' 1
   
   Residual standard error: 1.791 on 90 degrees of freedom
     (2 observations deleted due to missingness)
   Multiple R-squared:  0.9701, Adjusted R-squared:  0.9681 
   F-statistic: 487.1 on 6 and 90 DF,  p-value: < 2.2e-16
anova(LM.dN)
   Analysis of Variance Table
   
   Response: d15NIle
             Df Sum Sq Mean Sq  F value    Pr(>F)    
   TPAla      1 9123.6  9123.6 2845.515 < 2.2e-16 ***
   Location   5  247.6    49.5   15.446 6.011e-11 ***
   Residuals 90  288.6     3.2                       
   ---
   Signif. codes:  0 '***' 0.001 '**' 0.01 '*' 0.05 '.' 0.1 ' ' 1
coef.2 <- c(
  LM.dN$coefficients[1]
)
p4 <- anova(LM.dN)[1,5] 
R4 <- summary(LM.dN)$adj.r.squared

ggplot(data = data.reg.phenorm, aes(x = (TPAla-1), y = d15NIle))+
  geom_point(aes(color=Location))+
  geom_smooth(method=lm, aes(y = predict(LM.dN, data.reg.phenorm), color=Location), lty=1, se=TRUE)+
  xlab(expression(TP[Ala-Phe]-1))+
  ylab(expression(delta^{15}*N[Ile-Phe]*" (\u2030)"))+
  theme_light()+
  facet_wrap(~ Location)
   `geom_smooth()` using formula = 'y ~ x'
   Warning: Removed 2 rows containing non-finite outside the scale range
   (`stat_smooth()`).
   Warning: Removed 2 rows containing missing values or values outside the scale range
   (`geom_point()`).
```

```
DN.reg <- data.frame(
  "TP(Glx-Phe)" = coef.1,
  "TP(Ala-Phe)" = coef.2,
  row.names = "d15NIle"
)

DN.reg
           TP.Glx.Phe. TP.Ala.Phe.
   d15NIle    6.993086     5.00945
```

1. We tested to see if location had a significant effect on the slope of the regression. The P values associated with this test were 0.0620129 and 0.7624793 for TP(Glx-Phe) and TP(Ala-Phe), respectively. This indicates that location DOES NOT have a significant effect on the slope of the regression.
2. Removing the interaction with location, we tested to see if the slope is significant. The P value associated with these tests were 4.7929884^{-69} and 6.6886931^{-70}. This indicates that the slope of the regression IS significant.
3. The \(R^2\) values were 0.9683 and 0.9681354, respectively.

## Regression for Pro

```
noquote("Checking to see if location affects the slope of the regression...")
   [1] Checking to see if location affects the slope of the regression...

noquote("Regressing d15N(Pro-Phe) against TP(Glx-Phe)")
   [1] Regressing d15N(Pro-Phe) against TP(Glx-Phe)
LM.dN = lm(d15NPro ~ (TPGlx-1) * Location, data = data.reg.phenorm)
summary(LM.dN)
   
   Call:
   lm(formula = d15NPro ~ (TPGlx - 1) * Location, data = data.reg.phenorm)
   
   Residuals:
       Min      1Q  Median      3Q     Max 
   -4.5700 -0.7884 -0.0331  0.7997  3.8791 
   
   Coefficients:
                         Estimate Std. Error t value Pr(>|t|)    
   TPGlx                   4.9855     0.5993   8.319 1.29e-12 ***
   Location5 N            -2.9989     1.4833  -2.022   0.0463 *  
   Location8 N            -3.5261     1.5519  -2.272   0.0256 *  
   LocationALOHA S        -2.9080     1.2230  -2.378   0.0197 *  
   LocationALOHA W        -2.0496     1.0489  -1.954   0.0540 .  
   LocationOSP            -6.0965     0.7174  -8.498 5.61e-13 ***
   TPGlx:Location8 N       0.3250     0.8594   0.378   0.7063    
   TPGlx:LocationALOHA S   0.2186     0.8293   0.264   0.7927    
   TPGlx:LocationALOHA W  -0.1595     0.7685  -0.207   0.8361    
   TPGlx:LocationOSP       1.5585     0.6883   2.264   0.0261 *  
   ---
   Signif. codes:  0 '***' 0.001 '**' 0.01 '*' 0.05 '.' 0.1 ' ' 1
   
   Residual standard error: 1.367 on 85 degrees of freedom
     (3 observations deleted due to missingness)
   Multiple R-squared:  0.9785, Adjusted R-squared:  0.976 
   F-statistic: 387.6 on 10 and 85 DF,  p-value: < 2.2e-16
anova(LM.dN)
   Analysis of Variance Table
   
   Response: d15NPro
                  Df Sum Sq Mean Sq   F value    Pr(>F)    
   TPGlx           1 7065.6  7065.6 3783.7407 < 2.2e-16 ***
   Location        5  148.9    29.8   15.9527 4.621e-11 ***
   TPGlx:Location  4   22.5     5.6    3.0097   0.02252 *  
   Residuals      85  158.7     1.9                        
   ---
   Signif. codes:  0 '***' 0.001 '**' 0.01 '*' 0.05 '.' 0.1 ' ' 1
coef.1 <- c(
  LM.dN$coefficients[1],
  LM.dN$coefficients[1] + LM.dN$coefficients[7],
  LM.dN$coefficients[1] + LM.dN$coefficients[8],
  LM.dN$coefficients[1] + LM.dN$coefficients[9],
  LM.dN$coefficients[1] + LM.dN$coefficients[10]
)
p1 <- anova(LM.dN)[3,5] 
R1 <- summary(LM.dN)$adj.r.squared

noquote("Regressing d15N(Pro-Phe) against TP(Ala-Phe)")
   [1] Regressing d15N(Pro-Phe) against TP(Ala-Phe)
LM.dN = lm(d15NPro ~ (TPAla-1) * Location, data = data.reg.phenorm)
summary(LM.dN)
   
   Call:
   lm(formula = d15NPro ~ (TPAla - 1) * Location, data = data.reg.phenorm)
   
   Residuals:
       Min      1Q  Median      3Q     Max 
   -5.2512 -0.9991  0.0466  1.0334  3.9677 
   
   Coefficients:
                         Estimate Std. Error t value Pr(>|t|)    
   TPAla                   3.9433     0.5555   7.099 3.38e-10 ***
   Location5 N            -1.9122     1.5901  -1.203    0.232    
   Location8 N            -2.8036     1.6937  -1.655    0.102    
   LocationALOHA S        -1.1175     1.1426  -0.978    0.331    
   LocationALOHA W        -0.1306     1.0927  -0.120    0.905    
   LocationOSP            -3.6459     0.6919  -5.269 1.00e-06 ***
   TPAla:Location8 N       0.2048     0.7844   0.261    0.795    
   TPAla:LocationALOHA S  -0.5015     0.7031  -0.713    0.478    
   TPAla:LocationALOHA W  -0.7496     0.6877  -1.090    0.279    
   TPAla:LocationOSP       0.3859     0.6130   0.630    0.531    
   ---
   Signif. codes:  0 '***' 0.001 '**' 0.01 '*' 0.05 '.' 0.1 ' ' 1
   
   Residual standard error: 1.578 on 86 degrees of freedom
     (2 observations deleted due to missingness)
   Multiple R-squared:  0.9711, Adjusted R-squared:  0.9677 
   F-statistic: 288.6 on 10 and 86 DF,  p-value: < 2.2e-16
anova(LM.dN)
   Analysis of Variance Table
   
   Response: d15NPro
                  Df Sum Sq Mean Sq   F value    Pr(>F)    
   TPAla           1 7101.3  7101.3 2850.7491 < 2.2e-16 ***
   Location        5   71.1    14.2    5.7112 0.0001348 ***
   TPAla:Location  4   17.7     4.4    1.7763 0.1409763    
   Residuals      86  214.2     2.5                        
   ---
   Signif. codes:  0 '***' 0.001 '**' 0.01 '*' 0.05 '.' 0.1 ' ' 1
coef.2 <- c(
  LM.dN$coefficients[1],
  LM.dN$coefficients[1] + LM.dN$coefficients[7],
  LM.dN$coefficients[1] + LM.dN$coefficients[8],
  LM.dN$coefficients[1] + LM.dN$coefficients[9],
  LM.dN$coefficients[1] + LM.dN$coefficients[10]
)
p2 <- anova(LM.dN)[3,5] 
R2 <- summary(LM.dN)$adj.r.squared


noquote("Now re-running the regression without location interaction")
   [1] Now re-running the regression without location interaction

noquote("Regressing d15N(Pro-Phe) against TP(Glx-Phe)")
   [1] Regressing d15N(Pro-Phe) against TP(Glx-Phe)
LM.dN = lm(d15NPro ~ (TPGlx-1) + Location, data = data.reg.phenorm)
summary(LM.dN)
   
   Call:
   lm(formula = d15NPro ~ (TPGlx - 1) + Location, data = data.reg.phenorm)
   
   Residuals:
       Min      1Q  Median      3Q     Max 
   -4.7350 -0.7281 -0.1052  0.7952  3.5777 
   
   Coefficients:
                   Estimate Std. Error t value Pr(>|t|)    
   TPGlx             5.6562     0.2252  25.118  < 2e-16 ***
   Location5 N      -4.5869     0.6985  -6.567 3.34e-09 ***
   Location8 N      -4.3685     0.6861  -6.367 8.21e-09 ***
   LocationALOHA S  -3.8364     0.5776  -6.642 2.38e-09 ***
   LocationALOHA W  -3.7767     0.5715  -6.608 2.78e-09 ***
   LocationOSP      -4.3103     0.5102  -8.449 5.18e-13 ***
   ---
   Signif. codes:  0 '***' 0.001 '**' 0.01 '*' 0.05 '.' 0.1 ' ' 1
   
   Residual standard error: 1.427 on 89 degrees of freedom
     (3 observations deleted due to missingness)
   Multiple R-squared:  0.9755, Adjusted R-squared:  0.9738 
   F-statistic: 590.6 on 6 and 89 DF,  p-value: < 2.2e-16
anova(LM.dN)
   Analysis of Variance Table
   
   Response: d15NPro
             Df Sum Sq Mean Sq  F value    Pr(>F)    
   TPGlx      1 7065.6  7065.6 3470.289 < 2.2e-16 ***
   Location   5  148.9    29.8   14.631 1.869e-10 ***
   Residuals 89  181.2     2.0                       
   ---
   Signif. codes:  0 '***' 0.001 '**' 0.01 '*' 0.05 '.' 0.1 ' ' 1
coef.1 <- c(
  LM.dN$coefficients[1]
)
p3 <- anova(LM.dN)[1,5] 
R3 <- summary(LM.dN)$adj.r.squared

ggplot(data = data.reg.phenorm, aes(x = (TPGlx-1), y = d15NPro))+
  geom_point(aes(color=Location))+
  geom_smooth(method=lm, aes(y = predict(LM.dN, data.reg.phenorm), color=Location), lty=1, se=TRUE)+
  xlab(expression(TP[Glx-Phe]-1))+
  ylab(expression(delta^{15}*N[Pro-Phe]*" (\u2030)"))+
  theme_light()+
  facet_wrap(~ Location)
   `geom_smooth()` using formula = 'y ~ x'
   Warning: Removed 3 rows containing non-finite outside the scale range
   (`stat_smooth()`).
   Warning: Removed 3 rows containing missing values or values outside the scale range
   (`geom_point()`).
```

```
noquote("Regressing d15N(Pro-Phe) against TP(Ala-Phe)")
   [1] Regressing d15N(Pro-Phe) against TP(Ala-Phe)
LM.dN = lm(d15NPro ~ (TPAla-1) + Location, data = data.reg.phenorm)
summary(LM.dN)
   
   Call:
   lm(formula = d15NPro ~ (TPAla - 1) + Location, data = data.reg.phenorm)
   
   Residuals:
       Min      1Q  Median      3Q     Max 
   -5.6229 -0.7607 -0.0742  0.8116  3.6730 
   
   Coefficients:
                   Estimate Std. Error t value Pr(>|t|)    
   TPAla             3.9174     0.1774  22.078  < 2e-16 ***
   Location5 N      -1.8419     0.7002  -2.631 0.010028 *  
   Location8 N      -2.1243     0.6985  -3.041 0.003086 ** 
   LocationALOHA S  -2.3099     0.5840  -3.955 0.000152 ***
   LocationALOHA W  -1.9712     0.5825  -3.384 0.001059 ** 
   LocationOSP      -2.6269     0.5124  -5.127 1.67e-06 ***
   ---
   Signif. codes:  0 '***' 0.001 '**' 0.01 '*' 0.05 '.' 0.1 ' ' 1
   
   Residual standard error: 1.605 on 90 degrees of freedom
     (2 observations deleted due to missingness)
   Multiple R-squared:  0.9687, Adjusted R-squared:  0.9666 
   F-statistic: 463.9 on 6 and 90 DF,  p-value: < 2.2e-16
anova(LM.dN)
   Analysis of Variance Table
   
   Response: d15NPro
             Df Sum Sq Mean Sq   F value    Pr(>F)    
   TPAla      1 7101.3  7101.3 2755.6717 < 2.2e-16 ***
   Location   5   71.1    14.2    5.5208 0.0001766 ***
   Residuals 90  231.9     2.6                        
   ---
   Signif. codes:  0 '***' 0.001 '**' 0.01 '*' 0.05 '.' 0.1 ' ' 1
coef.2 <- c(
  LM.dN$coefficients[1]
)
p4 <- anova(LM.dN)[1,5] 
R4 <- summary(LM.dN)$adj.r.squared

ggplot(data = data.reg.phenorm, aes(x = (TPAla-1), y = d15NPro))+
  geom_point(aes(color=Location))+
  geom_smooth(method=lm, aes(y = predict(LM.dN, data.reg.phenorm), color=Location), lty=1, se=TRUE)+
  xlab(expression(TP[Ala-Phe]-1))+
  ylab(expression(delta^{15}*N[Pro-Phe]*" (\u2030)"))+
  theme_light()+
  facet_wrap(~ Location)
   `geom_smooth()` using formula = 'y ~ x'
   Warning: Removed 2 rows containing non-finite outside the scale range
   (`stat_smooth()`).
   Warning: Removed 2 rows containing missing values or values outside the scale range
   (`geom_point()`).
```

```
DN.reg <- data.frame(
  "TP(Glx-Phe)" = coef.1,
  "TP(Ala-Phe)" = coef.2,
  row.names = "d15NPro"
)

DN.reg
           TP.Glx.Phe. TP.Ala.Phe.
   d15NPro    5.656163    3.917444
```

1. We tested to see if location had a significant effect on the slope of the regression. The P values associated with this test were 0.0225198 and 0.1409763 for TP(Glx-Phe) and TP(Ala-Phe), respectively. This indicates that location MIGHT have a significant effect on the slope of the regression.
2. Removing the interaction with location, we tested to see if the slope is significant. The P value associated with these tests were 4.4013899^{-73} and 2.7104047^{-69}. This indicates that the slope of the regression IS significant.
3. The \(R^2\) values were 0.9738468 and 0.9665887, respectively.

## Regression for Asx

```
noquote("Checking to see if location affects the slope of the regression...")
   [1] Checking to see if location affects the slope of the regression...

noquote("Regressing d15N(Asx-Phe) against TP(Glx-Phe)")
   [1] Regressing d15N(Asx-Phe) against TP(Glx-Phe)
LM.dN = lm(d15NAsx ~ (TPGlx-1) * Location, data = data.reg.phenorm)
summary(LM.dN)
   
   Call:
   lm(formula = d15NAsx ~ (TPGlx - 1) * Location, data = data.reg.phenorm)
   
   Residuals:
        Min       1Q   Median       3Q      Max 
   -2.08438 -0.46900  0.02209  0.41085  2.04097 
   
   Coefficients:
                         Estimate Std. Error t value Pr(>|t|)    
   TPGlx                   4.9529     0.3574  13.857  < 2e-16 ***
   Location5 N            -1.3937     0.8847  -1.575  0.11889    
   Location8 N            -2.3289     0.9256  -2.516  0.01375 *  
   LocationALOHA S        -3.6600     0.7295  -5.017 2.84e-06 ***
   LocationALOHA W        -3.7268     0.6256  -5.957 5.63e-08 ***
   LocationOSP            -4.0661     0.4279  -9.503 5.19e-15 ***
   TPGlx:Location8 N       0.6244     0.5126   1.218  0.22648    
   TPGlx:LocationALOHA S   1.2613     0.4946   2.550  0.01256 *  
   TPGlx:LocationALOHA W   1.2211     0.4584   2.664  0.00923 ** 
   TPGlx:LocationOSP       1.1308     0.4106   2.754  0.00719 ** 
   ---
   Signif. codes:  0 '***' 0.001 '**' 0.01 '*' 0.05 '.' 0.1 ' ' 1
   
   Residual standard error: 0.815 on 85 degrees of freedom
     (3 observations deleted due to missingness)
   Multiple R-squared:  0.9941, Adjusted R-squared:  0.9934 
   F-statistic:  1424 on 10 and 85 DF,  p-value: < 2.2e-16
anova(LM.dN)
   Analysis of Variance Table
   
   Response: d15NAsx
                  Df Sum Sq Mean Sq   F value  Pr(>F)    
   TPGlx           1 9341.8  9341.8 14062.973 < 2e-16 ***
   Location        5  111.2    22.2    33.474 < 2e-16 ***
   TPGlx:Location  4    6.9     1.7     2.592 0.04221 *  
   Residuals      85   56.5     0.7                      
   ---
   Signif. codes:  0 '***' 0.001 '**' 0.01 '*' 0.05 '.' 0.1 ' ' 1
coef.1 <- c(
  LM.dN$coefficients[1],
  LM.dN$coefficients[1] + LM.dN$coefficients[7],
  LM.dN$coefficients[1] + LM.dN$coefficients[8],
  LM.dN$coefficients[1] + LM.dN$coefficients[9],
  LM.dN$coefficients[1] + LM.dN$coefficients[10]
)
p1 <- anova(LM.dN)[3,5] 
R1 <- summary(LM.dN)$adj.r.squared

noquote("Regressing d15N(Asx-Phe) against TP(Ala-Phe)")
   [1] Regressing d15N(Asx-Phe) against TP(Ala-Phe)
LM.dN = lm(d15NAsx ~ (TPAla-1) * Location, data = data.reg.phenorm)
summary(LM.dN)
   
   Call:
   lm(formula = d15NAsx ~ (TPAla - 1) * Location, data = data.reg.phenorm)
   
   Residuals:
        Min       1Q   Median       3Q      Max 
   -2.43275 -0.64719  0.04738  0.68964  2.47047 
   
   Coefficients:
                         Estimate Std. Error t value Pr(>|t|)    
   TPAla                  3.96577    0.35373  11.211  < 2e-16 ***
   Location5 N           -0.44518    1.01258  -0.440 0.661298    
   Location8 N           -1.51319    1.07859  -1.403 0.164239    
   LocationALOHA S       -2.36743    0.72765  -3.254 0.001630 ** 
   LocationALOHA W       -2.07022    0.69583  -2.975 0.003800 ** 
   LocationOSP           -1.71795    0.44064  -3.899 0.000191 ***
   TPAla:Location8 N      0.37144    0.49952   0.744 0.459146    
   TPAla:LocationALOHA S  0.45260    0.44772   1.011 0.314901    
   TPAla:LocationALOHA W  0.43389    0.43791   0.991 0.324559    
   TPAla:LocationOSP      0.03062    0.39034   0.078 0.937650    
   ---
   Signif. codes:  0 '***' 0.001 '**' 0.01 '*' 0.05 '.' 0.1 ' ' 1
   
   Residual standard error: 1.005 on 86 degrees of freedom
     (2 observations deleted due to missingness)
   Multiple R-squared:  0.9909, Adjusted R-squared:  0.9898 
   F-statistic: 933.8 on 10 and 86 DF,  p-value: < 2.2e-16
anova(LM.dN)
   Analysis of Variance Table
   
   Response: d15NAsx
                  Df Sum Sq Mean Sq   F value    Pr(>F)    
   TPAla           1 9373.4  9373.4 9278.9143 < 2.2e-16 ***
   Location        5   56.8    11.4   11.2475 2.328e-08 ***
   TPAla:Location  4    3.3     0.8    0.8187    0.5167    
   Residuals      86   86.9     1.0                        
   ---
   Signif. codes:  0 '***' 0.001 '**' 0.01 '*' 0.05 '.' 0.1 ' ' 1
coef.2 <- c(
  LM.dN$coefficients[1],
  LM.dN$coefficients[1] + LM.dN$coefficients[7],
  LM.dN$coefficients[1] + LM.dN$coefficients[8],
  LM.dN$coefficients[1] + LM.dN$coefficients[9],
  LM.dN$coefficients[1] + LM.dN$coefficients[10]
)
p2 <- anova(LM.dN)[3,5] 
R2 <- summary(LM.dN)$adj.r.squared


noquote("Now re-running the regression without location interaction")
   [1] Now re-running the regression without location interaction

noquote("Regressing d15N(Asx-Phe) against TP(Glx-Phe)")
   [1] Regressing d15N(Asx-Phe) against TP(Glx-Phe)
LM.dN = lm(d15NAsx ~ (TPGlx-1) + Location, data = data.reg.phenorm)
summary(LM.dN)
   
   Call:
   lm(formula = d15NAsx ~ (TPGlx - 1) + Location, data = data.reg.phenorm)
   
   Residuals:
        Min       1Q   Median       3Q      Max 
   -2.33128 -0.49187 -0.03393  0.49708  1.89092 
   
   Coefficients:
                   Estimate Std. Error t value Pr(>|t|)    
   TPGlx             5.9118     0.1331  44.401  < 2e-16 ***
   Location5 N      -3.6641     0.4130  -8.872 6.91e-14 ***
   Location8 N      -3.1440     0.4057  -7.750 1.41e-11 ***
   LocationALOHA S  -3.0389     0.3415  -8.898 6.11e-14 ***
   LocationALOHA W  -3.1813     0.3379  -9.414 5.22e-15 ***
   LocationOSP      -3.7203     0.3016 -12.334  < 2e-16 ***
   ---
   Signif. codes:  0 '***' 0.001 '**' 0.01 '*' 0.05 '.' 0.1 ' ' 1
   
   Residual standard error: 0.8437 on 89 degrees of freedom
     (3 observations deleted due to missingness)
   Multiple R-squared:  0.9933, Adjusted R-squared:  0.9929 
   F-statistic:  2213 on 6 and 89 DF,  p-value: < 2.2e-16
anova(LM.dN)
   Analysis of Variance Table
   
   Response: d15NAsx
             Df Sum Sq Mean Sq   F value    Pr(>F)    
   TPGlx      1 9341.8  9341.8 13123.950 < 2.2e-16 ***
   Location   5  111.2    22.2    31.239 < 2.2e-16 ***
   Residuals 89   63.4     0.7                        
   ---
   Signif. codes:  0 '***' 0.001 '**' 0.01 '*' 0.05 '.' 0.1 ' ' 1
coef.1 <- c(
  LM.dN$coefficients[1]
)
p3 <- anova(LM.dN)[1,5] 
R3 <- summary(LM.dN)$adj.r.squared

ggplot(data = data.reg.phenorm, aes(x = (TPGlx-1), y = d15NAsx))+
  geom_point(aes(color=Location))+
  geom_smooth(method=lm, aes(y = predict(LM.dN, data.reg.phenorm), color=Location), lty=1, se=TRUE)+
  xlab(expression(TP[Glx-Phe]-1))+
  ylab(expression(delta^{15}*N[Asx-Phe]*" (\u2030)"))+
  theme_light()+
  facet_wrap(~ Location)
   `geom_smooth()` using formula = 'y ~ x'
   Warning: Removed 3 rows containing non-finite outside the scale range
   (`stat_smooth()`).
   Warning: Removed 3 rows containing missing values or values outside the scale range
   (`geom_point()`).
```

```
noquote("Regressing d15N(Asx-Phe) against TP(Ala-Phe)")
   [1] Regressing d15N(Asx-Phe) against TP(Ala-Phe)
LM.dN = lm(d15NAsx ~ (TPAla-1) + Location, data = data.reg.phenorm)
summary(LM.dN)
   
   Call:
   lm(formula = d15NAsx ~ (TPAla - 1) + Location, data = data.reg.phenorm)
   
   Residuals:
        Min       1Q   Median       3Q      Max 
   -2.31531 -0.65632 -0.00487  0.74252  2.70567 
   
   Coefficients:
                   Estimate Std. Error t value Pr(>|t|)    
   TPAla             4.1710     0.1106  37.697  < 2e-16 ***
   Location5 N      -1.0030     0.4366  -2.297   0.0239 *  
   Location8 N      -1.0237     0.4356  -2.350   0.0209 *  
   LocationALOHA S  -1.7474     0.3641  -4.799 6.31e-06 ***
   LocationALOHA W  -1.4888     0.3632  -4.099 9.07e-05 ***
   LocationOSP      -2.1501     0.3195  -6.730 1.54e-09 ***
   ---
   Signif. codes:  0 '***' 0.001 '**' 0.01 '*' 0.05 '.' 0.1 ' ' 1
   
   Residual standard error: 1.001 on 90 degrees of freedom
     (2 observations deleted due to missingness)
   Multiple R-squared:  0.9905, Adjusted R-squared:  0.9899 
   F-statistic:  1568 on 6 and 90 DF,  p-value: < 2.2e-16
anova(LM.dN)
   Analysis of Variance Table
   
   Response: d15NAsx
             Df Sum Sq Mean Sq  F value    Pr(>F)    
   TPAla      1 9373.4  9373.4 9354.292 < 2.2e-16 ***
   Location   5   56.8    11.4   11.339 1.695e-08 ***
   Residuals 90   90.2     1.0                       
   ---
   Signif. codes:  0 '***' 0.001 '**' 0.01 '*' 0.05 '.' 0.1 ' ' 1
coef.2 <- c(
  LM.dN$coefficients[1]
)
p4 <- anova(LM.dN)[1,5] 
R4 <- summary(LM.dN)$adj.r.squared

ggplot(data = data.reg.phenorm, aes(x = (TPAla-1), y = d15NAsx))+
  geom_point(aes(color=Location))+
  geom_smooth(method=lm, aes(y = predict(LM.dN, data.reg.phenorm), color=Location), lty=1, se=TRUE)+
  xlab(expression(TP[Ala-Phe]-1))+
  ylab(expression(delta^{15}*N[Asx-Phe]*" (\u2030)"))+
  theme_light()+
  facet_wrap(~ Location)
   `geom_smooth()` using formula = 'y ~ x'
   Warning: Removed 2 rows containing non-finite outside the scale range
   (`stat_smooth()`).
   Warning: Removed 2 rows containing missing values or values outside the scale range
   (`geom_point()`).
```

```
DN.reg <- data.frame(
  "TP(Glx-Phe)" = coef.1,
  "TP(Ala-Phe)" = coef.2,
  row.names = "d15NAsx"
)

DN.reg
           TP.Glx.Phe. TP.Ala.Phe.
   d15NAsx    5.911821     4.17102
```

1. We tested to see if location had a significant effect on the slope of the regression. The P values associated with this test were 0.0422147 and 0.5167335 for TP(Glx-Phe) and TP(Ala-Phe), respectively. This indicates that location MIGHT have a significant effect on the slope of the regression.
2. Removing the interaction with location, we tested to see if the slope is significant. The P value associated with these tests were 1.9535493^{-98} and 9.6368499^{-93}. This indicates that the slope of the regression IS significant.
3. The \(R^2\) values were 0.9928941 and 0.9898958, respectively.

## Regression for Met

```
noquote("Checking to see if location affects the slope of the regression...")
   [1] Checking to see if location affects the slope of the regression...

noquote("Regressing d15N(Met-Phe) against TP(Glx-Phe)")
   [1] Regressing d15N(Met-Phe) against TP(Glx-Phe)
LM.dN = lm(d15NMet ~ (TPGlx-1) * Location, data = data.reg.phenorm)
summary(LM.dN)
   
   Call:
   lm(formula = d15NMet ~ (TPGlx - 1) * Location, data = data.reg.phenorm)
   
   Residuals:
       Min      1Q  Median      3Q     Max 
   -3.9554 -0.4333  0.0000  0.6478  3.0509 
   
   Coefficients:
                         Estimate Std. Error t value Pr(>|t|)    
   TPGlx                 -27.6699    88.6776  -0.312 0.756460    
   Location5 N            39.0807   105.5075   0.370 0.712817    
   Location8 N            -3.9070    17.6743  -0.221 0.826047    
   LocationALOHA S         0.1634     1.1953   0.137 0.891890    
   LocationALOHA W        -0.7950     1.0252  -0.776 0.442088    
   LocationOSP            -5.5348     1.3467  -4.110 0.000165 ***
   TPGlx:Location8 N      36.0809    89.8210   0.402 0.689808    
   TPGlx:LocationALOHA S  29.6415    88.6794   0.334 0.739740    
   TPGlx:LocationALOHA W  30.0585    88.6788   0.339 0.736217    
   TPGlx:LocationOSP      30.9323    88.6794   0.349 0.728858    
   ---
   Signif. codes:  0 '***' 0.001 '**' 0.01 '*' 0.05 '.' 0.1 ' ' 1
   
   Residual standard error: 1.336 on 45 degrees of freedom
     (43 observations deleted due to missingness)
   Multiple R-squared:  0.9242, Adjusted R-squared:  0.9074 
   F-statistic: 54.87 on 10 and 45 DF,  p-value: < 2.2e-16
anova(LM.dN)
   Analysis of Variance Table
   
   Response: d15NMet
                  Df Sum Sq Mean Sq F value    Pr(>F)    
   TPGlx           1 815.78  815.78  457.37 < 2.2e-16 ***
   Location        5 157.40   31.48   17.65 1.196e-09 ***
   TPGlx:Location  4   5.42    1.36    0.76    0.5568    
   Residuals      45  80.26    1.78                      
   ---
   Signif. codes:  0 '***' 0.001 '**' 0.01 '*' 0.05 '.' 0.1 ' ' 1
coef.1 <- c(
  LM.dN$coefficients[1],
  LM.dN$coefficients[1] + LM.dN$coefficients[7],
  LM.dN$coefficients[1] + LM.dN$coefficients[8],
  LM.dN$coefficients[1] + LM.dN$coefficients[9],
  LM.dN$coefficients[1] + LM.dN$coefficients[10]
)
p1 <- anova(LM.dN)[3,5] 
R1 <- summary(LM.dN)$adj.r.squared

noquote("Regressing d15N(Met-Phe) against TP(Ala-Phe)")
   [1] Regressing d15N(Met-Phe) against TP(Ala-Phe)
LM.dN = lm(d15NMet ~ (TPAla-1) * Location, data = data.reg.phenorm)
summary(LM.dN)
   
   Call:
   lm(formula = d15NMet ~ (TPAla - 1) * Location, data = data.reg.phenorm)
   
   Residuals:
       Min      1Q  Median      3Q     Max 
   -4.3289 -0.4733  0.0000  0.8868  3.6903 
   
   Coefficients:
                         Estimate Std. Error t value Pr(>|t|)   
   TPAla                  -3.3618    12.5921  -0.267  0.79068   
   Location5 N            10.0542    14.6252   0.687  0.49525   
   Location8 N           -23.9708    60.4651  -0.396  0.69361   
   LocationALOHA S         0.6496     1.1300   0.575  0.56820   
   LocationALOHA W         0.4040     1.0806   0.374  0.71019   
   LocationOSP            -3.8167     1.4082  -2.710  0.00941 **
   TPAla:Location8 N      26.5127    47.6501   0.556  0.58063   
   TPAla:LocationALOHA S   4.7214    12.5993   0.375  0.70958   
   TPAla:LocationALOHA W   4.8445    12.5985   0.385  0.70236   
   TPAla:LocationOSP       5.3560    12.6007   0.425  0.67278   
   ---
   Signif. codes:  0 '***' 0.001 '**' 0.01 '*' 0.05 '.' 0.1 ' ' 1
   
   Residual standard error: 1.561 on 46 degrees of freedom
     (42 observations deleted due to missingness)
   Multiple R-squared:  0.8944, Adjusted R-squared:  0.8714 
   F-statistic: 38.95 on 10 and 46 DF,  p-value: < 2.2e-16
anova(LM.dN)
   Analysis of Variance Table
   
   Response: d15NMet
                  Df Sum Sq Mean Sq  F value    Pr(>F)    
   TPAla           1 781.23  781.23 320.6611 < 2.2e-16 ***
   Location        5 163.96   32.79  13.4595 4.254e-08 ***
   TPAla:Location  4   3.63    0.91   0.3729    0.8267    
   Residuals      46 112.07    2.44                       
   ---
   Signif. codes:  0 '***' 0.001 '**' 0.01 '*' 0.05 '.' 0.1 ' ' 1
coef.2 <- c(
  LM.dN$coefficients[1],
  LM.dN$coefficients[1] + LM.dN$coefficients[7],
  LM.dN$coefficients[1] + LM.dN$coefficients[8],
  LM.dN$coefficients[1] + LM.dN$coefficients[9],
  LM.dN$coefficients[1] + LM.dN$coefficients[10]
)
p2 <- anova(LM.dN)[3,5] 
R2 <- summary(LM.dN)$adj.r.squared


noquote("Now re-running the regression without location interaction")
   [1] Now re-running the regression without location interaction

noquote("Regressing d15N(Met-Phe) against TP(Glx-Phe)")
   [1] Regressing d15N(Met-Phe) against TP(Glx-Phe)
LM.dN = lm(d15NMet ~ (TPGlx-1) + Location, data = data.reg.phenorm)
summary(LM.dN)
   
   Call:
   lm(formula = d15NMet ~ (TPGlx - 1) + Location, data = data.reg.phenorm)
   
   Residuals:
       Min      1Q  Median      3Q     Max 
   -4.3493 -0.5518  0.0887  0.8830  3.1191 
   
   Coefficients:
                   Estimate Std. Error t value Pr(>|t|)    
   TPGlx             2.5197     0.3009   8.375 5.14e-11 ***
   Location5 N       3.1629     1.0012   3.159  0.00271 ** 
   Location8 N       3.3711     1.0062   3.350  0.00156 ** 
   LocationALOHA S  -0.9623     0.6962  -1.382  0.17316    
   LocationALOHA W  -1.0679     0.6956  -1.535  0.13118    
   LocationOSP      -3.8281     0.7711  -4.964 8.75e-06 ***
   ---
   Signif. codes:  0 '***' 0.001 '**' 0.01 '*' 0.05 '.' 0.1 ' ' 1
   
   Residual standard error: 1.322 on 49 degrees of freedom
     (43 observations deleted due to missingness)
   Multiple R-squared:  0.9191, Adjusted R-squared:  0.9092 
   F-statistic: 92.75 on 6 and 49 DF,  p-value: < 2.2e-16
anova(LM.dN)
   Analysis of Variance Table
   
   Response: d15NMet
             Df Sum Sq Mean Sq F value    Pr(>F)    
   TPGlx      1 815.78  815.78 466.510 < 2.2e-16 ***
   Location   5 157.40   31.48  18.003 4.248e-10 ***
   Residuals 49  85.69    1.75                      
   ---
   Signif. codes:  0 '***' 0.001 '**' 0.01 '*' 0.05 '.' 0.1 ' ' 1
coef.1 <- c(
  LM.dN$coefficients[1]
)
p3 <- anova(LM.dN)[1,5] 
R3 <- summary(LM.dN)$adj.r.squared

ggplot(data = data.reg.phenorm, aes(x = (TPGlx-1), y = d15NMet))+
  geom_point(aes(color=Location))+
  geom_smooth(method=lm, aes(y = predict(LM.dN, data.reg.phenorm), color=Location), lty=1, se=TRUE)+
  xlab(expression(TP[Glx-Phe]-1))+
  ylab(expression(delta^{15}*N[Met-Phe]*" (\u2030)"))+
  theme_light()+
  facet_wrap(~ Location)
   `geom_smooth()` using formula = 'y ~ x'
   Warning: Removed 3 rows containing non-finite outside the scale range
   (`stat_smooth()`).
   Warning: Removed 43 rows containing missing values or values outside the scale range
   (`geom_point()`).
```

```
noquote("Regressing d15N(Met-Phe) against TP(Ala-Phe)")
   [1] Regressing d15N(Met-Phe) against TP(Ala-Phe)
LM.dN = lm(d15NMet ~ (TPAla-1) + Location, data = data.reg.phenorm)
summary(LM.dN)
   
   Call:
   lm(formula = d15NMet ~ (TPAla - 1) + Location, data = data.reg.phenorm)
   
   Residuals:
       Min      1Q  Median      3Q     Max 
   -4.6077 -0.4499  0.1462  0.9234  3.6205 
   
   Coefficients:
                   Estimate Std. Error t value Pr(>|t|)    
   TPAla            1.58428    0.24105   6.572  2.8e-08 ***
   Location5 N      4.32584    1.11130   3.893 0.000294 ***
   Location8 N      4.39989    1.12143   3.923 0.000267 ***
   LocationALOHA S  0.08616    0.70264   0.123 0.902900    
   LocationALOHA W  0.14549    0.70541   0.206 0.837436    
   LocationOSP     -2.62893    0.80144  -3.280 0.001893 ** 
   ---
   Signif. codes:  0 '***' 0.001 '**' 0.01 '*' 0.05 '.' 0.1 ' ' 1
   
   Residual standard error: 1.521 on 50 degrees of freedom
     (42 observations deleted due to missingness)
   Multiple R-squared:  0.8909, Adjusted R-squared:  0.8778 
   F-statistic: 68.07 on 6 and 50 DF,  p-value: < 2.2e-16
anova(LM.dN)
   Analysis of Variance Table
   
   Response: d15NMet
             Df Sum Sq Mean Sq F value    Pr(>F)    
   TPAla      1 781.23  781.23  337.60 < 2.2e-16 ***
   Location   5 163.96   32.79   14.17 1.238e-08 ***
   Residuals 50 115.71    2.31                      
   ---
   Signif. codes:  0 '***' 0.001 '**' 0.01 '*' 0.05 '.' 0.1 ' ' 1
coef.2 <- c(
  LM.dN$coefficients[1]
)
p4 <- anova(LM.dN)[1,5] 
R4 <- summary(LM.dN)$adj.r.squared

ggplot(data = data.reg.phenorm, aes(x = (TPAla-1), y = d15NMet))+
  geom_point(aes(color=Location))+
  geom_smooth(method=lm, aes(y = predict(LM.dN, data.reg.phenorm), color=Location), lty=1, se=TRUE)+
  xlab(expression(TP[Ala-Phe]-1))+
  ylab(expression(delta^{15}*N[Met-Phe]*" (\u2030)"))+
  theme_light()+
  facet_wrap(~ Location)
   `geom_smooth()` using formula = 'y ~ x'
   Warning: Removed 2 rows containing non-finite outside the scale range
   (`stat_smooth()`).
   Warning: Removed 42 rows containing missing values or values outside the scale range
   (`geom_point()`).
```

```
DN.reg <- data.frame(
  "TP(Glx-Phe)" = coef.1,
  "TP(Ala-Phe)" = coef.2,
  row.names = "d15NMet"
)

DN.reg
           TP.Glx.Phe. TP.Ala.Phe.
   d15NMet    2.519698    1.584285
```

1. We tested to see if location had a significant effect on the slope of the regression. The P values associated with this test were 0.5568351 and 0.8267115 for TP(Glx-Phe) and TP(Ala-Phe), respectively. This indicates that location DOES NOT have a significant effect on the slope of the regression.
2. Removing the interaction with location, we tested to see if the slope is significant. The P value associated with these tests were 1.0849949^{-26} and 6.9791901^{-24}. This indicates that the slope of the regression IS significant.
3. The \(R^2\) values were 0.9091693 and 0.8778488, respectively.

## Regression for Glx

```
noquote("Checking to see if location affects the slope of the regression...")
   [1] Checking to see if location affects the slope of the regression...

noquote("Regressing d15N(Glx-Phe) against TP(Glx-Phe)")
   [1] Regressing d15N(Glx-Phe) against TP(Glx-Phe)
LM.dN = lm(d15NGlx ~ (TPGlx-1) * Location, data = data.reg.phenorm)
summary(LM.dN)
   Warning in summary.lm(LM.dN): essentially perfect fit: summary may be
   unreliable
   
   Call:
   lm(formula = d15NGlx ~ (TPGlx - 1) * Location, data = data.reg.phenorm)
   
   Residuals:
          Min         1Q     Median         3Q        Max 
   -2.522e-14 -1.052e-15 -4.010e-16  9.560e-16  4.730e-14 
   
   Coefficients:
                           Estimate Std. Error    t value Pr(>|t|)    
   TPGlx                  7.700e+00  2.665e-15  2.889e+15   <2e-16 ***
   Location5 N           -4.300e+00  6.596e-15 -6.519e+14   <2e-16 ***
   Location8 N           -4.300e+00  6.901e-15 -6.231e+14   <2e-16 ***
   LocationALOHA S       -4.300e+00  5.439e-15 -7.906e+14   <2e-16 ***
   LocationALOHA W       -4.300e+00  4.665e-15 -9.218e+14   <2e-16 ***
   LocationOSP           -4.300e+00  3.190e-15 -1.348e+15   <2e-16 ***
   TPGlx:Location8 N     -2.900e-15  3.822e-15 -7.590e-01    0.450    
   TPGlx:LocationALOHA S -1.598e-15  3.688e-15 -4.330e-01    0.666    
   TPGlx:LocationALOHA W -2.179e-15  3.418e-15 -6.370e-01    0.526    
   TPGlx:LocationOSP     -2.515e-15  3.061e-15 -8.220e-01    0.414    
   ---
   Signif. codes:  0 '***' 0.001 '**' 0.01 '*' 0.05 '.' 0.1 ' ' 1
   
   Residual standard error: 6.077e-15 on 85 degrees of freedom
     (3 observations deleted due to missingness)
   Multiple R-squared:      1,  Adjusted R-squared:      1 
   F-statistic: 4.422e+31 on 10 and 85 DF,  p-value: < 2.2e-16
anova(LM.dN)
   Warning in anova.lm(...): ANOVA F-tests on an essentially perfect fit are
   unreliable
   Analysis of Variance Table
   
   Response: d15NGlx
                  Df  Sum Sq Mean Sq    F value Pr(>F)    
   TPGlx           1 16170.5 16170.5 4.3789e+32 <2e-16 ***
   Location        5   158.5    31.7 8.5823e+29 <2e-16 ***
   TPGlx:Location  4     0.0     0.0 2.0450e-01 0.9352    
   Residuals      85     0.0     0.0                      
   ---
   Signif. codes:  0 '***' 0.001 '**' 0.01 '*' 0.05 '.' 0.1 ' ' 1
coef.1 <- c(
  LM.dN$coefficients[1],
  LM.dN$coefficients[1] + LM.dN$coefficients[7],
  LM.dN$coefficients[1] + LM.dN$coefficients[8],
  LM.dN$coefficients[1] + LM.dN$coefficients[9],
  LM.dN$coefficients[1] + LM.dN$coefficients[10]
)
p1 <- anova(LM.dN)[3,5] 
   Warning in anova.lm(...): ANOVA F-tests on an essentially perfect fit are
   unreliable
R1 <- summary(LM.dN)$adj.r.squared
   Warning in summary.lm(LM.dN): essentially perfect fit: summary may be
   unreliable

noquote("Regressing d15N(Glx-Phe) against TP(Ala-Phe)")
   [1] Regressing d15N(Glx-Phe) against TP(Ala-Phe)
LM.dN = lm(d15NGlx ~ (TPAla-1) * Location, data = data.reg.phenorm)
summary(LM.dN)
   
   Call:
   lm(formula = d15NGlx ~ (TPAla - 1) * Location, data = data.reg.phenorm)
   
   Residuals:
       Min      1Q  Median      3Q     Max 
   -3.4431 -0.6038 -0.0917  0.8062  2.8167 
   
   Coefficients:
                         Estimate Std. Error t value Pr(>|t|)    
   TPAla                   6.1352     0.4192  14.636   <2e-16 ***
   Location5 N            -2.7435     1.2000  -2.286   0.0247 *  
   Location8 N            -2.9875     1.2782  -2.337   0.0218 *  
   LocationALOHA S        -1.9852     0.9535  -2.082   0.0403 *  
   LocationALOHA W        -2.0020     0.8246  -2.428   0.0173 *  
   LocationOSP            -1.1141     0.5222  -2.133   0.0358 *  
   TPAla:Location8 N      -0.2106     0.5920  -0.356   0.7229    
   TPAla:LocationALOHA S  -0.9153     0.5470  -1.673   0.0979 .  
   TPAla:LocationALOHA W  -0.7394     0.5190  -1.425   0.1579    
   TPAla:LocationOSP      -1.1635     0.4626  -2.515   0.0138 *  
   ---
   Signif. codes:  0 '***' 0.001 '**' 0.01 '*' 0.05 '.' 0.1 ' ' 1
   
   Residual standard error: 1.191 on 85 degrees of freedom
     (3 observations deleted due to missingness)
   Multiple R-squared:  0.9926, Adjusted R-squared:  0.9917 
   F-statistic:  1142 on 10 and 85 DF,  p-value: < 2.2e-16
anova(LM.dN)
   Analysis of Variance Table
   
   Response: d15NGlx
                  Df  Sum Sq Mean Sq    F value    Pr(>F)    
   TPAla           1 16133.8 16133.8 11372.0879 < 2.2e-16 ***
   Location        5    61.5    12.3     8.6765 1.119e-06 ***
   TPAla:Location  4    13.0     3.3     2.2925   0.06605 .  
   Residuals      85   120.6     1.4                         
   ---
   Signif. codes:  0 '***' 0.001 '**' 0.01 '*' 0.05 '.' 0.1 ' ' 1
coef.2 <- c(
  LM.dN$coefficients[1],
  LM.dN$coefficients[1] + LM.dN$coefficients[7],
  LM.dN$coefficients[1] + LM.dN$coefficients[8],
  LM.dN$coefficients[1] + LM.dN$coefficients[9],
  LM.dN$coefficients[1] + LM.dN$coefficients[10]
)
p2 <- anova(LM.dN)[3,5] 
R2 <- summary(LM.dN)$adj.r.squared


noquote("Now re-running the regression without location interaction")
   [1] Now re-running the regression without location interaction

noquote("Regressing d15N(Glx-Phe) against TP(Glx-Phe)")
   [1] Regressing d15N(Glx-Phe) against TP(Glx-Phe)
LM.dN = lm(d15NGlx ~ (TPGlx-1) + Location, data = data.reg.phenorm)
summary(LM.dN)
   Warning in summary.lm(LM.dN): essentially perfect fit: summary may be
   unreliable
   
   Call:
   lm(formula = d15NGlx ~ (TPGlx - 1) + Location, data = data.reg.phenorm)
   
   Residuals:
          Min         1Q     Median         3Q        Max 
   -2.347e-14 -1.052e-15 -1.720e-16  9.550e-16  4.798e-14 
   
   Coefficients:
                     Estimate Std. Error    t value Pr(>|t|)    
   TPGlx            7.700e+00  9.417e-16  8.177e+15   <2e-16 ***
   Location5 N     -4.300e+00  2.921e-15 -1.472e+15   <2e-16 ***
   Location8 N     -4.300e+00  2.869e-15 -1.499e+15   <2e-16 ***
   LocationALOHA S -4.300e+00  2.416e-15 -1.780e+15   <2e-16 ***
   LocationALOHA W -4.300e+00  2.390e-15 -1.799e+15   <2e-16 ***
   LocationOSP     -4.300e+00  2.134e-15 -2.016e+15   <2e-16 ***
   ---
   Signif. codes:  0 '***' 0.001 '**' 0.01 '*' 0.05 '.' 0.1 ' ' 1
   
   Residual standard error: 5.967e-15 on 89 degrees of freedom
     (3 observations deleted due to missingness)
   Multiple R-squared:      1,  Adjusted R-squared:      1 
   F-statistic: 7.643e+31 on 6 and 89 DF,  p-value: < 2.2e-16
anova(LM.dN)
   Warning in anova.lm(...): ANOVA F-tests on an essentially perfect fit are
   unreliable
   Analysis of Variance Table
   
   Response: d15NGlx
             Df  Sum Sq Mean Sq    F value    Pr(>F)    
   TPGlx      1 16170.5 16170.5 4.5413e+32 < 2.2e-16 ***
   Location   5   158.5    31.7 8.9005e+29 < 2.2e-16 ***
   Residuals 89     0.0     0.0                         
   ---
   Signif. codes:  0 '***' 0.001 '**' 0.01 '*' 0.05 '.' 0.1 ' ' 1
coef.1 <- c(
  LM.dN$coefficients[1]
)
p3 <- anova(LM.dN)[1,5] 
   Warning in anova.lm(...): ANOVA F-tests on an essentially perfect fit are
   unreliable
R3 <- summary(LM.dN)$adj.r.squared
   Warning in summary.lm(LM.dN): essentially perfect fit: summary may be
   unreliable

ggplot(data = data.reg.phenorm, aes(x = (TPGlx-1), y = d15NGlx))+
  geom_point(aes(color=Location))+
  geom_smooth(method=lm, aes(y = predict(LM.dN, data.reg.phenorm), color=Location), lty=1, se=TRUE)+
  xlab(expression(TP[Glx-Phe]-1))+
  ylab(expression(delta^{15}*N[Glx-Phe]*" (\u2030)"))+
  theme_light()+
  facet_wrap(~ Location)
   `geom_smooth()` using formula = 'y ~ x'
   Warning: Removed 3 rows containing non-finite outside the scale range
   (`stat_smooth()`).
   Warning: Removed 3 rows containing missing values or values outside the scale range
   (`geom_point()`).
```

```
noquote("Regressing d15N(Glx-Phe) against TP(Ala-Phe)")
   [1] Regressing d15N(Glx-Phe) against TP(Ala-Phe)
LM.dN = lm(d15NGlx ~ (TPAla-1) + Location, data = data.reg.phenorm)
summary(LM.dN)
   
   Call:
   lm(formula = d15NGlx ~ (TPAla - 1) + Location, data = data.reg.phenorm)
   
   Residuals:
       Min      1Q  Median      3Q     Max 
   -3.3377 -0.7749 -0.1058  0.8535  2.8438 
   
   Coefficients:
                   Estimate Std. Error t value Pr(>|t|)    
   TPAla             5.3022     0.1370  38.689  < 2e-16 ***
   Location5 N      -0.4794     0.5375  -0.892 0.374799    
   Location8 N      -1.1541     0.5367  -2.150 0.034227 *  
   LocationALOHA S  -2.1978     0.4625  -4.752 7.69e-06 ***
   LocationALOHA W  -1.7638     0.4477  -3.939 0.000162 ***
   LocationOSP      -1.9320     0.3945  -4.898 4.31e-06 ***
   ---
   Signif. codes:  0 '***' 0.001 '**' 0.01 '*' 0.05 '.' 0.1 ' ' 1
   
   Residual standard error: 1.225 on 89 degrees of freedom
     (3 observations deleted due to missingness)
   Multiple R-squared:  0.9918, Adjusted R-squared:  0.9913 
   F-statistic:  1798 on 6 and 89 DF,  p-value: < 2.2e-16
anova(LM.dN)
   Analysis of Variance Table
   
   Response: d15NGlx
             Df  Sum Sq Mean Sq    F value    Pr(>F)    
   TPAla      1 16133.8 16133.8 10747.7699 < 2.2e-16 ***
   Location   5    61.5    12.3     8.2001 2.105e-06 ***
   Residuals 89   133.6     1.5                         
   ---
   Signif. codes:  0 '***' 0.001 '**' 0.01 '*' 0.05 '.' 0.1 ' ' 1
coef.2 <- c(
  LM.dN$coefficients[1]
)
p4 <- anova(LM.dN)[1,5] 
R4 <- summary(LM.dN)$adj.r.squared

ggplot(data = data.reg.phenorm, aes(x = (TPAla-1), y = d15NGlx))+
  geom_point(aes(color=Location))+
  geom_smooth(method=lm, aes(y = predict(LM.dN, data.reg.phenorm), color=Location), lty=1, se=TRUE)+
  xlab(expression(TP[Ala-Phe]-1))+
  ylab(expression(delta^{15}*N[Glx-Phe]*" (\u2030)"))+
  theme_light()+
  facet_wrap(~ Location)
   `geom_smooth()` using formula = 'y ~ x'
   Warning: Removed 2 rows containing non-finite outside the scale range (`stat_smooth()`).
   Removed 3 rows containing missing values or values outside the scale range
   (`geom_point()`).
```

```
DN.reg <- data.frame(
  "TP(Glx-Phe)" = coef.1,
  "TP(Ala-Phe)" = coef.2,
  row.names = "d15NGlx"
)

DN.reg
           TP.Glx.Phe. TP.Ala.Phe.
   d15NGlx         7.7    5.302195
```

1. We tested to see if location had a significant effect on the slope of the regression. The P values associated with this test were 0.9352411 and 0.0660517 for TP(Glx-Phe) and TP(Ala-Phe), respectively. This indicates that location DOES NOT have a significant effect on the slope of the regression.
2. Removing the interaction with location, we tested to see if the slope is significant. The P value associated with these tests were 0 and 1.3261366^{-94}. This indicates that the slope of the regression IS significant.
3. The \(R^2\) values were 1 and 0.9912666, respectively.

## Regression for Phe

We note that for Phe we can obviously not normalize to Phe, so we are regressing the raw value.

```
noquote("Checking to see if location affects the slope of the regression...")
   [1] Checking to see if location affects the slope of the regression...

noquote("Regressing d15N(Phe) against TP(Glx-Phe)")
   [1] Regressing d15N(Phe) against TP(Glx-Phe)
LM.dN = lm(d15NPhe ~ (TPGlx-1) * Location, data = data.reg)
summary(LM.dN)
   
   Call:
   lm(formula = d15NPhe ~ (TPGlx - 1) * Location, data = data.reg)
   
   Residuals:
       Min      1Q  Median      3Q     Max 
   -3.3993 -0.9049 -0.0397  0.8101  5.6727 
   
   Coefficients:
                         Estimate Std. Error t value Pr(>|t|)   
   TPGlx                   0.1161     0.7048   0.165  0.86956   
   Location5 N             3.6999     1.7446   2.121  0.03685 * 
   Location8 N             4.6155     1.8253   2.529  0.01330 * 
   LocationALOHA S        -1.9322     1.4385  -1.343  0.18278   
   LocationALOHA W         0.6794     1.2337   0.551  0.58331   
   LocationOSP             2.4885     0.8438   2.949  0.00412 **
   TPGlx:Location8 N      -0.7391     1.0108  -0.731  0.46666   
   TPGlx:LocationALOHA S  -0.3804     0.9754  -0.390  0.69749   
   TPGlx:LocationALOHA W  -1.8194     0.9039  -2.013  0.04730 * 
   TPGlx:LocationOSP      -1.0520     0.8096  -1.299  0.19734   
   ---
   Signif. codes:  0 '***' 0.001 '**' 0.01 '*' 0.05 '.' 0.1 ' ' 1
   
   Residual standard error: 1.607 on 85 degrees of freedom
     (3 observations deleted due to missingness)
   Multiple R-squared:  0.7277, Adjusted R-squared:  0.6957 
   F-statistic: 22.71 on 10 and 85 DF,  p-value: < 2.2e-16
anova(LM.dN)
   Analysis of Variance Table
   
   Response: d15NPhe
                  Df Sum Sq Mean Sq F value Pr(>F)    
   TPGlx           1   0.02   0.017  0.0065 0.9358    
   Location        5 573.73 114.747 44.4195 <2e-16 ***
   TPGlx:Location  4  13.01   3.254  1.2595 0.2923    
   Residuals      85 219.58   2.583                   
   ---
   Signif. codes:  0 '***' 0.001 '**' 0.01 '*' 0.05 '.' 0.1 ' ' 1
coef.1 <- c(
  LM.dN$coefficients[1],
  LM.dN$coefficients[1] + LM.dN$coefficients[7],
  LM.dN$coefficients[1] + LM.dN$coefficients[8],
  LM.dN$coefficients[1] + LM.dN$coefficients[9],
  LM.dN$coefficients[1] + LM.dN$coefficients[10]
)
p1 <- anova(LM.dN)[3,5] 
R1 <- summary(LM.dN)$adj.r.squared

noquote("Regressing d15N(Phe) against TP(Ala-Phe)")
   [1] Regressing d15N(Phe) against TP(Ala-Phe)
LM.dN = lm(d15NPhe ~ (TPAla-1) * Location, data = data.reg)
summary(LM.dN)
   
   Call:
   lm(formula = d15NPhe ~ (TPAla - 1) * Location, data = data.reg)
   
   Residuals:
       Min      1Q  Median      3Q     Max 
   -3.5691 -0.9549 -0.0723  0.8374  5.6520 
   
   Coefficients:
                         Estimate Std. Error t value Pr(>|t|)  
   TPAla                  0.05741    0.57682   0.100   0.9210  
   Location5 N            3.81879    1.65120   2.313   0.0231 *
   Location8 N            3.91663    1.75884   2.227   0.0286 *
   LocationALOHA S       -1.59929    1.18656  -1.348   0.1813  
   LocationALOHA W       -0.06079    1.13467  -0.054   0.9574  
   LocationOSP            1.80201    0.71855   2.508   0.0140 *
   TPAla:Location8 N     -0.33555    0.81455  -0.412   0.6814  
   TPAla:LocationALOHA S -0.38896    0.73008  -0.533   0.5956  
   TPAla:LocationALOHA W -1.15983    0.71409  -1.624   0.1080  
   TPAla:LocationOSP     -0.54078    0.63652  -0.850   0.3979  
   ---
   Signif. codes:  0 '***' 0.001 '**' 0.01 '*' 0.05 '.' 0.1 ' ' 1
   
   Residual standard error: 1.639 on 86 degrees of freedom
     (2 observations deleted due to missingness)
   Multiple R-squared:  0.7145, Adjusted R-squared:  0.6813 
   F-statistic: 21.52 on 10 and 86 DF,  p-value: < 2.2e-16
anova(LM.dN)
   Analysis of Variance Table
   
   Response: d15NPhe
                  Df Sum Sq Mean Sq F value Pr(>F)    
   TPAla           1   0.35   0.353  0.1315 0.7178    
   Location        5 568.97 113.794 42.3625 <2e-16 ***
   TPAla:Location  4   8.80   2.200  0.8190 0.5165    
   Residuals      86 231.01   2.686                   
   ---
   Signif. codes:  0 '***' 0.001 '**' 0.01 '*' 0.05 '.' 0.1 ' ' 1
coef.2 <- c(
  LM.dN$coefficients[1],
  LM.dN$coefficients[1] + LM.dN$coefficients[7],
  LM.dN$coefficients[1] + LM.dN$coefficients[8],
  LM.dN$coefficients[1] + LM.dN$coefficients[9],
  LM.dN$coefficients[1] + LM.dN$coefficients[10]
)
p2 <- anova(LM.dN)[3,5] 
R2 <- summary(LM.dN)$adj.r.squared


noquote("Now re-running the regression without location interaction")
   [1] Now re-running the regression without location interaction

noquote("Regressing d15N(Phe) against TP(Glx-Phe)")
   [1] Regressing d15N(Phe) against TP(Glx-Phe)
LM.dN = lm(d15NPhe ~ (TPGlx-1) + Location, data = data.reg)
summary(LM.dN)
   
   Call:
   lm(formula = d15NPhe ~ (TPGlx - 1) + Location, data = data.reg)
   
   Residuals:
       Min      1Q  Median      3Q     Max 
   -3.5030 -1.0198 -0.1133  0.8822  5.3110 
   
   Coefficients:
                   Estimate Std. Error t value Pr(>|t|)    
   TPGlx            -0.8204     0.2551  -3.216 0.001814 ** 
   Location5 N       5.9174     0.7913   7.478 5.06e-11 ***
   Location8 N       5.0966     0.7773   6.556 3.50e-09 ***
   LocationALOHA S  -0.7901     0.6544  -1.207 0.230477    
   LocationALOHA W  -1.1574     0.6475  -1.787 0.077269 .  
   LocationOSP       2.2562     0.5780   3.904 0.000184 ***
   ---
   Signif. codes:  0 '***' 0.001 '**' 0.01 '*' 0.05 '.' 0.1 ' ' 1
   
   Residual standard error: 1.617 on 89 degrees of freedom
     (3 observations deleted due to missingness)
   Multiple R-squared:  0.7115, Adjusted R-squared:  0.6921 
   F-statistic: 36.59 on 6 and 89 DF,  p-value: < 2.2e-16
anova(LM.dN)
   Analysis of Variance Table
   
   Response: d15NPhe
             Df Sum Sq Mean Sq F value Pr(>F)    
   TPGlx      1   0.02   0.017  0.0064 0.9362    
   Location   5 573.73 114.747 43.9074 <2e-16 ***
   Residuals 89 232.59   2.613                   
   ---
   Signif. codes:  0 '***' 0.001 '**' 0.01 '*' 0.05 '.' 0.1 ' ' 1
coef.1 <- c(
  LM.dN$coefficients[1]
)
p3 <- anova(LM.dN)[1,5] 
R3 <- summary(LM.dN)$adj.r.squared

ggplot(data = data.reg, aes(x = (TPGlx-1), y = d15NPhe))+
  geom_point(aes(color=Location))+
  geom_smooth(method=lm, aes(y = predict(LM.dN, data.reg), color=Location), lty=1, se=TRUE)+
  xlab(expression(TP[Glx-Phe]-1))+
  ylab(expression(delta^{15}*N[Phe]*" (\u2030)"))+
  theme_light()+
  facet_wrap(~ Location)
   `geom_smooth()` using formula = 'y ~ x'
   Warning: Removed 3 rows containing non-finite outside the scale range
   (`stat_smooth()`).
   Warning: Removed 3 rows containing missing values or values outside the scale range
   (`geom_point()`).
```

```
noquote("Regressing d15N(Phe) against TP(Ala-Phe)")
   [1] Regressing d15N(Phe) against TP(Ala-Phe)
LM.dN = lm(d15NPhe ~ (TPAla-1) + Location, data = data.reg)
summary(LM.dN)
   
   Call:
   lm(formula = d15NPhe ~ (TPAla - 1) + Location, data = data.reg)
   
   Residuals:
       Min      1Q  Median      3Q     Max 
   -3.5468 -0.9855 -0.1114  0.9168  5.4174 
   
   Coefficients:
                   Estimate Std. Error t value Pr(>|t|)    
   TPAla            -0.4994     0.1804  -2.768 0.006847 ** 
   Location5 N       5.3323     0.7120   7.489 4.55e-11 ***
   Location8 N       4.5685     0.7103   6.432 5.93e-09 ***
   LocationALOHA S  -1.1784     0.5938  -1.984 0.050246 .  
   LocationALOHA W  -1.5943     0.5923  -2.692 0.008475 ** 
   LocationOSP       1.8418     0.5210   3.535 0.000646 ***
   ---
   Signif. codes:  0 '***' 0.001 '**' 0.01 '*' 0.05 '.' 0.1 ' ' 1
   
   Residual standard error: 1.632 on 90 degrees of freedom
     (2 observations deleted due to missingness)
   Multiple R-squared:  0.7036, Adjusted R-squared:  0.6839 
   F-statistic: 35.61 on 6 and 90 DF,  p-value: < 2.2e-16
anova(LM.dN)
   Analysis of Variance Table
   
   Response: d15NPhe
             Df Sum Sq Mean Sq F value Pr(>F)    
   TPAla      1   0.35   0.353  0.1325 0.7167    
   Location   5 568.97 113.794 42.7061 <2e-16 ***
   Residuals 90 239.81   2.665                   
   ---
   Signif. codes:  0 '***' 0.001 '**' 0.01 '*' 0.05 '.' 0.1 ' ' 1
coef.2 <- c(
  LM.dN$coefficients[1]
)
p4 <- anova(LM.dN)[1,5] 
R4 <- summary(LM.dN)$adj.r.squared

ggplot(data = data.reg, aes(x = (TPAla-1), y = d15NPhe))+
  geom_point(aes(color=Location))+
  geom_smooth(method=lm, aes(y = predict(LM.dN, data.reg), color=Location), lty=1, se=TRUE)+
  xlab(expression(TP[Ala-Phe]-1))+
  ylab(expression(delta^{15}*N[Phe]*" (\u2030)"))+
  theme_light()+
  facet_wrap(~ Location)
   `geom_smooth()` using formula = 'y ~ x'
   Warning: Removed 2 rows containing non-finite outside the scale range
   (`stat_smooth()`).
   Warning: Removed 2 rows containing missing values or values outside the scale range
   (`geom_point()`).
```

```
DN.reg <- data.frame(
  "TP(Glx-Phe)" = coef.1,
  "TP(Ala-Phe)" = coef.2,
  row.names = "d15NPhe"
)

DN.reg
           TP.Glx.Phe. TP.Ala.Phe.
   d15NPhe  -0.8204298  -0.4994384
```

1. We tested to see if location had a significant effect on the slope of the regression. The P values associated with this test were 0.2923311 and 0.516532 for TP(Glx-Phe) and TP(Ala-Phe), respectively. This indicates that location DOES NOT have a significant effect.
2. Removing the interaction with location, we tested to see if the slope is significant. The P value associated with these tests were 0.9361778 and 0.7166581. This indicates that the slope of the regression IS NOT significant.
3. The \(R^2\) values were 0.6921018 and 0.6838599, respectively.

## Regression for Tyr

```
noquote("Checking to see if location affects the slope of the regression...")
   [1] Checking to see if location affects the slope of the regression...

noquote("Regressing d15N(Tyr-Phe) against TP(Glx-Phe)")
   [1] Regressing d15N(Tyr-Phe) against TP(Glx-Phe)
LM.dN = lm(d15NTyr ~ (TPGlx-1) * Location, data = data.reg.phenorm)
summary(LM.dN)
   
   Call:
   lm(formula = d15NTyr ~ (TPGlx - 1) * Location, data = data.reg.phenorm)
   
   Residuals:
        Min       1Q   Median       3Q      Max 
   -10.3449  -0.3366   0.3664   1.0459   3.2987 
   
   Coefficients:
                         Estimate Std. Error t value Pr(>|t|)
   TPGlx                   1.3165     0.9707   1.356    0.179
   Location5 N             0.2407     2.4026   0.100    0.920
   Location8 N            -0.2337     2.5138  -0.093    0.926
   LocationALOHA S        -1.2553     2.0352  -0.617    0.539
   LocationALOHA W        -2.3390     1.7188  -1.361    0.178
   LocationOSP            -0.8648     1.1621  -0.744    0.459
   TPGlx:Location8 N       0.3140     1.3920   0.226    0.822
   TPGlx:LocationALOHA S   1.1684     1.3836   0.844    0.401
   TPGlx:LocationALOHA W   1.8090     1.2687   1.426    0.158
   TPGlx:LocationOSP      -0.1534     1.1150  -0.138    0.891
   
   Residual standard error: 2.213 on 75 degrees of freedom
     (13 observations deleted due to missingness)
   Multiple R-squared:  0.7032, Adjusted R-squared:  0.6636 
   F-statistic: 17.77 on 10 and 75 DF,  p-value: 4.484e-16
anova(LM.dN)
   Analysis of Variance Table
   
   Response: d15NTyr
                  Df Sum Sq Mean Sq  F value    Pr(>F)    
   TPGlx           1 750.08  750.08 153.0931 < 2.2e-16 ***
   Location        5  97.37   19.47   3.9748  0.002981 ** 
   TPGlx:Location  4  23.22    5.80   1.1846  0.324537    
   Residuals      75 367.46    4.90                       
   ---
   Signif. codes:  0 '***' 0.001 '**' 0.01 '*' 0.05 '.' 0.1 ' ' 1
coef.1 <- c(
  LM.dN$coefficients[1],
  LM.dN$coefficients[1] + LM.dN$coefficients[7],
  LM.dN$coefficients[1] + LM.dN$coefficients[8],
  LM.dN$coefficients[1] + LM.dN$coefficients[9],
  LM.dN$coefficients[1] + LM.dN$coefficients[10]
)
p1 <- anova(LM.dN)[3,5] 
R1 <- summary(LM.dN)$adj.r.squared

noquote("Regressing d15N(Tyr-Phe) against TP(Ala-Phe)")
   [1] Regressing d15N(Tyr-Phe) against TP(Ala-Phe)
LM.dN = lm(d15NTyr ~ (TPAla-1) * Location, data = data.reg.phenorm)
summary(LM.dN)
   
   Call:
   lm(formula = d15NTyr ~ (TPAla - 1) * Location, data = data.reg.phenorm)
   
   Residuals:
        Min       1Q   Median       3Q      Max 
   -10.4288  -0.4106   0.4505   1.0584   3.5330 
   
   Coefficients:
                         Estimate Std. Error t value Pr(>|t|)
   TPAla                  1.00750    0.79371   1.269    0.208
   Location5 N            0.61943    2.27208   0.273    0.786
   Location8 N            0.48657    2.42020   0.201    0.841
   LocationALOHA S       -0.72060    1.70384  -0.423    0.674
   LocationALOHA W       -1.69620    1.63946  -1.035    0.304
   LocationOSP           -0.05925    0.98873  -0.060    0.952
   TPAla:Location8 N      0.09682    1.12084   0.086    0.931
   TPAla:LocationALOHA S  0.78156    1.04862   0.745    0.458
   TPAla:LocationALOHA W  1.37825    1.03075   1.337    0.185
   TPAla:LocationOSP     -0.38754    0.87586  -0.442    0.659
   
   Residual standard error: 2.255 on 76 degrees of freedom
     (12 observations deleted due to missingness)
   Multiple R-squared:  0.6881, Adjusted R-squared:  0.647 
   F-statistic: 16.76 on 10 and 76 DF,  p-value: 1.562e-15
anova(LM.dN)
   Analysis of Variance Table
   
   Response: d15NTyr
                  Df Sum Sq Mean Sq  F value    Pr(>F)    
   TPAla           1 724.89  724.89 142.5243 < 2.2e-16 ***
   Location        5  94.81   18.96   3.7281  0.004516 ** 
   TPAla:Location  4  32.98    8.25   1.6211  0.177601    
   Residuals      76 386.54    5.09                       
   ---
   Signif. codes:  0 '***' 0.001 '**' 0.01 '*' 0.05 '.' 0.1 ' ' 1
coef.2 <- c(
  LM.dN$coefficients[1],
  LM.dN$coefficients[1] + LM.dN$coefficients[7],
  LM.dN$coefficients[1] + LM.dN$coefficients[8],
  LM.dN$coefficients[1] + LM.dN$coefficients[9],
  LM.dN$coefficients[1] + LM.dN$coefficients[10]
)
p2 <- anova(LM.dN)[3,5] 
R2 <- summary(LM.dN)$adj.r.squared


noquote("Now re-running the regression without location interaction")
   [1] Now re-running the regression without location interaction

noquote("Regressing d15N(Tyr-Phe) against TP(Glx-Phe)")
   [1] Regressing d15N(Tyr-Phe) against TP(Glx-Phe)
LM.dN = lm(d15NTyr ~ (TPGlx-1) + Location, data = data.reg.phenorm)
summary(LM.dN)
   
   Call:
   lm(formula = d15NTyr ~ (TPGlx - 1) + Location, data = data.reg.phenorm)
   
   Residuals:
        Min       1Q   Median       3Q      Max 
   -10.1635  -0.6921   0.3425   1.2504   3.0441 
   
   Coefficients:
                   Estimate Std. Error t value Pr(>|t|)    
   TPGlx             1.7862     0.3571   5.002 3.36e-06 ***
   Location5 N      -0.8714     1.0997  -0.792   0.4305    
   Location8 N      -0.6132     1.0813  -0.567   0.5723    
   LocationALOHA S   0.1141     0.9497   0.120   0.9047    
   LocationALOHA W   0.3066     0.9224   0.332   0.7404    
   LocationOSP      -2.1183     0.8061  -2.628   0.0103 *  
   ---
   Signif. codes:  0 '***' 0.001 '**' 0.01 '*' 0.05 '.' 0.1 ' ' 1
   
   Residual standard error: 2.224 on 79 degrees of freedom
     (13 observations deleted due to missingness)
   Multiple R-squared:  0.6845, Adjusted R-squared:  0.6605 
   F-statistic: 28.56 on 6 and 79 DF,  p-value: < 2.2e-16
anova(LM.dN)
   Analysis of Variance Table
   
   Response: d15NTyr
             Df Sum Sq Mean Sq F value    Pr(>F)    
   TPGlx      1 750.08  750.08 151.675 < 2.2e-16 ***
   Location   5  97.37   19.47   3.938  0.003067 ** 
   Residuals 79 390.68    4.95                      
   ---
   Signif. codes:  0 '***' 0.001 '**' 0.01 '*' 0.05 '.' 0.1 ' ' 1
coef.1 <- c(
  LM.dN$coefficients[1]
)
p3 <- anova(LM.dN)[1,5] 
R3 <- summary(LM.dN)$adj.r.squared

ggplot(data = data.reg.phenorm, aes(x = (TPGlx-1), y = d15NTyr))+
  geom_point(aes(color=Location))+
  geom_smooth(method=lm, aes(y = predict(LM.dN, data.reg.phenorm), color=Location), lty=1, se=TRUE)+
  xlab(expression(TP[Glx-Phe]-1))+
  ylab(expression(delta^{15}*N[Tyr-Phe]*" (\u2030)"))+
  theme_light()+
  facet_wrap(~ Location)
   `geom_smooth()` using formula = 'y ~ x'
   Warning: Removed 3 rows containing non-finite outside the scale range
   (`stat_smooth()`).
   Warning: Removed 13 rows containing missing values or values outside the scale range
   (`geom_point()`).
```

```
noquote("Regressing d15N(Tyr-Phe) against TP(Ala-Phe)")
   [1] Regressing d15N(Tyr-Phe) against TP(Ala-Phe)
LM.dN = lm(d15NTyr ~ (TPAla-1) + Location, data = data.reg.phenorm)
summary(LM.dN)
   
   Call:
   lm(formula = d15NTyr ~ (TPAla - 1) + Location, data = data.reg.phenorm)
   
   Residuals:
        Min       1Q   Median       3Q      Max 
   -10.3672  -0.7329   0.4131   1.3263   2.8997 
   
   Coefficients:
                   Estimate Std. Error t value Pr(>|t|)    
   TPAla             1.1537     0.2629   4.388 3.46e-05 ***
   Location5 N       0.2222     1.0174   0.218   0.8277    
   Location8 N       0.3413     1.0182   0.335   0.7384    
   LocationALOHA S   0.7489     0.8793   0.852   0.3969    
   LocationALOHA W   1.1603     0.8638   1.343   0.1830    
   LocationOSP      -1.3801     0.7517  -1.836   0.0701 .  
   ---
   Signif. codes:  0 '***' 0.001 '**' 0.01 '*' 0.05 '.' 0.1 ' ' 1
   
   Residual standard error: 2.29 on 80 degrees of freedom
     (12 observations deleted due to missingness)
   Multiple R-squared:  0.6615, Adjusted R-squared:  0.6361 
   F-statistic: 26.05 on 6 and 80 DF,  p-value: < 2.2e-16
anova(LM.dN)
   Analysis of Variance Table
   
   Response: d15NTyr
             Df Sum Sq Mean Sq  F value    Pr(>F)    
   TPAla      1 724.89  724.89 138.2313 < 2.2e-16 ***
   Location   5  94.81   18.96   3.6158  0.005329 ** 
   Residuals 80 419.52    5.24                       
   ---
   Signif. codes:  0 '***' 0.001 '**' 0.01 '*' 0.05 '.' 0.1 ' ' 1
coef.2 <- c(
  LM.dN$coefficients[1]
)
p4 <- anova(LM.dN)[1,5] 
R4 <- summary(LM.dN)$adj.r.squared

ggplot(data = data.reg.phenorm, aes(x = (TPAla-1), y = d15NTyr))+
  geom_point(aes(color=Location))+
  geom_smooth(method=lm, aes(y = predict(LM.dN, data.reg.phenorm), color=Location), lty=1, se=TRUE)+
  xlab(expression(TP[Ala-Phe]-1))+
  ylab(expression(delta^{15}*N[Tyr-Phe]*" (\u2030)"))+
  theme_light()+
  facet_wrap(~ Location)
   `geom_smooth()` using formula = 'y ~ x'
   Warning: Removed 2 rows containing non-finite outside the scale range
   (`stat_smooth()`).
   Warning: Removed 12 rows containing missing values or values outside the scale range
   (`geom_point()`).
```

```
DN.reg <- data.frame(
  "TP(Glx-Phe)" = coef.1,
  "TP(Ala-Phe)" = coef.2,
  row.names = "d15NTyr"
)

DN.reg
           TP.Glx.Phe. TP.Ala.Phe.
   d15NTyr    1.786172    1.153652
```

1. We tested to see if location had a significant effect on the slope of the regression. The P values associated with this test were 0.3245371 and 0.177601 for TP(Glx-Phe) and TP(Ala-Phe), respectively. This indicates that location DOES NOT have a significant effect on the slope of the regression.
2. Removing the interaction with location, we tested to see if the slope is significant. The P value associated with these tests were 4.5473247^{-20} and 4.0935218^{-19}. This indicates that the slope of the regression IS significant.
3. The \(R^2\) values were 0.6604962 and 0.6360717, respectively.

## Regression for Lys

```
noquote("Checking to see if location affects the slope of the regression...")
   [1] Checking to see if location affects the slope of the regression...

noquote("Regressing d15N(Lys-Phe) against TP(Glx-Phe)")
   [1] Regressing d15N(Lys-Phe) against TP(Glx-Phe)
LM.dN = lm(d15NLys ~ (TPGlx-1) * Location, data = data.reg.phenorm)
summary(LM.dN)
   
   Call:
   lm(formula = d15NLys ~ (TPGlx - 1) * Location, data = data.reg.phenorm)
   
   Residuals:
       Min      1Q  Median      3Q     Max 
   -5.2454 -0.8785 -0.0010  1.1130  4.6034 
   
   Coefficients:
                         Estimate Std. Error t value Pr(>|t|)  
   TPGlx                   0.7590     0.7858   0.966   0.3368  
   Location5 N            -1.5017     1.9450  -0.772   0.4422  
   Location8 N            -2.7898     2.0350  -1.371   0.1740  
   LocationALOHA S        -1.7898     1.6037  -1.116   0.2676  
   LocationALOHA W        -2.3717     1.3755  -1.724   0.0883 .
   LocationOSP            -2.4734     0.9407  -2.629   0.0102 *
   TPGlx:Location8 N       0.2948     1.1269   0.262   0.7943  
   TPGlx:LocationALOHA S   0.8136     1.0874   0.748   0.4564  
   TPGlx:LocationALOHA W   1.3233     1.0077   1.313   0.1927  
   TPGlx:LocationOSP       0.4815     0.9026   0.533   0.5951  
   ---
   Signif. codes:  0 '***' 0.001 '**' 0.01 '*' 0.05 '.' 0.1 ' ' 1
   
   Residual standard error: 1.792 on 85 degrees of freedom
     (3 observations deleted due to missingness)
   Multiple R-squared:  0.4134, Adjusted R-squared:  0.3444 
   F-statistic:  5.99 on 10 and 85 DF,  p-value: 8.14e-07
anova(LM.dN)
   Analysis of Variance Table
   
   Response: d15NLys
                  Df  Sum Sq Mean Sq F value    Pr(>F)    
   TPGlx           1  72.657  72.657 22.6286 7.959e-06 ***
   Location        5 112.643  22.529  7.0165 1.565e-05 ***
   TPGlx:Location  4   7.032   1.758  0.5476    0.7013    
   Residuals      85 272.921   3.211                      
   ---
   Signif. codes:  0 '***' 0.001 '**' 0.01 '*' 0.05 '.' 0.1 ' ' 1
coef.1 <- c(
  LM.dN$coefficients[1],
  LM.dN$coefficients[1] + LM.dN$coefficients[7],
  LM.dN$coefficients[1] + LM.dN$coefficients[8],
  LM.dN$coefficients[1] + LM.dN$coefficients[9],
  LM.dN$coefficients[1] + LM.dN$coefficients[10]
)
p1 <- anova(LM.dN)[3,5] 
R1 <- summary(LM.dN)$adj.r.squared

noquote("Regressing d15N(Lys-Phe) against TP(Ala-Phe)")
   [1] Regressing d15N(Lys-Phe) against TP(Ala-Phe)
LM.dN = lm(d15NLys ~ (TPAla-1) * Location, data = data.reg.phenorm)
summary(LM.dN)
   
   Call:
   lm(formula = d15NLys ~ (TPAla - 1) * Location, data = data.reg.phenorm)
   
   Residuals:
       Min      1Q  Median      3Q     Max 
   -5.2433 -0.8389  0.1024  1.0778  4.6606 
   
   Coefficients:
                         Estimate Std. Error t value Pr(>|t|)  
   TPAla                  0.70059    0.63095   1.110   0.2699  
   Location5 N           -1.60863    1.80615  -0.891   0.3756  
   Location8 N           -2.47335    1.92389  -1.286   0.2020  
   LocationALOHA S       -1.51379    1.29791  -1.166   0.2467  
   LocationALOHA W       -1.72804    1.24115  -1.392   0.1674  
   LocationOSP           -1.88174    0.78597  -2.394   0.0188 *
   TPAla:Location8 N      0.06382    0.89099   0.072   0.9431  
   TPAla:LocationALOHA S  0.45183    0.79859   0.566   0.5730  
   TPAla:LocationALOHA W  0.74992    0.78110   0.960   0.3397  
   TPAla:LocationOSP      0.06874    0.69625   0.099   0.9216  
   ---
   Signif. codes:  0 '***' 0.001 '**' 0.01 '*' 0.05 '.' 0.1 ' ' 1
   
   Residual standard error: 1.793 on 86 degrees of freedom
     (2 observations deleted due to missingness)
   Multiple R-squared:  0.406,  Adjusted R-squared:  0.3369 
   F-statistic: 5.878 on 10 and 86 DF,  p-value: 1.041e-06
anova(LM.dN)
   Analysis of Variance Table
   
   Response: d15NLys
                  Df  Sum Sq Mean Sq F value    Pr(>F)    
   TPAla           1  77.655  77.655 24.1615 4.197e-06 ***
   Location        5 104.931  20.986  6.5297 3.413e-05 ***
   TPAla:Location  4   6.339   1.585  0.4931    0.7408    
   Residuals      86 276.402   3.214                      
   ---
   Signif. codes:  0 '***' 0.001 '**' 0.01 '*' 0.05 '.' 0.1 ' ' 1
coef.2 <- c(
  LM.dN$coefficients[1],
  LM.dN$coefficients[1] + LM.dN$coefficients[7],
  LM.dN$coefficients[1] + LM.dN$coefficients[8],
  LM.dN$coefficients[1] + LM.dN$coefficients[9],
  LM.dN$coefficients[1] + LM.dN$coefficients[10]
)
p2 <- anova(LM.dN)[3,5] 
R2 <- summary(LM.dN)$adj.r.squared


noquote("Now re-running the regression without location interaction")
   [1] Now re-running the regression without location interaction

noquote("Regressing d15N(Lys-Phe) against TP(Glx-Phe)")
   [1] Regressing d15N(Lys-Phe) against TP(Glx-Phe)
LM.dN = lm(d15NLys ~ (TPGlx-1) + Location, data = data.reg.phenorm)
summary(LM.dN)
   
   Call:
   lm(formula = d15NLys ~ (TPGlx - 1) + Location, data = data.reg.phenorm)
   
   Residuals:
       Min      1Q  Median      3Q     Max 
   -5.0089 -0.8599 -0.0002  1.1382  4.4527 
   
   Coefficients:
                   Estimate Std. Error t value Pr(>|t|)    
   TPGlx             1.3714     0.2799   4.900 4.27e-06 ***
   Location5 N      -2.9517     0.8682  -3.400  0.00101 ** 
   Location8 N      -3.5637     0.8528  -4.179 6.83e-05 ***
   LocationALOHA S  -1.3766     0.7180  -1.917  0.05840 .  
   LocationALOHA W  -0.8926     0.7104  -1.256  0.21224    
   LocationOSP      -2.7367     0.6341  -4.316 4.11e-05 ***
   ---
   Signif. codes:  0 '***' 0.001 '**' 0.01 '*' 0.05 '.' 0.1 ' ' 1
   
   Residual standard error: 1.774 on 89 degrees of freedom
     (3 observations deleted due to missingness)
   Multiple R-squared:  0.3983, Adjusted R-squared:  0.3577 
   F-statistic: 9.818 on 6 and 89 DF,  p-value: 2.734e-08
anova(LM.dN)
   Analysis of Variance Table
   
   Response: d15NLys
             Df  Sum Sq Mean Sq F value    Pr(>F)    
   TPGlx      1  72.657  72.657 23.0983 6.208e-06 ***
   Location   5 112.643  22.529  7.1621 1.131e-05 ***
   Residuals 89 279.953   3.146                      
   ---
   Signif. codes:  0 '***' 0.001 '**' 0.01 '*' 0.05 '.' 0.1 ' ' 1
coef.1 <- c(
  LM.dN$coefficients[1]
)
p3 <- anova(LM.dN)[1,5] 
R3 <- summary(LM.dN)$adj.r.squared


ggplot(data = data.reg.phenorm, aes(x = (TPGlx-1), y = d15NLys))+
  geom_point(aes(color=Location))+
  geom_smooth(method=lm, aes(y = predict(LM.dN, data.reg.phenorm), color=Location), lty=1, se=TRUE)+
  xlab(expression(TP[Glx-Phe]-1))+
  ylab(expression(delta^{15}*N[Lys-Phe]*" (\u2030)"))+
  theme_light()+
  facet_wrap(~ Location)
   `geom_smooth()` using formula = 'y ~ x'
   Warning: Removed 3 rows containing non-finite outside the scale range
   (`stat_smooth()`).
   Warning: Removed 3 rows containing missing values or values outside the scale range
   (`geom_point()`).
```

```
noquote("Regressing d15N(Lys-Phe) against TP(Ala-Phe)")
   [1] Regressing d15N(Lys-Phe) against TP(Ala-Phe)
LM.dN = lm(d15NLys ~ (TPAla-1) + Location, data = data.reg.phenorm)
summary(LM.dN)
   
   Call:
   lm(formula = d15NLys ~ (TPAla - 1) + Location, data = data.reg.phenorm)
   
   Residuals:
       Min      1Q  Median      3Q     Max 
   -5.1378 -0.9362  0.1396  1.0246  4.3368 
   
   Coefficients:
                   Estimate Std. Error t value Pr(>|t|)    
   TPAla             0.9510     0.1959   4.854 5.06e-06 ***
   Location5 N      -2.2892     0.7731  -2.961 0.003920 ** 
   Location8 N      -3.0229     0.7712  -3.920 0.000173 ***
   LocationALOHA S  -1.0088     0.6448  -1.565 0.121179    
   LocationALOHA W  -0.4577     0.6431  -0.712 0.478493    
   LocationOSP      -2.3313     0.5657  -4.121 8.37e-05 ***
   ---
   Signif. codes:  0 '***' 0.001 '**' 0.01 '*' 0.05 '.' 0.1 ' ' 1
   
   Residual standard error: 1.772 on 90 degrees of freedom
     (2 observations deleted due to missingness)
   Multiple R-squared:  0.3924, Adjusted R-squared:  0.3519 
   F-statistic: 9.687 on 6 and 90 DF,  p-value: 3.265e-08
anova(LM.dN)
   Analysis of Variance Table
   
   Response: d15NLys
             Df  Sum Sq Mean Sq F value    Pr(>F)    
   TPAla      1  77.655  77.655 24.7184 3.152e-06 ***
   Location   5 104.931  20.986  6.6802 2.465e-05 ***
   Residuals 90 282.742   3.142                      
   ---
   Signif. codes:  0 '***' 0.001 '**' 0.01 '*' 0.05 '.' 0.1 ' ' 1
coef.2 <- c(
  LM.dN$coefficients[1]
)
p4 <- anova(LM.dN)[1,5] 
R4 <- summary(LM.dN)$adj.r.squared

ggplot(data = data.reg.phenorm, aes(x = (TPAla-1), y = d15NLys))+
  geom_point(aes(color=Location))+
  geom_smooth(method=lm, aes(y = predict(LM.dN, data.reg.phenorm), color=Location), lty=1, se=TRUE)+
  xlab(expression(TP[Ala-Phe]-1))+
  ylab(expression(delta^{15}*N[Lys-Phe]*" (\u2030)"))+
  theme_light()+
  facet_wrap(~ Location)
   `geom_smooth()` using formula = 'y ~ x'
   Warning: Removed 2 rows containing non-finite outside the scale range
   (`stat_smooth()`).
   Warning: Removed 2 rows containing missing values or values outside the scale range
   (`geom_point()`).
```

```
DN.reg <- data.frame(
  "TP(Glx-Phe)" = coef.1,
  "TP(Ala-Phe)" = coef.2,
  row.names = "d15NLys"
)

DN.reg
           TP.Glx.Phe. TP.Ala.Phe.
   d15NLys    1.371432   0.9509821
```

1. We tested to see if location had a significant effect on the slope of the regression. The P values associated with this test were 0.7012771 and 0.7408017 for TP(Glx-Phe) and TP(Ala-Phe), respectively. This indicates that location DOES NOT have a significant effect on the slope of the regression.
2. Removing the interaction with location, we tested to see if the slope is significant. The P value associated with these tests were 6.207765^{-6} and 3.1518659^{-6}. This indicates that the slope of the regression IS significant.
3. The \(R^2\) values were 0.3577125 and 0.3518732, respectively.
