# Supplementary material for: A Bayesian model for assessing organic matter supply in complex marine food webs using amino acid stable isotope analysis
Source: PeerJ. 2025 Nov 19;13:e20220. doi: 10.7717/peerj.20220 (PMC12640130; doi:10.7717/peerj.20220)
Supplement: Supplemental Information 7 — These are also available at GitHub (https://github.com/CH-Shea/Organic-Matter-Supply-Model). [file peerj-13-20220-s007.zip › Supplemental Files/S7_OMSM_ALOHA_sim-zoops_no-Thr.html]

Organic Matter Supply Model: Assessing model performance using simulated data from Station ALOHA - excluding threonine


Code 

- Show All Code
- Hide All Code

# Organic Matter Supply Model: Assessing model performance using simulated data from Station ALOHA - excluding threonine

#### Connor Shea

#### May 09, 2025

# Summary

The purpose of this notebook is to visualize isotopic separation of organic matter sources at Station ALOHA, and then test the ability of an AA-CSIA-based organic matter supply model to diagnose the relative importance of those sources to the zooplankton food web. The results are diagnostic of the model’s efficacy in this setting, and should be used to inform the interpretation of the model’s output when applied to natural samples.

This notebook is composed of the following sections:

### Sections:

1. **Setup**: Input data and parameters. \*\* User input required \*\*
2. **Source Separation**: Multivariate analysis of source distinction.
3. **Data Simulation**: Generate synthetic zooplankton data.
4. **Model Execution**: Fit the Bayesian model and run diagnostics.
5. **Model Assessment**: Compare outputs to simulation parameters.

# 1 Setup

In the first few chunks, the user should enter all of the basic information that the model code will need to proceed with analysis.

## Importing Data

First let’s import the data. In this case we will be importing particle data from Station ALOHA.

```
## All data should be stored in one, single .xlsx file.
## See OMSM_Data_Template.xlsx in Data folder for guidance on data formatting

################################################################################
## IMPORTING DATA ##
# Define the location of .xlsx file, and sheet name if multiple sheets are  present.
Data.all <-
  read_excel(
    "Data/AA-CSIA_ALOHA.xlsx",
    sheet = "Data")
```

We will export model results to a .Rdata file in the Data folder following analysis. Indicate the desired file name in the following chunk.

```
export.file.name <- "ALOHAsim_OMSM_no-Thr_posteriors.RData"
```

## Identifying Tracers

Next we’ll identify tracers to be included in the model and some key parameters on how to treat them. Here we’ll be using \(\mathrm{\delta^{15}\text{N}}\) values of Phe and Lys. We’ll be treating Phe as a conservative tracer.

```
#### IDENTIFYING SOURCE AND DESCRIPTIVE VARIABLES ####

# Indicate the name of the column describing whether a row in the data is a source or consumer sample.
# This column will be renamed to "Type" if it isnt already
Type.Variable <- "Type"
colnames(Data.all[Type.Variable]) <- "Type"


# Indicate the name of the column describing the organic matter source to which each sample belongs.
# This column will be renamed to "Source"
Source.Variable <- "Source"
colnames(Data.all[Source.Variable]) <- "Source"

# Indicate the names representing each possible source of organic matter to the food web/consumer.
# These should match the names present in the Group column of your data.
# The order of this vector will describe the order these groups are referenced in figures.
# The model can accomodate 2-6 organic matter sources, but will need to be modified to accommodate more.
Sources <- c("Surface","Large","Small")#,"Submicron","DOM")
nsources <- length(Sources) # calculating number of source for convenience later


# List the name of any additional variables that should be stored for analysis.
# The data in these columns must be complete for all samples.
Descriptive.Variables <- c("Location","Epoch","Event","Size","Sizecat","Depth")
Variables <- c("Type","Source", Descriptive.Variables)

#### SPECIFYING TRACERS TO BE USED IN MODEL ####

# Tracer names should match column names in the data spreadsheet
# We'll store them in two lists
Tracers <- list()   ## LEAVE THESE LISTS EMPTY ##
SDTracers <- list() ## LEAVE THESE LISTS EMPTY ##

# TRACERS FOR TROPHIC EQUATIONS
# These tracers will not be used to solve mixing equations
# FWL: Specify the name of the tracer that will estimate the full food web length
Tracers$FWL <- 
  c("d15NPro")
SDTracers$FWL <- 
  c("SDd15NPro")
# MTS: Specify the name of the tracer that will estimate the number of metazoan trophic steps
Tracers$MTS <- 
  c("d15NGlx")
SDTracers$MTS <- 
  c("SDd15NGlx")

# TRACERS FOR MIXING EQUATIONS
# Tracers for trophic equations must not be included in this list
Tracers$mix <-
  c(
    # "d15NAla",
    # "d15NAsx",
    # "d13CLeu",
    # "d13CIle",
    # "d15NPro",
    # "d15NThr",
    # "d15NSer",
    # "d15NGly",
    "d15NLys",
    "d15NPhe"
  )
SDTracers$mix <-
  c(
    # "SDd15NAla",
    # "SDd15NAsx",
    # "SDd13CLeu",
    # "SDd13CIle",
    # "SDd15NPro",
    # "SDd15NThr",
    # "SDd15NSer",
    # "SDd15NGly",
    "SDd15NLys",
    "SDd15NPhe"
  )

# Specify the names for all tracers with constant trophic discrimination  and their uncertainty(i.e., TDF(protozoan) = TDF(metazoan))
Tracers$constTDF <- 
  c(
    # "d15NPhe",
    "d15NAla", 
    "d15NPro", 
    "d15NSer", 
    "d15NGly", 
    "d15NLys"
  )
SDTracers$constTDF <- 
  c(
    # "SDd15NPhe",
    "SDd15NAla", 
    "SDd15NPro", 
    "SDd15NSer", 
    "SDd15NGly", 
    "SDd15NLys"
  )

# Specify the names for all tracers with variable trophic discrimination  and their uncertainty(i.e., TDF(protozoan) != TDF(metazoan))
Tracers$varTDF <- 
  c(
    "d15NGlx", 
    "d15NAsx",
    "d15NThr"
  )
SDTracers$varTDF <- 
  c(
    "SDd15NGlx", 
    "SDd15NAsx",
    "SDd15NThr"
  )

# Specify the names for all conservative tracers and their uncertainty (i.e., no trophic discrimination is expected)
Tracers$non <- 
  c(
    "d15NPhe"
    # "d13CThr", 
    # "d13CVal", 
    # "d13CLeu", 
    # "d13CIle", 
    # "d13CPhe"
  )
SDTracers$non <- 
  c(
    "SDd15NPhe"
    # "SDd13CThr", 
    # "SDd13CVal", 
    # "SDd13CLeu", 
    # "SDd13CIle", 
    # "SDd13CPhe"
  )

#### SPECIFYING CHOICES FOR TDF(PROTOZOAN) AND TDF(METAZOAN) ####
# Note: default values are those used in Shea et al. (in prep)

## What TDFs and SDs should be used to describe amino acid δ15N fractionation in metazoans?
## Note that these TDFs should NOT be normalized to any source amino acid.
TDF_meta <- data.frame("d15NAla" = 6.3, "SDd15NAla" = 2.6,
                       "d15NGly" = 2.9, "SDd15NGly" = 3.1,
                       "d15NThr" =-5.9, "SDd15NThr" = 1.5,
                       "d15NSer" = 2.6, "SDd15NSer" = 3.2,
                       "d15NVal" = 4.4, "SDd15NVal" = 2.6,
                       "d15NLeu" = 5.6, "SDd15NLeu" = 2.4,
                       "d15NIle" = 5.5, "SDd15NIle" = 2.4,
                       "d15NPro" = 5.8, "SDd15NPro" = 1.7,
                       "d15NAsx" = 5.7, "SDd15NAsx" = 1.9,
                       "d15NMet" = 1.6, "SDd15NMet" = 2.6,
                       "d15NGlx" = 8.0, "SDd15NGlx" = 1.7,
                       "d15NPhe" = 0.3, "SDd15NPhe" = 0.5,
                       "d15NTyr" = NA , "SDd15NTyr" = NA,
                       "d15NLys" = 1.2, "SDd15NLys" = 1.2)

## What TDFs and SDs should be used to describe amino acid δ15N fractionation in protozoans?
# Note that these TDFs should NOT be normalized to any source amino acid.
TDF_proto = data.frame("d15NAla" = 6.3, "SDd15NAla" = 2.6,
                       "d15NGly" = 2.9, "SDd15NGly" = 3.1,
                       "d15NThr" =-2.0, "SDd15NThr" = 0.6,
                       "d15NSer" = 2.6, "SDd15NSer" = 3.2,
                       "d15NVal" = 0.7, "SDd15NVal" = 1.6,
                       "d15NLeu" = 1.4, "SDd15NLeu" = 0.6,
                       "d15NIle" =-0.5, "SDd15NIle" = 2.7,
                       "d15NPro" = 5.8, "SDd15NPro" = 1.7,
                       "d15NAsx" = 0.8, "SDd15NAsx" = 1.4,
                       "d15NMet" = NA , "SDd15NMet" = NA ,
                       "d15NGlx" = 0.5, "SDd15NGlx" = 1.0,
                       "d15NPhe" = 0.3, "SDd15NPhe" = 0.5,
                       "d15NTyr" = NA , "SDd15NTyr" = NA,
                       "d15NLys" = 1.2, "SDd15NLys" = 1.2)


################################################################################
## Making some master lists of tracers - no changes necessary here ##
# All tracers
Tracers$all <- 
  c(
    Tracers$constTDF,
    Tracers$varTDF,
    Tracers$non
  )
SDTracers$all <- 
  c(
    SDTracers$constTDF,
    SDTracers$varTDF,
    SDTracers$non
  )
# Tracers that fractionate
Tracers$frac <- 
  c(
    Tracers$constTDF,
    Tracers$varTDF
  )
SDTracers$frac <- 
  c(
    SDTracers$constTDF,
    SDTracers$varTDF
  )
# Ensuring that trophic tracers are not also mixing tracers
if(sum(Tracers$mix %in% c(Tracers$FWL,Tracers$MTS)) > 0) {
  stop("Trophic tracers should not be included in the list of mixing tracers.")
}
# Tracers that fractionate and will be used to fit the model
Tracers$mixfrac <- 
  Tracers$mix[which(Tracers$mix %in% Tracers$frac)]
SDTracers$mixfrac <- 
  SDTracers$mix[which(SDTracers$mix %in% SDTracers$frac)]
# we also need to bypass all multivariate analyses if <3 tracers
runMV <- TRUE
if(length(Tracers$mix) < 3){
  runMV <- FALSE
}

# Define the Order you would like tracers referenced in, if any
Order <- c("d15NGlx", "d15NAsx", "d15NAla", "d15NIle", "d15NLeu", "d15NPro", "d15NVal", 
           "d15NGly", "d15NSer", "d15NLys", "d15NPhe", "d15NThr", "SAA",
           "d13CGlx", "d13CAsx", "d13CAla", "d13CIle", "d13CLeu", "d13CPro", "d13CVal", 
           "d13CGly", "d13CSer", "d13CLys", "d13CPhe", "d13CThr", "EAA")
SDOrder <- c("SDd15NGlx", "SDd15NAsx", "SDd15NAla", "SDd15NIle", "SDd15NLeu", "SDd15NPro", "SDd15NVal", 
             "SDd15NGly", "SDd15NSer", "SDd15NLys", "SDd15NPhe", "SDd15NThr", "SDSAA",
             "SDd13CGlx", "SDd13CAsx", "SDd13CAla", "SDd13CIle", "SDd13CLeu", "SDd13CPro", "SDd13CVal", 
             "SDd13CGly", "SDd13CSer", "SDd13CLys", "SDd13CPhe", "SDd13CThr", "SDEAA")
# If all tracers used aren't specified in the Order vector then it will be redefined
if(length(which(Order %in% Tracers$all))<length(Tracers$all)){
  Order <- Tracers$all
  SDOrder <- SDTracers$all
}
```

## Processing Data

The next thing we will do is process the data. We will remove any samples for which the data is incomplete or any samples we would just like to otherwise exclude. We will also choose to programatically define source groups based on quantitative criteria.

```
#### Retaining only columns for variables defined in the above chunk ####
Data.all <- Data.all[c(Variables,Tracers$all,SDTracers$all)]

#### Removing samples with incomplete data ####
Data.all <- na.omit(Data.all)

#### Isolating organic matter source data ####
## Organic matter source data will be stored in a separate data frame
Data.sources <- subset(Data.all, Type == "Source")

#### Programatically defining organic matter source groups ####
## would you like to programatically define organic matter source groups?
if(TRUE){
  Data.sources$Source <- factor(NA, levels = Sources)
  Data.sources$Source[
    which(Data.sources$Depth < 100 & Data.sources$d15NPhe < 0)
  ] <- "Surface"
  Data.sources$Source[
    which(Data.sources$Depth > 190 & 
            (Data.sources$Sizecat == "Large" | Data.sources$Sizecat == "Trap"))
  ] <- "Large"
  Data.sources$Source[
    which(Data.sources$Depth > 190 & Data.sources$Sizecat == "Small")
  ] <- "Small"
  # Data.sources$Source[
  #   which(Data.sources$Depth > 190 & Data.sources$Sizecat == "Submicron")
  # ] <- "Submicron"
  # Data.sources$Source[
  #   which(Data.sources$Depth > 190 & Data.sources$Sizecat == "DOM")
  # ] <- "DOM"
  Data.sources <- subset(Data.sources, !is.na(Source))
}

# Defining the preferred Order in which to reference organic matter sources
Data.all$Source <- factor(Data.all$Source, levels = c(Sources,"Consumer"))

# To define the order that specific descriptive variables should be referenced, adapt the below example.
# Data.all["Size"] <- factor(Data.all[["Size"]], levels = c(
#   c("0.3-1 μm", "1-5 μm", "1-6 μm", "6-51 μm", ">51 μm", "Sediment Trap",
#   "0.2-0.5 mm", "0.5-1.0 mm", "1-2 mm", "2-5 mm", ">5 mm")
# ))
#
```

# 2 Source Separation

First lets visualize the value of each tracer in each organic matter source.

```
plot_sources(Data.sources, Tracers)
```

Next, to visualize the between group patterns in the data we will do some multivariate analyses. We’ll start by carrying out a PCA. This should give us a decent of idea of what kind of if between-group separation is a major component of variation in this data set. It will also help us visualize which tracers are driving separation between certain organic matter sources and which are providing redundant information.

```
# Fitting PCA and adding Sample and Type as a supplemental qualitative variable
PCA = PCA(Data.sources[c(Variables,Tracers$mix)], scale.unit = FALSE, quali.sup = Variables, graph = FALSE)

# ## Uncomment these lines to see some diagnostics of how the PCA ran
# # Plot component variance
# fviz_eig(PCA, addlabels = FALSE, geom = "bar")
# 
# # Print a summary of the PCA results
# summary(PCA)

plot_sources_PCA(PCA, Data.sources)
```

We’ll also fit an LDA since we will use this later to visualize zooplankton samples in our mixing space.

```
if(runMV == TRUE & length(Sources >2)){
  ntypes = nlevels(as.factor(Data.sources[["Source"]]))
  # fitting the model with leave one out cross validation
  LDA.test = lda(Source ~ . ,data = Data.sources[c("Source",Tracers$mix)], CV = TRUE,
                 prior = rep(1/ntypes, ntypes))
  
  
  
  # ## uncomment these lines to see the results of leave one out cross-validation 
  # ## and see some LDA model diagnostics
  # print model result
  # LDA.train
  
  # # create a table which compares the classification of the LDA model to the actual producer type
  # ct.prod.norm <- table(Data.sources[["Source"]], 
  #                       LDA.test$class)
  # # total percent of samples correctly classified is the sum of the diagonal of this table
  # noquote(c('% successfully categorized: ', sum(diag(prop.table(ct.prod.norm)))))
  
  
  
  # Refitting the model using all of the available training data
  LDA.full = lda(Source ~ . ,data = Data.sources[c("Source",Tracers$mix)], CV = FALSE, prior = rep(1/ntypes, ntypes))
  
  ## uncomment this line to see a summary of the full LDA
  # LDA.full
  
  # store locations of training data in LD space for later plotting
  pred.train = predict(LDA.full, Data.sources[Tracers$mix])
  class.train = data.frame("Source" = Data.sources[["Source"]], pred.train$x)
  
  
  plot_sources_LDA(class.train)
}
```

# 3 Data Simulation

Next we will simulate some zooplankton data as a way to test the efficacy of the model under different ecological scenarios. We will simulate it using known ecological parameters and the same TDFs that will be supplied to the food web model, except for Phe which will undergo 0.3‰ trophic discrimination during simulation even though its treated as conservative in the model. If the organic matter source data provides adequate separation of mixing end members and the sources of uncertainty are well enough constrained then the model should return parameters similar to those defined in the following chunk.

For Station ALOHA, we will simulate 50 sample with random mixing coefficients. PTS will vary at random from 0 to 1 and MTS from 1 to 2, resulting in FWLs from 1 to 3.

```
## Sim_Zoop uses the exact same TDFs, and makes the same assumptions about conservative
## and fractionating tracers, as defined in the identifying_tracers chunk

## Sim_Zoop_RealPhe uses 0.3‰ trophic discrimination in Phe to simulate zooplankton
## data even if the model will assume it is conservative.

Sim_Zoop_RealPhe( # use this if treating Phe as a conservative tracer in the model
  # Sim_Zoop( # use this if treating Phe as a fractionating tracer in the model
  Random_Samples = TRUE,
  nzoops = 50, # If Random_Samples = TRUE define number of samples
  incr = 0.2, # if Random_Samples = FALSE define spacing of samples in compositional space
  PTS = c(0,0.5,1), # If Random_Samples = TRUE, random values for PTS between min and max in this vector are generated
  # If Random_samples = FALSE, samples are generated at all PTS values given
  MTS = c(1,1.5,2), # If Random_Samples = TRUE, random values for MTS between min and max in this vector are generated
  # If Random_samples = FALSE, samples are generated at all MTS values given
  TDF_m = TDF_meta[Tracers$frac], # trophic discrimination factors
  TDF_p = TDF_proto[Tracers$frac], # trophic discrimination factors
  Sources = Sources,
  Data.sources = Data.sources,
  Tracers = Tracers,
  Variables = Variables,
  disperse = 0.5,
  seed = 000
)
```

## Plotting Simulated Data

Let’s plot out the tracer values of organic matter sources and zooplankton.

```
plot_Source_Consumer_Sim(
  Data.sources,
  Data.zoops,
  base.sim,
  Tracers, 
  LDA.full)
```

# 4 Model Execution

Next, we will use Markov Chain Monte Carlo to find PDFs describing the most likely solutions to our mixing problem. We will write a BUGS model, then use JAGS to do the MCMC.

## Setting up for MCMC

Organizing the input data for MCMC.

```
Data.OMSM <- 
  OMSM_Datalist(
    Data.sources, 
    Data.zoops, 
    Sources, 
    Tracers, 
    TDF_meta, 
    TDF_proto
    )
```

and defining our initial values.

```
Inits.OMSM <- 
  OMSM_Initlist(
    seed = 222,
    Nchains = 3,
    nzoops,
    nsources,
    length(Tracers$all)
    )
```

## Drafting a BUGS model

Now we’ll define our BUGS model.

```
# Generate the BUGS model. No edits typically needed unless modifying model structure.

OMSM <- 
  OMSM_Gen_Model(
    nsources, 
    Data.OMSM
    )
```

## Running MCMC in JAGS

Now we can run the MCMC!

```
samsPerChain <- 1000 # needed  below for narrative, so we give it a name.
monitor_vars = c("mean_b", "mean_z",
                 "TDF_meta", "sdTDF_m",
                 "TDF_proto","sdTDF_p",
                 "pz"       , "FWL"  , "PTS", "MTS",
                 c("mean_A", "mean_B", "mean_C", "mean_D", "mean_E", "mean_F")[1:length(Sources)],
                 c("sd_A", "sd_B", "sd_C", "sd_D", "sd_E", "sd_F")[1:length(Sources)]
)

rjo_1 <- # S3 object of class "runjags"
  run.jags(model = OMSM,
           data = Data.OMSM,
           inits = Inits.OMSM,
           silent.jags = FALSE,
           n.chains = Nchains,
           adapt  = 5000,
           burnin = 10000,
           thin = 10,
           sample = samsPerChain,
           method = "parallel",
           modules = "glm",
           monitor = monitor_vars
  )
   Calling 3 simulations using the parallel method...
   Following the progress of chain 1 (the program will wait for all chains
   to finish before continuing):
   Welcome to JAGS 4.3.1 on Fri May  9 16:17:54 2025
   JAGS is free software and comes with ABSOLUTELY NO WARRANTY
   Loading module: basemod: ok
   Loading module: bugs: ok
   . Loading module: glm: ok
   . . Reading data file data.txt
   . Compiling model graph
      Resolving undeclared variables
      Allocating nodes
   Graph information:
      Observed stochastic nodes: 361
      Unobserved stochastic nodes: 204
      Total graph size: 6843
   
   WARNING: Unused variable(s) in data table:
   sdX_A
   sdX_B
   sdX_C
   
   . Reading parameter file inits1.txt
   . Initializing model
   . Adapting 5000
   -------------------------------------------------| 5000
   ++++++++++++++++++++++++++++++++++++++++++++++++++ 100%
   Adaptation successful
   . Updating 10000
   -------------------------------------------------| 10000
   ************************************************** 100%
   . . . . . . . . . . . . . . . . . Updating 10000
   -------------------------------------------------| 10000
   ************************************************** 100%
   . . . . Updating 0
   . Deleting model
   . 
   All chains have finished
   Simulation complete.  Reading coda files...
   Coda files loaded successfully
   Note: Summary statistics were not produced as there are >50 monitored
   variables
   [To override this behaviour see ?add.summary and ?runjags.options]
   FALSEFinished running the simulation
```

## Diagnostics

Here we run some diagnostics. Initially we use John K. Kruschke’s function `diagMCMC` to check out model parameters and make sure things are running properly. When these diagnostics look good, we set `eval=FALSE` in the chunk header and make nicer parameter plots comparing known parameter values and the MCMC posteriors for those parameters, which is seen below.

```
## Kruschke's utility functions (edited by NF)
# edit "parname" to print additional parameter diagnostics as desired
# numbers in hard brackets pertain to samples and tracers
if (c(TRUE,FALSE)[1]) source("Utilities/DBDA2E-utilities.R") 
diagMCMC(rjo_1$mcmc, parName="FWL[1]")
diagMCMC(rjo_1$mcmc, parName="MTS[1]")
diagMCMC(rjo_1$mcmc, parName="PTS[1]")
diagMCMC(rjo_1$mcmc, parName="pz[1,1]")
diagMCMC(rjo_1$mcmc, parName="pz[1,2]")
diagMCMC(rjo_1$mcmc, parName="pz[1,3]")
# diagMCMC(rjo_1$mcmc, parName="pz[1,4]")
diagMCMC(rjo_1$mcmc, parName="mean_z[1,9]")
```

# 5 Model Assessment

Now let’s gather up posteriors from the OMSM and plot those relative to the tue values we used to simulate the data to get an idea for how the model is performing.

```
OMSM_Extract_Posts(
  rjo_1 = rjo_1,
  Data.zoops = Data.zoops,
  Sources,
  Variables,
  Tracers
)
```

## Source tracer values

First we’ll generate some plots of the mean source tracer value posteriors (\(\mu\_{j,i}\)) relative to the original source data.

```
plot_sourcepost_sim(posts.long, Data.sources, Tracers)
   Warning: Using `size` aesthetic for lines was deprecated in ggplot2 3.4.0.
   ℹ Please use `linewidth` instead.
   This warning is displayed once every 8 hours.
   Call `lifecycle::last_lifecycle_warnings()` to see where this warning was
   generated.
```

## Basal tracer values

Now let’s generate plots of the MCMC posteriors for the zooplankton sample-specific parameters. We’ll want to plot the model posteriors for the tracer values at the base of the food web. We’ll do this for all the individual tracers, but also try and visualize the multivariate patterns using LDA. Note that trophic and mixing tracers are plotted on the left, though trophic tracers were not used to fit the mixing model and are not included in LDA on the right.

```
plot_basepost_sim(posts, posts.long, Data.sources, Data.zoops, base.sim, Tracers, Variables, LDA.full)
```

## Consumer tracer values

We also want to look at the mean\_z parameter for each zooplankton sample to see if and where the posteriors for mixing tracers are deviating from the data. If they are, that means either the TDFs are not accurate or there is a missing organic mater source.

```
plot_zooppost_sim(
  posts.long,  
  Data.zoops, 
  Data.sources,
  base.sim,
  Tracers
)
```

## Trophic parameters

We also want to plot comparisons of the trophic parameters used to simulate the zooplankton data and the model posteriors for those parameters. Posterior HDIs (95%, 90%, 75%, and 50%) are shown in blue bars, with posterior modes indicated with text. True values are indicated by yellow lines. Righthand plots show relationships between true and modeled values, with discrepancy plots showing the difference between modeled and true values.

```
plot<-
plotall_trophicpost_sim(posts, zoops.f)
   No id variables; using all as measure variables
   No id variables; using all as measure variables
plot
```

We’ll briefly quantify model error as well by looking at the discrepancy between modeled and true trophic parameter values. Expand the code chunk to see the mean and maximum error rates for each parameter.

```
Post_Error_Quant_Trophic(
  zoops.f,
  posts
)
   [1] "Mean error rates associated with each trohic parameter:"
          PTS        MTS        FWL 
   0.13919392 0.09053542 0.16223810 
   [1] "maximum error - PTS"
   [1] 0.3187045
   [1] "maximum error - MTS"
   [1] 0.2554363
   [1] "maximum error - FWL"
   [1] 0.4092299

plot_Post_Error_Trophic(
  zoops.f,
  posts
)
   No id variables; using all as measure variables
   No id variables; using all as measure variables
   [[1]]
```

```
   [[2]]
```

## Mixing coefficients

Last we’ll do the same for mixing coefficients.

```
aspect = length(Sources)/6
```

```
plot <- plotall_fpost_sim(posts, zoops.f, Sources)
   Warning: `aes_string()` was deprecated in ggplot2 3.0.0.
   ℹ Please use tidy evaluation idioms with `aes()`.
   ℹ See also `vignette("ggplot2-in-packages")` for more information.
   This warning is displayed once every 8 hours.
   Call `lifecycle::last_lifecycle_warnings()` to see where this warning was
   generated.
   No id variables; using all as measure variables
   No id variables; using all as measure variables
   Warning in eval(family$initialize): non-integer #successes in a binomial glm!
   Warning in eval(family$initialize): non-integer #successes in a binomial glm!
   Warning in eval(family$initialize): non-integer #successes in a binomial glm!
   Warning in eval(family$initialize): non-integer #successes in a binomial glm!
   Warning in eval(family$initialize): non-integer #successes in a binomial glm!
   Warning in eval(family$initialize): non-integer #successes in a binomial glm!
plot
```

We’ll also quantify model accuracy for mixing coefficients. Expand the code chunk to see the mean and maximum error rates for each source.

```
Post_Error_Quant_f(
    zoops.f,
    posts
)
   [1] "Mean error rates associated with each mixing parameter:"
      Surface      Large      Small 
   0.08680924 0.20202707 0.14679025 
   [1] "maximum error - Surface particles:"
   [1] 0.2795733
   [1] "maximum error - Large particles:"
   [1] 0.6698853
   [1] "maximum error - Small particles:"
   [1] 0.537626

plot_Post_Error_f(
    zoops.f,
    posts,
    Sources
)
   No id variables; using all as measure variables
   No id variables; using all as measure variables
   [[1]]
```

```
   [[2]]
```

# Exporting results

The last thing we want to do here is export out mixing model results to a .RData file. Load this file into other R scripts to do additional analyses.

```
save(posts,
     posts.long,
     Data.all,
     zoops.sim,
     file = paste0("Data/",export.file.name))
```

# Comparing to model results including Thr

Now we’d like to build a couple simple plots illustrating how including/excluding a trophic AA like Thr impact our ability to accurately solve the mixing problem.

```
data.true <- zoops.f[Sources]
data.model <- posts$f$mode[Sources]
# data.model$Model <- "OMSM - no Thr"
# data.model$Model <- factor(data.model$Model, levels = c("OMSM - full","OMSM - no Thr"))


mix.disc <- data.model-data.true
mix.disc$Model <- "OMSM - no Thr"
mix.disc$Model <- factor(mix.disc$Model, levels = c("OMSM - full","OMSM - no Thr"))

data.model.long <- 
  melt(data.model[Sources], 
       value.name = "f_model",
       variable.name = "Source"
  )
   No id variables; using all as measure variables
data.true.long <- 
  melt(data.true[Sources], 
       value.name = "f_true",
       variable.name = "Source"
  )
   No id variables; using all as measure variables
data.truemod.long <-
  cbind(
    data.true.long,
    data.model.long["f_model"]
  )
data.truemod.long$f_disc <-
  data.truemod.long$f_model -
  data.truemod.long$f_true


load("Data/ALOHAsim_OMSM_posteriors.RData")
posts.OMSM2 <- posts
posts.OMSM2.long <- posts.long

data.true <- zoops.f[c("Surface","Large","Small")]
data.model.full <- 
  cbind(
    posts.OMSM2$f$mode[c("Surface","Large","Small")]
  )

mix.disc.full <- data.model.full-data.true
mix.disc.full$Model <- "OMSM - no Thr" # posts.mode$Model
mix.disc.full$Model <- factor(mix.disc.full$Model, levels = c("OMSM - full","OMSM - no Thr"))


data.model.full.long <- 
  melt(data.model.full[Sources], 
       value.name = "f_model",
       variable.name = "Source"
  )
   No id variables; using all as measure variables
data.true.long <- 
  melt(data.true[Sources], 
       value.name = "f_true",
       variable.name = "Source"
  )
   No id variables; using all as measure variables
data.truemod.full.long <-
  cbind(
    data.true.long,
    data.model.full.long["f_model"]
  )
data.truemod.full.long$f_disc <-
  data.truemod.full.long$f_model -
  data.truemod.long$f_true


plot.TM.surf <-
  ggplot()+
  geom_smooth(data = subset(data.truemod.long, Source == "Surface"),
    aes(x = f_true, y = f_model, color = "Without Threonine"),
        fill = "salmon",
    # method = "lm"
    method = "glm", method.args = list(family = binomial())
  )+
  geom_smooth(data = subset(data.truemod.full.long, Source == "Surface"),
    aes(x = f_true, y = f_model, color = "Full Model"),
        fill = "skyblue1",
    # method = "lm"
    method = "glm", method.args = list(family = binomial())
  )+
  geom_point(data = subset(data.truemod.long, Source == "Surface"),
    aes(x = f_true, y = f_model),
    size = 1, stroke=1, shape = 1, color = "red4"
  )+
  geom_point(data = subset(data.truemod.full.long, Source == "Surface"),
    aes(x = f_true, y = f_model),
    size = 1, stroke=1, shape = 1, color = "steelblue4"
  )+
  scale_color_manual(values = c("royalblue4","red3"))+
  labs(color = "")+
  geom_abline(slope = 1, intercept = 0, size = 1, color = "goldenrod")+
  coord_cartesian(ylim = c(0,1), xlim = c(0,1), expand = FALSE)+
  scale_x_continuous(breaks = c(0,0.5,1))+
  scale_y_continuous(breaks = c(0,0.5,1))
# plot.TM.surf

plot.TM.large <-
  ggplot()+
  geom_smooth(data = subset(data.truemod.long, Source == "Large"),
    aes(x = f_true, y = f_model, color = "Without Threonine"),
        fill = "salmon",
    # method = "lm"
    method = "glm", method.args = list(family = binomial())
  )+
  geom_smooth(data = subset(data.truemod.full.long, Source == "Large"),
    aes(x = f_true, y = f_model, color = "Full Model"),
        fill = "skyblue1",
    # method = "lm"
    method = "glm", method.args = list(family = binomial())
  )+
  geom_point(data = subset(data.truemod.long, Source == "Large"),
    aes(x = f_true, y = f_model),
    size = 1, stroke=1, shape = 1, color = "red4"
  )+
  geom_point(data = subset(data.truemod.full.long, Source == "Large"),
    aes(x = f_true, y = f_model),
    size = 1, stroke=1, shape = 1, color = "steelblue4"
  )+
  scale_color_manual(values = c("royalblue4","red3"))+
  labs(color = "")+
  geom_abline(slope = 1, intercept = 0, size = 1, color = "goldenrod")+
  coord_cartesian(ylim = c(0,1), xlim = c(0,1), expand = FALSE)+
  scale_x_continuous(breaks = c(0,0.5,1))+
  scale_y_continuous(breaks = c(0,0.5,1))
# plot.TM.large

plot.TM.small <-
  ggplot()+
  geom_smooth(data = subset(data.truemod.long, Source == "Small"),
    aes(x = f_true, y = f_model, color = "Without Threonine"),
        fill = "salmon",
    # method = "lm"
    method = "glm", method.args = list(family = binomial())
  )+
  geom_smooth(data = subset(data.truemod.full.long, Source == "Small"),
    aes(x = f_true, y = f_model, color = "Full Model"),
        fill = "skyblue1",
    # method = "lm"
    method = "glm", method.args = list(family = binomial())
  )+
  geom_point(data = subset(data.truemod.long, Source == "Small"),
    aes(x = f_true, y = f_model),
    size = 1, stroke=1, shape = 1, color = "red4"
  )+
  geom_point(data = subset(data.truemod.full.long, Source == "Small"),
    aes(x = f_true, y = f_model),
    size = 1, stroke=1, shape = 1, color = "steelblue4"
  )+
  scale_color_manual(values = c("royalblue4","red3"))+
  labs(color = "")+
  geom_abline(slope = 1, intercept = 0, size = 1, color = "goldenrod")+
  coord_cartesian(ylim = c(0,1), xlim = c(0,1), expand = FALSE)+
  scale_x_continuous(breaks = c(0,0.5,1))+
  scale_y_continuous(breaks = c(0,0.5,1))
# plot.TM.small

plot.disc.surf <-
  ggplot()+
  geom_smooth(data = subset(data.truemod.long, Source == "Surface"),
    aes(x = f_true, y = f_disc, color = "Without Threonine"),
        fill = "salmon",
    method = "lm"
    # method = "glm", method.args = list(family = binomial())
  )+
  geom_smooth(data = subset(data.truemod.full.long, Source == "Surface"),
    aes(x = f_true, y = f_disc, color = "Full Model"),
        fill = "skyblue1",
    method = "lm"
    # method = "glm", method.args = list(family = binomial())
  )+
  geom_point(data = subset(data.truemod.long, Source == "Surface"),
    aes(x = f_true, y = f_disc),
    size = 1, stroke=1, shape = 1, color = "red4"
  )+
  geom_point(data = subset(data.truemod.full.long, Source == "Surface"),
    aes(x = f_true, y = f_disc),
    size = 1, stroke=1, shape = 1, color = "steelblue4"
  )+
  scale_color_manual(values = c("royalblue4","red3"))+
  labs(color = "")+
  geom_abline(slope = 0, intercept = 0, size = 1, color = "goldenrod")+
  coord_cartesian(ylim = c(-1,1), xlim = c(0,1), expand = FALSE)+
  scale_x_continuous(breaks = c(-1,-0.5,0,0.5,1))+
  scale_y_continuous(breaks = c(-1,-0.5,0,0.5,1))
# plot.disc.surf

plot.disc.large <-
  ggplot()+
  geom_smooth(data = subset(data.truemod.long, Source == "Large"),
    aes(x = f_true, y = f_disc, color = "Without Threonine"),
        fill = "salmon",
    method = "lm"
    # method = "glm", method.args = list(family = binomial())
  )+
  geom_smooth(data = subset(data.truemod.full.long, Source == "Large"),
    aes(x = f_true, y = f_disc, color = "Full Model"),
        fill = "skyblue1",
    method = "lm"
    # method = "glm", method.args = list(family = binomial())
  )+
  geom_point(data = subset(data.truemod.long, Source == "Large"),
    aes(x = f_true, y = f_disc),
    size = 1, stroke=1, shape = 1, color = "red4"
  )+
  geom_point(data = subset(data.truemod.full.long, Source == "Large"),
    aes(x = f_true, y = f_disc),
    size = 1, stroke=1, shape = 1, color = "steelblue4"
  )+
  scale_color_manual(values = c("royalblue4","red3"))+
  labs(color = "")+
  geom_abline(slope = 0, intercept = 0, size = 1, color = "goldenrod")+
  coord_cartesian(ylim = c(-1,1), xlim = c(0,1), expand = FALSE)+
  scale_x_continuous(breaks = c(-1,-0.5,0,0.5,1))+
  scale_y_continuous(breaks = c(-1,-0.5,0,0.5,1))
# plot.disc.large

plot.disc.small <-
  ggplot()+
  geom_smooth(data = subset(data.truemod.long, Source == "Small"),
    aes(x = f_true, y = f_disc, color = "Without Threonine"),
        fill = "salmon",
    method = "lm"
    # method = "glm", method.args = list(family = binomial())
  )+
  geom_smooth(data = subset(data.truemod.full.long, Source == "Small"),
    aes(x = f_true, y = f_disc, color = "Full Model"),
        cfill = "skyblue1",
    method = "lm"
    # method = "glm", method.args = list(family = binomial())
  )+
  geom_point(data = subset(data.truemod.long, Source == "Small"),
    aes(x = f_true, y = f_disc),
    size = 1, stroke=1, shape = 1, color = "red4"
  )+
  geom_point(data = subset(data.truemod.full.long, Source == "Small"),
    aes(x = f_true, y = f_disc),
    size = 1, stroke=1, shape = 1, color = "steelblue4"
  )+
  scale_color_manual(values = c("royalblue4","red3"))+
  labs(color = "")+
  geom_abline(slope = 0, intercept = 0, size = 1, color = "goldenrod")+
  coord_cartesian(ylim = c(-1,1), xlim = c(0,1), expand = FALSE)+
  scale_x_continuous(breaks = c(-1,-0.5,0,0.5,1))+
  scale_y_continuous(breaks = c(-1,-0.5,0,0.5,1))
   Warning in geom_smooth(data = subset(data.truemod.full.long, Source ==
   "Small"), : Ignoring unknown parameters: `cfill`
# plot.disc.small


ggarrange(
  ggplot() + geom_blank() + ggtitle("Modeled\nvs True") + 
    theme(plot.title = element_text(hjust = 0.65)),
  ggplot() + geom_blank() + ggtitle("Disrepancy") + 
    theme(plot.title = element_text(hjust = 0.55)),
  
  plot.TM.surf + no.x.axis + ylab("f(surface)"), 
  plot.disc.surf + no.x.axis + 
    theme(axis.title.y = element_blank()), 
  
  plot.TM.large + no.x.axis + ylab("f(large)"), 
  plot.disc.large + no.x.axis + 
    theme(axis.title.y = element_blank()),
  
  plot.TM.small + ylab("f(small)") + xlab("True Value"),
  plot.disc.small + xlab("True Value") +
    theme(axis.title.y = element_blank()),
  
  ncol = 2, nrow = 4,
  heights = c(0.4,1,1,1.2),
  widths = c(1.1,1),
  common.legend = TRUE,
  legend = "bottom"
)
   `geom_smooth()` using formula = 'y ~ x'
   Warning in eval(family$initialize): non-integer #successes in a binomial glm!
   `geom_smooth()` using formula = 'y ~ x'
   Warning in eval(family$initialize): non-integer #successes in a binomial glm!
   `geom_smooth()` using formula = 'y ~ x'
   Warning in eval(family$initialize): non-integer #successes in a binomial glm!
   `geom_smooth()` using formula = 'y ~ x'
   Warning in eval(family$initialize): non-integer #successes in a binomial glm!
   `geom_smooth()` using formula = 'y ~ x'
   `geom_smooth()` using formula = 'y ~ x'
   `geom_smooth()` using formula = 'y ~ x'
   Warning in eval(family$initialize): non-integer #successes in a binomial glm!
   `geom_smooth()` using formula = 'y ~ x'
   Warning in eval(family$initialize): non-integer #successes in a binomial glm!
   `geom_smooth()` using formula = 'y ~ x'
   `geom_smooth()` using formula = 'y ~ x'
   `geom_smooth()` using formula = 'y ~ x'
   Warning in eval(family$initialize): non-integer #successes in a binomial glm!
   `geom_smooth()` using formula = 'y ~ x'
   Warning in eval(family$initialize): non-integer #successes in a binomial glm!
   `geom_smooth()` using formula = 'y ~ x'
   `geom_smooth()` using formula = 'y ~ x'
```
